# Quality of life perceptions amongst patients co-infected with Visceral Leishmaniasis and HIV: A qualitative study from Bihar, India

**DOI:** 10.1371/journal.pone.0227911

**Published:** 2020-02-10

**Authors:** Mohit Nair, Pragya Kumar, Sanjay Pandey, Shahwar Kazmi, Laura Moreto-Planas, Alok Ranjan, Sakib Burza

**Affiliations:** 1 Médecins Sans Frontières, New Delhi, India; 2 All India Institute of Medical Sciences, Patna, India; 3 Médecins Sans Frontières, Barcelona, Spain; Queensland University of Technology, AUSTRALIA

## Abstract

**Background:**

Co-infection with Visceral Leishmaniasis (commonly known as Kala Azar, KA) and Human Immunodeficiency Virus (HIV) is increasingly being diagnosed among patients in Bihar. This qualitative study is the first assessment of self-reported quality of life among patients co-infected with KA-HIV in the Asian context.

**Methods:**

We conducted semi-structured, in-depth interviews and adopted an inductive thematic analysis to generate evidence on the quality of life of patients co-infected with KA and HIV. Patients were purposively sampled until saturation was attained.

**Results:**

We found that patients highly valued income or livelihood potential and health as indicators of a good quality life, and routinely went into debt accessing care in the private setup. This was due to perceptions of poor quality of care in the government setup and a lack of knowledge regarding available government services at the district level. KA symptoms were often misdiagnosed in the private sector as seasonal fever, while care providers found it difficult to disentangle the clinical symptoms of KA and HIV; hence, patients presented late to district hospitals. Patients perceived a high level of stigma, largely due to their HIV status, and routinely reported that HIV had “destroyed” their life.

**Conclusions:**

Inadequate social support and referral pathways that were not conducive to patient needs negatively impacted patients’ quality of life. The dual burden of poverty interacting with the severity and chronicity of KA-HIV co-infection means financial support, increased community engagement, and collaborative decision making are crucial for co-infected patients. Increased provider awareness of co-infection and effective stigma-reduction interventions should be integrated to ensure that appropriate and effective access to care is possible for this vulnerable population. A sustainable long-term strategy requires a people-centered approach wherein the perceptions and life circumstances of patients are taken into account in the medical decision making process.

## Background

Globally, 36.7 million people were living with Human Immunodeficiency Virus (HIV)/Acquired Immunodeficiency Syndrome (AIDS) worldwide at the end of 2016 [1]. India alone has over 2.1 million people living with HIV/AIDS (PLHIV) and 80,000 new infections as of 2016 [2]. Within India, the state of Bihar is the third most populous state, with over 110 million people. Despite a relatively low HIV prevalence (0.21–0.33%) within the state, Bihar’s high population density indicates that up to an estimated 300,000 people in the state live with HIV/AIDS and the rate of new HIV infections is increasing [3, 4]. Since Bihar is endemic for both HIV and Kala Azar (KA), there have been an increasing number of reported patients suffering from both diseases [5].

KA is caused by the protozoan parasite L. donovani and is endemic to Bihar, which accounts for 80% of India’s total registered cases as of 2018 [6]. The disease causes several symptoms ranging from prolonged fever, anorexia, hepatosplenomegaly with anemia, darkening of the skin, and loss of weight [7, 8]. Leishmania amastigotes have developed survival strategies which are enhanced by HIV co-infection [9] and accelerate progression of disease [10]. The result of infection with KA in patients living with HIV is typically a rapid deterioration in symptoms, and a more severe, accelerated, atypical and refractory form of the disease [11,12]. Even post treatment for KA, relapse is the rule rather than the exception. Evidence on the prevalence of HIV in patients with KA in India is scarce, although estimates range from 2–6% [13]. Following elimination efforts, the overall caseload of KA is decreasing, but the proportion of KA cases co-infected with HIV is steadily rising, reaching 20% of reported adult KA cases in some highly endemic districts of Bihar [11–17, 18].

KA tends to affect the poorest and most vulnerable segments of the population, and HIV leaves patients even more vulnerable to economic hardships [19–20]. The economic toll of KA on households can be significant by itself: one study from Nepal found that average total costs incurred per episode of KA were higher than the median annual per capita income, and six out of seven affected households had to either take out loans or sell part of their livestock to meet care needs [21]. Furthermore, since co-infected patients often face a delayed diagnosis of KA, mortality and relapse are higher in comparison to non-HIV patients: Lindoso et al reported mortality ranging from 8.7–23.5% and relapses between 10–56% of co-infected cases [22–23].

Despite the emerging pattern of KA-HIV co-infection, there have been limited studies analyzing the presentation of co-infection among patients in India, and no qualitative studies looking at patient perceptions of quality of life in the Asian context. Although commonly seen in PLHIV, diagnosis of TB in patients with HIV-Kala Azar co-infection has been recently identified as an emerging problem, affecting up to 20% of patients with HIV and Kala Azar [24–27].

The Indian healthcare delivery system is structured in three tiers: primary health centers (PHCs) and block primary health centers (BPHCs) serve as the first point of contact in rural areas, district and sub-divisional hospitals constitute secondary care, and state-level research institutions and medical colleges constitute the tertiary level of care. Medicines and consultations are provided free of cost at the primary healthcare level, but the majority of patients prefer to access care in the private healthcare system. HIV care and treatment is provided free-of-cost at district hospitals, and patients must obtain care from antiretroviral therapy (ART) centers located within the district hospital closest to their place of residence. Patients typically make monthly visits to ART centers to pick up a monthly stock of medications. These visits also serve as a check-in for counseling and medication adherence. CD4 and viral load testing usually occurs every 6 months rather than on a monthly basis. As for KA, a national initiative to eliminate the disease has been in operation in India since 2010 [28]. The KA elimination program is sponsored by the Central Government, which provides funding for the costs of insecticides, diagnostic kits, drugs, and other cost of operations, but many district hospitals outside endemic belts lack the capacity to diagnose KA [28]. The care-seeking behavior and quality of life among patients co-infected with KA-HIV has not been documented anywhere in Asia.

The main objectives of the study were to explore perceptions of quality of life among patients co-infected with KA and HIV and to identify which factors patients deem to be most important when conceptualizing a “good quality of life.”

## Materials and methods

The study utilized semi-structured, in-depth interviews as its method of inquiry. Broad domains of “quality of life” were included in the interview guide based on a detailed literature review, but interviewers retained the flexibility to draw on new areas of inquiry and patient-driven conceptions of domains related to quality of life as they emerged.

### Study population and recruitment

In Bihar, Medecins Sans Frontieres (MSF) has been treating patients co-infected with KA and HIV since 2007. Patients co-infected with HIV who had recently completed treatment for KA were identified and interviewed at the All India Institute of Medical Sciences (AIIMS) facility in Patna. Patients were eligible for participation in the study if they were:

- ≥18 years of age
- Clinically stable and willing to provide informed consent
- HIV positive
- Recent episode of Kala Azar in the last 3 months, which had been treated

We only included patients who had a recent episode of Kala Azar in the last 3 months in order to minimize potential recall bias. The research team obtained written informed consent from all participants before interviews commenced. Participants also consented to audio recordings of interviews, and all participants were provided a token amount for lost daily wages.

### Data collection

The research team was comprised of four interviewers who were fluent in a variety of local languages, including Hindi, Maithli and Bhojpuri. Following the informed consent procedure, the interviewers conducted semi-structured, face-to-face interviews: the first few questions were intentionally generic in nature, in order to build a rapport with the patient, before transitioning to questions related to the patient’s illness and perceptions of quality of life. A maximum of two interviewers were present in each interview in order to minimize any prevailing power dynamics which may make it more difficult for the patient to participate openly. For female participants, a female interviewer led the interviews. The lead interviewer trained all interviewers in asking questions in an open-ended manner without using any leading questions or suggestive body language in order to minimize bias. All interviews were audio-recorded with the consent of the interviewee. In addition, handwritten notes were taken by the interviewers during the interview which were compared after the interviews to debrief after each interview. All audio-recordings were subsequently transcribed and translated (from Hindi, Bhojpuri, or Maithli) into English, before being entered into NVIVO 11 qualitative data analysis software for coding and analysis. Since the research team was also fluent in these languages, sections of the transcripts were back-translated at random to ensure that data translation was performed accurately. The duration of interviews ranged from 15 minutes to 50 minutes, depending on the availability of the respondent. All interviews took place over a period of one month. There were no incomplete interviews and all respondents consented to be audio-recorded for the duration of the interview. 33 participants were invited to participate in the interviews, and four participants refused to participant, yielding a response rate of 88%. Data collection and analysis were conducted concurrently, allowing inconsistencies in the nature of inquiry to be rectified as they emerged organically. The interview guide was adapted in an iterative manner to draw upon emergent themes. Patients were purposively sampled until saturation was attained (i.e. the point at which no new insights emerge from the data).

### Data analysis

Following data transcription, translation, and entry into NVIVO software, two of the interviewers read and re-read transcripts several times. As categories and themes started to emerge, the researchers independently engaged in an inductive analysis by following the steps of open coding and identifying categories pertaining to quality of life, and analyzing patterns and relationships between categories. Once a coding scheme emerged from the data, the researchers compared their respective codebooks and arrived at a consensus to develop a final coding framework. The final coding scheme was iteratively derived as each transcript was re-read and coded by two researchers. The broad categories and themes which formed the coding framework included: most important domains related to quality of life (self-reported), financial difficulties and high out-of-pocket expenditures, incorrect or delayed diagnosis of KA, stigma associated with HIV (internalized and enacted), physical symptoms of illness, anxiety and hopelessness, expectations of better support from government, as well as inadequate care and referral networks.

## Results

A total of 29 patients were interviewed, including 16 patients co-infected with Kala Azar and HIV and 13 patients co-infected with Kala Azar, HIV, and TB. Respondents ranged in age from 23 years to 64 years (Table 1). Among 29 patients, 24 were men (83%) and 5 were women (17%), reflecting the background distribution of KA-HIV patients overall [22].

**Table 1:** Demographic characteristics of patient population.

	Patients (N = 29) N (%)
**Age group (years)**	
18–30	8 (27.6)
31–50	15 (51.7)
>50	5 (17.2)
No age reported	1 (3.4)
**Type of diagnosis**	
HIV-KA HIV-KA-TB	16 (55.2) 13 (44.8)
**Gender**	
Male	24 (82.8)
Female	5 (17.2)

We conducted a sub-analysis of KA-HIV-TB patients separately from KA-HIV patients to determine whether any unique themes emerged, however there were no meaningful differences beyond the addition of a few symptoms (dry cough, night sweats, drastic weight loss, and longer duration of sickness in a few cases). The following themes emerged from a combined inductive analysis of all patient transcripts.

### What constitutes a good quality of life?

Prior to delving deeper into the various components of quality of life, patients were first asked to identify indicators they deemed to be most important for a “good quality of life.” Invariably, the first indicators that consistently came up were income and livelihood, which were linked to others factors such as education, housing, and a clean living environment: “For a good life, the most important thing in this world is money. If you have money, you will not face any difficulties. After that, one can get their children educated, get jobs, or one can start some business…my children can earn, eat and be well” [Male, Age 37]. With income, most participants said they could improve their living conditions, including the type of food they eat and the educational opportunities for their children. The connection between good quality food and health was also not lost on participants: “if we get good quality food, then health will also be good” [Male, Age 43].

Health also featured prominently among factors contributing to a good quality of life for most patients. Firstly, patients desired a permanent cure that would “remove the disease from its roots,” and frequently felt burdened by the idea of eating medicines for a lifetime for a disease like HIV: “I would like to say that, for my family’s happiness, some kind of medicine should be developed so that the disease can be taken away by the roots. If such medicine will come, then there will be more happiness in the home” [Male, Age 28]. Secondly, patients asked for medicines which would allow them to live a quality life, in addition to living a long life, and wished the health system would cater to some of these needs: “There is frequent bleeding from the nose…[the doctor in Bhopal, Madhya Pradesh] told me there is a 2–2.5 cm mass present and the infection is dangerous and requires treatment. But he told me that…being HIV positive, I would be sent to the HIV hospital, and since I am not a resident, it would be troublesome for me. He told me that I should go home and get treatment from there… If something could be done regarding my nose and my pimples- they should get any required investigations done like the CT scan, and take me to a throat specialist. Now that I will be living more, why not improve quality of life for me?” [Male, Age 36]. And finally, patients desired access to medicines in a convenient and timely manner, and expressed dismay at the limitations on their mobility for employment. Some patients wished to obtain medicines closer to home, send a friend to obtain them, or receive financial assistance to offset the cost of their commute: “only one problem is there. I have to come every month for [HIV] medicines. They do not give it to another person [on my behalf]. How can I come every month? I think about going to another place for work. But how can I go?” [Male, Age 24].

### Patients reported high out-of-pocket expenditures when accessing treatment for acute episode of Kala Azar in the private sector

The first point of contact in the care seeking pathway for ‘new’ symptoms of Kala Azar was often informal providers such as the rural medical practitioners who set up shop in remote corners of villages which are not covered by primary health centers. No patients reported satisfactory treatment and resolution of symptoms of Kala Azar at this stage, so patients continued to switch between private medical providers. In general, patients preferred to access private providers over the free care provided by the government system, which contributed to high out-of-pocket expenditures: “I spent a lot of money…thousands of rupees…they prescribed all kinds of investigations of about Rs. 3600 each, and 75 such investigations were done. After that, they told me to do a CT scan, but even after that, my disease was not diagnosed” [Male, Age 40]. This was a recurrent theme which represented the experiences of nearly all respondents in the study. Patients borrowed from neighbors, took out loans, relied on family as a source of livelihood, and kept accessing care in the private sector until “no money was left.” In the private sector, patients were often subjected to a series of tests and lab investigations with no clear outcome: “it took one month. The private doctor could not relieve me of my symptoms. He gave me 14 injections, but it did not relieve my fever” [Male, Age 23]. Other patients were dependent on their employers for financial assistance and relied on their perception of severity of illness to seek care: “I didn’t visit anybody…Master said I should go to a normal medical shop- his medicine suits me” [Male, Age 37]. In general, almost no patients were aware of free treatment at government health facilities for Kala Azar.

When asked about general perceptions of medical care in the government system, many patients said the system suffered from long lines, poor quality of medicines, poor attendance by doctors, and poor overall care. Additionally, patients thought the disease “would get cured early in private,” and preferred to seek private care for illnesses that were perceived to be minor in nature: “I thought it’s only a fever, not a very big disease, so let’s consult in private” [Male, Age 30].

### Incorrect or delayed diagnosis of Kala Azar

Patients often spent thousands of rupees accessing “big private hospitals” where diseases were either misdiagnosed as typhoid, malaria, and seasonal illness, or simply not diagnosed at all: “we threw all our money at it, but still could not find the cause of her illness. Just said seasonal fever, tiredness, and shortage of blood…they didn’t detect that I have Kala Azar in the private setup. They detected it in the government setup” [Female, Age 48]. “I was told I was having typhoid, so I was having tension and taking injections for typhoid, malaria, and some are telling me to go here or there…I went everywhere they told me to go, but they were telling me I was having typhoid. No one was telling me Kala Azar” [Male, Age 40].

Patients also had very little awareness about what HIV was, even though many patients had heard about or had prior knowledge of KA. Many understood HIV as some deadly disease, which itself contributed to delays in care seeking, as patients did not want to disclose the diagnosis to other family members: “I knew about my HIV for one year, but I didn’t tell my son [primary caregiver] out of fear that he will get tensed with such news” [Female, Age 64]. This patient informed us that she chose to delay seeking treatment “due to financial issues” as she assumed it would cost a lot of money. This form of delayed care seeking behavior contributed to delays in diagnosing acute episodes of KA.

### Patients believed that contracting HIV has “ruined” their life

“Mentally, I was feeling that my life has been destroyed. If I die now, then it will be better. I will not bother anyone” [Male, Age 23]. There was a pervasive belief that between TB, KA, and HIV, HIV in particular had ruined patients’ lives. This manifested in different ways, including perceived and enacted stigma in the community (explored as an independent theme below) and a loss of control over other elements of personal life. For instance, some patients were concerned that they could not get married until they got treated for HIV: “HIV can spread to my partner- how can I marry then, knowing that it will spread to her too? My life is already ruined. Why ruin her life too?” [Male, Age 25]. Patients who were married often abstained from sexual intercourse out of fear of spreading HIV to the partner: “She was also tested [for HIV], but she doesn’t have the disease. We are sitting [staying] together, but not doing sex. My bed is separate.” [Male, Age 40]. Still others felt like they were never going to beget any children after being counseled by doctors: “I am not going to beget children with this disease as it is HIV…the doctor told me this” [Male, Age 23].

Similarly, some patients limited their contact with children: “with children, we don’t eat…we don’t kiss them on the hands and face” [Male, Age 39]. Much like other symptoms of anxiety and hopelessness about the future, this pointed to a loss of control over their own lives as one participant pointed out: “The effect of disease was that like I am going somewhere and someone hit me and drove away- God put these diseases on me…destroyed everything…I prayed to God to either take away my suffering or take me away from here…away from this world” [Male, Age 50].

### Perceived and enacted stigma for HIV infection

Our study found that patients experienced a high degree of stigma for HIV infection. TB and KA did not pose as much of a problem, however. “I had gone to a nearby place for shaving, but they said he has HIV and that barber removed me. I went to another place, but that first barber also informed him that I had HIV, and he also denied service to me” [Male, Age 28]. Patients did not disclose their HIV positive status easily: “In Andhra Pradesh, patients diagnosed with HIV are killed off by injections, so I thought I should keep this a secret and I didn’t tell anyone at home also” [Male, Age 36]. Patients were open about their KA diagnosis, however: “If someone asks what I have, I say Kala Azar…they know it’s Kala Azar, nothing more” [Male, Age 40]. Many had witnessed stigmatizing attitudes towards other people living with HIV in the community: “His wife was having HIV and she died. He survived, and people knew he had HIV. Sometimes, there were fights- then people used to say ‘hit him with a stick, otherwise if he bites you with his teeth, you will have HIV’” [Male, Age 28]. Patients also reported being refused care at the village level (“in my village, nobody wants to apply the injection”), being afraid of social rejection (“people look down upon those who have the disease”), and being socially isolated (“they used to come play Holi with me, but now they don’t come”).

Stigmatizing attitudes were not limited to community members. One patient mentioned that his family made him eat and sleep separately out of fear of contracting the disease: “everyone’s food was cooked together- only mine was separate” [Male, Age 35]. This often manifested in internalized stigma (“the defect we get in body cannot be removed or fixed”) as patients stated that society associates HIV disease with dirt: “the disease [HIV] is a bad one…people say that I am a dirty boy” [Male, Age 23].

The reasons behind the high level of stigma against HIV in particular stemmed from its association with spreading through sexual intercourse (“did something bad to get this disease”), lack of awareness (“people taunt me- if I spit, they say that the disease spreads by spitting”), and perception of HIV as a serious disease without a cure (“they hate HIV positive patients because it is a very serious disease”).

### Physical symptoms of Kala Azar made it difficult for patients to earn a livelihood

Patients commonly presented with typical Kala Azar symptoms, including reduced appetite (“food was no longer pleasant to me”), drastic weight loss (“my usual weight is around 70 kg- it reduced to 50 kg”), tiredness, pain (“the pain in my abdomen was always there”), persistent fever, black discoloration of the skin, generalized weakness (“I started feeling weakness”), and loss of strength during sex (“we don’t sleep together anymore, because I don’t have the strength I had earlier”). The symptoms took a toll on the patient’s body, and often translated into an inability to work and reduced income for the family: “I can’t do any work. Since the illness, my body has become weak and my body can’t work anymore” [Female, Age 45]. In other cases, patients reported feeling a lack of desire to work altogether and developed a pessimistic view of future prospects for work: “now, I have started believing that I will not be able to do any kind of work.” [Male, Age 50]

Where participants did retain the desire to work, they worried that they would have to change their line of work, as Kala Azar symptoms left them too exhausted to earn a livelihood through current means: “I can sit and work, but now I cannot do heavy work. I used to pull rickshaws. I could pull a load of 6 quintals or 7 quintals, but now my body….I can’t do it” [Male, Age 50].

### Patients commonly experienced anxiety and hopelessness about the future

Co-infection with KA-HIV had a significant impact on mental health and well-being of patients and their respective families. Almost all patients who were interviewed expressed anxiety about their current state of affairs: “I used to think- from where did this disease occur? A lot of tension came in my mind. I have 3 small children- have to get them married. How will it happen? It will not happen” [Male, Age 40]. Many patients had either been told they would not survive by other members of their community or perceived this to be true themselves: “The people from the society- those from the olden times- they said that people don’t survive this disease [HIV]” [Female, Age 45]. As a result, patients were often worried about a bleak future for their family in their absence:

“Now, if I don’t earn, then the only thing that will happen is that I will have to die hungry. Even in this period, I’ll have to do something, otherwise my four children will wander around, or die hungry. My kids’ lives will be ruined- everyone’s lives will be ruined” [Male, Age 43]. One patient went as far as to say that if he died, his “family will end,” while some others responded by saying they wished they were dead when Kala Azar symptoms were at their worst:

“I was having fever with chills…I was shivering due to chills and put a blanket over my body, but even after that, I was very uncomfortable…my weight had become 41 kg from 64 kg. You won’t believe me, but I even abused God and asked for my death” [Male, Age 40].

While HIV infection was a major source of concern for all patients, some patients also worried about the discoloration of their skin resulting from KA infection and the effects of weight loss: “My eyes had sunken. My hands became very thin and wiry. I did not like looking at myself in the mirror” [Male, Age 26].

All of this was linked to a pervasive loss of control over one’s own life. Patients frequently turned to God and felt that you can’t get anything in life “just by thinking about it” [Male, Age 60]. Kala Azar symptoms left patients wondering about lost income potential, lost educational opportunities for their children (especially in cases where adolescents had to leave studies to take care of their parents), and frequently resulted in tension, irritation, or simply self-imposed isolation from society: “[I felt like] no one should talk to me- I used to not like talking to people either” [Male, Age 43]. This was occasionally adopted as a protective behavior as a few participants reported feeling anger or irritation towards others: “I feel lonely and I feel like I should be alone…if anyone says anything to me, I feel like killing him or insulting him. I used to feel bad. I used to feel alone…each passing day, my body became thinner and thinner” [Male, Age 43].

### Expectations of better support from government

Since KA disproportionately affects the poorest segments of society, financial assistance from the Government, whether it is for medical care, housing, or food rations, was reported as a very important factor for patients. However, several patients reported that financial assistance schemes have either shut down or are at risk of shutting down: “I used to get government rations…they’ve come here now and they are saying construct a toilet, or else that will shut down too. Here I am suffering from an illness- who is going to build a toilet? The food ration is also shutting down for below poverty line people” [Male, Age 43]. Other patients claimed they have never received any assistance from the government: “we never got anything…not even on the delivery of the kids. We have 7 kids, but no money [was] received from the government” [Male, Age 45].

Caste and poverty intersect in stark ways for patients co-infected with KA-HIV to hamper access to care, which leaves them even more reliant on social security schemes from the government: “I am a poor man. I had 5 katha [approximately 0.4 acres] land- I sold it, my family sold it, but even then, the disease did not get cured. I am a Harijan [lowest status in the Hindu caste hierarchy as noted in the Indian constitution] by caste…I am a poor man. My children did not build their houses, they got busy with my problems. The government should do something to give me some benefit” [Male, Age 50].

Finally, even though patients were aware that the Government is providing money to KA patients who completed treatment ($100 USD equivalent), the vast majority said they had not received this money: “if you get treated for Kala Azar at the government hospital, then you get money. Hm? I still haven’t gotten it” [Male, Age 60]. Patients hoping for money on the basis of HIV positive status were left equally disappointed: “I was told at the department that there is so and so grant for HIV patients and I even filled the form, but no one listens” [Male, Age 36].

### Inadequate care and support networks

The multidimensional effects of poverty and illness often left patients with poor social and familial support networks. In many cases, patients had lost other family members to HIV: “My wife has the same disease, the one that is spread by sex, you know…She was very sick. I got her treated for a long time. They referred her to Muzaffarpur…by the time I got her there, she passed away. Then I was left alone with the children- they are very young. My health was failing” [Male, Age 37]. Others had children who had to leave school to earn a living, or parents who passed away, leaving limited financial or social support: “Who should I rely on for support? Mummy is not there, Papa is not there- they have all expired” [Male, Age 43].

### Referral networks are insensitive to patient needs

Patients generally accessed care in the private setup before being referred to district hospitals and, ultimately, specialized KA-HIV care and treatment services in the state capital (Patna). However, while these referral networks functioned well, they often did not prioritize patient needs. For instance, patients reported not being informed about their diagnosis prior to arriving at specialist centres:

“They told me you go to Patna for investigations and after that, we will treat you here. They did not tell me they were referring me…they sent me by fooling me. When I was referred here, I was not aware of the disease (HIV). The person in the district hospital had fooled me and sent me here. If they didn’t want to treat me, then he should have told me directly…I would have come with all preparations. He should have told me to go with all preparations like clothes for treatment” [Male, Age 24].

This often contributed to a lack of trust and transparency between doctors and patients. One patient reported that she “asked for her report, didn’t get it, and went away from the hospital” when she was told that she had to “go to Patna” [Female, Age 48]. It was not until the Mukhiya [community head] of the village got involved that the patient was convinced to seek care.

When tests were ordered, patients were not informed why these tests were being conducted and proper counseling was not conducted. This was especially true among private doctors where patients sought care: “private doctor does not tell us the truth, because he used to hide the truth from us” [Female, Age 48]. This level of distrust was common among patients. One patient reported that the doctor intentionally disclosed his HIV diagnosis to his father: “they told my father that I had contracted HIV-AIDS and that it was because I had an extramarital affair. They told him that directly…I felt really bad at that time. He could have told me this when I was alone in the room, not when my father was around…I felt that I had done something wrong. He scolded me in front of my father” [Male, Age 26].

## Discussion

Even though Kala Azar is endemic in over 98 countries, more than 90% of global cases occur in Bangladesh, Brazil, Ethiopia, India, South Sudan, and Sudan; it is associated with malnutrition, population displacement, compromised immune systems, poor housing, and lack of financial resources [27]. Our study similarly found patients co-infected with Kala Azar and HIV were highly vulnerable and prone to the compounding effects of poverty and poor health. Many patients relied on migrant labor for a livelihood, which was affected when KA and HIV were diagnosed. Migrants typically had to return to their district to obtain medicines for HIV on a monthly basis which disrupted income generation and employment.

Several studies in the literature discuss the clinical manifestations, immunopathological mechanisms, or treatment outcomes for patients co-infected with Kala Azar and HIV [26–28], yet only one study conducted by Alemayehu et al in Northwest Ethiopia examines the quality of life of these patients [29]. Our study is an important effort in systematically assessing the quality of life of patients co-infected with Kala Azar and HIV in Asia. Our results indicate that the acute illness of KA is compounded with the stigma associated with HIV, which creates a challenging clinico-social dynamic that likely results in late diagnosis, increased barriers to accessing treatment, and ultimately poorer outcomes due to late presentation.

Our findings uncovered that financial limitations have a big impact on the quality of life of patients similar to the study conducted by Alemayehu et al [29], but we found several other nuances specific to the Indian context. Patients relied heavily on financial assistance, and accessed care at the private setup first, often running into debt and exhausting all forms of social and financial support. Inadequate care and support as well as referral pathways that were not patient centered hampered access to care and contributed to distrust of health providers among patients. Furthermore, perceived stigma against PLHIV at the societal and health provider levels left patients isolated and reticent to disclose their illness to the community, and in some cases, their own families.

There was very limited awareness of treatment centers for Kala Azar among patients and a poor understanding of KA and HIV or modes of transmission. Prior to arriving in Patna, most patients had not been counseled by any provider and had not been informed about their diagnosis. Preference for seeking care at a private setup in the Indian context, regardless of socioeconomic status, is well-documented in the literature [30–38]. Our study mirrored some of these findings and discovered that patients perceived the government setup to be suffering from long lines, poor quality of medicines, poor attendance by doctors, and poor overall care. Considering the highly vulnerable status of patients co-infected with KA-HIV and examining the level of importance they afford to factors such as health, income and livelihood for living a “good quality life,” there are many potential areas of interventions to improve quality of life for these patients.

First and foremost, the government must ensure that there is no discontinuity in the level of financial assistance provided to below-poverty-line households, and that existing support is not conditioned on meeting other criteria. Additionally, funding which is routinely provided for KA patients (approximately $100 USD) is not consistently provided to co-infected patients; this should be provided regardless of HIV status and any existing funding schemes must be linked between vertically structured health programs in order to ensure continuity in care. The vast majority of patients co-infected with Kala Azar and HIV in our study reported that they were not provided the money they were promised by Government authorities, despite the fact that co-infected patients are in even more need of financial assistance than KA patients without HIV. Such patients routinely experienced repeat relapses and long term chronic diseases, and financial barriers make it even harder to access care and treatment. Ensuring that patients receive this amount to offset the costs of accessing treatment and alleviating the pressures of poverty on the family will make medical care more accessible, while early and effective diagnosis and management of these patients will serve to reduce transmission of KA, making sustainable elimination a more realistic possibility. Given that significant funding has already been set aside in the form of incentives to health workers who refer KA cases to health facilities and incentives to KA patients to compensate for lost daily wages as part of the KA elimination drive in India, the same level of commitment can and should be extended to KA-HIV co-infected patients, who are more likely to require repeated treatments.

Secondly, stakeholders working on KA elimination must focus on ensuring that referral pathways are oriented to the needs of patients. While district hospitals referred patients to Patna for treatment, many failed to explain the reasons for referral or counsel patients on what they can expect over the course of the treatment period. Furthermore, the counseling should take into account various misconceptions held by patients about the mode of HIV transmission as well as the future prognosis. Several patients felt dismayed by the fact that they could never marry, expect kids, or have sexual intercourse, which contributed to feelings of life “being destroyed.” A patient-centered approach offering health education and counseling to patients and their families can offset some of these beliefs, which will ultimately lead to a better quality of life. Similarly, HIV-related stigma must be addressed by improving knowledge of HIV transmission among healthcare providers, ensuring implementation of universal precautions across all health facilities, and improving patient education and outreach as part of counselling services. Counseling, in particular, will be crucial to alleviating feelings of hopelessness or anxiety about the future with respect to KA-HIV co-infection.

Finally, treatment protocols can take into account the myriad factors that affect quality of life. Some patients wished to receive 3 months of antiretroviral medications at one time rather than having to travel every month to the ART center, which would provide them with additional flexibility to continue migrant work. This should be considered in all national AIDS strategies, given the increased vulnerability of migrants, and resources should be extended to ensure mobile populations can access effective HIV prevention, treatment, and care and support services. Differentiated ART delivery models are available and recommended for clinically stable patients, including less frequent clinic visits and less frequent medication refills (every 3–6 months instead of every month) according to the 2016 World Health Organization (WHO) consolidated antiretroviral guidelines [39, 40]. The guidelines also recommend that ART refills be provided as close to people’s homes as possible, including the possibility of out-of-facility or group collection. Adopting such models of care can ensure patients are able to access medications without sacrificing livelihood opportunities in the process.

Despite these findings, our study was limited in that we only recruited patients with KA-HIV co-infection who presented to healthcare facilities. As a result, the results may not adequately represent members of the population who are unable to seek care, and may not be generalizable to all KA-HIV patients in Bihar. Future studies should strive to include these patients in quality of life assessments. Additionally, since this was a qualitative assessment, we were unable to report any associations with measurable health outcomes.

## Conclusion

In conclusion, patients with Kala Azar and HIV coinfection report a poor quality of life and are at risk for being lost between two vertical programs. Given the fact that Kala Azar in HIV patients is a chronic condition, the government should consider providing co-infected patients with equitable financial assistance to offset cost-related barriers to accessing treatment in order to improve adherence and facilitate recovery.

## Supporting information

S1 File English interview guide. (DOCX)

S2 File Hindi interview guide. (DOCX)

S3 File Transcripts. (ZIP)

## Abbreviations

AIDS: Acquired Immunodeficiency Syndrome
AIIMS: All India Institute of Medical Sciences
ART: Antiretroviral Therapy
CT: Computed Tomography
HIV: Human Immunodeficiency Virus
KA: Kala Azar
MDR: Multi Drug Resistant
MSF: Medecins Sans Frontieres
PKDL: Post Kala azar Dermal Leishmaniasis
PLHIV: People Living with HIV/AIDS
RMRI: Rajendra Memorial Research Institute of Medical Sciences
TB: Tuberculosis
UNAIDS: Joint United Nations Programme on HIV/AIDS
VL: Visceral Leishmaniasis
WHO: World Health Organization
