# Supplementary material for: Quality of life perceptions amongst patients co-infected with Visceral Leishmaniasis and HIV: A qualitative study from Bihar, India
Source: PLoS One. 2020 Feb 10;15(2):e0227911. doi: 10.1371/journal.pone.0227911 (PMC7010301; doi:10.1371/journal.pone.0227911)
Supplement: S1 File — (DOCX) [file pone.0227911.s001.docx]

**English Interview guide**

1. Tell me about yourself:
   1. **Prompts:** where are you from? How long have you lived there? How many people in your family? What kind of work do you do?
2. How has this past year been for you?
3. Tell me how you first found out about your HIV-VL coinfection.
   1. **Prompts:** how did you find out? How did you cope? How did you inform family members or community members, if at all? How has the diagnosis affected you, if at all?
4. What do you understand by the term ´Quality of Life´?
5. What factors do you think contribute to a good quality of life?
   1. **Prompts:** Why are these factors important? Well-being? Appreciation of life? Family life? Health? Living environment?
6. How do you feel about your quality of life?
   1. **Prompts:** How is your living situation? How has the diagnosis affected your job?
7. How do you feel about your care and treatment?
   1. **Prompts:** How are the staff? What, if anything, would you change anything about your care and treatment?
8. What are your goals and expectations from life?
   1. **Prompts:** Have these changed since the diagnosis at all? How do you feel about the future?
