# Supplementary material for: Quality of life perceptions amongst patients co-infected with Visceral Leishmaniasis and HIV: A qualitative study from Bihar, India
Source: PLoS One. 2020 Feb 10;15(2):e0227911. doi: 10.1371/journal.pone.0227911 (PMC7010301; doi:10.1371/journal.pone.0227911)
Supplement: S2 File — (DOCX) [file pone.0227911.s002.docx]

**Hindi interview guide**

1. मुझे अपने बारे में बताओ: ए। संकेत: आप कहाँ से हैं? आप कितने समय तक वहां रहे? आपके परिवार में कितने लोग है? तुम किस तरह का काम करते हो?

2. यह पिछला साल आपके लिए कैसा रहा है?

3. मुझे बताएं कि आपको पहली बार अपने एचआईवी-वीएल संयोग के बारे में कैसे पता चला। ए। संकेत: आपको कैसे पता चला? आपने कैसे सामना किया? यदि आप परिवार के सदस्यों या समुदाय के सदस्यों को सूचित करते हैं, तो कैसे करें? निदान ने आपको कैसे प्रभावित किया है, अगर बिल्कुल भी?

4. जीवन की doQuality शब्द से आप क्या समझते हैं?

5. आपको लगता है कि जीवन की अच्छी गुणवत्ता में कौन से कारक योगदान देते हैं? ए। संकेत: ये कारक महत्वपूर्ण क्यों हैं? हाल चाल? जीवन की प्रशंसा? पारिवारिक जीवन? स्वास्थ्य? पर्यावरण? 6. आप अपने जीवन की गुणवत्ता के बारे में कैसा महसूस करते हैं? ए। संकेत: आपकी जीवित स्थिति कैसी है? निदान ने आपकी नौकरी को कैसे प्रभावित किया है?

7. आप अपनी देखभाल और उपचार के बारे में कैसा महसूस करते हैं? ए। संकेत: कर्मचारी कैसे हैं? क्या, अगर कुछ भी हो, तो क्या आप अपनी देखभाल और उपचार के बारे में कुछ भी बदल सकते हैं?

8. जीवन से आपके लक्ष्य और अपेक्षाएं क्या हैं? ए। संकेत: क्या निदान के बाद से ये बदल गए हैं? आप भविष्य के बारे में कैसा महसूस करते हैं?
