# Supplementary material for: Quality of life perceptions amongst patients co-infected with Visceral Leishmaniasis and HIV: A qualitative study from Bihar, India
Source: PLoS One. 2020 Feb 10;15(2):e0227911. doi: 10.1371/journal.pone.0227911 (PMC7010301; doi:10.1371/journal.pone.0227911)
Supplement: S3 File — (ZIP) [file pone.0227911.s003.zip › Transcripts/Patient 11 Male Age 40.docx]

**Patient – 11, Age – 40, male**

I: Tell me about yourself; where are you from?

R: I came from [redacted]

I: How long have you lived there?

R: at home?

I: Yes in [redacted]

R: I live in [redacted] for last 2-3 generation

I: so u are born there. How many people in your family?

R: yes I born there. 5 people..No...6 people including mother

I: who

R: one daughter , two sons, one wife ,one myself and one mother. Father is no more.

I: what kind of work do you do?

R: I work kind of cutting *taar-khazur*, selling *taari* and some farming

I: what about your house?

R: I have kuccha house like hut

I: are you living on your own land ?

R: no, it is of my landlord

I: tell me from starting when were you completely well?

R: I was completely well 2 years back then I developed fever, difficulty in digesting food . after some medications it get relived for one month then recurs. When my disease was diagnosed then I came here from medical

I: what else happened other than digesting food?

R: I cannot digest food, even after drinking water I started vomiting and was having diarrhea.

I: any other symptoms?

R: I was having weakness and fever.

I: tell me everything

R: when I ate then loose motion occurred, pain in abdomen and became black. I spend a lot of money and then finally went to medical where investigations were done . there I was told that you can be treated in [redacted] so I was referred to [redacted].

I: where did you first went to seek treatment?

R:first I went to my village for treatment.

I: to whom? Doctors or ….medical store

R: Doctor ….to good doctors

I:is it a govt hospital ?

R: Private

I: what did they told?

R: they told that my liver is not working and lastly my disease was diagnosed in Medical.

I: Where?

R:Medical, [redacted]

I: Medical ? what it is?

R: hospital….it is government hospital

I: after village you directly went there

R: yes

I: what happened there?

R: some investigation were done

I: medication were given or not?

R: no, only some box was given

I: what was inside it?

R: tablet was there to eat during night

I: how many days it’s been continuing?

R: from 8 months

I:at [redacted] only?

R: yes

I: then how did you came to [redacted]?

R:medication from that box is continuing but the rest treatment is continuing from [redacted]

I:what happened when you came to [redacted]?

R: Some investigation were done and then medication and injectables were given. I was discharged after 40 days

I2: which disease you came to knew first? Kala-azar or HIV?

R: I knew about it simultaneously

I: Where?

R: [redacted]

I: you have told that you spent a lot of money

R: yes, about 5 lakh

I2: where and in which thing?

R: I went from [redacted] to [redacted] for treatment

I2: tell me more ? where did you went?

R: I went to [redacted]

I2: whom did you went?

R: doctors

I2: was it a medical store or big hospital?

R: big hospital … and good doctors were sitting there

I: was it private?

R: yes , it was private

I: how much they demand?

R: they wrote every single investigation of about Rs.3600…every single investigation…. And 75 such investigation were done…many investigations. After that they told to do CT scan but even after that my disease was not diagnosed.

I2: how do they behave with you?

R: they told me that my liver is damaged .

I2: anything else they told you?

R: no, nothing else after that lastly I cannot speak …at last… so I went to Medical.

I2: in which hospital?

R: medical …Government hospital

I2: Where?

R: in [redacted]

I2: what did you done there?

R: when I went there I have done registration ..After registration I went to doctors. They had written some investigations, Ultrasonography, many investigation were written . after all the investigations were done , disease was diagnosed and madam told me that you cannot be treated here but you can be treated in [redacted].

I2: what was told about your disease?

R: you have both this disease …Kala-azar and that

I2: Kala-azar and?

R: this…HIV

I2: Did they told something about this disease? What is this disease? How it spreads?

R: yes they told me…..

I2: at government hospital

R: No, at [redacted]

I2: at that government hospital what was told? Only name was told?

R: only name was told

I2: Anything else

R: no nothing else

I: you already spend a lot of money…can u tell me why did you visited Private hospital first instead of govt.?

R: I was not having that much knowledge, I am not literate

I2: did you know earlier that free treatment is given at government hospital?

R: yes, I know that but still didn’t went there

I2: why so? Can you tell me what is the reason?

R: there was minor fever or something so I think I may have some problem so went there . I ran for 1 year but still didn’t get any relief . when my condition of my house become so bad then people told me that it is last position so go to medical. So I went to medical.

I: when you came to [redacted] then what did the doctor told you about your disease?

R: they told me about this disease.

I : they may told you what this disease is and how it occur?

R: he told me that was you living outside, I told that I didn’t went outside. He ask that then how it occurred to you. This disease occur due to many reason that what they told.

I: anything else ?

R: anything else…. The doctor told me that you don’t have to go anywhere from here for 40 days then you can be treated.

I2: where? In [redacted]?

R: yes, [redacted]

I: Did they told something about kala-azar?

R: yes, it occurs due to mosquito bite

I2: from mosquito bite? Where it was told?

R: yes, in [redacted]

R: there they teach that there are various mosquitoes, sand fly and many more. The doctor told that due to HIV many disease can occur. That is what they make me understand.

I2: can I ask one question… how do HIV spreads ? you already said about kala-azar but how HIV spreads?

R:It spreads through blood.

I2: by blood media. What are the reasons to spread ? did they told you?

R: if your blood get touched to other people then it will spread to other people so you have to take preventions.

I2: when you knew about HIV then how did you felt? You knew about both disease simultaneously then how did you felt?

R: at that time I felt bad.

I2: tell me a bit more

R: very bad…I got tension

I2: which type of tension?

R: I got disease so what should I do.

I2: did you knew that this disease can be treated or not ? at that time?

R: I didn’t knew that much

I2: what are the things that you hope to do but the disease affected it?

R: it may happen due to contact by blood.

I2: ok..what are the thing you expected to do but you cannot be able to do due to disease?

R: I don’t have that much knowledge

I2: it’s not like that…its like what you feel that is what the question is

I: he want to ask that you have disease then you feel bad and not able to do something?

R: yes I feel like that and I feel ashamed also

I2: ashamed ! why?

R: I feel bad that how do this disease occurred to me.

I: what do you feel ? explain it

R: I have this disease so I feel bad

I: why do you feel so bad?

R: due to this disease

I2: what is different in this disease? Every year you are getting fever, cold or cough but what is different in this disease that you feel bad about it…You feel ashamed ?

R:this disease happened to me … I might have done something wrong .

I2: it occur due to mistake ?

R: blood was also transfuse 3 year back, I got an accident. My younger son’s jaw got completely damaged.

I2: the one who come along with you , do she undergone test for that

R: yes , my wife has disease.

I2: both…only HIV or Kala-azar also

R: HIV only, she don’t have kala-azar

I: do she know about the disease

R: yes

I2: do she knows that how this disease spread?

R: yes

I2: how are your children?

R: children..children..disease…children are well

I: how did you told this to your family or neighbor?

R: I don’t tell them. Only my wife knows that and my family members.

I2: ok family members know that…what did you told them?

R: that this disease occurred to me

I2; did you told the name of disease

R: yes I told to my mother. Mother is the only old member in my family, my children are younger

I2: do they know what the disease is?

R: yes, they know that

I2: they have heard the name

R: yes they have heard the name.

I: you told them also

R: yes, I don’t tell this to my neighbor. If someone ask then I tell that I am having Kala-azar.

I2: you tells about Kala-azar but do you tell about HIV?

R: No one asks, if someone asks what I have, I say Kala Azar.

I2: if someone asks then can you tell?

R: how you say I’ll do that.

I: do you have any problem to tell about your HIV status to someone?

R: Yes, I can tell.

I2: what do you feel when you tell that?

R: I do feel something. I feel like I am having bad disease.

I: what is bad in this?

R: people have sense of hatred for this disease.

I - You have felt that people taboo? How, like tell one example.

R - I hear, people taboo this disease

I - What something, sometimes in village, sometimes something happened.

R - Not like this happened but one happened/

I - What?

R - It is a story of 5-7 years back. He got this disease then used to hear about him many people of the village said that could not get touched to his body this is a disease.

I - What else said the people? About him.

R - Not anything else. Only this thing was said.

I - Good, this about which you are speaking about that disease, you are speaking about HIV or Kala-Azar, speaking about which disease?

R - Only these two diseases were.

I - Bothe the diseases, he had?

R - Yes

I - This you know, surely.

R - Yes

I - Then people meet him? Or did not meet?

R - Used to meet him

I - Means, after this disease

R - Yes, everyone, used to meet

I - Good, but was not like before.

R - Yes, was not like before.

I - You feared this only?

R - Yes

I - Good, when you knew about this disease, then what did you feel that in future you could do something that you are not able to do because of this disease?

R - Yes because of disease, I used to think that to teach children, make house, after earning that now I think no.

I - Now that your, It is from 3 years no?

R - Nine months have happened, eating this drug

I - Okay, so what-what happened during these 9 months? Means apart from disease, how were you feeling mentally?

R - Something is being felt.

I - What did you feel.

R - Strength has increased. Little

I - This disease has been treated, then after that strength has increased?

R - Yes

I - Now, when disuse started, in that time, what did you think?

R - I used to think a lot in the mind.

I - Yes then tell what all you used to think?

R - When disease was known then?

I - Yes

R - Used to think, form where this disease occurred, how it occurred, a lot of tension came in mind. I have 3 children, small, small have to do marriage. How will it happen will not happen.

I - From this what impact happe4ned after this disease on job-earning?

R - Cannot get strength, Sir

I - Can work as much as before now?

R - No, No

I - What, What are you feeling, why care you not able to work?

R - Cannot get strength

I - Cannot get strength

R - Now, then can you do farming work?

R - No, cannot do

I - Then how you run your house?

R - Run the house form loan-borrowing.

I - Now then you are not doing any work?

R - Not doing any work now

I - Tell this thing what all is needed for good life, what do you think? That my life, becomes very god. I can live properly.

R - Means treatment happens properly.

I - Means what all is needed?

- Like you are saying that treatment happens properly? So how you treatment happened? Tell me about treatment.

R - Treatment was done properly.

I - Was done properly?

R - Yes

I - Is there something which did not happen?

R - No, no problem happens in treatment.

I - Can you tell that what all you see in treatment? What things should happen in treatment? What thing matter?

R - Treatment, sir is going right, now job-work, clothes-money, problem remains, children eat one time only.

I - Before the disease, conditions were like this or became worse after the disease?

R - Became very bad after the disease. When disease was not there then fooding went properly. Properly all the family ate. Now income is not there so problem occurs.

I - What kind of problem in happening, what all is happening?

R - Of eating-drinking, brought bricks for making house, sold all.

I - So could not make house?

R - No

I - How do you feel in eating-drinking?

R - Right in eating-drinking.

I - This effect which has come of disease, form this any change in your family member in talking after disease.

R - No

I - Before disease

R - No

I - Means any change in your wife’s behaviour or of neighbours or of child.

R - No

I - Not anything?

R - No

I - Neighbours are like before?

R - Yes

I - If you are said that what all you need for good life then what will you want for your life? That you can life happily.

R - Could not understand sir

I - Am saying that you have to be happy in your life now. What all you want to do afterwards means what all should happen in your life that you could be happy everything could go properly?

R - Children remain happy from house

I - How? I am asking that only? For this happiness, what all is needed?

R - No, house, money

I - Something else

R - What other thing land etc.

- If land is not there then cannot take house.

I - Whose land is this?

R - Master’s

I - His land is then? Did not understand? What did you tell?

R - It is Master’s land. Two gave rations have passed. There was no land told him that he give little land to make house?

I - Then what do you want to do to make your family life god? Your family remains properly, remains happily.

R - That all, house, eating-drinking

I - What do you want to do for that?

R - I can do work and what else will do?

I - If disease was not there. What else could you do?

R - Doing work, saving some money, would get children educated, make house, if some money would be there then future would get forward. Now nothing is happening. It is a lot trouble.

I - If you did not have disease then what else would you want to do?

R - Used to think that will do any big business investing my money. When money will be there. These all I thought.

I - Then, it occurs then you want to work now but don’t feel like due to disease.

R - Yes because of disease, don’t get strength.

I - What would talked about money, the effect which happens on money then when you go to get treatment. They you get any money?

R - Get Rs. 600/-

I - And treatment is free?

R - Yes

I - And what else would be needed during treatment from what things you get ease?

R - Ease –a-a-get up-down, get Rs.600/- called from their first reserving vehicle, got Rs. 600/-

I - What all happened in last 8 months. Anything which you want to tell.

R - Livelihood is running very probably in last 8 months from when fell feel keep children sometimes in this relation sometimes in that relation when no option remains at home.

I - Someone in your neighbourhood has this disease?

R - No

I - Kala-Azar

R - No

I - No-one has Kala-Azar?

R - Yes happened 10 years ago. He had only Kala-Azar.

I - Now, the treatment you are undergoing in [redacted], What do you think about that?

R - Treatment undergoes properly.

I - Means how it goes properly. What all you felt good.

R - I like everything there.

I - What all happens with you? When you go to [redacted] what all happens in whole day. How is the doctor?

R - Everything fine, ge3t food, doctor is fine, check-up-investigation everything happens.

I - Any problem also happens?

R - No problem occurs

I - Any money is also spent there?

R - No, no money is spent, if nay penny is spent on medicine from outside then I get the money.

I - The staff which work there, how is their behaviour?

R - Fine

I - Everything is fine?

R - Yes

I - IN the treatment, you feel anything is bad?

R - No, nothing is bad. Everything is fine.

I - Then would you like to change anything there so that your treatment occurs better.

R - Treatment is right.

I - Anything like this?

R - No problem occurs in treatment

I - How many days you have stayed in [redacted].

R - There when I came for first time then remained for 1 month and 8 days (40 days)

I - In that 40 days, something bad happened with you?

R - No

I - Everything good only happened?

R - Yes everything good only happened. Good treatment is done.

I - What all happened. What all you did there in whole day? Tell in detail.

R - IN hospital

I - Yes

R - There only check-up, investigation occurred. BP check, weight, snacks, food everything doctor sahib saw.

I - And now that you remain at home what all you do?

R - Do not do anything?

I - All day?

R - Yes remain at home.

I - In the house remain as sleep or in neighbourhood?

R - Yes keep, roaming

I - Where do you roam?

R - Market?

I - With whom?

R - Alone

I - Roam alone?

R - Yes

I - Previously also roamed alone?

R - Previously used too rom with 2-3 people. Now roam alone. Roam very less.

I - Now Why you go alone? Is there any reason?

R - I go alone now. There is no reason. When body become week nobody cares.

I - Means people don’t talk to you much?

R - No

I - Don’t want to go with you?

R - No

I - And what all you want to do in your life? Ahead

R - What will like to do in life?

I - Now you are feeling little better than before no?

R - Yes, feeling better than before

I - Now, you can work nor no?

R - Yes can work lightly

I - Do you do?

R - I would do light work.

I - What all things

R - Will sell vegetables then what would be able to do thatched.

I - Now what all you could do and what not? Now

R - Two months back when I went back after taking treatment for here then earned something, requesting my master.

I - What work you did?

R - That only thatched?

I - Now when your treatment is going on?

R - No, not from one month.

I - If you will get well then what else will you like to do?

R - What else, will do this work only.

I - Will do this work only?

R - Yes, am not literate, that will do another thing.

I - What do you want to do for your family?

R - For family, that only there are 2 children had thought that will open some general store, will be employed.

I - And about their studies?

R - Studies, send in government (School)

I - Send in government?

R - Don’t have money for eating so send in government.

I - They go in government, in which class they study?

R - One in 3^rd^, one in 5^th^

I - After disease, is there any effect on his studies

R - Yes used to get tuition also before. Now there is no money.

I - So, you have stopped the tuition

R - Yes

I - But goes to school now?

R - Yes

I - Now, you have come [redacted] so for what you came now?

R - For check-up

I - What check-up?

R - These thing happened rashes.

I - From what time did this occur?

R - From 2 months back it occurred.

I - What type of investigations occurred now?

R - Investigation has occurred, now medication is undergoing.

I - In investigation what blood was taken?

R - Skin was cut

I - For that only you have cure?

R - Yes

I - Then what was told about the rash?

R - Told, is an effect of Kala-Azar

I - From what time is it?

R - This investigation occurred 2 months back

I - After this what effect occurred on your life?

R - Body does not look good on seeing.

I - The people in neighbourhood, when the see then they give different type of reaction that this is spread?

R - Yes there many like this. Educated people talk but illiterate people don’t talk. Educated people say that th4e disease will not spread by talking.

I - The people who are illiterate, what do they think?

R - They feel bad. They say he has disease, if he will remain in front, then will spread.

I - Then what you say to him?

R - I say –say what you say. Educated people don’t think anything.

I - Then do you reply them also?

R - Did not give

I - And friends etc, say anything after seeing this? With whom you remained before.

R - No

I - Do they remain now also?

R - Stay sometimes

I - Sometimes, not like before?

R - Yes

I - Sometimes while talking, they say something about this?

R - No

I - Due to disease what effect has occurred on you mentally?

R - What should I do? 7 5 types of headache remain in my mind.

I - What did you think? Tell me completely.

R - Used to think, to get forward by earning something, do for the children what else. This all I used to thin. Now there is no solution.

I - What is more important for you, good mind or body?

R - Both, there should be no problem from body and children also remain happy.

I - What do you think. Why did it happen so late coming here? Means in RMRI.

R - Could not know about disease. After knowing came the next day after that.

I - Now, the money spent in treatment, how did you give that all?

R - Where?

I - The treatment in private?

R - There we household items, had bought bricks for making house by selling all this. These was, money, jewellery for daughter. Sold everything. Ended everything then after donation got treatment.

I - You had to ask for donation?

R - Yes village people … 100-50 with that food for children and treatment occurred.

I - How is it now?

R - It is fine now, now I could earn Rs. 10/-

I - Now, what all problem do you have? Eating-drinking

R - There is all problem in eating-drinking

I - What other problem are there?

R - There is nothing in the house, nothing

I - Then what all should be right?

R - Eating, drinking, house

I - How much do you eat now in the day? 2 times – 3 times?

R - Eat 3 times

I - What all do you eat?

R - In the morning, 12’O Clock, and in night

I - What all do you eat?

R - In the night I eat milk.

I - What did you eat before, what do you eat now?

R - Previously used to eat anything, vegetables, meat, fish means something today, something tomorrow now whatever is cooked, I eat it.

I - During this, how did your relation with your wife remain? Did you see any difference?

R - No, remains lovingly.

I - What does she say, about this?

R - What does she say? It was to happen, so it happened.

I - Any problem from this rash?

R - There is no problem sir

I - She has any problem?

R - She also feels weakness during walking.

I - No, about this rash, she said anything?

R - No says, let’s go to show it.

I - Anything else she does not say?

R - No

I - Supports you every time?

R - Yes accompanies every time. Doctor sahab calls after 10 days or 8 days if have to stay for 4 days then she stays.

I - When know about disease for first time. Then some interrogation was done with you? That how happened, Why happened?

R - Wife?

I - Yes

R - Had asked this disease came from where, blood transfusion. Three years ago fell and got treatment in [redacted]. Had stayed for 3 months. Child is young his jaw got completely destroyed.

I - This disease HIV. How does it spread? One you told from blood and what all?

R - Doing mistakes

I - What mistakes?

R - Like I have HIV will spread by touching other. Doctor sahab suggested to keep away from these things.

I - Then did you do anytime with someone?

R - No

I - Like you stayed away?

R - Used to study at home

I - So after that have you taken precaution for that?

R - Yes, all precaution, I think I have got, other should not get sir

I - For this what all precautions have you taken?

R - Remain safely

I - When you have intercourse with your life. Then you take any precaution? Means anything like this you use?

R - Yes, use condoms

I - Anything else?

R - No

I - What are advantages of condom?

R - Disease is not spread to others like AIDS, Kala-Azar.

I - What happens in this disease?

R - Bad thing occurs. Children should remain fine

- Doctor Shahab told for check-up children?

I - Then did you get it done?

R - No, I don’t get time

I - When did doctor sahab tell you?

R - Two months back

I - You did not get it done till now? What did doctor Shahab tell about this disease?

R - I don’t know anything

I - You go your check-up done. Then anything came out in it?

R - Yes, a little disease

I - Did tell any name of disease?

R - No sir.
