# Supplementary material for: Quality of life perceptions amongst patients co-infected with Visceral Leishmaniasis and HIV: A qualitative study from Bihar, India
Source: PLoS One. 2020 Feb 10;15(2):e0227911. doi: 10.1371/journal.pone.0227911 (PMC7010301; doi:10.1371/journal.pone.0227911)
Supplement: S3 File — (ZIP) [file pone.0227911.s003.zip › Transcripts/Patient 12 Male Age 30.docx]

**Patient 12 age 30, male**

I- Did you have difficulty on the way? Was there much of traffic jam?

R- Yes.

I- When did you leave your house?

R- I left my house…. (Pause…trying to recall)

1. Hmmm….

When did you leave? Where is your house?

R- My house is in [redacted]. I left my house yesterday night at 9PM.

I you left yesterday night itself?

R yes.

I How did you come…? By Bus?

R yes…….. By Bus.

I Till where did you come by Bus?

R By Bus, I came to [redacted] then I came to [redacted].

I Ohh…! How much time does it take to come?

R Time taken to come from there is 6-7 hours.

I Did you have jam there too?

R No. There was no jam at night. Today morning it was there. Once we came then returned back. Then came again to reach here. Again the same jam was there.

I You had to return back once.

R Yes

R And your house is there itself in [redacted].

R 3Km ahead of [redacted].

I ohhh…!! Have you been coming there from the beginning?

R yes…. I live there.

I You were born in [redacted] itself?

R Yes… 3 Km ahead of [redacted].

I Who all are there in your family?

R In my house Mummy, Papa I My four Brothers younger to me, my four children and my wife.

I All your four children are young?

R yes.

I All four live with you?

R yes. All four live with us.

I How many sons & daughters?

R One boy and rest girls.

I Boy is the eldest?

R No… 1 daughter is eldest then son and then 3 daughter.

I So… you have five children?

R yes. I have 5 children.

I So, all 5 live with you?

R yes all 5 live with me.

I Do all 5 of them study?

R yes all 5 study.

No not 5….. 2 are very young at present. 3 of them study.

I in Govt. School?

R in government and in 1 in private. The elder son

I what work do you do?

R work of mistri

I the electricity wires

(Interrupting)

R Plaster white wash and all

I ok. So you have the work in house construction

R yes

I U have been doing this work from the beginning or did u do other work initially?

R no initially I did that and when I learnt this then I started doing this.

I what did you do initially?

R initially I worked as a labor.

I the work of loading

(Interrupting)

R giving bricks…. Cement then when I learnt I started doing this.

I from when are you doing this?

R This work I have been doing from 15-20 years.

I from 15-20 years???

R yes 20 years.

I how was your last year?

R Last year was very bad.

I Why was it bad? What happened?

R Like my health

I Hmm

R Then I came here. Treatment started

I From when r u having this disease? When did u come to knew?

R (interrupting) I came to know this on 20^th^ of this month.

I In November?

R In 2017

I 2017 November?

R Yes in November, then my kalazaar was diagnosed

I (interrupting)… from when did it all start? From November?

R This started 1-2 month earlier than that. I was outside then I came home. I came to [redacted] district. There I got treated in govt. set up. Got tested then they said me to go to [redacted] hospital when I went there [redacted] sent me to [redacted]. I came here. And my treatment started.

I you said u came from somewhere to [redacted] were u living outside that time?

R yes

I where used to go outside to do job?

R in [redacted]

I So you work in [redacted]?

R yes. In [redacted]

I from there when do u come?

R 2.5-3 months then return home then 2.5 months at home. Or 4 months at home.

I before [redacted] did you live at some other place?

R No before Himachal few years back 8 years back lived at [redacted] district. [redacted]

I u lived there worked there?

R yes there I worked there itself I learned then I went there

I [redacted]?

R yes. Has been working for 8-9 years there.

I Your illness started from [redacted]?

R Yes… Yes from [redacted].

I hmmm… then you came to [redacted]?

R Yes.

I Where did you get medical consultation in [redacted]?

R [redacted] in govt. hospital.

I There you were told about [redacted] (interrupted)

R There they got me tested and sent me to [redacted] from there they sent me here in [redacted] here my treatment started.

I what treatment did you receive in [redacted]?

R That. (Pause) what you say…. HIV’s treatment didn’t start like (interrupted)

I Where did you come know about HIV?

R I came to know about HIV at [redacted].

I First time you came to know here?

R yes here only I came to know

I and kalazaar? What was diagnosed first kalazaar or HIV?

R Here only I came to know from here itself the treatment started

I when you first went to [redacted], what were u told there?

R They directly referred here.

I But was any tests done there?

R yes. Tests were done. (Interrupted)

I They didn’t tell you anything?

R Blood test was done and told me that go to [redacted] on 2^nd^. You will have to stay there for few days and I came here.

I weren’t you told anything else in [redacted]?

R No… Nothing else was told to me in [redacted].

I They only referred to [redacted]?

R Yes, they only referred me to [redacted]. In [redacted], they gave me papers which I took and came here.

I Ok. So in [redacted], Blood test was done but nothing was told?

R No…. I wasn’t told anything.

I Apart from bold, any other test? From bone?

R Nothing. Nothing.

I ok so after being referred you came to know in [redacted].

R yes. In [redacted] I came to know.

I Ok… So did you know both things simultaneously? Kalazaar and (interrupted)

R yes. Both together. First Kalazaar then HIV

I Did you get fever?

R yes I got fever from beginning.

I Tis too from two months prior to November?

R Yes Two months prior.

I From around September?

R Yes Yes….

I How did you feel when you first came to know about your disease?

R Like…. I had weakness…. Weight started decreasing I was 64Kg in weight… we3ight kept decreasing did not understand… did not get treated anywhere. Just kept working …… asked somebody and took medicines from any doctor when fever accrued. Remained all right for 1-2 months… kept working ….. I became very lean and thin …….. Then I got tested in [redacted]… From [redacted] then sent to [redacted]… From [redacted] they sent me her

I in [redacted].

R Yes, In [redacted]. Here tests were done, medicines started now I am fine.

I Now you feel fine?

R Yes, Now I feel fine.

I In [redacted], when you first came to know about your disease, how was your mental situation then? How did you feel? Or did you feel that this disease is bad. What did you think about the disease?

R Yes…. Yes…. The disease I thought was bad.

I Did you know about this disease earlier?

R No, I didn’t know about the disease. When thy told then I came to know that this disease is bad.

I You didn’t know… Not even about Kalazaar?

R Not even about kalazaar. However, I had heard that Kalazaar occurs. But I was scared of HIV.

I You got scared of HIV?

R Yes…. Yes…

I What did you think that time?

R Like……. People say……. Call HIV as AIDS….. I thought I will die.

I Ohhh…!! So you thought that people die of HIV?

R Yes.

I Apart from this, what did you think? How could your life’s situation be affected by your disease?

R I thought many things.

I What all?

R Many things….. Thought about many things…. Many things were missing in life….

I What?

R Many things were missing from the time the treatment started it is fine.

I What things were missing?

R Like… It was difficult to live together with your wife.

I Were you told not to live together with your wife?

R No… before the treatment.

I Why there was problem? Before treatment you didn’t even know about the disease.

R Yes, I didn’t know but like the strength I had earlier, I didn’t have then.

I Ohh…!! That’s why you had difficulty.

R Yes, that’s why I had difficulty.

I From the time disease started you had difficulty?

R Yes, from that time.

I And in family, did you get your wife tested?

R Yes, I got my wife tested. She also has.

I Your wife also has…… Is she getting treated?

R Yes… It is going on …. Medicines are going on.

I When you came to know about the disease, was she with you?

R No… When I returned, they told to get wife and children tested… then me and my wife was sent… when they tested children they didn’t have…. Wife was not told that I was sent here…. Then, Jitendra Ji who lives there passes which month is this?

I April is going on.

R April is going on ……… From April 6, her medicines have started.

I Your wife’s?

R Yes…. My Wife’s

I You didn’t tell your wife that time?

R No…. I told……I told.

I And other member in family? Did you tell them??

R Rest all are fine.

I Do they know… People who live with you Maa Papa…

R Yes… Mother Father live with me.

I But do they know about the disease?

R Yes…. They Know.

I They came with you for your treatment?

R Yes. They came… Father came

I Ok…. So when you returned home, you got your wife treated?

R Yes.

I From where is her treatment going on?

R There only…. [redacted]… (Correcting) from [redacted] her treatment is going on.

I Ok… If you sit and think about a good life, what do you think? What all should be there for a good life? Like money, house, few people think……. What you think?

R I also think the same.

I What all?

R That my life remains good….. Earn money.... educate my children…live a good life.

I Anything apart from this? In a good life (Interrupted)

R In a good life, things remain fine

I What do you want to say saying fine… (Paused)……like family environment.

R Yes...Yes….. Like may my health stay fine… As I am now, I stay that way. May I not face any difficulty…? I can work…. Earn some money… educate my children.

I Has this disease affected all that what you thought earlier?

R No, nothing like this. Earlier, I did not know from the time my treatment has started, I am fine….. I feel that I am fine… nothing like that… I can work… feed my children… I can do something.

I Ok….. So you think you will be able to accomplish all that you thought earlier?

R Yes…….Yes.

I Has this disease affected your job?

R Now, it is becoming all right. Earlier, I did not feel like working… had difficulty lifting weight. Had problem in doing hard labor. Now it’s nothing like this. Now I think that there is no problem even if I earn the whole day.

I Ok….. So now you feel there is improvement?

R Yes…..! I feel there is improvement. But I will have to take medicines.

I You feel better compared to before, right?

R Yes… I am better than before.

I For how many days was your job affected?

R I have been working again from 1 month. There was a gap of 3-4 months. There was no work.

I How were the expenses managed then?

R There was problem. We are in debt. What to do?

I You are the only earning hand in family?

R Yes… Yes …

I There is no help from anyone else…

R No father is there. He does agricultural works. We don’t have much land of our own.

I Ok so you get help to some extent.

R I get some help, but I have four younger brothers have to look after them also. I am separate

I You look after your younger brothers?

R No… No… Mother father does I live separately my expenses are separate.

I Don’t they earn at present?

R No… two brothers are studying, one (12+) final, and other intermediate final.

I Ok… So do you feel satisfied by your treatment that is going on?

R Yes… Yes… It is very good.

I The people who give you medicines.

R Yes… They do very good.

I What are the good things there? What are the things that can be improved? (Both silent) about the treatment?

R Treatment (mumbles)

I Do you want any improvement? Or are you satisfied? What do you feel?

R I am fine.

I The doctors who come to see you there. How is their behavior with you?

R Very good… Very nice… very good they are.

I When you went to government set up. How was it there?

R There too it was fine.

I Was there any difference?

R Initially I went there. They did some tests and sent me here rest (trailing)

I Do you appreciate any difference in treatment amongst the two places?

R Yes… Yes… have the treatment is good. In Patna it is good

I Better from there?

R Yes… Yes…

I In what respect do you say it is better? I mean, why do you say its better have?

R Like… there it’s not that comforting not very clean.

I hmmm…. And?

R Here every comfort is present… of fooding lodging in every respect, it is good medicine is also fine…

I So after how many days do they call you have?

R Initially they called after a month… then after 3 months…

I And now… after 3 months?

R Yes…

I So do you want any improvement the way you are being treated? the medicines that you are given the schedule on which you are called and the way they behave with you … if you think that if it was there, it would have been better… do you think so ?

R. Yes… Yes…

I Would you like to have any improvement?

R. (long pause) I am not understanding.

I Do you want so? That this thing is bad. It should be corrected anything like this where you are treated? (Pause) Is there anything which if present would be better? Treatment think a bit and say.

R. In treatment, I would say a bit about money which is there (trailing).

I What about money?

R. Like (Interrupted).

I Yes…. What?

R. Like… I have to spend 1200/- 1300/- Rs. For fare.

I Fare for your coming?

R. Hmmm…… Here I get 1000/- rupees for two people

I You get 1000/- Rs…

R. Yes…

I What improvement do you want? You get the money right?

R. Yes I get.

I What improvement do you want?

R. I have to spend 200 – 400/- Rs. From my pocket… nothing else the medicine I get from here is fine.

I What you get free of cost?

R. Yes… Yes…

I don’t have to spend.

I So what do you want? You have to spend 200 – 300/- Rs from your pocket.

R. Yes… Yes…

I How much do you earn from the work you do?

R. If I work for thirty days I get 12000/- Rs.

I According to daily work?

R. Yes… Yes…

If I work daily, if I will be absent … I will be less.

I Do you plan to do something else in life? Your expectation from life (pause) do you think so, that you would like to do? (Long pause) Have you thought something in future? Will buy house…

R. Yes… Yes… I will buy house.

I The house you live in is yours?

R. Yes… Yes… I am thinking about building a house.

Yes It is mine. Have whitewashed it have not got the ceiling cemented man think many thing but all of that doesn’t happen… when it’s not there what to think about it.

I So what do you think?

R. I think if I had money … I would build a good house.

I At present, how much land do you have?

R. My land (pause) six room build in it one room for every brother. One lives in each room, using tins.

I Brothers live with you?

R. We all live together…

I Kitchen is separate?

R. My kitchen is separate there.

Kitchen is separate as bests is there, ceiling is not cemented if I was good could have done something good… even money… I could also get money and use it to make house… that’s it….

I Even after the disease, do you think, you will be able to accomplish what you thought earlier about building a house?

R. (Interrupting) Yes… Yes… now I will build… I feel so… now I am fine, I will build.

I You feel fine now… You feel strong from within?

R. Yes… Yes… I feel fine from within I can build.

I During the illness, did you fill that your dreams will be fulfilled?

R. During the illness, I did not fill this …during illness, I felt that I will now be separated from my child … I felt that I am now going to live for a few days only I felt this.

I Now it’s fine…?

R. Its fine from how many days…

I Are you taking the medicine?

R. My medicine from 11^th^ to 20^th^ month.

I From November?

R. Yes from November now its fine, no problem.

I Would you like to say something else about yourself?

R. It’s ok only this much.

I You told you get money … 1000/- Rs. You get, do you get this in government setup as well? When you go there?

R. Here…?

I Not here… Not in Patna …. Uh…. Uh….the place where you went.

R. There I don’t get. There I have to spend for the fare for coming & going.

I OK….

R. Take medicine & go …

I You are getting your wife treated from there?

R. Yes…

I Why not from here? Is there any problem?

R. No… Here it takes move fare to come here I like get medicine for HIV monthly from there … from there mouthier is 50 K.M. from my house in 200/- Rs. I am back home… 100/- Rs in coming & 100/-Rs. In returning coming here will take 500/-RS.,600/-Rs. That is nearer so got transferred from here to there.

I You got treatment from there?

R. Yes… Yes... from there only… medicine for Kalaazar is take from here.

I OK…

When you got sick initially, how much time elapsed before you reached the hospital? Or consult somebody? Where did you go first? At the very first.

R. At first, I consulted in [redacted].

I Whom..?

R. Myself...

I NO… to whom? Private practitioners as RMPs?

R. Government Hospital.

I You went there first?

R. Yes… Yes…

I consulted many private practitioners earlier the doctors are there, right? I consulted there like many stomach I had frequent stools most important, I had loss of appetite frequent stools I ate something 3 to 4 times in a day & 3 to 4 time in night. They said this is there, that is there, so the food is not digested… I consulted private doctors.

I You consulted there only in [redacted]?

R. NO… No… in my own place in [redacted].

I Means… two months later when you came to [redacted] then You (interrupted)

R. Before that, 2 – 3 months before I consulted.

I 2 – 3 months before (interrupted)

R. 2 – 3 months before I consulted & went there…

I Returned back…

R. To [redacted]. To work… mean I was better.

I When did your disease start then? Like you told in September.

R. Yes... when I went there to [redacted]… more and more weakness I felt… I worked for 2 -3 months… I had profound weakness… I had breathlessness… did not feel like to work. Then I come and got treated & started medications.

I Then you again consulted government hospital in [redacted]. ?

R. Yes… Yes… (Pause) there… I consulted … from there, they sent me to [redacted].

I Yes…

R. And have from [redacted].

In [redacted] like the private doctors are there right. ?

I Yes… Yes…

Was it a small rented shop or a big hospital?

R. No… just a small one but he was also MBBS. Nothing like that… His fees was also 300/-to 250/- Rupees. I gave and got checked fever as I already told, somebody said its typhoid, and someone said something else.

I You had fever & loose stools.

R. Yes… Yes…

I This seems to have started before September.

R. Yes… Yes… from before.

I How many days before September?

R. 6 – 7 months before September.

I Ok... so 6 – 7 months before September your problems started in [redacted]… then you came to [redacted]… you got treated & went back.

R. Yes… came to my house then my medicines are going on …

I The private doctors you consulted was it a single one or multiple?

R. There… I consulted three doctors… somebody said here it’s not good it’s good there. I consulted three doctors.

I What were the tests done there?

R. This only….. of fever… (Pause)

I Which tests? Blood test urine test?

R. Yes… blood test & urine test was done …. X–Ray was done

I Nothing would be known from those? Nothing was told to you?

R. Nothing was told to me .one doctor said its typhoid. One said there is that of livers. So…. Loose stools that is there gave medicines. He did not do x-ray and all.

I So.... You were taking all those medicines. No improvement…. was there any improvement?

R. No…

I Then you returned back to Himachal.

R. Yes…

I Your health was not fine that time? You returned to work?

R. No … where was it fine? I felt it is fine … I have children so I will have to something… So I went to work.

I OK….

R. There I went… worked…; while working I felt profound weakness.

I For how many days were you able to work nicely? After going?

R. After going worked for 10 – 20 days in a month as I told you … I lived for 90 days there 3 months.

I Were you able to work for 90 days?

R. Yes… I could work for 90 days worked then took a leave

I Took a leave.

R. Yes… I took medicines sometimes from the doctor who was there…

I You consulted in private in [redacted] also?

R. Yes… Yes… the doctors are there was? I said I am having loose stools. He gave medicines … took 2 – 3 tablets and it was fine for 2 – 3 days. I went 1 – 2 times. I kept working.

I How long could you work continuously?

R. Worked for 2 – 4 days then gap of 2 days.

I What problem did you have!

R. I had weakness fever the body became cold out of fever… I felt cold.

I Then you returned back

R. Yes… Yes…

I In your neighborhood, do they talk about disease?

R. No… in neighborhood … nobody knows...

I Nobody knows… (Pause)

In your knowledge, is somebody there with the same disease?

R. No nobody is there…

I Tell me a thing. When you first felt something is wrong… something is not right in the body… why did you go to private set up? Why not government?

R. Like … I had fever. I didn’t know that its kalaazar or HIV only… I consulted in private so that I am cured early.

I You feel you get cured early in private?

R. Yes… Yes…

I felt I had weakness… the place where I worked in [redacted], government doctors was there. I built his house he told that I was becoming very weak. Either consult in [redacted] nearby or go to your house. Go to house and consult in government set up… This is what he said.

I He told you then you came here. After that, you went to government set up…

R. Then I came to my house and directly went to government set up consulted in [redacted]. Then came to [redacted]. I came hence. It was he who told me this… either to get treated there on go to house and consult in government set up…

I Did you feel earlier that treatment is not good in government? What did you feel?

R. No… I did not feel anything like this I thought if I take treatment in private, I will be fine sooner…

I Why? Why did you think so? The long query and all? The patient load? Why did you think it will be earlier in private?

R. No no nothing like that. I thought it’s only fever not a very big disease. So let’s consult in private.

I Its minor illness… so is private.

R. Yes… Yes… So I get treated in private.

I How much money did you spend in private?

R. There much of the money was spent. Treatment once continued for the three months. When they told typhoid… treatment continued for three months. I felt as if I was fine…

I So how much did you have to spend in those three months?

R. Around 10,000….

I You spent 10,000….

R. Yes… Yes… Yes…

I How is your family life?

R. Everything is fine now everyone is happy earlier, I also used to drink alcohol.

I Earlier you used to drink? Before the disease?

R. Yes before the disease. Before my treatment started, I used to drink a lot… what is there to hide from you?

I For how many years did you take alcohol?

R. I took for around 7 – 8 years, people said because of drinking alcohol how much has his health deteriorated … now I don’t drink anymore now I am fine… Everything is fine…. now my family members are also happy. Children & all….

I After you came to know about disease since then you left alcohol?

R. Since then … I left….when I found out about this, then. Now I don´t touch it.

I That’s good…

R. (pause) the doctor told if you want to live your life, quit alcohol.

I Anything else... you would like to say?

R. That’s it….

I Thank you very much….
