# Supplementary material for: Quality of life perceptions amongst patients co-infected with Visceral Leishmaniasis and HIV: A qualitative study from Bihar, India
Source: PLoS One. 2020 Feb 10;15(2):e0227911. doi: 10.1371/journal.pone.0227911 (PMC7010301; doi:10.1371/journal.pone.0227911)
Supplement: S3 File — (ZIP) [file pone.0227911.s003.zip › Transcripts/Patient 13 Male Age 28.docx]

**Patient 13, Age 28, Male**

1. Yes, so tell me a bit about yourself. For how many days have you been residing in [redacted]?
2. Hm?
3. For how many days have you been in [redacted]?
4. [redacted] home? Meaning from childhood.
5. You were born there?
6. Yes.
7. And what work do you do?
8. I ride the tractor.
9. Oh tractor? Where?
10. At home only.
11. In [redacted] only?
12. In the home only. In the village.
13. I see. So, how many days of the week were you able to work previously? Did you work every day in the week or were there also days where you didn’t get work or something like that happened?
14. Hmm…like I work right…so…6 months, 1 year is how I work, otherwise I lose the tractor in the middle, right? Then, I sit at home.
15. No, all the time, I will not be having Babuji’s tractor all the time!
16. You drive someone else’s tractor?
17. I drive other people’s tractors.
18. Oh I thought it was your tractor.
19. Now how would it be mine? I’m a poor man. I drive someone else’s.
20. I see and do you work somewhere outside as well?
21. No I don’t work outside.
22. You’re always here?
23. Yes we are two brothers, two people, my brother drives a truck.

I: I see so who else is in your house?

1. At home, we are two brothers and kids. I don´t have parents.
2. Are you married?
3. I am married- 3 kids…I have 2 daughters and 1 son.
4. And are both brothers living together or separately?
5. We both brothers..live separately... from that time when our parents were alive..
6. You became separate..
7. We became separate..brother..brother separated himself..
8. I see. Are your parents alive now?
9. No parents are not there.
10. No? I see. How old is your smallest child?
11. My smallest child…how big is he?
12. Means 2 years, 3 years?
13. Hmmm…2.5 years.
14. 2.5 years, I see. Okay. So your previous year…tell me from the beginning. When were you completely fine? How many days ago?
15. This has been…it has been more than one year.
16. Means one year ago, you had no problem?
17. I had no problem one year ago.
18. Eating? Drinking? All your work?
19. Everything. I used to do everything.
20. No difficulty?
21. No.
22. Then what happened? What started happening?
23. What started…I got pain here in my waist. On the right side.
24. Waist means, what we call the bum- is that you are talking about?
25. From here..from here..I had pain..there was pins and needle sensation in my entire leg..in right leg.. After that, when I went to show it here and there, then people…

I: Where did you go? Did you go to the government setup or private setup?

1. In the private setup. I have even saved the documents from the private setup.
2. Was it a hospital or was it like those small corner doctors in the villages?
3. I first showed the small corner doctors! After that a doctor Sir at [redacted].
4. He sits there?
5. He sits, and I have saved his paper too. When I showed him, he wrote all the investigations he did. So he had written the investigation for Kala Azar-HIV. So he sent me to [redacted] Hospital [government setup].
6. So firstly, did he tell you that you had HIV?
7. Yes. He’s the one who informed me.
8. So when he said this, how did you feel hearing it?
9. My heartbeat increased..I had HIV.. I am not educated. Nurse, what is this HIV?
10. Oh you had never even heard the name?
11. I had never heard the name. The other…chote mote village doctor whom I showed it to…I told him and then he said this is a dangerous illness. I told him what happened? I don’t know.

I: What happens and everything- you hadn’t even heard the name?

1. No. So he’s the one who said- go show it in [redacted] Hospital, so then I came to [redacted]. In [redacted]..in block..then from there medicines for pain were given..medicines were started..
2. So this, meaning, the HIV medicine started?
3. Yes.
4. I see. After that?
5. After that, when I ate the medicine, right, I got a lot of chest pain.
6. How many days after you started the medicine did the chest pain start?
7. Hmm…chest pain started 1-1.5 months afterward. And along with that fever.
8. Earlier you did not have fever?
9. No I had fever! But it was slight, normal.
10. But you said first that all you had was pain in your legs and a tingling sensation?
11. Tingling sensation, like that yes
12. No other problem?

R: No. And I also had a fever. I had all three.

1. You also had fever. Was the fever present every day?
2. Yes, fever.
3. I see, and? From 7, how was your appetite?
4. I did not feel hungry.
5. And weight?
6. Where did I have weight?
7. No meaning tiredness, did your clothes feel like-?
8. Yes, tiredness- there was tiredness.
9. Meaning when you rode the tractor, did you not have any problem in riding it?
10. I did not feel like riding it. I just had the desire to sleep, that’s it.
11. So you felt tiredness?
12. Yes tiredness?
13. There was fever?
14. Yes.
15. And along with that, you had a tingling sensation in your leg?
16. Yes.
17. You didn’t have an appetite?
18. No.
19. Did you eat food?
20. Food…[​*mumbling​*]I was not even able to eat food.
21. For how many days did this continue?
22. 5…7-8 months. I have been bearing with it for one year, the leg condition and the fever-these two conditions.
23. So then when you showed it to the PMCH doctor, he didn’t start you on any medicine?
24. He started medicine.
25. But he sent you?
26. He…there is some investigation, right sister? He prescribed all these investigations. In the first one, this came out.
27. HIV?
28. Yes.
29. Did he tell you about any other illness?
30. Nothing else showed up.
31. He just told you one thing?
32. Yes.
33. So then you went-
34. He sent me to the block [hospital].
35. And then what happened?
36. So then when I went to the block, when there was an investigation there, it came out in that.
37. HIV came out. Did they tell you about any other illness?
38. Yes, in that, what do you call it…this…
39. Kala Azar?
40. No.
41. TB?
42. No, yes yes that was checked, but it did not show up then. That showed up here [in[redacted]] only.
43. I see- it did not show up then?
44. It did not show up.
45. So they only started your HIV medicine?
46. Hmm [yes].
47. And 1.5 months after that, you are saying you started having chest pain?
48. Yes, yes pain.

I: And then what happened?

1. Then uh, here when I came to Jalalpur block no…then one person told me, go to [redacted] hospital…go to the block. So when I went to the block right, took some blood out- when that happened, the Kala Azar showed up in that.
2. So they told you that you had Kala Azar in Jalalpur Block?
3. Yes they told me I had Kala Azar in the block.
4. They did not catch it at [redacted] hospital?
5. No, I did not do a Kala Azar test there.
6. Oh you hadn’t even gotten a test there?
7. No. I did not get it done, for fever and all.
8. You just started that medicine?
9. Yes I got my medicine started from there.
10. After that? When your Kala Azar was diagnosed here, then?
11. After Kala Azar was diagnosed, then that…a person reached near me at my house. In the morning. He got me here and got me admitted. Two days later.
12. Did you know anything about Kala Azar?
13. I did not know anything.
14. So you did not know anything about either illness?
15. I did not know anything.
16. That what happens? How it happens?
17. I don’t know how it happens.
18. You don’t know. And after that, when you came there, then?
19. When I came here, everything was mixed together and whatever happened.
20. So when there was an investigation here, when were you informed you had TB?
21. Fluid was aspirated from here..
22. Where you used to get the pain in your chest?
23. Yes.
24. And there hadn’t been an x-ray of this before?

R: It had not happened. Where? Nothing was done.

1. Here you were told that you have water inside your chest?
2. Yes.
3. And that you have TB?
4. Hmm [yes].
5. Okay tell me one thing- this is about your illness- when your illness had not yet started, meaning, according to you, what factors are important in leading a good quality life?

R: [​*pause​*]I do not know.

1. No think for yourself. Everyone thinks what is important to them in their life. Many people think they should have a house, money, kids should study- what do you think?
2. We all just think that we haven’t studied. I am an uneducated man...so, I don’t know about all this- how all- I have been feeling very dejected. How this illness and all happened.
3. But do you think anything like this, that- and what did you think earlier about what you wanted to do in life?
4. First, I used to think that- I used to work a lot. A lot.
5. What all had you thought of doing?
6. Nothing happens just by thinking about it, sister!
7. Even then, at least tell me-
8. I had thought I would build a house- okay? I had thought I would put color or plaster on the house- it won’t happen now. It is not happening.

I: Anything else you had thought?

R: What else? What else would I think?

1. No, no at that time. Like you used to ride a tractor, were in good health. What else had you thought that you would do?

R: Hmmm. Yes, I thought I would do all this. I would educate my kids.

1. Educate the kids? Anything else? Like there is- educating kids, or you mentioned housing, something of your own- like you used to ride other people’s tractors, so did you ever want something that would be your own? Starting a business of your own- had you ever thought of anything like this?

R: No, I had not thought about all this.

1. Not thought about it. So you just thought that the house should just be painted, and you will educate your kids.
2. OK so now what do you think? What all things will you be able to accomplish from this?
3. Now what will I be able to do? Now I am not able to do
4. You are not able to work?
5. No.
6. You are not able to ride the tractor either?
7. Yes. I will- I will ride the tractor now, but at present, I am sitting at home.
8. Since how many days have you not worked?
9. I haven’t worked.
10. Since how many days?
11. Since the treatment has started, no? Since then.
12. Since when is the treatment happening?
13. I haven’t kept the papers.
14. Approximately? 2 months? 4 months? 5 months? 6 months?
15. Mmmmm…I have stayed approximately 1.5 months [for treatment].
16. You’ve stayed for 1.5 months? So you have not ridden the tractor for 1.5 months?
17. No. And after that, after discharge, I have been sitting at home.
18. I see, so even after getting discharged from here, you are not able to ride it?
19. I have not ridden it.
20. I see, so how do you manage the household expenses?
21. Household expenses..I get some money from here and there..the loans.​(inaudible 11:00-11:06​)
22. No she does not work. She goes to work for other people.
23. Like?

R-The house is there na?

I: Yes

1. There only she goes..here and there to work..
2. Household work? cleaning and all?
3. No..not all that..in farms and all..cleaning of grasses..sowing
4. Yes..yes..sowing ..for that she goes?
5. Hmm
6. And do your children work at all?
7. I have small, small kids.
8. No one older among them?
9. They are approximately 4-5 years old. So they are that big.
10. Then they’re small. I see. Then, how is the living environment around you? Like how is your house? Is it a mud and thatched house?
11. Ceiling is not cemented?
12. Its made..not cemented..
13. Oh..not cemented..
14. So tell me this- when you were told this, uh, what kinds of illnesses you have – what have you been doing since then? What have you been thinking about yourself?
15. Now what am I thinking? I will look…if I get work or business anywhere, I will do it.
16. What kind of work?
17. My health will be alright, only then, right? Now I don’t feel like going anywhere.
18. You don’t feel like it?
19. No.
20. Not feeling like doing any kind of work? [​*question is met with silence​*]You have told me that you’ve not even started riding the tractor yet?

R: No I have not started.

I: Do you feel like you can start something once you leave here?

1. I think about it…something…but they tell me not to do it, my folks at home. That don’t ride it now, leave it.
2. I see, and who else did you tell when you find out about your illness? Did you tell your wife?
3. Yes she knows.
4. She knows. Anyone else? Your brother?
5. Yes one of my brothers knows.
6. Your brother. And the brother’s wife?
7. She also knows.
8. I see, and the kids? Well, the kids are small.
9. You know my girls? They have been checked- they have it [HIV].
10. I see. They don’t have it. And your wife?
11. She has it.
12. I see…then you…so she must be taking medicine also?
13. She is also on medicine.
14. From that block only?
15. Yes, [redacted].
16. From [redacted] block, I see. So tell me this- the place where you live…are there other people there who have a similar illness? Your neighbours who you may have heard about? [​*pause​*] Someone who may be on medicine, or someone you may have heard about, who may have had this problem?

R: This I do not know, sister.

1. No? Don’t know about anyone in your neighbourhood?
2. No.
3. I see. Then, your treatment that started- first it started for HIV?
4. Yes.
5. And after that, Kala Azar?
6. Kala Azar.
7. You said Kala Azar brought you here? [​*long pause​*]To what extent are you satisfied are you with the treatment that is ongoing here?

R: It is fine.

I: Any trouble here?

R: No, no. I do not have.

1. How is the behavior of the staff towards you?
2. Whose?
3. Like the staff who give medicine, the other staff, doctors- how is their behaviour towards you?
4. It’s good. It is very good.
5. Very good? Any trouble?
6. No there is no trouble at all.
7. No trouble. Have you gotten any assistance from the government anywhere? Any money you have gotten or anything?
8. The Sirs have told me to give account number..I have not got my account opened yet..
9. So get an account opened?
10. Where do I have the money?!?
11. Nowadays, you do not need money to open an account. You can open a zero-balance account too.
12. I have opened a zero-balance account, but the link is broken. I have opened it 1-1.5 months-you know where you put your thumb? That is not working. I was told now it’ll happen in 1-1.5 months time. You will get it then.
13. I see, so get that done, because you will only benefit from that. Okay? So now what do you think? Like it is you and your wife both. Both of you are eating medicine. So how do you think-what will you do going forward, meaning for your kids, or what do you think for yourselves or for your wife?
14. What will I think? I will see…this medicine- I do not know for how many days it will be required.

I: The medicine will be required for a lifetime.

1. No, sister, for this…uh, the water that has come out, right?
2. For TB?
3. Yes.
4. For TB, 1 year…it is required for 6 months-1 year. Then, you will be checked and if nothing comes out, then it will be stopped.

R: It has been six months..six months has completed..

1. We will see it again after checking it.
2. For this?
3. Meaning this, or sputum or we will see with some other investigation. Whether your germ has gone away or not. After that, we will test. For HIV, you must know that the medicine will continue for a lifetime?

R: Yes I have heard, people have said that this medicine continues for a lifetime.

1. Yes and this Kala Azar that is there- that medicine also finishes, meaning that medicine also

does not run for a long time. That is also done for you. So now what do you think, like what work will you do, whenever you feel a bit…a bit better then?

1. If I get better, then I will do a little bit of work. It will not be possible like before- will it?
2. No, you tell me. What do you think?
3. I will drive a vehicle only, I will not be able to do other work.
4. So if you get better, you can drive the vehicle?
5. Yes, I will ride that only. I get 25-30,000 rupees.
6. In one month?
7. In one month?
8. I see…and the kids, then? Does he study anywhere?
9. Yes, they all go to the government school to study.
10. In the government school…
11. Youngest..(corrects)..middle daughter goes to aanganwadi.
12. The one who is younger than 3 years of age?
13. Yes, yes. And the small son, he is not capable of going around anywhere right now- he roams around. He is very small.
14. I see. And your wife does not have the other illness, no? Kala Azar and-
15. No, she doesn’t have that.
16. She just has HIV?
17. That’s it.
18. So she doesn’t take medicine for anything else?
19. She does not eat.

1. I see. Would you like to say anything more? Would you like to change anything else? Anything that you think would be better if it happened this way instead of that way? [​*long* *pause from patient​*]Where your treatment is happening, do you desire anything more from there? Anything more?
2. From here?
3. From [redacted]?
4. Its good..I meam to say that..sister..is there an injection for this?
5. Yes
6. Uh?
7. Yes..
8. If I get that..shall I not be fine?
9. Why won’t it get better? If you keep taking the medicine, you will be fine.
10. This won’t be finished?
11. HIV? [​*patient says yes​*]. HIV germs do not get finished like that. Once it gets in the body, but many days- if you eat the medicine properly, you can live for a long time healthily. It’s not like that. Okay? [​*patient sounds uncertain​*]There are other important things with this, like paying attention to eating and drinking properly- you have to eat well, like you should eat nutritious foods-
12. [​*pauses​*]Now I eat alright. The sisters [nurses] have said to eat after taking the medicine at 9 am. Hmm…that you have to eat it at 9 sharp. So I eat at 9 by looking at the clock.
13. Yes, so then you will be alright. If you are eating medicine properly, eating food, people stay alive for a while- nothing happens like that- in this [illness]. It’s just that you will have to think ahead- you will have to plan for the future- what all you have to do. That’s what I want to

know from you- what do you think about? What do you think about the future moving forward? … What more things could you do through which she could get better? [​*long pause​*] Would you like to say anything more about this?

R: I don’t have anything else.

1. So when you found this out, what did your wife say to you? How did she…you must have told your wife?

R: No they told me in [redacted]- that you have to get everyone tested.

I: So you must have gone to inform, that they are calling. So what did she say to you?

R: Document was there in [redacted]..in block..it was diagnosed..I took it and submitted in block.

1. That is fine. But when they called your wife, your wife must have known she is being called for this reason. Did she know about HIV?

R: No, she-

I: She also didn’t know?

1. Didn’t know. I took everyone, son and daughter, took everyone and went. So two daughters have it- she also has it- they don’t have it now, both daughters are okay. She [wife] has it.

I: The son?

1. The daughters, the two daughters I have, no?
2. And the smallest son didn’t get it?
3. For him..sir in [redacted] has said- he will not have it now..he is small kid..his disease cannot be diagnosed..let him be 2-5 years of age..then his test will be done..
4. Okay, so your wife- what do you talk about amongst yourselves?
5. No, now we don’t talk.
6. No, you must think ahead, right? When she found out that she also has HIV, so what did she-how did she react with you?
7. [​*long pause​*]
8. Did she say anything to you? That she is sad about something, or she said something.
9. [​*long pause​*]
10. Would you like to say anything about this?
11. How did it happen- now how do I know?
12. No no, not how the illness happened. How did you both feel when you heard, meaning that

she also got it [the illness]? Or that you have HIV- your family members like your brother-when they heard, in what way did they show their sadness? [​*phone ringing​*]

1. Meaning what did he say to you?
2. My brother?
3. Yes.
4. Like he said- when he saw- when he heard, he said- its not that​(mumbles​)..the blade that is used for shaving..that also causes
5. Yes, no no, it happened that’s no problem- what did he feel after this happened, meaning that…okay he said it can happen from shaving?

R: Yes.

1. I see. So did your brother say anything to you? What- any sadness or-
2. No...ofcourse he was sad.
3. That is what I’m asking. Was he sad?
4. Yes, he was sad.
5. And your brother’s wife?
6. She also was.
7. And your wife?
8. Hmmm…she must be. [​*looks uncertain and unwilling to talk​*]
9. Okay. Would you like to say anything else?
10. What more can I say?
11. Okay okay, well thank you very much.
