# Supplementary material for: Quality of life perceptions amongst patients co-infected with Visceral Leishmaniasis and HIV: A qualitative study from Bihar, India
Source: PLoS One. 2020 Feb 10;15(2):e0227911. doi: 10.1371/journal.pone.0227911 (PMC7010301; doi:10.1371/journal.pone.0227911)
Supplement: S3 File — (ZIP) [file pone.0227911.s003.zip › Transcripts/Patient 15 Female Age 64.docx]

**Patient – 15, Age – 64, HIV VL-TB**

I - So, who else lives with you in your home?

R - 4 children.

I - 4 sons?

R - No, 2 sons, and 3 daughters, 1 daughter has got married.

I - Is son younger or elder?

R - This son is on 2^nd^ birth order then a girl then a boy and then a girl.

I - So, what work do you do now, mataji?

R - No one is there

I - You don’t do any work? No, what do you do? Or where from do you get your living?

R - I used to collect the clothes, wash the clothes, and then fold them.

I - Ok, You wash clothes?

R - Yes

I - You iron those clothes also?

R - No, No……I only wash clothes, and accept grains wrapped in clothes. This much grain [*showing both hands*]

I - They give grains for that?

R - Yes

I - Do you also do any field or farming work?

R - When I was fine I used to work with “Khurpi” [a tool for digging soil]

I - Ok, so when you were fine, you used to harvest crops with khurpi and sow the seed as well?

R - Yes. Yes..

I - The wags you used to get from that?

R - Yes

I - Do your other sons work?

R - Yes that son does work of ironing after his father passed away. He earns and ask me to leave the work as I got weaker.

I - What does your son do?

R - Ironing

I - Do other children study?

R - Yes, one is studying and other are at home

I - Ok, Tell me about your disease, How long back were you apparently well?

R - 7 months.

I - 7 months before you didn’t have any symptoms?

R - Since (Kartik) month

I - Ok from (Kartik)?

R - Yes, Fever started and I was not feeling well so, visited a doctor but was not relieved.

I - So, where did you go first?

R - [redacted]

I - [redacted], in [redacted]?

R - My house is in [redacted].

Other person – It is a block.

I - So you visited private or government?

R - Private

I - Why did you want to private why not government?

R - Didn’t go

I - Didn’t go

R - Yes

- So, what checkup you had in [redacted]. Was he a doctor?

R - Yes

I - What did he say?

R - He tested my blood, and diagnosed me with seasonal fever.

I - He told seasonal fever and tested blood? Anything else?

R - Yes, Nothing else.

- He prescribed medicine, got fever, did not get down.

I - For how many days did you go to the doctor at [redacted]?

R - 4 times

I - Over how many months, did you visit doctor at [redacted]?

R - 1 month

- When I didn’t improve despite 1 month of treatment, I went to my mother’s house.

I - Where your mother’s house?

R - [redacted] in [redacted].

- (Other person: After that she went to a government hospital as well)

I - Where is that government hospital?

R - In [redacted].

I - Why did you go to [redacted]?

R - One person took me to [redacted]. Nothing was told there, an injection was given.

I - What did they do in [redacted]?

R - They gave me medicines, did my X-ray and some blood tests.

I - Yes.

R - A lot number of medications and injection were given, but my symptoms were not relieved.

I - So, in government hospital also, you were given medications and injections but didn’t get relieved?

R - Yes

I - For how many days you visited government hospital?

R - One time

- (Other person: For 3 weeks)

I - Where did you go after that?

R - Then I came to [redacted].

I - Where in [redacted].

- (Other person: [redacted])

I - To any privation practitioner in [redacted]?

R - Yes.

I - What did he do?

R - He only investigated, and gave medicine

I - Did no one tell you about your disease?

R - No

I - Was nothing took at these 3 places and government hospital as well? After testing cured, too, did they not tell diagnosis?

R - No

I - Then, what happened, when you came to [redacted]?

R - He only gave me medicine of fever, and I had it. He charged Rs. 400/- twice. But fever used to recur soon.

I - So, two times you had to pay Rs. 400 (----, but was of no use/benefit?

R - Yes, no benefit. The doctor asked me not to come here- it is closed now. Go to medical. And then I started crying-

I - So, medical is [redacted]?

- (Other person – Yes)

I - So, how many months had passed in all this?

R - 4 months.

I - How much have you spent in private?

R - बाप रे बाप! Rs. 30000/- and more.

- Son used to send the money and all was getting used. I said to the son, that I will not survive the disease.

I - Does your son work faraway?

R - Yes, this son only (the other person)

I - Where do you work? (To other person)

R - In Kolkata.

I - So, you used to send money from there?

- (Other person – I took loan from there, and then used to send money)

I - Ok!

R - There is only me, and my 4 children, and no one, else is there to take care of us.

I - What was the first thing that happened when you fell ill? Fever?

R - Yes, only fever

I - Was fever persistent?

R - Yes, fever was persistent. Fever was accompanied with chills. When I used to take medication, or cover me up. I used to get some relief, just to recure. Then I said to my son, “I am not getting relieved and I am not going to live. Do come here, if you want me to live”.

I - Was there any problem other than fever?

R - Yes

I - What else?

R - That,……… only there was itching in my vagina.

I - Did you also have bleeding ?

R - No, Nothing

I - हौआता means what? Itching?

R - Yes

I - Any other problem?

R - No

I - Blood in vomitus , or cough?

R - No, cough has now occurred with fever.

I - Has cough occurred with fever now or earlier?

R - Long time after fever, and after starting medication then cough appeared.

I - Was there any weight loss, or clothes got loose?

R - Yes, Yes.

I - Appetite?

R - No

I - In beginning of the disease.

R - No, I had go blind, 2 other people used to carry me.

I - Ok! Could you not see?

Other Person - She could not see by her left eye.

R - Nothing, I was doing no work since the month of Kartik. I food was no more pleasant to me. Now I eat everything.

I - So have you been sent here form [redacted]?

R - Yes

I - For how long have you been getting treatment here in [redacted]?

Other Person - We came here on 6 march)

I - So, it has been 10-15 days?

- For how long have, you been admitted here?

R - Till now

I - So, you are admitted here since 6 march?

R - Yes

I - Since you have been feeling better than earlier, have you been told what has happened to you?

R - I don’t have any problem right now.

I - Has it been told about the disease she’s suffering from ?

- (to other person)

- (other person) – yes

I - Do you know what disease you have?

R - Yes, 2 diseases.

I - Ok! Tell what disease?

R - TB and HIV

I - Have you heard of these disease before?

R - Yes, I have heard it in village.

I - What have you heart? TB or HIV

R - I have never suffered from TB before

I - Have your heard its name?

R - Yes

I - How did you feel, when you got to know about your disease?

R - Only itching of HIV

I - How did you feel, good or bad?

- Did you feel anything?

R - Yes, I felt agony.

- Probably I did something wrong to God, that’s why it happened to me.

- I had good health, when I was young

- I earned alone after my husband passed away. My son had also started earning at a very young age.

- There was no home. Roof was leaky, we got our home made.

- Fear of all those led to HIV

I - Did you know about your HIV earlier?

R - Yes

I - When did you got to know?

R - I got to know it 1 year back

I - How did your husband die?

R - He died of many diseases?

I - How long back did he die?

R - 4 years back, in 2014

I - How did he die?

- Other person – He was diagnosed with HIV in 2014, when he was about to get treatment, he got cancer.

I - At what site?

R - Oral Cancer

- (Other person- he came to Patna, and developed nerve pain, so he was referred from Patna that he can’t be cured. So, we brought him back to home, and he passed away in 5-6 days.

- We he passed away in 5-6 days.

- We didn’t pay attention to his HIV.)

I - So, was he not diagnosed with HIV then?

- Other person – we didn’t know that openly.

I - To respondent, - did you know about his HIV?

R - No, I was a fool, I took him here and there. Someone told me to go to Patna.

I - To other person – were you not there at that time?

R - He (Son) was for away earning.

I - Was he younger?

R - Yes

- The cancer that had happened to him right?

I - (To son) So you thought that he died of cancer?

R - Yes

I - Was she not diagnosed with HIV, when her husband (To sun) was diagnosed with HIV?

R - No

I - When was she tested 6-7 months back?

- Son – Yes

I - But she said, she knows here HIV for last 1 year (to son)

Son - She didn’t tell me.

- I to respondent – How did you get to know?

R - When I went to [redacted].

I - So, what was the problem?

R - I was getting itching. That’s why I got testesd.

I - How as your health then?

R - I had fever

I - I year back?

R - Yes

I - She is saying that she knew it for last 1 year

Son - She might be knowing, but she didn’t understand what is HIV.

R - I knew, but I didn’t tell, for the fear of he getting tensed to hear the news.

I - So, how long back did you know about your HIV?

R - 1 year

I - So, you went to [redacted]?

R - Yes.

I - So, did you develop fever then?

R - Yes

I - So, you didn’t get treatment on knowing that you have HIV?

R - No

I - Why?

R - Due to financial issues.

I - So, you thought, it would cost you money?

R - Yes

- My younger son had got his leg fractured, so money was spent in that.

- So, I was afraid, and didn’t tell anyone.

I - So you told when you feel ill?

R - Yes, When I got bed ridden, and could not see, then I told my son that I will not survive the disease.

I - How was your last year?

R - It was fine

I - How was this year?

R - In the beginning I was fine. I could wash clothes of customers.

I - Have you told about your disease to anyone in your home?

R - No

I - To your other children?

R - Yes

I - How did they react?

R - I felt a lot like…how this happened. [*inaudible*]

I - Have you heard of anyone else having this disease?

R - One person had. He went.

I - Went or died?

R - He died.

I - Anyone else?

R - No

I - Have you told your neighbours about your disease?

R - No

I - Didn’t you pay attention when you got to know about your husband’s HIV?

R - No, I just started earning after he died.

I - What all do you think is needed to lead a good life?

R - I think that my son should remain until i….

I - Not of now but when you didn’t had the disease? It happen like some people need money or you had though of getting repaired your house. So what do you think should be there? Some work you wanted to do?

R - Yes

I - What work you wanted to do?

R - I wanted to do every work as my health & body allowed. But for six month I am not able to do work.

I - How much were you satisfied by your health at that time?

- In one rupee How much percent?

R - In one rupee…… I don’t understand; my son used to earn but it wasn’t enough to feed.

I - Did you ever feel life is meaningless, I should die, Why am I alive?

R - No, no, no I had never felt like that. As soon as I found out I had HIV, I started realizing the kids will be orphaned. Their father is no more, grandparents are no more, and even now I will not be around.

I - No one is there to book after?

R - No

I - What do you think the treatment, medicines, and doctors you have met, and the health facilities you are getting are adequate, or is there anything lacking?

R - My nephew from mother’s home took me here and there everyone is good.

I - Ok! How were the different doctors you have visited?

R - That’s what I have said.

- At last when I got blind, two……and other people started saying, that I am not going to survive.

- Then one man said that he knew a man, who went to Patna, and survived Then I asked my son to take me to Patna.

I - Who told you about a man who survived when was taken to Patna, and about whom was it concerned?

R - A man whose clothes I used to wash told me.

I - A man whose clothes you used to wash told you that you’ll be cured if you go to Patna?

R - Yes, yes. My aunt’s daughter was sick. She went to Patna and became alright. My real sister said that I won’t survive, and if that would happen, then my four kids will be orphaned. There is no one to look after them. I told my son- take me to Patna, take me to Patna. I didn’t even let him finish eating, and I said take me to Patna, take me to Patna [*crying, visibly upset*]. Then my son went to another person’s house and ate there, did not eat at my house. My son…this son left everything and stayed with me and took care of me, brought me here and gave me a new life. What else can I say? [*smiling*] Now I take pleasure in eating.

I - How do you feel now?

R - I am Ok

I - Food & Appetite?

R - Food & Appetite are good now.

- My appetite has increased.

- I have not been eating salt since I got fever.

I - Why were you not eating salt?

R - Due to fever, salt didn’t taste pleasant.

R - Since I have come here, and started medication, I have started eating everything. Now whatever I get, I eat everything. When I get better and get discharged from [redacted] and go back to my place, people will see me- they used to say that I won’t be alive anymore. I won’t be alive anymore. And I used to say [to my son], take me to Patna, take me to Patna. Then my father in law died in between. Although he was well. He used to say I am there for you, but why my daughter-in-law is behaving like this? [*Patient had become unstable in between and worried family members*]. Since my father-in-law, I got delayed in reaching Patna, otherwise my child would have brought me here earlier. I rented a car and I was not aware where I am going, and where he is taking me. I was not able to see, but now, I can see everything.

I - Could you not see?

R - Nothing

I - Could you walk without support?

R - No,

I - No at first?

R - Somebody used to hold me.

I - I see, by holding.

R - Yes, by holding. I was shown my father-in-law’s face. I did not cry. Now, when I hear it, I feel bad.

I - Your son was also telling that your mental status was not well at that time.

R - No no, it was not good.

I - You could not understand your Father-in-Law passing away also?

R - I could not understand, where my Father-in-Law had gone.

R - I came here, had food, and got some health back, then I realized he has R passed away. (Crying)

R2 - Whenever she remembers, she starts crying.

R - Now when I miss him, I start crying.

I - His Ok! You were not able to understand above his death that time.

R - I could not cry then. (Crying while saying)

I - It is Ok! When you yourself are not well , how can you help others?

- Now more important is to take care of yourself first.

R - Yes

I - What you were experiencing at the time, were you also having pain?

R - No, no pain

I - Ok!

- Beside this, did you ever require, or had treatment?

R - No, no

I - Never you fell ill earlier?

R - Never

I - Were you happy earlier?

R - Yes, Yes

I - Could you do your work?

R - Yes, I was happy, earning, and eating. I could work in field using khurpi.

I - Didn’t you have any problem at that time?

R - No, I had no problem.

I - Did you feel safe or secure? How do you call it in your language? Didn’t you have any fear of somebody harming you? Were you ever afraid of someone or your neghbour of any harm?

R - No, no

I - So, your neighbours are good?

R - Yes, they are good.

I - Is anyone bad among them?

R - No

I - Ok!

I - So, do you feel you are better now?

R - Yes

I - Your appetite has also improved?

R - Yes

I - What do you want to say about the treatment you are getting here? How the doctors and staff behave with you, Do they talk well?

R - Yes they are good.

I - Are you satisfied with your treatment?

R - Yes, I am happy.

I - Do you want any changes and improvement in the treatment given here?

R - Yes, Yes

I to Son - You say do you want the change in treatment from here.

Son - The improvement I want is that she should be cured for ever after getting treatment from here.

R - Yes, only that.

I - For ever means that you don’t have to come here again?

Son - Yes

R - Yes

I - So, for now they have called you for follow up?

Son - After one year. After 5-6 months.

I - After 5-6 months, after one year?

R - Yes.

I - Why do you want that?

Son - I experience a lot of trouble coming back and forth. She’s alone at home.

R - I am alone at home.

I - Anything else? Do you face any loss of wages or anything?

Son - There will be loss of wages- I don’t have expectations of her working. She’ll sit and she’ll eat.

R - My son says that he won’t let me do anything.

I - Taking here and there…

Son - There is some difficulty, yes. I don’t stay here. I have a small, 10 year old brother.

R - He [my son] is eager to earn.

I - Other than this what improvement do you want?

R - From here, I want HIV to be cured from root (जड़ से)

I - How was the behavior of people here, with you, and did you live here happily?

R - Yes, everything was fine.

Son - Whatever was told to nurse, too was done quickly. When I first came here, I was worried about how it will all get done we got happy at nurses’ behavior.

- We have seen such people for the first time in hospital (good life)

I - What else do you wish to do in life?

R - Now my son wants me not to do anything.

I - So your son wishes that you should not do anything?

R - Should not do anything. He says you should just meet people, and you sit and eat at the entrance. Then only you will survive.

I - What were you saying, that you all eat at your doorstep?

R - My son says that she all eat this much only, but will sit at home, will not go to work.

Son - She thinks to get her daughter married.

I - How old is she?

R - She’s of age of getting married. She has become adult.

I - Anything else, you wish to do?

R - Whatever work I used to think about…stopped. Stopped.

I - So, what do you think can you do all these?

- Now you have got better

R - Yes, but…… how will it get done!!

I - Money?

R - Yes

I - पैसा जो है, उसी में आ जाएगा│

R - Yes

I - More important is your health.

- If your health is good, then only can you focus on other things.

R - Yes

I - See, earlier you were not able to understand anything your mental status was not good.

R - Yes

I - Now your mental status has become better. Now you can talk, you are laughing.

R - Yes, Yes.

- Now I can also see far of things

Son - She should not work at least for a year, so that like however much she has improved should not be compromised. Providing the rest is the right thing to do.

I - And she’s gotten old too.

Son - Yes she’s gotten old also, so we shouldn’t expect too much.

R - I am worried about HIV.

I - You don’t need to worry about that. You will get medicine from there also.

R - I have to take the medicine for my whole life.

I - His Ok! Now a days, people even have to take antihypertension lifelong.

- Lifelong medication is for many disease.

- There’s no problem in that

- आपको अपना मन ठीक रखना है│

- Like you take your food daily, so do take medicine.

R - Yes, Yes, I take it happily.

I - You should be healthy.

- Keep taking medicines, and you all be healthy.

R - Why would I be worried?

- I all take medicine.

I - Do you wish to do anything else?

R - No

Pt_15 continued…………

I - You were first told about HIV.

- What were you told after HIV,……… what disease?

Son - Kala-azar

I - Where did you get to know about T.B.?

Son - Here

I - You didn’t know (about T.B.) earlier?

Son - No.

I - Was there any symptom, for which you got tested?

Son - She had cough

I - You told, you didn’t have cough earlier!

Son - She didn’t have, but she was tested over there. She had a dry cough.

I - Were you tested earlier?

Son - X-ray was done there.

- But sputum was tested here, so got to know

I - Was fever persistent throughout the day, or just in night?

R - Fever was persistent, all the time.

I - Did you have night sweats as well?

R - Yes, yes, yes.

I - Ok!

- You didn’t have cough at that time?

R - I didn’t have cough in the beginning, but as my body was ravaged by fever, coughing increased.

Son - She did not have a cough in the beginning.

I - Then you started coughing?

R - Yes.

- There I was told, that you need not come back- your medication will be stopped. But fever increased further, and I was coughing heavily too.

I - So, were you told anywhere you went about T.B.?

R - Nowhere else. I was only given cough suppressant - then I could digest everything. I started eating roti and dal. I stopped eating rice [*believing a myth that it is inadvisable to eat rice, banana, or curd when you have a cough*]. I left all cold foods. Yes, because of cough, I left. Due to fever, I didn’t eat rice; for many days, I did not have rice. When I came here, I ate rice, I ate everything, and while eating…[*inaudible*]

I - Was you sputum ever blood tinged?

R - Never, never.

I - Weakness?

R - Weakness was so much that I had to take support of my son.

Son - She could not walk on her own.

R - I could not…*[trailing off*]
