# Supplementary material for: Quality of life perceptions amongst patients co-infected with Visceral Leishmaniasis and HIV: A qualitative study from Bihar, India
Source: PLoS One. 2020 Feb 10;15(2):e0227911. doi: 10.1371/journal.pone.0227911 (PMC7010301; doi:10.1371/journal.pone.0227911)
Supplement: S3 File — (ZIP) [file pone.0227911.s003.zip › Transcripts/Patient 16 Male Age 40.docx]

**Patient 16 Age 40 HIV - VL – TB**

I: What do you work?

R: Earlier I was driving vehicle ...now I left it

I: in vehicle… which vehicle?

R: Large vehicle

I: Truck?

R: yes, I was driving this many days before. When my health (*interrupted by I*)

I: How many years before?

R: 3-4 yrs. My health got bad, I started feeling weakness and I didn’t like to drive during daylight

I: Tell me about yourself right from the beginning that when were you completely well and you didn’t have any problem?

R:No, I didn’t having any problem earlier

I: in which year? How many years before?

R: 2000(*trailing*).. 2 years before

I: you already said 3-4 yrs and now

R: I left vehicle 3-4 yrs before. I left vehicle earlier

I: Why?

R: just… I didn’t felt like my work.

I: means you didn’t felt like your work for past 4 yrs . what did you felt at that time?

R: weakness…

I: were you completely well before past 4 yrs ? you didn’t have problem in driving

R: I was having strength and I was well and didn’t had any problem.

I: After that

R: I felt weakness and I don’t like to stay in daylight

I: any other problem…weight loss?

R: earlier I was of 64kg and 200 gm.

I: Earlier means when?

R: 4 years before… 1-2 months before past 4 years. After that I was having fever and itching over here (*referring to a location*)

I: itching

R: yes, fungal infection over here. So I got checkup in [redacted]. I don’t lie with you. I was having minor fever so they were telling it is malaria or typhoid or other things.. I had taken medication and got relieve for few days and then it recurs.

I: you was having papules over skin at that time?

R: no, I was not having papules

I: then fever?

R: no, Itching

I: So, you didn’t have fever but only weight loss and itching

R: My weight became 53kg

I: your weight became 53 kg from 64kg so you lost 9 kg

R: no, not 53 kg but 63

I: 63 then you lost only 1 kg.

R: At that time I was having itching and got checkup in [redacted]

I: In [redacted]…Private or government?

R: Private and then they referred me to government and I got medication from there.

I: From private you were referred to government setup?

R: yes, investigations were done over there and medications were given.

I: were you informed about what you have?

R: HIV

I: How many years before you went to government hospital?

R: 2 months before.

I: now or at that time

R: no at that time, 4 years before. I have all the reports, it’s almost 3 yrs from now. You can see that ,I don’t remember that much.

I: I’ll see that

I: At that time you was having weakness, itching then you went to pvt and they referred to government hospital. Did they told you how to take medicine?

R: I have to take this at 9:00 O’clock. I have taken medicine and I don’t have any problem.

I: you don’t have problem. Everything is fine?

R: I am having some strength now.

I: it’s now but I am asking about that time.

R: no, I am having better strength than before.

I: let’s start from beginning. At that time you had started taking medicine for HIV, are you going to take medication?

R: I’ll go on 26^th^

I: Were you doing your work?

R: Yes, I am doing my work now.

I: what happened after that at that time? I am asking you all about 3-4 years before.

R: At that time I was driving vehicle.

I: Means you was taking medication and driving vehicle also

R: At that time I was not taking medications.

I: but did you knew that you are having HIV?

R: No, I didn’t knew that. When investigation were done then I found out.

I: But when you knew about that then you left your work or?

R: left my work

I: after that you were doing nothing

R: no, I do some work at home, at your or other house.

I: Farm work?

R: yes

I: means you seed some grain…what all you were doing?

R: I grow some green vegetables in field and then sell it. It’s village area, what can be done. We are poor so anyhow I have to sustain my family.

I: How has this past year been for you?

R: Fine

I: means in past years your health was good

R: when I was having Kala-azar (interrupted by I )

I: you were having HIV and you were selling vegetables then what happened after that

R: I was fine then after some days….I was bedridden for 1-1and ½ yrs then I went to Hajipur for checkup where I was told that I was having typhoid.

I: you found about HIV then after

R: after many days

I: 1yr or 2 yrs?

R: After 2 yrs

I: After 2 yrs you was having fever or what happened?

R: yes I was having fever with chills then I undergone some investigations and I was told that I was having typhoid so I was having tension that I am taking injections for typhoid, Malaria and some are telling that you go here or there and went everywhere where they told me but they were telling that I was having typhoid, no one was telling Kala-azar. I think that when I am taking injections for typhoid then why I am still having fever with chills. This was my tension. I was shivering due to chills and put blanket over the body even after that I feel very uncomfortable. You don’t trust but I abuse God even and ask for my death.

I: means that much pain

R: yes that much severe that my weight become 41 kg.

I: your weight became 41 kg from 64 kg.

R: Yes, I measured my weight here. Many people like my elder brother told me that [redacted] is very good doctor and regularly visit foreign so go there for checkup instead of [redacted]. When I went to Dr. [redacted] he referred me to [redacted].

I: Did he told you there that what is the disease?

R: he done test for Kala-azar there

I: what were the investigations they had done there?

R: they investigated for every disease, HIV was reported and then Kala-azar but TB was not found out there. Here sir has investigated, examined hydrocele and the report came from outside then he started medications. I take medicines from [redacted].

I: you didn’t know about TB but only about HIV and Kala-azar. When Kala-azar was diagnosed even after that was your fever with chills same like before?

R: No, that was ended.

I: that was here, no?

R: on the day I got admitted here due to God where my fever has gone, I don’t know. On Friday at 3:30 I was admitted and god has taken all my problems. From that time onwards I don’t have anything and all well. Work (interrupted by I)

I: here you was told about TB after investigation?

R: hydrocele get enlarged and having that much large (indicated by hand) swelling. People from village told me that hydrocele is enlarged so give warm compression by burning cowdung. I told this to doctor also but he told me that antibiotic is given to you… 14-14 injection cost Rs.400 were given so it would resolve but didn’t resolved. Here Sir came and checked me, he had taken pus from that swelling and sent outside for test and when report came then I found out.

I: From where injection cost Rs. 400 was given?

R: [redacted]

I: when you was having fever with chills…that time?

R: yes, at that time

I: At that time you was also having Hydrocele?

R: yes, both occurred at same time

I: when you went to Dr. [redacted] did he examined your hydrocele?

R: No

I: he told you that you are having Kala-azar and send here.

R: Yes he send here

I: Other than this did you have any problem like cough?

R: No, no cough

I: Blood stained sputum?

R: No, nothing. I am not having cough, no sputum, everything is well and I don’t have problem.

I: at that time when your health was not good

R: not at that time also

I: you was having weight loss, fever, itching and hydrocele, anything else?

R: Sir has given cream that causes relieve. Sir told me to use coconut oil and that gives me relieve. That cream resolve burning sensation. I came here after every 15 day. On 27^th^ I went to [redacted] to take medicine, I am taking medicine from [redacted] hospital and there I was told that this medicine continues for one month, everything is well and there is no problem. He had done checkup and told that. I am having medicine for 3 more days up to 26^th^ but on 25^th^ there is strike…people in the bus was telling so. I’ll go on 26^th^.

I: How has the past year been for you before coming here?

R: My health was not good…7 months my health was not good having fever with chills and I was very weak. No one in the village think that I will remain alive. Whatever little land I was having I mortgage it. Went for checkup…we are poor so from where do we take money. So, I went for checkup…no facilities was available. People advised me to go there and then I went to [redacted] where sir told me that here you will be treated from outside and you could have to spend money so you go to Patna. I was never admitted in hospital…I came to [redacted] and get admitted. Everything going well now. First time when I went from here my weight was 59kg and 900gm.

I: 3 and ½ yrs before when you came to know about HIV then how did you felt? Did you know about HIV, what is this disease?

R: I came to know about this after itching

I: when you came to know about HIV

R: It came in report

I: how did you feel at that time?

R: At that time I was having itching. I went for checkup to [redacted] because my itching was not decreasing… fungal infection, he had written investigation for HIV so I undergone that test. In that test it was found out and then he referred me to [redacted].

I: did you told about this to someone?

R: No

I: what did you think how you got this disease?

R: I was thinking how and from where I got this disease. My health got deteriorated in [redacted] district (recording paused)and there local doctor has given me some injections by syringe, how it occurred or not ? from that time I feel like it occurred because of that.

I: who else is in your family?

R: Mother and father is not alive.

I: your wife? Are you married?

R: Yes, I am married and have 3 children. They all are tested and they don’t have disease.

I: did you told this to your wife when you know about your disease?

R: she was also tested but she don’t have disease. We are sitting together but not doing sex. My bed is separate. It may occur due to mosquito bite doctor told me this, after that I am very cautious.

I: Have you told about your illness to everyone? To Mother?

R: No, no one knows in my family but only elder brother knows about it.

I: when your wife undergone for test then she didn’t know why she was being tested?

R: No, She was just tested in [redacted] government hospital.

I: for what reason she was being tested she didn’t knows about it. Only your brother knows about it and no none else.

I: When your brother heard this then what did he told?

R: He told that “it may happen by any mean and I don’t know how it happens”.

I: Any one in your neighbor having the same disease?

R: No

I: Kala-azar? TB? HIV? Anything?

R: No

I: do you heard about anyone like you friend?

R: No, I don’t knows about it.

I: First you started taking medicine for HIV and then you got TB and Kala-azar for which you came here.
What are the things required for good quality of life?

R: I become well, health get well, till my life ends I live well that’s what I want.

I: anything else required for god quality of life? When you was disease free at that time?

R: when I was not having disease then I think I shall do this or that.

I: what do you think?

R: To make my house, bricks are also brought. To make my house and live in better condition…family, children and all live well. I bought brick by earning.

I: After that? Now?

R: Now I don’t have courage, my health deteriorated, when Kala-azar occurred after that I don’t have courage. I have eaten food, taken juice of beet….I grow beet. I have 2 kattha land and I have also grown beet on it…I eat that every morning.

I: Do you feel anything other than that? Did you have any pain?

R: no, I was not having pain.

I: Did you have any feeling like my life is worthless, I should die?

R: I was having very much tension.

I: when?

R: before and after having Kala-azar. When Kala-azar occurred and I was having fever with chills then I lost my courage and I felt like I will not live more. I sat in squatting position, my whole body turned black, my body lacks blood, my nails turned black then I think that I shall not be remain alive. I told to my elder brother that I shall not live more and take care of my 3 children.
I have kept some money about Rs.22,000-30,000 in name of my daughter and I will not live more I told all this to my brother. If I didn’t came to [redacted] then I would not be alive then. The situation was too bad. No one knows that I was having Kala-azar. When I went to [redacted] then I know about Kala-azar.

I: when you came here then you know about TB?

R: after 15 day I came to know about it when report came.

I: How is your living environment? Are you happy with it?

R: Fine

I: Neighbors

R: Good

I: which type of house you have?

R: House made of bricks

I: do the place where you live is good?

R: Yes

I: are you satisfied with the doctors around you?

R: yes, people from block go there for checkup.

I: Are you happy with it?

R: yes, we go for investigations there and for what is the problem or not…

I: How much do you feel yourself protected? Means do you think that you are safe? Happy?

R: Fine, there is no any problem. If I have any problem then I tell this to sir. If I don’t have any problem then how do I complain to Sir. Sometimes I complain him about itching, he has given cream for that and I don’t have any problem. After taking medicines I don’t have problem. I was worried with itching for few days. 2 months before I was not able to sit, not able to eat. I ate foot by standing due to itching.

I: you was having trouble in sitting, was it due to Hydrocele?

R: No, there is no problem due to that. Now it got shrunken.

I: No, earlier?

R: no, earlier it was bigger and causes pain sometimes then pain subsided.

I: Are you satisfied with the treatment going here?

R: Yes it is good. For me it’s like God. It has given me a new life. (R2 repeat)
I was like dead, my situation was too bad. Villagers didn’t think that I will remain alive. In village two persons one me and other having heart disease who died people think that both will die soon. Due to my behavior everyone was feeling so regret. Everyone weeps after seeing me. I made everyone happy due to my good behavior. I was not doing substance abuse….I drive vehicle for few days due to my own wish. I learnt driving in [redacted] by wish after paying money. I made license also. I was driving small vehicle and then people said that large vehicle has more earning. What earning?... nothing. Everyone die due to lack of food, slept anywhere on the way, caught in traffic for long time. Do all pain ends with money only? I became well but person having heart disease died 1-1 and ½ month ago. In Delhi his cranium was opened… he was son of Mukhiya.

I: so you are satisfied with the treatment?

R: it is too good.

I: now you are well so when you go village then what do you want do?

R: I think that I live well. I shall do some earning to construct my house.

I: you was saying that your house is made of bricks.

R: it is made of bricks but it is very old, broken from somewhere. The wall is of 20 inch and made by my grandfather.

I: you want to construct house, anything else you think that you will do?

R: I think that I shall do business of green vegetables, not do any heavy work. I shall sell vegetables in market. This is what I am thinking nothing else.

I: are you thinking anything about your children?

R: I want to educate my child.

I: Do they studying somewhere?

R: yes, one is in 4^th^ standard and he is in grandmother’s house. I don’t have money in my hand and money is needed for education.

I: rest 2 two of them?

R: they are at house, one of them goes to government school.

I: Do you want any change in treatment here? If this would be there then it would be better?

R: if it improves then it would be better.

I: not in your illness but in treatment which is going there

R: treatment is going on and everything is fine. There is not any problem, when sir came he examines me completely … my back, my abdomen. He has examined me now also. For us they are like God, I would not remain alive. When I remember about all this then I started weeping. When I land up in [redacted] then I took 2-3 hours to reach hospital.

I: No one was accompanying you?

R: elder brother was there. Whenever I remember about all this then I started weeping. All this things gone now so leave it. I always remain tension about this.

I: Anyway, you are well now. Take medicine on time and anything else you want to say?

R: no, nothing.

Thank you
