# Supplementary material for: Quality of life perceptions amongst patients co-infected with Visceral Leishmaniasis and HIV: A qualitative study from Bihar, India
Source: PLoS One. 2020 Feb 10;15(2):e0227911. doi: 10.1371/journal.pone.0227911 (PMC7010301; doi:10.1371/journal.pone.0227911)
Supplement: S3 File — (ZIP) [file pone.0227911.s003.zip › Transcripts/Patient 17 Male Age 50.docx]

**Patient -17 Age 50, HIV VL**

*Note: After interview was over, patient asked me not to share anything about his HIV positive status with his grandson who was waiting outside the interview room. When asked why, he said he has an elder brother who is well educated, and he doesn’t want him to find out about the illness. He fears if people found out about his seropositive status, they will say he is untouchable with dirty habits.*

1. Can you tell me how did you happen to come her? How did you come to know about [redacted]?

R. I went to [redacted]….. In [redacted], I came know that the treatment here is very good …..right?

I. Yes

R. So.. I came here…

After coming here, I met the doctor ……. Very good medicines were given to me … on good medicines. I am now healthy.. fine… I have no problem…. The only problem is that of age… I am a bit weak person … I wish some good medications are given to me … that something better but there is no disease………… I am very good….

1. There is no problem ?

R. Yes

I. And your.... what we call discharge… there was treatment for 30 days…. Right? When did it get over?

R. I don’t remember it… but its written in the papers.

I. How many months has it been approximately?

R. about (pouse)

Kartik, Aghan, poos, Magh, Fagun, Chait, Baisakh (Counting hindi calendar moths)… seven months have passed.

1. It had been 7 months?

R. Yes

I. Ok… tell me when did you just come to know about this disease?

R. Initially. I had high fever and didn’t eat food.

I. for how many days did it last?

R. About …… 2-3 months

I. You had fever for 2-3 months? Continuously?

R. Continuously

I. & you could not eat food?

R. No… I could not eat anything.

I. Ok…. Then?

R. But when I came to this hospital, my fever also was cured.. and bread (roti) too… 3-4… 3-4 bread

I. Could eat…

I. But after retwining, when you came to know about the disease.. when you came to know for the first time… The first thing that you had was fever.

R. Yes (interrupting)

I. And abdomen problems

R. Exactly..

I. What else did you feel?

R. I had weakness

I. Weakness..

R. Yes..

I could walk for some distance and then stop… I staggered & feel.

1. Hmm

R. Now.. everything is fine…

I. Everything … eating and everything else?

R. Everything … yes.. I can eat as much as I want

I. As you told how much? 3 months?

For 3 months you had it?

R. Yes .. 3 months

I. Why didn’t you visit a doctor then?

R. Oh my goodness.. money.. I am a poor man.. 5 katha land that was there.. I sold it.. my family sold it… then also the disease didn’t get cured … I am Harijan by cast you understand “Harijan”, right?

I. Yes.

R. Like…. You know Ambedkar right?

I. Right… Right.. I understood…. Understood

“Harijan”… yes

R. Yes…. “Harijan”

I. Yes.. Yes… Yes

R. I am a poor man… my children did not build there houses… got busy with my problems… the only thing is that the government should do something so as to give me some benefit.

I. Yes.

Ok.. you tell me one thing when you first had disease…. Whom did you consult first?

R. I went to [redacted]

I. [redacted]… who is he?

R. A very famous doctor in [redacted]… Then I went to [redacted].. to consult Dr. [redacted].

I. Both of them are private practitioners?

R. Yes… Private… No (corrects) government doctors…

I. Both are” “?

R. Yes… then I went to [redacted].

I. 1 minute.. Do the two doctors sit in a big government hospital or they have private clinic?

R. No… Private clinic… their fees is Rs. 400..

I. Ok..

R. Then … I went to [redacted].

In [redacted], the doctor, he´s either an MLA or an MP, that is written in the prescription .. he said that .. you have disease…

1. The two doctors about whom you said were good… what did they call you about the disease?

R. Medicines for fever were given but fever did not subside… and did not feel like to eat… ehn my family members said to eat something , I threw it…

(pause) when I came to this hospital, my life returned back.

1. Ok.. So you consulted two doctors, then you went to [redacted]… 4,5 (pouse) 7,8 doctors I consulted.. I then felt that I will surely die…. I thought that God will now take me back from this earth…
2. You are feeling this now?

R. Yes.

I. Why?

R. I mean I became like this…

I. You mean lecen & this? Weak?

R. I had no strength to walk from here to there?

I. To walk? To walk 5 foot?

R. Yes… Yes…

I came here .. my life returned back to me..

1. That’s good

R. I have no complaint … its good everything is fine.

I. why did it take you so along to go to a doctor how many days following the onset of disease, did you visit a doctor?

R. Earlier .. around 15 days passed… when I went there, photostate was done.. blood test was done … 10,000 Rs. Were spent in all that.

I. Yes its fever…

R. Yes…its typhoid (pause) saying so.. nobody told its kala-azar…. All my money was drained

I. But didn’t tell you?

R. didn’t tell

I. This is private?

R. Yes

Then we went there.

Í. Where?

R. [redacted]

I. [redacted], Government Hospital?

R. No…. That is also private

I. [redacted]is private?

R. Hmm.. name is there on prescription .

I. Is it a big hospital>

R. big hospital … He is M.L.A… He is “Yadav” [caste] he told that I had Kala-azar.

I. And?

R. and, he told this…

I. What?

R. What is the name? if (Pauses & thinks)

I. About the other disease?

R. Yes…

I. Do you know the name?

R. No…

I. Something like HIV?

R. Yes… Yes… Yes..

I. you came to know about Kala-azar in [redacted]?

R. Yes

I. Where did you know about HIV?

R. (Interrupting) that also he told…

I. He told?

R. Yes

I. Before that you did not know?

R. Nobody… nobody told me…

I. There did they tell you why it happen?

R. (pause)… I (pause) did not know anything.

I. Even the doctor (interrupted)

R. No.. No..

I. The, what did he tell?

R. He told me to go to [redacted]. I went to [redacted].. the doctor referred me here..

I. Ok..

R. I came here

I. In private .. that big hospital.. how was their behaviour with you?

R. it was very good..

I. it was very good.

R. Yes

I. and in [redacted]?

R. In [redacted] also.

I. and before that in [redacted]?

R. He is also good man. He also sent me to [redacted]. Should we refer you to [redacted]or would you like to take medicine from [redacted]? I said register me from [redacted], and then I started getting medicines from [redacted].

I. What did they tell you?

Why to come there? In [redacted]?

R. For the disease … disease will be cured..

R. The doctor…

I. Did you come yourself or were you brought here?

R. No… three persons brought me here…

My wife, my daughter & myself and no other person was sent.

1. So you came to know about both Kala-azar & HIV before coming here?

(pause)

Is that right?

R. Yes

I. You knew this before coming here, [redacted] (interrupted)

R. yes… he told

I. In [redacted]

R. In [redacted]

I. But that time they did not tell you why & how it happens?

R. No.

I. Where did you know about that, then?

R. here.. (stammers) itself

I. What did they tell you?

R. They told that if in young age some mistake is made…

Something happened… No sir…In my life… I have not done anything wrong

1. Hmm

R. Stealing... [*inaudible*]

I remember .. but I have not done anything wrong… (pause) yes, one man was there in village. He had a wound … it was out… I bandaged .. the blood that was coming out .. I bandaged

1. Ok

R. Only that I remember

I. & nothing else?

R. No

I. If I ask you, that when you first came to know about the disease, what was happening in your body?

R. complete peace… nothing is there in the world… look… all this went here (pointing to show the thin body).. but now its fine.. here.. I mean I became like this (expressed cean & thin body with hand)

I. Very thin it became?

R. Yes.. very much

I. desire to eat?

R. Neither had desire “…. Nor to speak…

I am speaking… its all the blessing of God and doctor…

1. What did you pray to God that time?

R. I prayed that God! Either take away my suffering or take me away from here … away from this world.

I sold my farm too…. My children were devastated… I am a very poor man (pause)

Sir, I amm………………..

1. Yes….

R. Whenever you send this, tell them that they are the God for me it they send something for me.

I. Yes… yes….

R. May my house be built………..

I. Live in hut? Do you understand hut?

I. Yes…..

R. Hut of straws….

And I say it correctly… (pause) that I feel bad about my situation… very hardly we get to eat…. Its not a lie….

1. Hmmm..hmm..

R. If you ask my name in my village…so this is the thing.

Its true I am a poor man

1. Was your sleep affected?

R. Yes… Like this I slept (showing) postures) … and sleeping supine was fine…

I. How is your house? Kuttcha or Pucca?

R. No it’s a hut…

I. What all dreams did you have before disease? & how did the disease affect those?

R. The effect of disease was that like I am going somewhere & someone hit me & drove me away (why pause).. I am going somewhere & again someone hit me & drove me away.. (Long pause)…

I am going in a river to swim someone lifted me up & threw…

1. Who did like this?

R. The God did… In dreams..

I. Ok.. you felt like this?

R. I felt like this…

I. You felt this in you body?

R. yes.. yes.. yes..

That somebody threw me away…

1. Then I thought .. said to my family that I will not die… Thrice have I gone.. like the seven sea crossing to the God.. my days are not yet over.. so I keep returning back.. I don’t have the disease.. Sir, may many thanks that I came here for my children.
2. Nothing is like this…

What you think you will be doing after going back?

R. Now I think … That my sons are already devastated due to me..

I. Why so! Why devastated?

R. much of the money is spent on me…

I. Ok.. how much money was spent for the treatment?

R. 3, 4 lakhs rupees have been spent.

I. 3, 4 Lakhs? (surprised)

R. Yes.. its not a false talk…

I have these many prescriptions (Pause)

I. Oh my God!

Everything was done in private?

R. Yes..

I. Why did you go to private? Did you know there is treatment in government?

R. No.. No..

I. you didn’t know?

R. No.. nobody told me.. but the doctor of [redacted] said….

I. Ok

R. Nobody told me (pause)

I. hmm..

R. wherever the record goes.. will this go somewhere?

I. No.. No.. nowhere … only me will listen .. our team

R. Ok.. team is there?

I. Yes.. yes..

R. That is what I am saying that either get a house built for me or give me some money.

I. Yes… yes..

R. ‘[redacted]” s my name… I am Harijan by caste

I. What threatened you most?

HIV or Kala-azar?

R. Kala-azar

I. Why?

R. Due to Kala-azar only, I had loss of appetite… I didn’t know anything called HIV.

I. Means… you did not hear?

R. Yes.. Yes..

I. Ok

R. But about Kala-azar, people said it causes progressive wasting and leads to death..

I. In you neighbourhood in village, do other people have kala-azar?

R. Yes..

I. What all is there?

R. He has.. didn’t I tell you that he has HIV? (Pause)

I. who?

R. He is shopkeeper..

I. who?

R. Shopkeeper.. his medicines are going on from here…

I. Called him and he too is fine …

I. Ok

R. Yes.. he is fine

I. of HIV… in future what do you think! What do you want from life?

R. From life, I want that may God lead me further ahead…. My health is now fine.

I. Do you know for HIV you will have to take medicines daily..? every month (interrupted)

R. Yes.. yes…

I. you know this?

R. Yes.. Yes.. one medicine I will have to take.

Whole life… life

But sir… weakness is still there.. .if I don’t eat anything for breakfast, [*inaudible*]

I cough a bit…

1. At present, it happens? .. slightly?

R. Yes

I. OK

R. Earlier, what work you did?

R. Earlier, did farming..

I. Now… you sold all your farm!

R. Yes

I. Then what will you do in future?

R. What shall I do? I sent my children to [redacted]…

They are working as labourers…

I. How old one you children?

R. Around .. (pauses.. one 17-18 years of age.. and one is young.. . he was studying but left studies.. He is also working as Labourer.. (Pause)

What to do?

I. Hmm

R. God put them on me…

Destroyed everything..

I. When you come to know about the disease. Did you tell you wife? (interrupted)

R. Yes.. Yes.. I only told my wife.

I. Does anybody else know about it?

R. Nobody knows..

I. only wife?

R. Only

I. Was she tested?

R. Yes..

I. She.. does she have?

R. Her medicines are going on…

I. Did you know this that time itself on you knew it earlier?

R. No.. that time itself

I. In [redacted]?

R. Yes..

I. Did you experience any change in your wife’s behaviour before and after knowing the disease?

R. I don’t even go..

I. Meaning? Where you don’t go?

R. Means (pause) we don’t sleep together anymore..

I. Ok.. tell me something more about this..

R. The strength that I had earlier ..

I don’t have now

I. Ok

R. I say to God.. it hurts..

That o God! My house is destroyed.. I now live in sorrow.

R. Yes.. my house.. (pauses)

I earned money for my children.. everything was spent on my disease.

I. Yes

R. I had saved money .. everything was spent.. I say the right thing to you.. no false talks..

I. Did you notice any change in behaviour of your wife?

R. No.

I. When you told her for the first time, what did she feel? What did she tell you?

R. 10-15 days.. she told that you must have done something wrong.. she had doubt.. that .. you must have done something wrong, only then you got this disease.

I say-no.

He had the cut too had a wound … look..

Isn’t it cut? Here?

I. yes

R. I too had a cut and he also had a cut.. when he had a cut. I bandaged…

The wounds came in contact.

I. Did she agree with it?

R. Yes.. she agreed..

I. So, now, any problem?

R. No.. no..

I. Ok.. then.. would you like to say something else! About the disease!

R. about the disease I would my the God bless…

My the disease come in this hospital and end..

I. Ok… thank you very much…
