# Supplementary material for: Quality of life perceptions amongst patients co-infected with Visceral Leishmaniasis and HIV: A qualitative study from Bihar, India
Source: PLoS One. 2020 Feb 10;15(2):e0227911. doi: 10.1371/journal.pone.0227911 (PMC7010301; doi:10.1371/journal.pone.0227911)
Supplement: S3 File — (ZIP) [file pone.0227911.s003.zip › Transcripts/Patient 18 Male Age 60.docx]

**Patient 18 VL HIV Age 60**

I: Who all are there at your house?

R: Right now, currently?

I: Yes. Who all live with you right now, where you are living in the village currently? Who all are there living with you right now?

R: 3 sons ...1 wife, 1…. daughter-in-law (*explains the local word for daughter-in-law*)

I: Yes, the daughter-in-law. Does she have any kids too? I mean do you have any grandchildren?

R: No. No… Grandson yes… One from the first marriage now expired daughter-in-law, a granddaughter is there.

I: Ok... And… the other sons haven’t gotten married yet?

R: 2 marriages had happened... 1 has been kept outside [one of the wives doesn’t live with them because of legal issues]

I: Ok. The one whose wife had expired, has he married again?

R: Yes he has married.

I: His kid is with you?

R: Yes... One son is the…a year old.

I: Okay so... what work do you do?

R: I am a barber…a barber…I own a saloon, and then I do some farming here and there also.

I: So you have your own farmland too?

R: No-no. Others’ farmland by borrowing.

I: Okay... Tell me one thing about your disease…before which time were you completely all right?

R: Yes I was fine earlier.

I: Yes, how many days ago? 1 year ago...2 years ago...3 years ago?

R: Meaning…take it that around 2-4 months ago I was fine.

I: Okay….

R: Yes?

I: Yes?

R: 2-4 months ago I was fine.

I: 4 months ago?

R: Yes... and I used to sleep like…I used to look after cattle and sleep on the floor out in the open so maybe it’s possible that from there while handling dirt, cow dung, garbage, etc., I got infected.

I: What was your difficulty in the beginning?

R: In the beginning I got fever. *[Says the English word fever*]

I: Fever [In Hindi to confirm that he actually means fever]

R: Fever [In Hindi]. [*Takes a breath*] … after getting the fever I consulted [redacted], MBBS at [redacted] hospital.

I: [redacted] is a private consultant?

R: No… he is a government consultant.

I: Government?

R: Yes… He is a consultant at [redacted] hospital.

I: He has a big hospital there too right?

R: Yes...

I: In [redacted]?

R: Yes, he has a hospital in [redacted]. So I had consulted him and kept going to him for around…1 month approximately… for malaria etc. and other diseases I got checked. After the malaria test, I was told that… I should get the test for Kala-Azar done. On doing the Kala-Azar [*stammers somewhat while saying Azar*] Kala-Azar was detected and he told me to go to [redacted] hospital. [redacted], a relative told me... come to [redacted].. On coming to [redacted], I was told that here the facilities are not sufficient... ummm…that there is HY [meant HIV] found in me, so I should go to [redacted].

I: What has been found in you?

R: Ummm... this… one time I had a blood test... there was HY written…

I: HY? ...*HIV*?

R: HIV.

I: So who told you about this HIV? In [redacted]?

R: No. In [redacted].

I: [redacted].

R: In [redacted]. So, after being informed in [redacted], I came to [redacted]to [redacted] in here.. So these people asked that other whether I got it from a wife or someone else or how it happened what happened.. To tell them.. So I replied that I haven’t done anything wrong. I till today have never done anything wrong to anybody. [Denies having multiple sexual partners] I have a wife. Maybe something wrong might have happened…right? ..So.. Regarding this treatment has been started.. so.. Kala-Azar treatment is going on in [redacted]. After starting treatment, fever is fine. After I left here, there has been no fever. They told me that I should come on the 24^th^ and get a checkup done so I came on the 24^th^. This is what has happened.

I: Okay tell me one thing… When you were informed there in [redacted] hospital that you have this disease HIV, or when first in [redacted] you were informed that you had Kala-Azar, how did you feel in the beginning after hearing that you had the disease?

R: After being informed about Kala-Azar, they said to go here ...that it will go away

I: You knew about this [kala-azar]? What kind of a disease it is or whether there is a cure or not?

R: Yes I had heard the name earlier too and fever was occurring.. fever was occurring and I was undergoing treatment for the fever. HIV was told in [redacted].

I: Did you know about HIV beforehand? Had you even heard about the name?

R: No. Hadn’t ever heard the name.

I: Hadn’t heard even the name?

R: No. hadn’t heard.

I: Okay. So .. after that later, when you were informed you had HIV, were you informed about HIV? Like what kind of a disease it is, how does it spread?

R: No.

I: No. Was your wife etc [referring to sexual partners in general] called there for tests?

R: No, my wife wasn’t called up for any tests.

I: You mean they weren’t called to the [redacted] Hospital?

R: No. They weren’t called.

I: What did they do there then?

R: From there I came to [redacted].

I: So they sent you to [redacted] from there.

R: Yes. Chabi ji called me via phone, a boy accompanied me to [redacted].

I: Okay. Now that you know about the disease, who all in your house have you informed about your disease?

R: Nobody else has the disease. Everyone is fine.

I: No about you. What have you told them about yourself?

R: I have only told the family about Kala-Azar.

I: Oh, only about Kala-Azar. Not about the HIV.

R: No, haven’t talked about HIV.

I: What did you feel that you only informed them about Kala-Azar?
R: Even though the kid [who accompanied him to [redacted]] he hasn’t talked to anyone regarding this ummm[ in a somewhat low tone]

I: Do you feel something that you shouldn’t talk about HIV, or that others will think something about you, do you feel anything like this so you didn’t.. I mean why didn’t you tell anybody? Like you told them about Kala-Azar, but umm … Regarding this [HIV] you didn’t tell anybody.

R: No I didn’t tell anybody.

I: Okay. So.. In an around you in the neighbourhood are there any people who have this kind of an illness in your village, that is [redacted].

R: Yes, [redacted].

I: So in [redacted], like in your neighbourhood where people usually sit and talk, have you had any conversation regarding someone else also having [HIV]?

R: No right now I don’t know of anybody, but I’ll find out if someone has it.

I: Oh so currently nobody else you know has it.

R: The people in the neighbourhood believe that I had Kala-Azar and I underwent treatment so I should be fine now. I mean umm.. if anything of this sort [referring to HIV] they have my contact details so they’ll call me.

I: Any other complaint you had that time other than fever?

R: No, no other complaints.

I: Any night sweats?

R: Yes, I used to sweat a lot in the night.

I: Fever..

R: Yes?

I: Was present all the time?

R: No. Sometimes. Meaning, in the evening-in the night my clothes used to get wet due to the sweat and by the morning they would dry.. and around 4-5 AM I would have to go to urinate.

I: Earlier how much did you urinate? Meaning earlier wanting to urinate around 4-5 AM was normal to you or this was something different?

R: No after coming here.. umm I mean it was ok there.. meaning when fever started from then urination I thought treatment was going on anyway, I would become all right, but [redacted] couldn’t control it.

I: Weight?

R: Meaning?

I: Did you lose..

R: My weight had decreased [fumbles a bit]

I: Decreased. Did you measure your weight? What it was in the beginning and what it was later?

R: Yes. First it was around 60.

I: Okay, Then? When you went to [redacted] the doctor you went to, then?

R: No, I don’t remember that much.

I: You don’t remember. But your clothes you wore, they fit you properly or they felt loose?

R: It was winter season so I wore [multiple] clothes.

I: Oh, winter season. Any other complaints like cough?

R: No cough.

I: Any itching in the body?

R: Yes itching is there right now.

I: It’s there right now, but what about earlier?

R: No, it wasn’t there earlier.

I: How was your appetite?

R: It was less in the beginning but became normal later.

I: So after treatment your appetite has become normal.

R: Yes.

I: Okay. So.. in your family, only your eldest son knows about this [HIV].

R: yes. umm

I: About HIV. Kala Azar you have informed. But you haven’t informed about this [HIV]

R: Yes.

I: So as per you, to live a quality life, everyone has their own opinions… What are the things needed to live a quality life?

R: Good medicines are needed.

I: okay, good medicines are needed, very good.

R: other than that there should be focus on diet, cleanliness, there should be cleanliness in the surroundings.

I: Very good.

R: We need green vegetables, a proper diet, shelter should be proper.

I: So you say shelter, diet, surroundings and a 4^th^ thing you say.. medicines are required. Other than all these anything else you think that is required? All these things… how will they come? … Anything else you think is essential to lead a quality life?
R: essential.. well you see the son earns but there is always some tension whether he will send money or not

I: So he doesn’t send money?

R: No he does, but everyone is buy with their own families.. there is always tension

I: During the illness did you ever feel anything like your life is over, there is no point of living anymore?

R: Yes, yes. When I first came to [redacted], I felt very weak and I was feeling that I wont survive anymore.

I: You felt you wont survive anymore?

R: Yes I felt I wont survive.

I: why did you feel so?

R: Due to the weakness also I had started losing hope because first [redacted] said he couldn’t treat me anymore, then in [redacted] they told me they didn’t have any treatment [for HIV] but the, I thank this hospital because I am fine now.

I: So at that time did you feel anything like there is no point of living anymore?

R: Yes at that time.

I: Okay. And about your family like your wife, have you gotten her tested?

R: No I haven’t gotten her tested yet.

I: You haven’t gotten her tested yet? Like in [REDACTED] you said you were called, she wasn’t called?

R: No She is weak.. I mean she has high diabetes and she gets fever from time to time and also gets gastric problems from time to time, she is getting treated by [redacted].

I: She hasn’t had tests yet. Not even in the [redacted] Hospital.

R: No.

I: okay, that’s okay and you haven’t told her about yourself?

R: No

I: now that you have this disease how do you feel? Like right now you are undergoing treatment but before treatment the work you used to do, how well were you able to do it?

R: I used to work a lot earlier but now I can’t work that much. I feel weak, but I believe that If I eat and drink well and with treatment going on, I’ll become fine. That’s the thing.

I: So when you had this disease and you were consulting [redacted] during those 1-2 months, were you able to do your work that time?

R: Yes

I: You were able to? What could you do that time?
R: Shaving, looking after the animals.

I: How many pets do you have?

R: 2

I: What all?

R: 1 cow and her kid

I: She gives milk?

R: Yes more-or-less

I: Do you sell the milk too?
R: No our own people drink it.

I: Okay, so milking the cow and taking care of it etc. all these jobs, who does it?

R: The smaller kid does it.

I: Okay and sowing the farm and harvesting etc... You were able to do all this?

R: Earlier I was able to.

I: No, when you were ill?

R: No not that time. I sowed the crop and went and on coming here I fell ill so during the earlier time I was all well when doing the farm work.

I: And when you fell ill then?

R: I left it.

I: So that time you couldn’t do any work.

R: No.

I: So what do you think about the treatment that you are receiving here? Do you think there can be any improvement in the facilities you are receiving?

R: Right now, I don’t know much regarding this topic but I feel there should be an improvement.

I: Improvement in what?

R: With medicines or…

I: That is improvement in you. Like when you went to [REDACTED] to take treatment, how was the staff behaviour towards you, were they talking properly to you?

R: Yes it was good.

I: And the medicines provided there, are you satisfied with them?

R: Yes.

I: Any other thing which you think if it were present it would have been better?

R: I would like institution to move forward.

I: Tell me this do you fell when you go back…. After taking treatment when you went back to your village, what did you tell the people there?

R: People asked me if I was fine and I said I was. They asked me if I was still getting fever and I said I wasn’t. That was the talk.

I: After HIV did you try to find out what kind of a disease it is, how it spreads, or were you informed anything [like this] here in [redacted]?

R: Yes, they asked me if I had done anything of wrong behaviour [multiple sexual partners?] to anybody. They asked me this. I said that I had done no such thing umm… aa..Umm… [Takes a pause]... I had one [sexual] encounter before marriage. What should I tell you? Why should I hide this from you? It had happened then. Never happened after that.

I: So when you went back to your village, what did you tell the people? That what disease had you gotten?

R: yes... only Kala-Azar.

I: Only Kala-Azar...

R: Yes.

I: You don’t feel that they [village folk] might be talking something about you [regarding HIV]

R: No. Nobody talks to me about any such things.

I: Why? Don’t you have any friends in your village?

R: I do have friends, but I don’t talk to them about such topics.

I: You mean you talk about other topics?

R: Yes

I: So not much more regarding your health.

R: Yes.

I: Okay, what was your financial situation…? I mean did you receive financial aid from the Government or anywhere else?

R: No, no aid was given anywhere.

I: At [redacted] hospital?

R: No.

I: At [redacted]?

R: I had been given Rs. 600 to travel to [redacted] with my son.

I: To come to [redacted] from [redacted]?

R: No. When I was going from here, I think on the 10^th^….

I: That money was given by who?

R: At [redacted]…

I: It was given at [redacted]?

R: Yes, at [redacted] they gave me a fare of Rs. 600 to travel.

I: Okay, this didn’t happen at [redacted] or [redacted]?

R: No.

I: Okay, who goes to get the medicines, like every month do you go to get medicines?

R: I mean um... medicines of 1 month I had gotten here at [redacted].

I: Okay.

R: I had received a container, so I was given a container and I was told that umm... from...here...I was told to get registered in [redacted]. What should I say…transfer…like I have a difficulty in coming to [redacted]- then where will you get it done? Get it done in [redacted]. Then, I got it transferred to [redacted]…that’s the thing...

I: So after this have you gone there [[redacted]] to get medicines?

R: No I haven’t gone.

I: You haven’t gone yet.

R: No.

I: By the way how much money is spent to get there?

R: Where?

I: [redacted].

R: To get to [redacted], say from [redacted] to [redacted], approximately Rs. 100 you can assume.

I: 100. Currently what work are you doing? When you had gone back, then?

R: No work.

I: How are you making ends meet at home?

R: Household expenses are being taken care of by the children, so we are managing somehow, what can we do.

I: What do you think regarding the upcoming future? What things do you want to do in the future... anything you had earlier thought of... like earlier when you didn’t have the disease that time what all things had you thought of that you wanted to do? Comfortably think about the time before your illness and tell me.

R: Earlier I had thought I would construct a house properly... and get my children educated... I mean my children are educated now... meaning BA etc they have done... and that I’m now 50-60yo what will I be able to now anyways… now he [sons?] will only do everything... all this broke my confidence.

I: No about the time when you were healthy... that time you had lost faith? When your health was completely alright.

R: That time I had thought about constructing my house.

I: Constructing your house. What kind of a house do you have right now? Is it kuccha or is it cemented?

R: It is not a brick-and-cement house, but it is sealed with mud.

I: I see it is sealed with mud. All your children are married?

R: No. 2 are married and 2 are unmarried.

I: The 2 [married] are boys... or girls?

R: Boys.

I: Both are boys. So had you thought about their marriage at that time [before illness]? That you have to get them married?

R: Yes I had to get them married so I had thought that it will happen… what has to happen will happen... now what can one do. If one himself is not well then what can be done?

I: Now that you are saying that after taking medications you are feeling a little better, are you eating food properly?

R: Yes I am eating.

I: So now what all do you think. About the things you had thought of earlier, what is your opinion on those things?

R: That I again gather strength, that I eat well and work. That is the thing. I am a labour-class person, my body should be fine... I mean somehow I should be able to run the family decently... this is what I think.

I: Now do you think you can construct your house?

R: Why shouldn’t I be able to?

I: You can do it right?

R: Yes.

I: I mean the work you had left earlier, do you now think you can complete it?

R: Why not?

I: Okay. So for doing that what do you think now?

R: First I have to get well, then only can I do something.

I: … [Tries to say something]

R: Once I become healthy then only can I do something or get something done. That is the thing.

I: The medicines you are taking, due to them any difficulties you are having? Any complains due to the medicines?

R: No. No complaints regarding the medicines. Umm. Its okay... there are benefits.

I: So there are benefits. You are feeling better than before?

R: Yes.

I: Okay. And... You surrounding areas... are you satisfied with them?

R: Yes. Yes.

I: Like you had mentioned earlier that surroundings should be clean... your surroundings?

R: Yes it stays clean and I keep it clean.

I: You keep it clean... because you like that. How safe do you feel? [Tries to explain ‘safe’]

R: I am staying at home, I will want it to remain clean….

I: Not clean, safe. By safe I mean like one has a fear for anything... do you feel like this... do you fear anything?

R: No I don’t fear anything anything.

I: Before the illness?

R: No.

I: Neither they nor now. Okay. Right now how much... um... how much are you happy living your life?

R: Yes till now I am living. Thanks to you all I am still able to live happily. That’s the thing.

I: So now after going back, what work do you do from morning to evening?

R: From morning I go to work... I mean I go to the market... around 1.5km away via a motorcycle

I: You are able to drive the motorcycle?

R: No. No. I don’t drive it. Someone else takes me. People say don’t stand around.

I: So where do you go in the market? To cut hair [to work as a barber]?

R: No I go to a shop, I go there only, it’s a shop selling vegetables.

I: You go with vegetables. Is it your own shop?

R: No it’s in the market.

I: Okay. What do you do after that?

R: What will I do, I stay at home.

I: Any similar work, any other work you do?

R: No other work.

I: Are you able to sleep well?

R: My sleep at night has decreased.

I: Earlier? Earlier before the illness also it was the same or this has occurred now?

R: … earlier it was some less

I: So how was your sleep before the illness?

R: Before... it was okay. I mean I used to wake up at 4AM and go for a walk. I used to do everything.

I: Okay. For how many hours approximately did you used to sleep in the night?

R: Say around... umm…. Around 10 hours... around 10-11 [stammers] hours.

I: 10-11 hours you used to sleep?

R: Yes.

I: And after this illness occurred?

R: Around the same time.

I: So you didn’t face any difficulty in sleeping? Just now you were saying that you had decreased sleep.

R: Yes it is decreased maybe because I sleep in the morning so in the night I sleep less.

I: So because you sleep in the morning your sleep time at night is decreased. So otherwise if you haven’t slept in the morning you don’t have any as such problems in sleeping? In total you are sleeping the same amount of time?

R: Yes

I: Okay. For the things that you require daily, do you have enough money to buy these items?

R: Now what can I do. There is a requirement but funds are scarce so there is a compromise. Like instead of Rs. 100 of fruits I might my fruits worth Rs. 50. That is how the family sustains.

I: The people living with you like the little kid, the wife, their expenses are taken care by you or your son gives you money to look after them?

R: Till now it’s been taken care of by me only, my son sometimes gives money... sometimes he doesn’t... but now… there is some earning, but we somehow compromise and make our ends meet. Some grains from the field…and then somehow make ends meet.

I: So you have to compromise.

R: Yes.

I: You want to say something more? Right now do you have any more expectations form life? How do you plan to live the rest of your life?

R: Now what more will I live? I have accepted that after 60 I will start getting weaker. And... Now what more will I live... If my maintenance is good, I will live, if my maintenance decreases, I will die. Isn’t this what’s going to happen?

I: What will become weaker?

R: Like in old age, isn’t there weakness?

I: But don’t some people live till the age of 80-85 too? You are just 60 years old right now.

R: It happens it all depends on the maintenance.

I: Depends on what? Can you repeat that word once again? Could you say what you were saying once again?

R: Assume I am 60 years old. It’s possible in some years after 80 I die due to decrease of maintenance, my diet etc.

I: Oh, maintenance, so you mean how well you are taking care of your body.

R: Yes, yes. So you see I am already 60, I’ll live for 10-20 years more at the maximum. This is what it is, what else I will think. My children are old enough, they will eat, earn, and do whatever they want to. This is what it is.

I: [long pause] anything else you would like to say?

R: What else can I say, I have told you everything, what more can I say, everything- my grief my happiness – I have told you everything.

I: So right now you are happy.

R: Yes, I am happy with the treatment I am receiving... I am happy with the treatment…and as far as diet is concerned, there is a little problem because sometimes my sons send money, sometimes they don’t but [life] goes on… I think if the body is well and it receives a proper diet it will be maintained.

I: Whenever you are coming to a hospital, for treatment, what all things do you look for? What are the things that you think are essential for a hospital? What all facilities should be available?

R: There should be medicines [available] and….. What to say [clears throat] medicine is required... what else is required... medicine is required on time, proper treatment is required, the body should be healthy... this is what is needed... if there is any deficit... I am telling only….

I: Like for your HIV, for it you have to take medicines everyday..... You have to visit every month… do you have any difficulties?

R: Difficulties...[redacted] is now close by. I was given in writing that I should submit this [document] and I shall receive medicines every month in [redacted].

I: Any problems in coming to [redacted] and procuring your medicines?

R: If I don’t come someone else will come and take it.

I: Who will you send because you haven’t told anyone about your condition [HIV]…? If you had told….

R: They will think it is medication for Kala-Azar

[Giggles]

I: Yes you can say this. Anything else you want... [Other interviewer starts speaking]… What if you come to know about someone else getting HIV…. What will you think about that person?

R: If that person tells me then only will I get to know

I: Suppose someone tells you...

R: Say someone after tests is sent to [redacted] and gets to know of it, then only will he be able to tell me. [redacted] hospital is a small hospital, there the person won’t get to know. Only if the person is sent to [redacted] or [redacted] will he get to know.

I: We want to know why you think one shouldn’t tell others about HIV.

R: Because of gossip.

I: So you think others will gossip about you?

R: Yes, what will others say, what will the children say, what will the other family members say because this has happened to me because I have done something wrong so people will talk about it…

I: So this is why you think one shouldn’t tell others about having HIV.

R: Yes.

I: When you consulted [redacted] in [redacted], did you have to spend money there?

R: Yes, it cost me around Rs. 1600.

I: But you said that it was a Govt. Hospital

R: He sent me to the Govt. Hospital later.

I: Later? Then where was [redacted] from? Wasn’t he a doctor in that hospital itself?

R: He is from [redacted].

I: Okay. So that cost Rs. 1600. Other than that any other expenses?

R: Travelling to [redacted] and [redacted].

I: So travel expenses. You didn’t have to spend money on anything else?

R: No.

I: Okay.
