# Supplementary material for: Quality of life perceptions amongst patients co-infected with Visceral Leishmaniasis and HIV: A qualitative study from Bihar, India
Source: PLoS One. 2020 Feb 10;15(2):e0227911. doi: 10.1371/journal.pone.0227911 (PMC7010301; doi:10.1371/journal.pone.0227911)
Supplement: S3 File — (ZIP) [file pone.0227911.s003.zip › Transcripts/Patient 19 Male Age 23.docx]

**Patient-19, Age 23, HIV VL**

I - Tell something about you?

R - About me. I do this. What else?

I - From where are you?

R - From [redacted] City

I - Since when have you been living there?

R - There, since childhood.

I - How many members are there in family?

R - 3-4, 3-4 Brothers, Father, Mother, Brother’s wife, Little children, Brother’s children & my family.

I - Your family?

R - Wife

I - Okay, You are married. Do you have child also?

R - No. I had child but he died.

I - Okay, Died. After birth?

R - After birth. One month after birth.

I - After one month. Why so?

R - It happened that his health deteriorated, He got fever. Then he died while under treatment.

I - Could it not be found out from which disease did he die?

R - No sir. Could not be fount out

I - Now, You do not have nay child?

R - No

I - So all persons, as you told live together in house?

R - Yes Sir

I - And what work do you do?

R - I drive auto.

I - Okay, since when are you driving?

R - 3-4, since 4 years

I - Okay, So you have to do it daily. I mean per day is it not that someday you drive or someday not?

R - Yes, it is like that. Someday I drive, someday not. If I am able to drive , then I do , if not able to drive then I do not.

I - So, you have gotten a regular auto.

R - Yes sir, on fare

I - Okay, Tell from beginning, What, how did you get?

R - HIV?

I - Yes, whatever disease it is. Tell from beginning about that.

R - Got it because of doing wrong deeds.

I - Mean? Because of doing wrong deeds?

R - This wrong. I did, so I got it. If anyone was in my surroundings, they also got it.

2^nd^ I - When, when did it happen? When did you come to know that you got it?

R - I got fever & body aches. After I got fever, I went for treatment. Went everywhere. When I went to private, I was not diagnosed with any disease. I was asked to go to [redacted] Hospital. So I went there & I was diagnosed with it.

I - [redacted]? Where?

R - [redacted]

I - Where?

R - [redacted]

I - [redacted], where is it?

R - It is where I was admitted for Kala-azar.

2^nd^ I - [redacted]

I - Why did you go to private first?

R - I went to private for treatment of fever.

I - So you went to private instead of government? Any reason?

R - There was no reason sir

2^nd^ I - So you knew about government?

R - Yes sir

I - Also knew about private?

R - I was told that go, he is a good doctor. Go to him. You will get cured. So my father took me.

-

2^nd^ I - So how much did your spend in Private? How much?

R - Rs. 20000/- were spent

2^nd^ I - After this, After that you came to government. And in government where did you go first?

- I mean before [redacted]?

R - There´s a doctor in [redacted].

2^nd^ I - A doctor in [redacted]?

R - Yes

I - You are talking about private?

R - Yes

I - After that. After that when?

R - After that, my…my…When I was not able to beget child, went to [redacted]. There was Dr. [redacted], I visited her that I am not able to beget child.

R - So I went there, I was diagnosed with HIV there. After checking with bits. I was diagnosed with HIV [patient had gone for treatment of infertility, and they were tested for HIV]

I - So, you came to knew about HIV at that time? And where the treatment was started?

R - MR

I - So it was told there.

R - I was asked “Go there, you will be treated there stay there”. So when come to MR, medications were started.

2^nd^ I - When did it happen? When did you come to [redacted] for first time?

R - 1.5 years has passed.

I - 1.5 years passed. After you came 1^st^ time.

R - Yes

I - What did you come to know first about?

- Which disease did you have?

R - I come to know that, I was not able to beget child. So went for treatment for this. So treatment, when treatment was started, Diagnosed with HIV. I got frightened totally. If I got this disease, how will I get child? So doctor said “You will not get treatment, you will not be able to beget child go to [redacted]. You will be treated there”.

I - At that time were you aware of Kala-Azar?

R - About Kala-Azar? Sir I got fever and I had loss of appetite.

I - Okay

R - When I had fever, loss of appetite, the sir (Doctor) from where I used to get medication for HIV said, Go, Go to [*inaudible*]… And get treatment for Kala-Azar. I got tested for Kala-Azar or by sending someone.

R - After being tested, they said get admitted there. So I got admitted.

I - [redacted]?

R - Yes

I - So first you came to know about HIV? Then you came to know about a Kala Azar in [redacted].

- Okay. So how were you feeling when you came to know about this disease?

R - I was feeling weak.

I - No, I mean, how were you feeling mentally?

R - Mentally I was feeling that I have destroyed my life.

2^nd^ I - Why so? Why destroyed?

R - I have got disease, Now I will not be able to beget children. If child will be born, he may also get disease.

2^nd^ I - So are you talking about HIV or about Kala-Azar?

R - About Kala-Azar

2^nd^ I - So you are saying that if child will be born he will also have Kala-Azar?

R - Yes

2^nd^ I - Okay

R - That made me lose hope

I - So………..So, have you told this thing to your family members?

R - Yes Sir, Whole family knows

2^nd^ I - Okay

I - What, about both diseases? Or one disease?

R - About both diseases

I - You told at that time?

R - Yes sir

2^nd^ I - When you told for first time about HIV to your wife?

R - Yes I told her that I have got HIV

2^nd^ I - What did she say? What did she say first time?

R - She said if you have got it, then get treatment

2^nd^ I - Okay

R - What can be done now?

I - Does she know how does it happen?

R - Yes

I - Did she know at that time?

R - Yes sir

2^nd^ I - So after that, did you notice any change in her behaviour?

R - No change

2^nd^ I - No change, How was your relation with her after that?

R - No, After Kala-Azar, HIV has been diagnosed, not after that.

2^nd^ I - No? Means?

R - I do not have sexual intercourse with her.

2^nd^ I - No relation? No relation?

R - No

2^nd^ I - So you do not talk to each other?

R - We talk. I do not stay with her (Do not have intercourse with her)

-

R - We talk

I - So, is there any change from her side? In behaviour?

R - No, we live normally. What else change is needed? All remains happy. It is a disease. If there is a disease, everbody should live happily. I do not keep children with me. I keep them away. Only it is what else?

I - So you take these precautions from your side? Anything like this from family side?

R - There is no problem. Everyone remains normal. We eat-drink happily

I - What do you know about this disease?

R - Everyone knows about this disease.

I - No, What do you understand that what happens with this disease, why happens, how does it happen?

R - That. We get it after doing wrong. And after getting this disease life is destroyed.

I - So, since when were you told about disease? How many years back?

R - 2 years have passed sir

I - 2 years, so during these 2 years what have you suffered from? How was it before? And how it is now? In these 2 years?

R - Sir earlier, you know?, 1 year or 1.5 years back I started feeling weak, So I came to know about Kala-Azar that I have Kala-Azar. So I went to [redacted] and got admitted. There food/water…Medications were started. Investigations were done. Then I got same energy as I had earlier. Again I got same energy as it was earlier.

2^nd^ I - And mentally? Did you feel any change?

R - No sir

2^nd^ I - Before treatment? & After treatment?

R - No change

2^nd^ I - No change. So you feel same as you felt before?

R - Yes sir

2^nd^ I - Before means before disease?

R - No sir, same as before disease.

I - So when you came to know about disease that you have got this disease. How did you try to come out? How were, you feeling mentally? What were you doing to come out of that?

R - For coming out? Came to doctors so that somehow it gets cured. What else would I do sir? A person will go anywhere for his life. If you give this, he’ll eat it, if you give that, he’ll eat it.

-If it would have been curable, only then I would have been cured.

I - So did you tell your neighbours about disease?

R - Yes sir, They knew.

I - Neighbours I mean. Who live near your house?

R - Yes Sir, All know

I - Everyone know. So had there been change in them?

R - No Sir everyone sits beside me. They talk to me, everyone comes to me. There is no problem.

2^nd^ I - So your told them yourself?

R - Yes

I - Does anyone says about this disease?

- This disease can cause this or not?

R - No Sir, None thinks it is contagious.

I - No one?

R - No sir

I - So tell this that for leading a good life what are things needed?

R - For leading a good life, for living normally eating-drinking, any work with happiness. What else? (खाना – पीना)

I - So this was that, what you need?

- How do you want to feel mentally?

- I mean normally, for good life?

R - For living good life sir, when a person has illness, they do not have the same strength as before. When I had not disease, I used to work for 10 hours, drived auto. Now I cannot work for 10 hours. I can do only for 4-5 hours. I gets tired. I feel from inside that I will not be able to do more. I feel like that I have become weak. And I do not have desire to do work. I like to sit or rest only, wherever I get.

I - Apart from this. What else do you feel?

R - I feel apart from this that If I get another work. I will do that easily. Better than auto, if there is any work where I can sit down, which I can do well.

I - So how much did you earn before disease & after disease?

R - I am not able to earn for last 3-4 months. My family members look after me. Before that I earned sir.

I - …………..How should be the family life?

R - Family life should be that. Every one live happily with love to each other. What else?

I - And about neighbourhood? The people, what do you think about them?

R - All is fine sir. Every one talk to me. No one feels that I have disease & they will not sit beside. Everyone holds me, plays with me, and associates with me.

I - So you have come to [redacted]. What do you think about the treatment you are getting?

R - There is good facility sir

I - What is good there?

R - Food , Water, Medications, Neat & Clean society.

I - And staff working there?

R - Very good, they are very good. The sisters there talk very nicely, speak nicely. Everybody there talks with love.

I - Doctors there?

R - Yes

2^nd^ I - What is the condition of government hospital if you for treatment there?

R - There is [redacted]. There is very problem. If they come to know about HIV, they even do not like to touch you, eve doctors.

2^nd^ I - Anything else? And this [redacted]? What differences did you notice between [redacted] and [redacted]? Difference in behaviour of staff?

R - The staff in [redacted] sir. They talk with very love as if I belong to their family. Talking & whatever problem is there, is solved. And in [redacted] they say – stay away, go away- you have HIV- if there is a disease in your chest or whole body, they will treat you. If you say it´s HIV, they won´t treat you.

I - Okay, so anything in [redacted], do you want to be changed? That your treatment should be in different way, So that it becomes more better?

R - Yes Sir, I got problem- it is a convenience that I can get treated in [redacted].

I - Anything did you feel wrong in [redacted]?

R - Felt nothing wrong sir

2^nd^ I - Could we do it better?

R - If it is done more better, then it will be more good.

2^nd^ I - Like? What can be done?

R - Like Kala-Azar is treated, treatment of other diseases if I get. Like I got injured by hand.

I - What changes do you want in [redacted]?

I - In these hospitals?

R - The change should be that doctor talk nicely, the doctor there is like, stay away, stay away! Say these words.

I - And what do you want in life?

- What do you want to do?

R - I want in life that I beget children and I want nothing else.

I - Anything else?

R - For living, I live happily

I - Do you have our own house?

R - No, I have my own house, Sir [*inaudible*]

I - So it is yours

R - Yes sir

I - I mean your father’s?

R - Yes Father’s

I - Do you think that after getting this disease whatever you want to do in life will be affected?

R - Will be affected. Whatever I want to think. I will not be able to do that. Because I have go disease. Sometimes I suffer this, sometimes suffer that. This is a such disease I suffer something almost all time.

I - What is that you have not been able to do because of this?

R - Because of that, If I have to bring some medications, I am not able to collect money for that. So I am not able to bring medications.

I - So because of this disease are you in trouble for money?

R - Yes sir

I - How?

R - For example when I go to doctor for HIV medications, then doctors will write some medications from outside. I buy that outside medications. Costs about 3000 or 3500-4000 rupees. Sometimes it is 2000 rupees, then it is okay.

-

I - What was the time interval between ‘You got disease’ and ‘you visited a doctor’?

R - Could not get you sir?

I - I mean when you feel that you have disease, cold, fever and when you visited doctor yourself. So what was time period?

R - At least 1 month

I - 1 month

R - It took one month. Private doctor could not relieve. He gave injections, 14 injections. But it did not relieve fever.

I - So were you told there any anything what have you got?

R - No, So I asked them to have a blood test. Blood test was done but there was no HIV. He said you do not have HIV.

I - What were you feeling mentally?

R - Mentally I was feeling that my life has been destroyed. If I die now, then it will be better. I will not bother anyone.

I - Why were you feeling so?

R - I was feeling so because such disease. The people there said that he will not live now. Many people there used to say he will not live. That is in my hand- whether I will be alive or not.

2^nd^ I - About these two. Did they say this about both Kala-Azar & HIV or?

R - About HIV sir

2^nd^ I - And about Kala-Azar?

R - Did not say so sir.

2^nd^ I - What did you think about that? What did you think about that disease?

R - About that disease. There so many people got Kala-Azar, they got cured by injections. But for HIV-Kala Azar, there is a separate treatment.

- People feel disgusting about HIV but not about Kala-Azar.

2^nd^ I - Tell some more about it. I mean how do people behave? What do they think?

R - Behaviour is that, you have disease, Do not sit beside us. Stay away. All this what else?

I - Who are the people who do this?

R - Neighbourhood. They taunt. They say that it is a disease. If I spit, they say that disease spreads by spitting.

I - So till now, your neighbourhood people do that.

R - No sir, they do not do now.

I - Then, when did they do?

R - In beginning

I - So, how did it change?

R - It changed because [*inaudible*]. Our [redacted] sir. Our neighbourhood people said. [redacted] sir who is our Kala-Azar doctor. They said how the doctor speaks, how he talks. When he went, then people’s mind opened [saw things differently]. How the nurses, etc. do things.

-

I - Even after that do some people behave same or everyone changed?

R - It is Okay

I - It is okay. Have you get your wife tested?

R - Yes sir, She also has HIV

I - Okay, she also has. Does she also have Kala-Azar?

R - No sir

I - So what precautions are taken for this?

R - She does not have Kala-Azar. But she has Lump in chest (Breast).

I - So is she also under treatment?

R - She is getting treatment. We went to [redacted]. There she got treatment. One Lump was removed and one lump was left. Now it has increased to double its size. There were 2 lumps. They removed one and left other.

I - So tell this that the health facilities which are available what do you want? What do you think that what else should be here in health facilities?

R - Here are many animals sir, Cow, Buffalo. It is very dirty.

I - Where?

R - Near my house

I - Okay

R - There are many Cows & Buffaloes

I - So it should not be there?

R - Dirty, Very dirty

I - Near house?

R - No, Not near house; If my house is here [*inaudible*]

I - So it was about in your house surroundings. It is dirty. And that hospital. What in hospital? What are you feeling that in hospital what else, I mean I am not only talking about [redacted]. Whatever health facilities are available in your neighbourhood. Health facilities are available where you can go for treatment. [redacted] like hospital and should it be more? Or what do you think about that?

R - I was thinking that [redacted] is great…and this hospital is also good in appearance. If you go to [redacted] and [redacted], it is not very good, the patients are lying here, they are lying there.

2^nd^ I - Tell one thing when you felt for first time that you are getting disease. Something is happening.

R - There was tingling sensation in my legs, you know tingling right? So yes I felt tingling in my leg and I used to feel twitching in my hand.

2^nd^ I - After getting all this, how much did you wait before going to doctor for first time. How many days had passed before you visited doctor first?

R - After getting disease?

I - Yes. After you started feeling you have disease, your body is weak. How many days had passed?

R - First time when I go the disease, when I was told about it, I started feeling weakness in my whole body. I started losing weight. When I started getting fever, my father told me…let us go show it to a doctor in [redacted]. He’s a very famous doctor. He’s a private doctor. So when it did not get cured…

2^nd^ I - [*interrupting*] So one thing I wanted to ask- how much time had elapsed?

R: It had been 1.5 months.

I: It had been 1.5 months? You didn’t show anyone before then? [*Patient says* no]. So why did you wait so long?

R - Thought I’ll get cured in private.

2^nd^ I - Okay, you visited private after 1.5 month. Why did you wait for 1.5 month. Why did not you just go after 2-3days?

R - Sir there was very money problem.

I - Due to money?

R - Yes

2^nd^ I - With money problem. Then what, why so. Do you know that you get free treatment in government?

R - Sir there people said “Go to government, Treatment is Free”.

2^nd^ I - Okay, Okay

R - My father took me

2^nd^ I - The also you waited for 1.5 months. First you went to private. After that you went to government. Why not after 2-3 days? Why did not you go to government first time?

R - Money problem

2^nd^ I - But government is free

R - Government, Yes, Yes, Free. When got treatment for 1.5 months. I felt that 1.5 months has passed. Then people said “Go there, Treatment is done there only”.

2^nd^ I - Okay, but I am asking this that why did you think that first I will go to private, will not go to government first.

R - Good treatment is not done in private. No, in government. Go here, go there. So I asked father to get some investigations in private first.

2^nd^ I - So, first you decided you will go to private. But private costs more. So you waited for 1 or 1.5 months.

R - Yes

I - 1 month or 1.5 month

R - Then again, I will go to government

I - Okay, How much happy are you in your life?

R - What happiness will I get. As I can be, so I am living simply.

I - Is it like before?

R - No, How can it be like before?

I - So, what is the change?

R - It is that I earn, eat (खाते हैं) and what else? The defect we get in body, cannot be removed/rectified. If people, go to prison, It is known that they went to prison.

I - So why is it like that it is a defect? Disease can be of any type and to anyone.

R - This disease is a bad one. People know that how is it. How the people are, you know. People say that I am a dirty boy. This dirty, that dirty. They say this.

- [*inaudible*]

I - Okay

I - Where were you counselled about this disease?

R - I was counselled in MR.

I - Before that?

R - Earlier, When I went to [redacted] to a private, there I was told only that you have a such disease, so you should go to [redacted]. There is our sir. [redacted] Sir, No Amit Sir. He also teaches in our neighbourhood. So he said ‘Go to MR and it is treated there’

I - What were you counselled there at [redacted] that what happens in this disease? How does it happen? And why does it happen?

R - This disease, that they all said that it happens because of wrong things.

I - What wrong? Can you tell? What wrong

R - Wrong is wrong

I - What else was told?

R - Nothing else

I - What was told about Kala-Azar?

R - Kala-Azar occurs after bite by a sand fly (बालू मक्खी)

2^nd^ I - Anything else would you like to say? Anything?

R - What?

I - And you were counselled there. So what you thought earlier about this disease. Did you know earlier about this disease?

R - No sir

I - Had you hear about it?

R - No I had heard that. HIV, AIDS, occurs. [*inaudible…”some illness happens”]*

I - Did you have a change after you were counselled in what you thought earlier about this disease?

R - When I was counselled, I thought it can be cured a little. Nothing else is going to happen. Now something can be done.

I - Okay

R - It was that I may live 10 years lesser but at least I will survive.

- People live till 50 years but due to disease dies at age of 30-40 years. They gets weakened.

I - What new do you want to do in further?

R - I want this that. I beget children, everything remains okay.

I - Will this disease affect your children? What do you think?

R - I am not going to beget children with this disease as it is HIV.

I - Was this told by doctor? Or you think so yourself?

R - Doctor told

I - Where?

R - In MR, On Ground (नीचे)/basement where we get medications

I - Doctor said? Or where you get medications he said?

R - Yes

2^nd^ I - Anything else will you like to say? About good life. What is necessary?

R - About good life. As such there should be work.

I - What work do you think?

2^nd^ I - What work would you like to do in this situation?

R - In this situation, any work where we sit like shopkeeper .

I - Okay, Anything do you want in life?

R - Other in life, I want children.

I - Okay.
