# Supplementary material for: Quality of life perceptions amongst patients co-infected with Visceral Leishmaniasis and HIV: A qualitative study from Bihar, India
Source: PLoS One. 2020 Feb 10;15(2):e0227911. doi: 10.1371/journal.pone.0227911 (PMC7010301; doi:10.1371/journal.pone.0227911)
Supplement: S3 File — (ZIP) [file pone.0227911.s003.zip › Transcripts/Patient 2 Male Age 45.docx]

**Patient 2 Age 45 Male**

*R: Respondent, R2: Respondent´s wife*

I: So…you were there in [redacted]…..but for how long you are there in [redacted]?

R: here at [redacted]???

I: I mean for how long you have stopped going to [redacted]??

R: It has been a year….

I: so…..what work do you do here?

R2: He doesn’t do anything…not able to lift any weight…how will he work?

I: How do you manage expenses? So he doesn´t work at all?

R: no…he can’t …the whole body is full of aches…not able to lift any weight….

I: so..who earns then?? You do?

R: pause….Yeah…I do…

I: what do you do?....

R(wife):I sow seeds in the season….

I: can’t he do any work at all?

R: no…

I: not even sowing seeds…like you do….

R: no…..

I:How do you spend your time? (Pause) ..I mean What do you do after getting up?

Responder - I get up, take bath, ……… then have food …….. (Pause)

Interviewer - Then ………. take rest?

Responder - Yeah I take rest………..

Interviewer - Then……… till evening…chat with neighbours??

Responder - Yeah…4-5 friends come and we chat….

I: chat…anything else...like watching TV??

R: no…I don’t watch…

R2: we are poor people…We don’t have TV...

I: not even at neighbours place where people watch in groups??

R:No….it is not there….how the poor people will have TV??

I spent time with kids… small kids

Interviewer - Does kid go anywhere? To Aaganwadi? Oh he’s very small. How old is he?

R2 - Yeah…………he’s about a year old.

I: Is there an anganwadi nearby?

R: Yes.

I: Does he go there?

R: Yes he goes.

I - Do you get any ration (take home) from Aaganwadi?

R2 - No….we got nothing…

I: when you were pregnant??

R: no…we never got anything…not even on the delivery of the kids…we got 7 kids but no money received from the government.

I: Despite 7 kids, why? Now, the government gives incentives?

R: hmm…but we never got any money

I - How many small kids, How many Boys and girls.

R - 7 Small kids. 2 Girls and 5 Boys.

I - All kids are small or some are big?

R - No one is big …some are big. He is 10, 12 or 15 years old. He studies here and there.

any kids born in last year?

R: no…smallest one is of one and a half year… he runs….

I: Do you take her to the field too?

R: No he does not go to the field. Elder child not working. He’s actually small. If he works now, it will cause more trouble.

I: Okay so how was the last year for you? You have been here for one year, you’re saying right? You left [redacted] and the two of you are staying here.

R: Yes.

I: So how was the last year for you? How was it, means, as you are saying that you were ill. You are not able to work. In some way, what would you say- was it good or was it bad?

R2- Here the medicine is going very well.

R- If the medicine is going on, it is good.

I: And until the medicine was not going on?

R2: I used to lift him and move him.

I: So the weight was very little?

R: The last year….it was very bad. My weight was very little. I was 20 kg.

I: 20 kg?

R: Yes.

I: My weight had become 20-25 kg completely. Not able to lift any luggage.

I: You were not able to walk?

R2: No, no not at all.

Interviewer - How is the weight now?

Responder - Now….approximately…. it is 48-50 kg.

Interviewer - last year …were any children born?

Responder - No.

Interviewer - The smallest one is of 1.5 years, right?

Responder - Yes, he runs now [he is over 1 year old].

Interviewer - How did you come to know about illness at the very beginning?

Responder - At Brick-Kiln in [redacted]. I was working and started experiencing fever, went to a doctor and he gave the medicine and Injection.

Interviewer - That doctor lived around the same brick kiln?, was government or Private?

Responder - No he was around only. He was near [redacted].

Interviewer - What do you mean by [redacted].

Responder - [redacted]is a place in Haryana state.

I: the hospital was there in [redacted]…

R: yeah..it was there….

I: Was the doctor in government hospital or private?

R: [pause…thinking] It was private.

I: did he take money for consultancy??If he took it was private one as no doctor charges money in government hospital

R:yeah…he took money for the consultancy…

I: yeah..then it must be private as in government , you don’t have to pay for the consultancy…

Responder - It was a private…fever was continuous….in spite of having treatment…fever kept happening. I was taking medicines …but I could not get rid of the fever…

I: this fever episode was of which year? Do you remember? Of 2017? Before then?

R2: fever was there since November/December and the fever was there since then continuously….he got himself tested in hospital in…around October…[pause, silence]

I: so…fever was there and you were taking the medicines…

R: Kept taking it.

I: for how long the u took the medicines?

R: The medicine went on for about 6 months..

I: did he do some tests also?

R: yeah..he did some tests…

I: what did he convey to you regarding your illness?

R: Got tested, and was told that there is a little TB. Doctor told that there is a spot in lung and it was TB. And I was started on medicines..

I: was the medicines free of cost or u had to pay for it?

R: No we had to pay for the medicines,,

I: but the medicines for TB comes free of cost..isn’t it?

R:we went to private…we were not aware of the treatment options in government hospital ..that it is free of cost….

I: so…you had to purchase all the medicines for TB during those 6 months of treatment?

R: yeah…we gave money, did all the purchase….

I: ok…then what happened?

R:The treatment went for 6 months…….

I: was there any improvement?

R: I took the medicines…..But there was no relief…I bought the medicine had it but then realized that I will not survive and told my employer that I want to go home and I came home from there…when I came home, then…

I:

Interviewer - Meaning when you found out about your illness, about TB, was your wife with you?

Responder - Yes, My wife was there with me.

R2: When we returned home, I met my parents and my other relatives…they were all at home. My mother came, my fufaji came….

I: Then you told them that?

R: then they brought him to Patna..

I: no…but what happened at home in [redacted]? what was done there? Take your time and answer…

Responder: I went home…then..(thinking…) I went to get tests. I went to [redacted] and got tested. I went to a private practitioner…

I: so..what did he say?

R: he asked for blood test and x ray…. got myself tested (X – ray) in [redacted] to a private practitioner

I: so…u came back to [redacted] and got yourself tested… then what happened??

R: He told that I have TB.

Interviewer - But TB was told to you earlier also.

Responder - Yeah, But the doctor said that he would like to get another test as he doesn’t have faith in the test done in Haryana.

Interviewer - But this doctor was also private?

Responder - Yeah…this was also private doctor.

I: so this was also private treatment..till now u were not able to reach government hospital?

R: yeah…all private…

Interviewer - How much money u have spent on your treatment?

R: money ???

I: yeah..pls tell me approximately regarding your expenditure in the 6 months?

Responder - In haryana..(thinking)….for 6 months………. Rs.25000 to 30000/- (Thirty Thousand) has been deducted. My owner has deducted this from my money….

Interviewer - so your owner deducted 30000….due to your illness??

R: yeah…

I: how much money on medicines??

Responder - He cut that medicine for money- Rs.30000/- (Thirty Thousand) on medicine & investigation.

I: so..30000 on your medication and treatment… here?

R: Did that and then came here.

I: And how much for tests and everything? Combine everything and tell me.

R: no sister…that much spent in [redacted]…In [redacted], approximately over Rs. 100000/- (One Lakh) was spent ………

I: one lakh..(surprisingly)

R: yeah sister….I spent it at home….at last I became helpless. There was no one with me….No money was left. So I got some tests and all done with some doctor and…[*trailing off*]

Interviewer - so all one lakh spent in [redacted]?

Responder - Yeah all this money was spent in [redacted]. Spent on Blood test, urine test…after doing that, the fever kept coming….and then he said that Kalazar test is needed….then he did the Kala Azar test again and discovered Kala Azar. But then he said the treatment of Kalazar was not available there, hence I was referred to Patna. I came to Patna, I was admitted here in patna….since I got admitted here, I get medicine.

I: so..u got medicines here…

R: yeah sister….but first, I became a pauper.

I: yeah..u are telling this..and u were not earning also…and for work also you were saying that you couldn’t…if you had become 20 kilos…

R2: No work, the whole body was drying up (shrinking), becoming very thin.

R1: if I would not have come to [redacted]…my death was inevitable… I was very lean 20 kg, not able to work …..I came to [redacted] and luckily I was saved…

Interviewer - Who told you about [redacted]?

Responder - A person from [redacted].

Interviewer - Doctor or Someone else?

Responder - Doctor

Interviewer - [*long pause*] Your wife knew everything since beginning, about the disease status also?first TB..then Kalazar…then??

Responder - Yeah, But she was not there with me in [redacted]. My Father in Law was there with me in [redacted]. I was admitted for 1.5 months.

Interviewer - Oh………. Your Father in Law was there

R: Yes

I: Any other test was done?

Responder - yeah…tests were done here…report was given to me….TB and Kalazar was there, but then I was told I got another one also…HIV [was later diagnosed]. So, they told me that I have three diseases.

I: so..u were diagnosed to have three diseases? First TB… then kalazar and HIV…

R: They said one more.

I: So then, how did you feel when you heard about your disease?

Responder - What should I feel ……………… (Pause)? I thought whatever was destined to me has happened…….. and what has to happen will definitely happen……

Interviewer - Did you feel sad?

Responder - Yeah I feel sad, but the moment I came here. …in [redacted]…I was relieved

Interviewer - Did you tell your HIV status to your wife?

Responder - Yeah, When I went back from hospital to [redacted], I told my wife.

Interviewer - During the stay in Patna, Did you talk your wife on phone?

Responder - No……. I didn’t

Interviewer - Did you tell your disease status to anyone else except your wife?

Responder - Yeah, my Father in Law knew this as he was there with me in the hospital.

Interviewer - Rest of people in Community member?

Responder - No……… I haven’t told.

Interviewer - Only you, your wife?

R: I have not told it to anyone in my family…not even to kids…

I: so only your wife and father in law know about your status….how did your wife react to this news?

Responder2 - yeah…after knowing the status‘मेरा दिमाग़ ख़त्म हो गया’…I have small kids..if something would have happened to him, who will look after my kids??

Interviewer - What do you think about…meaning the live you are living now after your illness, meaning what things are important in order to live a good quality life?

Responder - he is taking good medicines now….The person should be alive and earn to livelihood to look after the children, medicine should be available in case of illness.

I: so medicines should be available….anything else - Surrounding?

R: I want to become the way I was before the illness…

I: ok….what all is necessary for you? like…you told regarding medicines…your health…

R: yeah sister..(pause)

I: so..u should be taking the medicines timely….

Responder - yeah….

Interviewer - How is environment? Air, light?

Responder - environment is good….

I: There’s no problem with that?

R: Everything there is good. all people are nice….

R: yeah sister…all are nice…all are earning well…are healthy….

I: how is the environment?

R: all is fine

I: regarding air..light..electricity??

R: Bulb is there, house is there as there…………. Of Kacchha, त्रिपाल पर सोते है, ज़मीन पर (Sleep on the floor). It is a kachha house

Interviewer - Is fan there?

Responder - No, there is no fan.

Interviewer - How do you cook?

Responder - On Chulha, using wood. Using sticks.

Interviewer - Did you cook in the morning?

Responder - No……… started early in the morning with [redacted] train. Hence I couldn’t cook.

Interviewer - What the children will eat at home? Do you have Chivra (चूड़ा) at home?

Responder - No,…………… we don’t have………. They will cook rice and eat with chokha (mashed potatoes). The big one is able to cook. He can eat.

Interviewer - Your family life should be good…what else do you think about this?

What are your expectation?

Responder - I think all the family members should be good..

I: what do you think regarding your family, like since you got your illness? What should be there so your family life is good?

R: I think that I should be get rid of the disease… If I become alright I can take care of my kids……. If I die…… (Sadly) my family will end. One person’s death means 10 people’s deaths. If I am alive…10 people will live…(long pause)

Interviewer - What are you doing now? Now you’re not able to do anything?

Responder - Nothing

Interviewer - But u told that after taking medication… you told me right your weight increased 20 to 48 kg. Still not able to do?

Responder - No……….My strength is coming back slowly….But I don’t have money to buy juice and milk, I am eating only rice, Dal &………….not eating something which will boost my power so that I start working.

Interviewer - So you think if you would have money…. You would have bought nutritions food for yourself?

Responder - Yeah, I would be able to buy and eat. I could have taken foods which bring me strength [*referring to juice and milk*]. I would get my strength back sooner.

Interviewer - What do you think about your environment, your living condition & your house?

Responder - How will I think it’s okay, sister? What will I think….. but I don’t have means……….. what can I do…I also wish that I should sleep well ….whatever I have, I spend my time on that only.

Interviewer - In [redacted]………. How was the living situation?

Responder - Same like here……….. we used to sleep on floor………….. use to cook using woods………

I: kids used to stay with you??

R: yeah..all of them were living with us….

I: how u used to cook there at brick kiln?

R: same..like woods and chulha….

Interviewer - Did [redacted] (Head of the Brick-kiln) used to provide some food to you?

Responder - No……… he used to provide the ration for all the family members for every 15 days……I usually got salt..spices..and other cereals(Long pause)

Interviewer - What do you think about the type of care & treatment which you are getting? Or the kind of doctor you are getting who are taking care of you. How are they??

Responder - Everything is fine, sister.

Interviewer - Please feel free,All of them talking to you nicely?

R: Yeah…they talk nicely…

I: The persons who used to give medicines to you..how was their behavior?

R: it was ok

I: Do u get medicine on time?

R: On time.

I: What is your perception regarding your medication and treatment?? Feel free to talk…

R:…yeah…I think just that how long will I eat this medicine. I can’t go out…every month I have to come…if I get three month treatment I could have gone out…could not go any where. Every month I have to get medicine.

I: so…u think that u can’t go out to earn as you have to come here every month…And u want medicines for at least 3 months… so you must feel bad about this?

R: I don’t feel bad Sister, but if I had gotten medicine for 2-3 months at once, I could have gone outside to earn. Like this, I am not able to go anywhere.

I: I see. This is definitely right. What else do you think this, uh…anything else you want to improve? The medicine you are getting and the treatment you are getting- what improvement do you want in this, so you will feel better? One thing is okay you said that whoever is around talks nicely to you…

R: Yes.

I: No one behaves badly with you.

R: No.

I: You are also getting benefit from the medicine. Other than this, do you ever think like this medicine…one thing you said is that if you had gotten all medicines at once, you could have gotten out to work. Like this you are having to come very month. Any other things besides this that you think can be improved?

R: What should I say… we are not that literate, so how can I say?…I don’t know anything..If I get 4-6 months treatment…I could look after kids and I could go out and earn…in this situation I can’t go out…I can’t earn…

R2: what should I say??we can’t express our feelings..how will we tell it to you…but there is one point that if we medicine together, we could have moved to some other place..earn and eat…….

Do you get wages here at [redacted]?

R: I get…..but only 4-5 kg of cereal….we can eat only once or twice a day…but how can we just eat? What about taking care of the kids…we have to make our house…have some money for back up, to take care of children’s needs and health.

I: I see. So there’s one thing that keeps coming up that if you could get the medicine in one go, then it would be good.

R: Yes.

I: Anything else, like you are having to come here every day. Any other solution you can think of? Some other think that can benefit you?

R: What benefit can we get, Sister?

I: Like you were also admitted for so many days. Some other think you thought like this should not have been like this. If this would be like that, it would be much better. Because you only will be able to tell us, right? If we are giving it, we are often not able to tell what problem we are facing.

R: Yes

I: You have been given an opportunity to express what you are thinking, meaning if this thing was better, then we would be more comfortable. Regarding medicines, regarding treatment, or some other thing you may be thinking of right now. You can take some time if you need 2 minutes time…but what you will tell us will be very important for us.

R: *Long pause*.

I: Okay if you are not able to understand then no problem. Okay like if something is nearby, like you are coming, you are coming by train. Do you need any other facility?....like…conveyance? Or like the medicine could be given to you over there? Would you benefit from that? ... No? How will you benefit from that also, you are saying you want the medicine at once to go elsewhere.

R: Want to go somewhere else.

I: This is your purpose right?

R: Yes.

I: Now how many months treatment have you done? Is your treatment complete?

R: TB Treatment is on…HIV treatment is also continuing…Kala Azar one is over.

I: Okay so HIV one is going on?

R: It is going on.

I: The HIV one you will have to take continuously.

R2: Yes they give for one month.

R: Told me that I have to take it for a lifetime.

I: Will have to eat it for a lifetime. Can do like this only- they give you for a few months, then you go, and then come back and get it again. It can happen like that.

R: That’s it right, I have to come back...Kids are very small…it is difficult leaving them alone…at home….and neither am I able to do anything [referring to lost work opportunities due to treatment]

I: So now what hopes do you have from your life? Meaning what do you think, like now you are aware that you have this illness. Now what do you think? What else can you do with your life…

R: Yes Sister. What can I say?

I: Even then what do you think? Like when we get better, or we are able to lift weight, what all will we do? By which my life will be better going forward? Do you think anything like this?

R: What should I think? I want to be as that of previous..I used to have spare money to feed…now I don:t have money…

I: meaning you want to be as you were before?

R: Yes…earlier I used to have saved money. Now it’s like…I used to have two paisa in my hand…now I don’t even have ten five rupees…if I go back from any place, kids ask…papa give us money…but now I don’t have money….If I had two paisa..I would have saved it …for marriage… …kid r of marrigable age…but nothing is there in the house… How will I do that

I: Who? The one who is 15-20 years old?

R: Yes the one who is 15-20 years old.

I: 15 years is young for marriage!

R: He is 17 years old now.

R2: In our culture, marriage happens at an early age.in ours..marriage happens at early age…he is tall..like me….marriage happens at early age….early age marriage…people in neighborhood says that such a young boy….. u have kept him at home…will not get a girl…will grow old… will grow more old….In 20 years…..25 years…will marry…will not get a girl..

I:ok..why is this so?

R: will say that boy is old…

I: in 20 years..he will be old??(Laughing)

R: He is as tall as his father…here marriage happens at 17-18 years..

I - How Young is your girl.

R2: She is young, quite young.

R – Both girls are younger than five Boys.

I.: Oh they’re both younger.

R: Yes both are younger.

I: Meaning you think that…so because of this, do you face any trouble in that? Meaning, because of your illness, what you had thought like you will get the married…do you face any trouble with this?

R: Yeah sir…I face trouble…I face trouble(repeated twice) I am the only bread winner…the main man earning. when the kids will reach marriageable age…how will I do then.. You have to think about this- how will I do it? And my wife…she is not that smart that she can do something on her own. If she could get some support, then you could think she may do something.

I: And what about your father in law?

R: No …He has his own family. Will he look after his family or look after our family?

I: And your mother and father?

R: Not there. They passed away.

I: So no one from there? Your brother that you were talking about-

R: The elder brother is separated from me.

I: He doesn’t help out at all?

R: No. He is not supportive at all…he left me alone and ran away when I was ill…no talking terms……

I: So you are not on speaking terms?

R: No.

I: so u don’t have anyone to support your family?

R: [*looks very sad]* None…

I: Neither from her family nor from your brother?

R: No. No help at all.

I: Meaning whatever needs to be done needs to be done by you.

R: Yes, whatever needed to be done, I had to do on my own. Nobody helped me.

I: Now what do you want?

R: Now what…Sir…I think that…I am this kind of man now, how will I get my child married…how the other children will live? How can I take care of them? This is what I think…that how will they live.

I: I see. And did you think this way before you found out about the illness?

R: No…I was not thinking so

I - What do you think after the disease.

R – Yes now I have started worrying about their upbringing and their marriage.

I: Now that your older son has grown up, where- does he work in some school nearby?

R: He stays at home only.

I: so for how many months you want the medicines together?

R: 3-4 month

I: And then 4 months later?

R: I’ll come and get it again.

I: And where are you thinking of going in those 3 months?

R: I will go somewhere and earn…with kids…

I: Like at the brick kiln?

R: Yes.

I: Do you get enough money at the brick kiln?

R: Yes I get it in the season.

I - How much money does it get on the brick kiln in a month? You were telling me in a week right?

R: yeah…we get ration in every week.

I: You are telling me about the food rations for 15 days.

R: And I have to stay there for 9 months at a time.

I: So how much money do they give every month?

R: for every 15 days ..we get ration..of two thousand..four thousand..then rest of the money will get during **season**(before rainy season when the brick kiln stops functioning and the workers are planning to go back to their native places)

I: If you both work, you will get for both people?

R: we will get for kids also

I: what about kids?

R:we get ration for a week..a week is of 15 days…

I: but a week is of 7 days??

R: no..there a week is of 15 days…kids get ration of four thousand rupees every week…

I: do kids also work?

R: no..but if they are with us..atleast they will eat…we two will earn..and they will eat ..

I: what about your wife? If she doesn’t work? Even then she will get the ration?

R: She will cook for us hence she will also eat…

I: no..I was enquiring about ration? Will she get that?

R; yes…she will get

I: mean.. whosoever is with you…u will get ration for every one?

R: yeah…for everyone…

I: Okay then.
