# Supplementary material for: Quality of life perceptions amongst patients co-infected with Visceral Leishmaniasis and HIV: A qualitative study from Bihar, India
Source: PLoS One. 2020 Feb 10;15(2):e0227911. doi: 10.1371/journal.pone.0227911 (PMC7010301; doi:10.1371/journal.pone.0227911)
Supplement: S3 File — (ZIP) [file pone.0227911.s003.zip › Transcripts/Patient 20 Male Age 24.docx]

**Patient 20, Age 24, HIV-VL**

I - Tell me about you. Where have you come from? Who all are in your family?

R - Sir, I have come from [redacted] district. I have taken treatment these in government hospital. They said to go to [redacted] for further treatment. I come to [redacted] and treatment is going very well.

I - Tell me from the beginning, how did you get this disease?

R - I don’t know, how did I get the disease. I only know, when my blood was investigated, I was diagnosed with Kala-Azar.

I - How were you before the diagnosis?

R - When I took meal, digestion was not proper and there was continuous fever. As I was not taking meals. So I consulted to various people (doctor).

I - How many months back, did this happen? (The first episode)

R - I had two episodes.

I - Had it happened two times?

R - Yes Sir.

I - Ok

R - I had gone to [redacted] (Hospital) and some medicines were given but…. (Pause)

I - (Interrupted) Medicine didn’t work?

R - No, Medicine didn’t work.

I - Ok (Accha..)

R - It happened again.

I - One minute, What they had said?

R - They had said that it was Kala-Azar.

I - Had they confirmed by doing investigation?

R - Yes

I - When was this happened?

R - I think, 2012

I - Did you know that you had Kala-Azar at that time?

R - Yes

I - Did you know about HIV, at that time? (Suffering from HIV)

R - No, they had said nothing about it.

I - What medicine were you prescribed at that time?

R - Capsule (Indicating size with fingers) was given.

I - For how many days, it was given?

R - Approximately ….Ahh…. 32-33 days

I - Ok

R - After taking it for these many days, I was not well. So I again went (to hospital) and they said that I had to take injection. I took 32 injections there. I was not well over after taking these injections.

Again I went (to hospital) and said him that I was not relieved from fever, fever was there as it was earlier.

I - How many days or how many months had passed?

R - Calculating…………….meaning…………..It was started in 2012. It was on & off for 6-12 months got relieved and then again started in the same manner.

I - Ok (Accha..)

R - While all these were happening…………. Someone told me to go to [redacted].

I - Had you first gone to Government hospital or Private hospital?

R - I had gone to Private hospital but they were not able to diagnose it.

I - Why had you gone to Private hospital first? Why not to Government hospital?

R - First Private……….Ahh………….It happens in villages usually.

I - Corner shops?

R - For minor ailment (problem) (हल्का फल्का)

I - Yes, for minor aliment.

R - I thought it was only fever, cold and cough, So I took medication (from private doctor).

I - Yes

R - After that, I was not relived after taking (those medicines). So I went to Government (Hospital), where I was told (about the disease)……….

I - Ok (Accha..)

R - ……that I had Kala-Azar when I had gone to (Government) they had told nothing (about HIV), only they had said about Kala-Azar. I came to [redacted] then I was told about HIV.

I - Oh my God! (बाप रे)

- Ok

- You first went to Private (Hospital), then in which Government hospital had you gone? [redacted]?

R - To [redacted] (Hospital)

I - Had you gone anywhere else?

R - No

I - Had you been sent from [redacted] (Hospital) to [redacted]?

R - Yes, Sir.

I - What they had told you about [redacted]?

R - They told me, “You go to [redacted] for investigations after that I will treat you here”.

- They didn’t told me that they were referring me.

I - Did they not tell you? (About refer)

R - No

- They sent me by fooling me.

I - How did you feel at that time?

R - That time I was disappointed. I thought, “If you don’t want to treat me there then you should have told me to go to [redacted] directly”.

I - Yes (……….)

R - Even that, I didn’t thought about it much. I thought even if he had send me [redacted], it was not a problem.

I - Yes (….)

R - After I came to [redacted], everything was good, food, medications and all. After starting treatment from here, I am good.

I - When you came to know about HIV for first time?

R - Here only.

I - [redacted]?

R - Yes Sir.

I - Had they investigated you for HIV at [redacted]?

R - No

I - Was any investigation done?

R - No

I - About how many tests do you know?

R - There ([redacted]) they had done 3-4 investigations but I don’t know about them.

I - Had blood investigation been done?

R - Yes

I - Ok. But you were not told about HIV.

R - No

I - Ok

- When you have come to [redacted]?

R - Calculate it. Amm…………. I have come in the month of kartik.

I - Oh (Accha)

R - I am good from the month of Ashvin (………….)

I - Ok. You stayed here for 30 days.

R - No. Not for 30 days I stayed here for 40 days.

I - You stayed for 40 days.

R - Including duration for investigations, I stayed here for 40 days.

I - When did you leave from here?

R - I left from here on the day of Chhat Puja. On the last day of Chhat Puja.

I - So, You have come today for follow up. Isn’t’ it?

R - Yes

I - Ok

- You tell me, how was your last year? Meaning, after you come to know about the disease, what did you feel in your body?

R - I was not able to understand from what kind of disease I was suffering from. And why I was not getting rid of it. I got really devastated. I had a hard time due to treatment.

I - What do you mean by devastated? (तबाह हो गया था)

R - I mean, I had to spent lot of money.

I - How much money you had spend?

R - So much money. I don’t remember the amount.

I - Approximately how much? Any idea?

R - No Sir.

I - Was it more than I lakh?

R - More than that sir. You can calculate it. I am suffering from 2012 and now it is 2017-18.

I - Hmm……..Hmm…….

- All these expenditure was done in Private hospital? Was these any charge in Government Hospital?

R - No

I - Ok

- What is the impact of this disease on your mental health?

R - Right Now, I am fine. I have no problem. But as I have done cutting of crops this season, so have gotten cold & cough.

- As sir have called me, I will tell about cough & cold. He will give medicines.

- Otherwise I am fine.

I - Ok

- What do you work?

R - I work as a farmer in fields.

I - Before the onset of disease?

R - Before the onset of disease as well as after it.

I - What was the impact of disease on your work during illness?

R - During illness…………….Amm……….When I was in village did not want to work. I didn’t wish to work. I wished to sleep all the day. I was not able to take meals properly. So I used to sleep all the time.

I - What did you thought about your future life during illness?

R - What would I thought I used to thought that I was poor and how would I able to get treatment. But I was doing my best (for treatment) up to the extent I can do.

I - Hmm Hmm

R - (Coughs) God was seeing all this and he saved me.

I - What were you thought about your future, during that time? What were you expecting from life at that time?

R - What would I expect. I was not able to understand anything at that time. What can I say?

I - What? What do you mean by you were not able to understand anything?

R - Meaning I was not able to understand anything (………………………………..) 7:07

I - Do your neighbours know about your disease?

R - No, Sir

I - Your family members?

R - No, Sir

I - No one know about your disease (in your family)?

R - Only my mother knows about it. She was living with me. Other people know that I am getting treatment for Kala-Azar in [redacted].

- They don’t know that I am suffering from HIV.

I - Do your mother know about your HIV.

R - No.

I - No. All Right

R - He (at [redacted] hospital) said to me to not tell anybody about my HIV status. He told me to keep this information restricted to myself.

I - Ok Who told you that?

R - xxxxx Sir

I - He told you

R - Yes Sir.

I - If anybody from your village came to know about your HIV status. What will they say about the disease?

R - I don’t know sir.

- They will say that he is untouchable. Don’t touch him.

I - Will they say it about HIV or Kala-Azar?

R - HIV

I - Why will they this about it (HIV)?

R - Why will they it. How can I tell you. I don’t understand it.

I - All Right. But are you sure that they will say like it?

R - I also know about it. Although I am illiterate but I know that it is an untouchable disease.

I - Ok

- Do you know how it get spread?

R - No Sir.

- I didn’t even know about it. When I came here, I heard it for the first time. I was not aware about all these things.

I - When they told you about the disease (HIV status), do you here any idea how you got this disease?

R - No

I - You do not have idea.

R - No

I - Are you married?

R - Yes, Sir.

I - Do your wife know about it (HIV status)?

R - No, She event don’t know.

I - All Right

- Do you know that she has also chances of getting this disease?

R - No.

- I have been told by them ([redacted] hospital) not to sleep or sit with my wife. So I have stopped sleeping along with my wife.

I - What did you tell to your wife about your disease?

R - I have told her, “Doctor has told me that I would not able to have child”. Because he has given me such medicine that I would not be able to have child. So what’s the purpose (of making sexual relationship)

I - All Right.

- What about sexual relationship with your wife?

R - I don’t do it now.

I - Hmm…..Do your wife know about your Kala-Azar?

R - Yes

I - There is no problem in it. They will not say that it is untouchable.

R - No, They will not say it.

I - Is there any other case of Kala-Azar in the village?

R - Yes. Many people had suffered. Around 35-40 people had Kala-Azar. I was among the last ones to have Kala-Azar.

I - Where did other people go for treatment?

R - First they used to come to [redacted] and after that they continue their treatment in [redacted] district.

- But I don’t know that any other people are also suffering from this (HIV).

- There are many people in my village (Suffering from the disease). If you go to search you will find about 40-50 cases.

I - Kala-Azar?

R - Yes

I - Have you any idea about HIV (Number of cases)?

R - (Laugh) They will be diagnosed only if they get investigated for it.

I - Meaning, People don’t talk about it? Itsn’t it?

R - No. No one

I - All Right

- What are your expectations from future? What will you do in your future?

R - I think that I have got my life again. So I should eat will, should feed my child well, Should make him educated and live a good life.

I - So , You want to eat well, feed your child well and what other things?

R - And I want to live with my family and nothing.

I - What is the behaviour of your wife and your family towards you?

R - There is all Right. They do not say me anything. Even If I go to wake and sit with any person, they don’t say me anything.

I - Do people talk to you in the same manner as they used to talk previously?

R - Yes

I - Your friends?

R - Everyone. In the same manner.

I - What is not in your life right now? If it was present it would has been better.

R - I think about children only. I have two children but I think that it would have been better if they were three.

- Although, it is better to have a small family. But I think that it would have been better if I had three children.

- Then I thought that two children is also sufficient. There will be a lot of expenditure in educating them.

I - Will you educate them private school?

R - They will study in Government school. I will not be able to afford (Private school). I am poor man.

I - Then where will be the expenditure?

R - What?

I - Where will be the expenditure, if they will study in Government school?

R - They don’t give books (In Government hospital)

I - Do they not give books?

R - No. They don’t. And also for school dress I have to spent.

I - I understand.

- Tell me about the treatment. How were the staffs there? How do you feel after taking to them?

R - They were very nice people.

I - (Interrupted) what do you mean by very nice?

R - The made my life. I used to cry a lot. I had believe that I will die. But they counselled me very well. They said, “first take treatment form here then tell us about the outcome of treatment”.

I - Who were they?

R - XXXXX Sir (12:30) and 3-4 other people.

- When I was referred here I was not aware of the disease (HIV). But when I know about it I used to cry. The person from [redacted] had fooled me and sent me here.

I - Did you cry?

R - Yes

I - What were the thoughts in your mind when you cry?

R - If the doctors (at [redacted] hospital) didn’t want to treat me then he should have told me directly to go to [redacted]. It would have been better. But he sent me secretly to [redacted] and told me to go to [redacted] only for investigation and then he will treat me at [redacted]. From [redacted], I called person (at [redacted]) and told him you have cheated me (Abuses him). He should had told me to go to [redacted] for treatment. What was the problem in it? I didn’t have problem in it. I would had came here with all preparation. But here all things were provided.

I - Who told you that?

R - There.

I - Where?

R - The person in the village. Meaning the person at District. He should had told me to go with all preparation like clothes for treatment. He sent me without any preparation.

I - Who had sent you?

R - The person at Government hospital

I - The Government hospital person?

R - Yes

- He told me to go to [redacted] for investigation and after that he will treat me. Only the investigations will be done at [redacted] and the treatment will be given at [redacted] hospital.

I - Had he said like that?

R - Yes

- When I came here ([redacted]). I was tricked. But then I thought it was matter of life. So I would take treatment. I was taking treatment from many places. So I thought Let’s try it here also.

I - How far is your home from here ([redacted])?

R - A very long distance.

I - How much? How you come from there?

R - I come by bus.

I - How much time does it take?

R - I boarded the bus at 5 o’clock in morning and reached here by 9:30 AM.

I - Oh my god!

- Is there only one bus?

R - No. I come here by direct bus. Otherwise I have to go to Bihar (Sharif), then have to change bus there and then again have to change bus [redacted].

I - Does it (Bus) come directly?

R - The bus in morning comes directly. So I used to board this for coming here ([redacted]). If I miss the bus then I have to go here and there. Fare cost also becomes more.

I - Do you after getting HIV you have to take medicines regularly? Daily.

R - Yes. I take medicines on all 30 days.

I - Ok

- From where do you get your medicines?

R - Here only.

I - Do you come to [redacted]?

R - Yes. This is my centre.

I - Is it nearest centre?

R - Yes. They had said about [redacted] district Centre. But I told them that I had not gone there and also had not seen. So I told them that I came here for treatment…..

I - (Interrupted): Which day do you come here for medicines every month?

R - On 28^th^

I - On 28^th^

R - Yes

I - Ok. Do you have taken medicine on all days till now?

R - Yes. Even now I have the container of medicine.

I - Do you face any problem in taking medicine form here in terms of travelling?

R - What will be the problem? I come and go by bus.

I - What is its effect on your daily income?

R - It costs around Rs.500-600

I - Does any monetary benefit given to you?

R - Nothing. Nothing Sir

- They had taken (sample) for investigation and had told me that some amount of money will be given. But nothing has been given.

I - Ok.

- Hmm………… You go to hospital for treatment what do you think about staff or any other things that if it was present then would have been better? What do you all things matter to you?

R - (Coughs)

- I think wherever I go there should be nice people.

I - Hmm

R - But there are not such people available.

- Only one person is here who is nice. He had helped me a lot, that’s why my life was saved.

- Otherwise I was suffering for a long time.

I - Anything else?

R - No Sir.

I - What do you think your life now?

R - I want live a good life now.

I - Do you cry now?

R - No. Not now.

- (Abuse) Only one problem is there. I have to come every month for medicines. They do not give it to other person. How can I come every month? I think about going to other place for work. But how can I go?

I - You have to come.

R - Yes Sir. This is the point.

- This is the only problem.

I - There is a good thing about it. Although it (HIV) is not treatable but if you take medicines regularly you will be able to live up to the same age compared if you did not have the disease.

R - After taking this medicine, it show beneficial effects.

I - Do it shows beneficial effect?

R - Yes

I - Has Kala-Azar been treated?

R - What?

I - Has kala-Azar been treated?

R - Yes.

I - Do you notice anything else in your body due to simultaneously having HIV as well as Kala-Azar?

R - My whole body was emaciated. There was blackish depigmentation of skin. My abdomen was swelled up.

I - Hmm

R - I didn’t feel any appetite.

I - How was Kala-Azar treated?

R - Here only Sir.

I - What was given for treatment?

R - Tablets as well as IV fluids were given.

I - For how many days you were treated?

R - Only 8 bottles (IV fluids) were given to me. I was lucky.

I - Do you have any other disease during this period?

R - No. I did not had any other disease. Not even diagnosed on investigation.

I - Did your wife got Investigated (for HIV)?

R - Yes Sir.

I - Blood Investigation?

R - Yes

I - Is she also suffering from HIV?

R - No. Investigation is not done in my village.

I - What?

R - Investigation is not done in my village.

I - Ok.

- Had she not come for Investigation?

R - She had come here for Investigation.

I - Had she come?

R - Yes

I - But……….

R - (Interrupted) No

I - She did not have.

R - No

I - Ok

R - I am thinking to get my wife investigated (for HIV) again in 2-3 months. Then after one year again. What’s the problem?

I - Investigation?

R - Yes

I - Good. It’s good

R - What is the problem in getting investigated every 1-2 year?

I - Do you know you have to use condom while having sex?

R - Yes, I know. They had told me. I will not have sex at all. So what’s the point of using it (condom)?

I - All Right.

- Do you want to have sex……..

R - (Interrupted) Not now. I can bet you I don’t even think of it. I think that for the pleasure of 5 minutes, I will ruin her life.

I - Do you think so?

R - I do.

I - Do you not have any interest now?

R - Yes. I do not have interest.

I - What does your wife think about it?

R - She don’t think anything about it. Already I have two children. I am happy with them.

I - As you said you do not have any interest (in sex)? What does your wife think about it?

R - I have told her. Assume it that I have been operated and you also have got operated.

- I also say to myself that I have been given medication (for not having child). That’s all we talk.

I - which type of house is your?

R - It was kaccha house.

I - It was, meaning?

R - Now I have made a room of bricks.

I - Have you made a room of bricks?

R - Yes

I - Did you made it before as after the illness?

R - After the illness.

I - Ok

R - I have made it with cheap bricks. Before it the house used to fall down in every rainy season. I am poor person sir. I have made it by saving the money.

I - How did you save the money?

R - By selling wheat, Rice, Bengal Gram, Masoor for 1-2 years. I collected Rs.40000 - 50000. Then I made with this money.

I - Do you sell these by yourself?

R - I do not have my father. He had died. My mother is alive. We are two brothers and I am older one.

I - Did you wish to do anything other than having child? But due to this disease you are not able to do it.

R - Now I think, if I can make progress it will be good. My children’s future will be good.

I - For example?

R - For example, If I can send them in a good school. I also want to have a good life.

I - Did you think that it was possible during the period of illness?

R - No. It was not possible. I was in trouble due to my own life. How could I think of other things?

- During the illness I was not able to do a single work.

I - True

I - Then, All Right, Thank You.
