# Supplementary material for: Quality of life perceptions amongst patients co-infected with Visceral Leishmaniasis and HIV: A qualitative study from Bihar, India
Source: PLoS One. 2020 Feb 10;15(2):e0227911. doi: 10.1371/journal.pone.0227911 (PMC7010301; doi:10.1371/journal.pone.0227911)
Supplement: S3 File — (ZIP) [file pone.0227911.s003.zip › Transcripts/Patient 21 Male Age 25.docx]

**Patient – 21, Age 25, Male**

I - What do you do?

R - Currently, I am not working.

I - What you used to do earlier?

R - I just live in village, [redacted].

I - In village, What you used to do?

R - Nothing

I - Study?

R - Yes I used to study

I - Which subject?

R - I was in Class X

I - How was your last year?

R - What happened to me, like…. I got fever first.

I - How long back you got fever?

R - I got fever in the year 2016. I took medication, and was relieved for 6 months. I bottle of medicine was infused. Then I came to my village, and saw a doctor, and he sent me to [redacted].

I - Where did he send?

R - [redacted]. He did tests and then sent me here.

I - Ok!

R - Here, on starting medication, my fever was relieved, (*inaudible*) ?

I - Did you have any other symptom other than fever 2 years ago?

R - No other symptoms, only fever.

I - Cough?

R - No coughing or anything.

I - Chest pain?

- Night sweats?

R - No I did not get sweating or anything.

I - Weight loss?

R - Yes, I had lost weight.

I - How was your appetite?

R - I had loss of appetite, and pain abdomen.

I - Ok! Anything else? Any other symtoms?

R - Pain abdomen, and fever

I - Was pain persistent?

R - The pain was always there over here...that´s it│

I - Ok!

- Any other problem other than this?

R - No other problem.

I - You went to Government doctor or Private practitioner?

R - No he was a Government doctor.

I - Did he charge fees?

R - Yes he charged fees. He is in the private sector only.

I - Private?

R - Yes private.

I - Did he get some tests (investigations) done?

R - Yes. He had done some tests.

I - How much were you charged in toto there?

- (Other person – 3 lakh)

R - I had spent Rs. 3 lakh

I - 3 lakh??

R - They were all cruel people- they didn´t tell us anything and we kept roaming.

R - I had spent there a lot on my body.

I - You used to visit the same doctor, or change doctors?

R - No, just one doctor

I - Did he not refer you some where else?

R - No

I - For how long did you come and go to their place

R - For one month, I have been going back and forth to the same doctor.

I - For how long? 5 months? 2 months?

- (Other person – 3 months)

R - 3 months

I - 3 lakh rupees was spent in how many months?

R - What I spent...the medicine for Kala Azar that I used to get…

I - What did private doctor tell about the diagnosis?

R - Doctor told me that I have Kala-azar fever………..Kala-azar……the injection for this is costly. I said this to my father, and my father borrowed money and gave it to me.

I - How much money was spent that time?

R - At least 3 Lakh

I - At a time?

R - Not at one time. As I got the medicine, I had to spend accordingly. No matter how poor someone is, they’ll do anything to save their life.

- I did not get better from there..
- Then one man told me to go to [redacted].

I - Who told you?

R - Other man told me, that go and see doctor in [redacted]. I was not satisfied for a year there. I got fever. After I got fever, I came to [redacted]and after coming to [redacted], after investigation in [redacted], I came here.

I - In [redacted], after investigation, did doctor tell you anything?

R - Told that I have Kala-azar.

I - Did doctor tell any other diagnosis?

R - Yes

I - What?

R - Told about HIV

I - Did doctor tell you anything about HIV, like how do it spreads, or what is HIV?

R - No, he did not tell anything.

- He only told that I have HIV, nothing else.

I - What happened then?

R - Nothing I came here, just medicine were started, investigation was done for a week.

I - What were you told here?

R - The tests were done. I did not have any history of coughing so they conducted sputum examinations and told me that I had mild TB.

I - So they told you that you TB based on your sputum examination?

R - Yes.

I - This investigation of T.B. or Sputum was not done anywhere else?

R - Nowhere else. Like I got X ray done, but nothing came in that.

I - Where was your Sputum tested?

R - Here, in [redacted].

I - You were absolutely fine 2 years back.

R - Yes

I - All problem started since 2016?

R - Yes

I - When you visited Private doctor in [redacted], did he tell you about HIV?

R - No, he told only about Kala-azar.

I - When you went to [redacted], and got to know that you have HIV, who was with you?

- (Other person – Father)

R - Father

I - How did you feel on hearing about your HIV status?

R - I felt strange- ultimately, how did happen to me?

I - How did your father feel?

R - My father also.

I - Did you tell about your disease to anyone in your family?

R - Told everyone in family.

I - Like your mother, father, anyone other than you 3

R - No-one else.

I - Who else is in your family?

R - Sister

I - Like joint family , uncle, aunty?

R - No

I - Ok! So, you, your sister, mom and dad?

R - We are 4 brothers.

I - Ok! So, how old are brothers?

R - All are younger to me. I am the eldest.

I - Did you tell to your younger siblings?

R - Yes, I told them.

I - One younger to you, how old is he?

R - I have an elder sister.

I - How as her reaction on knowing your HIV status?

R - She has got married.

I - Did you tell her?

R - No, didn’t tell her, she doesn’t stay here.

I - How old is your younger brother?

R - 15 years

I - You told him?

R - Yes, I told him

I - The one younger to him, how old is he?

R - 10 years

I - Then, is anyone younger to the one 10 years old?

R - Yes

I - Ok! So you have a brother & sister!

R - Yes

I - When you got to know about your disease, did negative thoughts come to your mind? Like, life is useless, or my life has got ruined? Or nothing can be done?

R - Yes, I did have such thoughts.

I - When did you feel so?

R - When I heard I have got HIV.

I - How did you arrange Rs. 3 lakhs?

R - I took loan from someone else.

I - Because, 3 lakh is a big sum.

R - My father took loan.

I - What does your father do?

R - Father does farming.

I - You have your won field/land?

R - No, he works on someone else’s field/Land.

I - Ok! Do you siblings do anything?

R - Nothing, they study.

I - Where do they study?

R - In village

I - How long did your undergo Kala-azar treatment?

R - One week. I stayed there for more than a month, but the treatment was only a week long.

I - Then?

R - They discharged me after two days. I got completely fine. Then I got fever after a year, and pain abdomen. I was not satisfied. So one friend of mine told me to go to [redacted], and see other doctor.

I - Do you know anyone, else around, with such disease?

R - One mam had

I - Is her alive?

R - Yes, he’s getting treatment from [redacted].

I - Did he tell you about his disease?

R - No, he didn’t tell me.

I - So, how you got to know?

R - I got to know, from someone else, then I asked him (Who had HIV). He told me that he got to know, about his HIV status during blood test done before his visit to Dubai. He was asymptomatic otherwise.

I - How do you feel when you hear someone else’s HIV status?

R - You have to realize your own mistake. Suppose…you don´t see others [don’t involve yourself in other people’s business- you just think about yourself. I think that, suppose what someone else does or does not do…[*inaudible*]

I - Why do you think people don’t tell one another about their HIV status? Like you too didn’t tell about your disease to people other than your family members?

R - I didn’t tell anyone outside of my family. Everyone knows.

I - What does everyone know?

R - That I had Kala-azar.

I - But about HIV?

R - They don’t know

I - That’s what I wanna know, why you or people around you don’t tell about your HIV disease?

R - Because, then they will hate HIV positive patient.

I - Why so?

R - Because it a very serious disease

I - Why will someone hate a person suffering from a serious disease? Kala Azar is also a serious disease?

R - I don’t know, but people do (hate)

I - How do you think?

R - I think if I get cured then it will be good, otherwise there is no use of my life.

I - I see. Why do you feel you should not tell anyone about your disease?

R - I feel person will think bad about me.

I - Any other thing you want to tell?

R - When I went to [redacted], doctor told me, that “I got the disease through injection”. I have no sexual relationship with anyone. I just study, I am from a poor family. No one wanted anything to do with me. Doctor (Private doctor of [redacted]) told me, when I got ill in 2016, doctor through injection, caused me the disease.

- I regretted. I got my treatment done from good doctor. (implies, despite searching a good doctor, I got HIV)

I - Who all are in your support?

R - Mummy, Papa that´s it.

I - Does any other relative keep you financially?

R - No support at all. I have taken loans from other people.

I - How will you pay back the loan?

R - Father is paying, through farming. As we keep saving up, we will keep paying the loan back. It may take two to five years. Maybe even ten years. My parents will not abandon me as long as they are alive. Even if they are not able to take care of themselves they cannot abandon me, they feel a sense of duty and responsibility for me. They will take out a loan as large as required for their son.

I - According to you, what will is needed to lead a good life?

R - I think that for me to lead a good life good medicines are required. If there is some medicine that will completely cure me, that will be nice…

- Interrupted by interviewer, no, when you were absolutely fine, what was your thought about good life?

- I had the thought, that after studies, I will do a good job, and my parents get happy.

- They did our upbringing, and when our time comes, we should help parents.

I - What else do you think should be there, to lead good life?

R - Study, then we should earn, and give happiness to parents, and brother, sister. What else is bigger happiness!!

I - Something in the environment?

R - If we earn, we will be happy, make others happy. If one studies only they can be happy, isn’t it? if one will not study, he will have to do manual labour. How many people can you support then (with such a job)? If one will study, then only can something. Now –a-days, study is very important.

I - How is the environment around your house?

R - It’s good

I - Neighbours?

R - It’s good.

I - They talk to you?

R - Yes, they do (talk)

I - Do you have friends?

R - Yes, I have friends.

I - How is the behavior of your friends, with you , for last 2 years, since you got in ?

R - It is the same, as it was 2 years

I - Any change in their behavior, owing to your illness?

R - Nothing, it is the same (as usual)

I - Neighbours?

R - They also behave normal (as usual)

I - Are you satisfied with your treatment?

R - Yes, it is good.

I - Is doctors’ behavior good towards you?

R - Yes, it is Ok

I - And staff behavior towards you?

R - It is good

I - Is there anything, that you wish to change? Like do you with any change in your treatment, [redacted]that could have been better?

R - I once read in newspaper, that a drug will come in the market, that will cure HIV. I always ask doctor’s about such drug. They said, when, it will come, we all will get to know.

I - Any change you wish in facilities being provided to you?

R - I am satisfied with the facilities.

I - When doctors at [redacted] told you about your HIV, was you treatment of HIV started in [redacted]?

R - No

I - Did doctor at [redacted] tell you, how it spreads, like how it happened to you?

R - Doctor told that, I got it from injection form infected patient.

I - How did your disease/illness affect your work?

R - I used to get fever.

I - Could you do anything?

R - I could not do anything. I used to get fever. I always wished to sleep. Used to be it high grade fever. I used to go home, and sleep, not doing anything.

I - Have you passed your 10^th^ class?

R - Yes; I passed 10^th^

I - Did you take admission anywhere for future studies?

R - No, I feel ill, I could not.

I - Had you passed your 10^th^ class, by the time u feel ill?

R - Yes

I - Why didn’t you take admission in next class (XI)?

R - I was admitted in hospital, for Kala-azar, when I had to take admission in next class.

I - And what till now? (Why not after that till 2 years)

R - I thought, when I will get fine, then will take admission for further studies.

I - What all you wish to do in life, after getting treated from here?

R - I think, If I study, I can work at some place. I want to study, like till XII. Like having XII class certificate, one can do anything.

I - XI or BA? What else you wish to do?

R - Later, I can start my business, taking loan from anyone, and will take care of my family.

I - What do you think about marriage?

R - I think, I can’t marry until I get treated.

I - Why do you think so?

R - Because HIV can spread to my wife.

I - You know, that this disease can’t be cured, once virus enters the body, you will have to take medicine whole life.

- So, will you not marry your whole life?

R - How can I marry then, When I know, it will spread to her too.

- My life is already ruined. Why run her life too?

I - What if a girl has HIV?

R - Then I myself is suffering. I will have to tolerate her too.

I - Ok! This is what your think, that you will have to tolerate her too! I will not feel good.

R - Yes

I - Ok! How do you think, will spend your life ahead?

R - How will I spend it? It will get

- It is said, there all come a medicine, that all cure HIV.

I - So, you are waiting for that medicine?

R - Yes, if such medicine comes, it’s good. If it does not, then I will spend my life like this only and will get younger brothers married.

I - Did your 15 years old brother ask you anything when he got to know about your disease?

R - He didn’t ask anything. He started crying, all started crying.

I - Ok! They are studying?

R - Yes.

I - Did you get any Government aid, while you suffering from disease? Like any financial help?

R - No, I didn’t get any Government assistance.

I - Here?

R - Here I get transportation charges, and facility for food and stay, as long as I stayed here.

I - How long you stayed here?

R - I stayed here for 40 days?

I - In your village, where from do you take your HIV medicine?

R - From Balliya.

I - And T.B. medicines?

R - From the center

I - How much does it cost you to reach there?

R - Rs. 500/-

I - Rs. 500/- from your village to centre?

R - Yes

I - How frequently do you go there?

R - Once a month

I - Do you get any assistance in transportation?

R - Nothing

I - Do you have any advice, if it were available to you, or any other wish?

R - I have heard, that patient gets the transportation charges. But it’s not available there. I have gone there 4-5 times. I go there by my own money.

I - Who gives you that money?

R - My father gives it, if he has, or he takes from someone else.

I - Now when you have HIV, T.B. and Kala-azar, what do you want to do regarding work?

R - I don’t wish to do anything.

I - Until when, you won’t do anything?

R - Until my body gets fine. Nobody lets me do work too.

I - Has your weight increased?

R - It has improved a lot.

I - How much was it earlier?

R - It was 49 kg

- Right now it is 60 kg

I - How much was it when you were fine?

R - 84 kg.

I - From 84 kg, it reached 49 kg?

R - No, from 84 kg, it reached 62 kg, then I got admitted here for treatment, then it reached 49 kg.

I - So, it came down to 49 kg from 62 kg.

R - Yes, in [redacted] it was 64-65 kg. When I came here, my weight decreased owing to tension due to my disease.

I - Right now how much do you weight?

R - Right now, I have reached 62 kg.

I - You wish to tell anything else about yourself.

R - What else can I say

I - Thank You
