# Supplementary material for: Quality of life perceptions amongst patients co-infected with Visceral Leishmaniasis and HIV: A qualitative study from Bihar, India
Source: PLoS One. 2020 Feb 10;15(2):e0227911. doi: 10.1371/journal.pone.0227911 (PMC7010301; doi:10.1371/journal.pone.0227911)
Supplement: S3 File — (ZIP) [file pone.0227911.s003.zip › Transcripts/Patient 22 Male Age 47.docx]

**Patient – 22, Age – 47, Male**

I - Tell something about yourself. Like how you came here for the first time, what do you do?

R - I have been practicing medicine in village since 1995. I also dispense medicine.

I - Great! Since when?

R - Since 1990, for 5 years in [redacted]. I am a good doctor, then started my own business. For God´s sake, I asked him…I am not on wrong track. I have children of 16-17 years age. Probably I got infected from the patient.

I - Ok!

R - Patient does not tell if he has any disease or not. Even if he dies, I have to suture blood and all as well. This happened to me.

I - Ok!

R - Kala Azar happened, so I used to go…There is Dr. [redacted]. I went to them. I used to give him 30 patients in a month. Dr. Said. “You have no disease, you are kidding,” it happened to me in jest. LC, Levofloxacin, were prescribed, for 5 days & Dr. said investigation will be done after 5 days. Then CBC was done, it was normal. [redacted] said blood at 3:00 AM will be taken. It came positive for Kala-Azar. I was flabbergasted. When saline was being infused for Kala-Azar treatment, a thought came to mind that since I had been feeling extremely fatigued I should get a HIV test done. My doctor was also surprised. I spoke myself.

I - That you have HIV or not?

R - Yes. It was tested and within 10 minutes, under microscope. The doctor was also surprised. My weakness had increased, I had 8-9 g blood (Haemoglobin) l and cough too had increased. All doctors were treating for typhoid with B.Coli and antibiotics. The fever that I had since the 24^th^, what do I tell you about it? The medicine started on 24^th^ and ended on 8^th^ April. I was tired, I felt I couldn’t feel my own spleen. Then I said leave it, Doctor, and then I left treatment. I went aside [stopped treatment], and whatever had to happen happened…even then I don’t have any regret- okay it happened- man is grown up, but still commits mistakes. He does research, he was doing research on me- you also do research- I told [redacted] also that you disturbed me for 40 days for no reason [*patient was frustrated at having been used as a guinea pig for research, kept longer for no reason*]. Dr. [redacted] told me that I would be kept here only for 1 day, as I’m a very busy person, but these people [in [redacted]], to make their attendance… [*frustrated at being kept so* long]. I feel very bad… I would have started a fight if I hadn’t been discharged today also.

I - When have you been discharged from [redacted]?

R - Now, today.

I - You have been here for 30 days or how much it is?

R - Calculate it, I have been here since 17 of the month.

- The doctors asked me why it took me this long to come here. I said that the Kala Azar treatment itself takes 28 days.

- They are fooling us because there is no cure for HIV the medicine that will be found there will be found here also…………Treatment is to restrict the disease it like shooting arrows in the dark, If get to know the disease correctly like T.B. which if caught is completely cure.

- This is my thought that to destroy India………these disease (HIV) have been made.

I - Who made this?

R - Whoever has made, some company has made. It is okay if it affects you when you are older, have had kids. Yesterday I saw an 18 year old testing positive (for HIV). I have asked my wife to stay away from me.

I - Do your wife know you have HIV?

R - Yes, test was done on that day, in front of her. So she knows. My children don’t know about it though.

I - What she said when she heard about it?

R - What can be done, take the medication if it’s all well with medication when there will be scientific research to stop it will stop.

I - Do you know how you did it happen?

R - I know this……. It has happened with infection.

I - Infection? What do you mean? How?

R - It has happened due to blood, during giving stiches to someone…………. I didn’t do wrong.

I - Ok, Tell what did you feel from starting? And when did it all happened?

R - I told……na…… I got fever….. I got this spot/lesion, took medication form homeopathic but didn’t knew the disease, he was a good doctor. My uncle told me that there is no use trying to get homeopathic treatment, it won’t be of any use.

- I am in medical line……..in disease this came out to be.

I - Hmm.

R - After this I said is that………. I went to [redacted] my nephew has his house beside [redacted], there was a function there I slept for some time in the outside, Got lot of mosquitoes bites and I got fever but In my bag I always carry antibiotic and took ‘NICE’ and ‘LEVOCETRIZINE’, I am saying this your perfectly, took the medication then……

I - How much back did this instance happened?

R - Before Holi

I - Ok Holi, few day?

R - Yes

I - First where did you go? Homeopathy?

R - Haan

I - Then to which doctor you went. Private or where?

R - Private doctor aa……..ku………In [redacted] hospital I went to get treatment.

I - Is it Government hospital?

R - Yes. Government hospital?

I - So, You went to Government hospital after Homeopathy?

R - Yes, there only ointment and other he wrote all these medication.

I - So, Till this time did you know that you had Kala-Azar or not?

R - This time I already got to know…….ahh…….. on 16^th^.

I - No..No when you went to Government hospital. Did you know that you have got Kala-Azar or HIV?

R - No

I - You didn’t know

R - No

I - There did they got any test done?

R - No..No test was done

I - What they used to say?

R - Oh….these people.. when I used to go and ask to see me….. they used to say take these medications…. use this medicine upto 2 months the wasted my time..

- I used to think.. it gets relieved for some time….then happen again… I thought it’s not good, it was time forest….. I think that 7-8 months ago….. ‘Saala’ this infection of HIV would have got in… slowly thereafter

I - Ok.. After Governemnt hospital where did you go?

R - Nowhere…the hospital he had said, I went there…I went to [redacted] hospital and then went to see a private doctor, a physician.

I - Ya.. Ya..

R - Physician also gave ointment and all…. of about Rs. 5000-6000, used it….. some said use milk, ghee and other…. ELTIMORE…., ELTI LOTION.. (BRAND NAME), all (smiles)

I - But they didn’t got to diagnose you of HIV or Kala-Azar?

R - Yes

I - Did they diagnose or not?

R - They couldn’t diagnose.

I - Then where did you go, after that (Private hospital)

R - After Private hospital……nothing……this……this was stated.

I - Who told you to come to [redacted]?

R - I told ….na…. Dr. [redacted] diagnosed me of Kala-Azar……… I went to [redacted], it’s a district hospital of Kala-Azar, I went there and he put saline to diagnose Kala Azar.

I - So You went first to homeopathy then Government hospital the Private hospital then to [redacted]?

R - I went to [redacted] where when they diagnosed that spleen in enlarged and Kala_Azar is diagnosed.

I - This was done in [redacted]? Whee did this happen? The diagnosis of Kala-Azar?

R - No one diagnosed Kala-Azar…. I said na they were “in the dark” (idiom) tested of fever, Kala-Azar…… when we go down there: a doctor sits there I asked to get a HIV test also.

I - All these happened in [redacted]? Is this a Government hospital?

R - Nooo……….. It’s Government Yes.

I - Then What did they say.

R - We have one day…….. one year ago…. Admitted one of our patient in there He also had Kala-Azar and HIV…….. I didn’t aksed anyone directly that’s why I went there I will not say wrong. I myself had got patients admitted there because it’s a seen place.

I - That’s why you asked to get it tested?

R - Yes, because after having fever, I got skin lesions, I was doubting that why is this happening (‘Saala’)

I - Hmm….

R - I was worried more of infection of this, (skin lesion) than fever.

I - What do you mean by this? The rashes (Skin lesion)?

R - Yes

I - You were more worried of rashes? Why such?

R - I have never had itchy rashes here… sitting somewhere & if it itches like this anywhere…… it does not seem good.

I - So, the lesion rashes on body were more problematic for year?

R - Yes

I - What else did you have?

R - Nothing

I - Fever and this?

R - Only these.

I - How was your mental status?

R - It was very good…. We 2 or 3 people used to come to Patna on motorcycle, had no tension, used to travel to [redacted], had no tension. It is only because of the disease.

I - Ok when you got to know about your disease, How did you feel?

- Mentally what did you go through?

R - Felt a big shock, but what can be done, whatever has to happen will happen; In this world many die of accidents……… (Pauses)………….

- Sir, I want to say that whatever has to happen will happen, when medicine is there to control the disease, I will take medicines.

I - What did you expect form life earlier? What do you think should be presented to live a good quality life?

R - It very important to have wish….. who doesn’t have a wish……. Wishes are infinite.

I - So, What were your wishes?

R - I had thought of having house in a city………… we are in village…. We have buried lands in bazaar….. wish was…… I had 1 son only and 2 girls but now dreams are shattered……God now much days I will go on like this on medication…… Sir whatever you say I will do… what else can I say.

I - What was told to you at [redacted]? To come to [redacted]?

R - They said to go to [redacted] at RM….eh…

I - [redacted]……Yes…….Yes

R - Hmm, So I said that I have accompanied 1 or 2 patient to [redacted], I don’t need the address, I can go on my own.

I - They themselves told to come to [redacted]?

R - They had told……They became anxious (Name) [redacted] Sir was not there, he had an accident…… I went to consult him at his house; There was no staff, there negligency is present….. Doctor need to remain for 8 hours…..; but don’t see patient, system is loose, someone goes here some there. I thought one day told a (Name) [redacted] Madam to increase 1-2 doctors. Madam used to sit at my medicine shop she is a civil surgeon…. We have good relation with her.

- Sir I will tell about my life…. That day also I was told undress my pant. I told that see whatever research you people are doing….. do it… I also know that you have called me by telling that of treatment of HIV. But HIV don’t have any cure, no any medicine.

I - Medicine is there but there is no permanent treatment means you have to take medicines daily But there is one thing if you take medicine daily on regular basis then the days you were to live then you will live some…….there is nothing that you have less days…..

R - Yes, I will take medicines.

I - There’s nothing else, not other problem, you will live the same.

R - I say that….. see.. (hiding his grief behind smile) (name) my wife that.. I am a very sentimental man……if I leave a work… say medicines….I will not leave it.. because someone has made it to stop it (the disease); But say that to stop me it not much bad I have children with God’s grace but for the harm to others........population of around 1 crore will reach by next year with this disease (HIV)

I - Ok, Tell me more what else were you going through mentally?

R - Nothing

I - Anything that you won’t be able to do after this?

R - No……No…….nothing my wife only said sit at a distance; she is also educated; said sitting at a distance you can see patients, the price of treatment and maintenance (of his disease) can be earned.

I - Means working by sitting at one place? So Did it had any other effect on work?

R - Work and all…… it can be taken that from 1-1.5 month I have not been to the (hospital/medicine shop ??) When I will go it will take 8-10 days to settle.

I - But Do you feel that you can enter into the same work?

R - Yes…. Yes…. It will not have problem

I - So, there won’t be any problem in earning?

R - No, there won’t be a problem.

I - As you were saying earlier of getting built a house. Will you be able to do that after this?

R - It can happen…. If God is willing then I may take a housing loan and complete the work too.

I - Every month you have to come to take medicine. Do you face any problems or You will face any?

R - Now…….Now…… it only starting of treatment.

I - How much distant will it before you?

R - 55-60 Km

I - So, How you come? Bike? U said?

R - Yes…… I get some work at regular intervals in [redacted] if there is some patient…… then we 2 people are there, then we come here travelling alone is not good, always travel with someone; if some accident happen, one is left he can inform; City is so big if one gets an accident alone, no one is there to ask.

I - Do any other person, neighbour or relative know about this disease?

R - No

I - Do you want to tell anyone?

R - Why would I, Regarding HIV Primary health centre tells everyone…. For family planning any operation is done. My wife is a ASHA on my area. She goes from home to home and (smiles to hide her shyness and grief) Firstly in family planning she tell everyone and is conducted by HIV hospital [redacted].

I - Ok....Ok....

R - We both are from medicals.

I - Did medical?

R - These…. Small work…… we don’t have degree.

I - These small thing (Private/Quacks ??)

R - These small we earn Rs. 2100, 200, 400.

I - Something else you want to say? What else difference you got in your life?

R - No….. Nothing for now… I will go home. Take care of food & health & maintenance I told (name) abc to tell me the antibiotics like if there is cough will I take cough syrup or not.

I - Since when are you having cough?

R - Cough has been there… but since treatment have started here…… within 5 days cough got relieved, no lie…. Continued I don’t had cough before…… but since the spleen has enlarged and fever of 102^o^F came I have get anxiety. In Kala Azar I also that fever is continuous.

I - So what do you fear more Kala-Azar or HIV?

R - From HIV?

I - Why such?

R - Because cure has not been found of HIV. Cure of Kala-azar & T.B. has been found.

I - You don’t have T.B.

R - No

I - Have you been tested (for T.B.)?

R - Yes, all repots are there, you can see in my file.

I - They are negative?

R - Yes, Negative.

- After Kala-azar, I know of HIV, For me, It’s a great set back। How I got this, I do not know. When I go back (to work), I will get tens of patient tested for HIV first. If one gives stiches, blood is there, disease due to the virus. True is the disease is due to virus otherwise. It is due to inappropriate sexual encounters also. If it is due to inappropriate sexual encounters like [redacted] who lived with thousands of women, is not having a disease. It is maybe common in cities – like in [redacted]. But it is unheard of in the rural areas where I live. Maybe those who work in cities and come back to the village bring it with them.

I - Have you had sexual relations with other people (other than your wife)?

R - No…. Never

I - Never…… Never it’s ok

R - If it remained the our life gets in children could get affected.

I - Does your wife also have it (HIV) or any child?

R - Child does not have

I - Hmm your wife does have

- Is there any difference in the behaviour of your wife? Or any difference I the relation between you two?

R - No any difference, she has more tension than me………. I said if you are having more tension then give me 2 pills of sulfas (a poison) and I will take.

I - Did she worked or does she work?

R - Yes she does

I - As ASHA?

R - As ASHA but not at their house, whoever needs can come, but not like going from home to home…. We are like… as born in [redacted] (Saala) live with royalty if there is work whether it is of Rs. 200 or 500. She also have good nature there’s no problem.

I - If I ask that to live a good live what all is needed in world?

R - See…. We should know our limit if I say I want to travel by plane then if God wants.. whatever is there a man only does wishes are infinite but we are what is can have 2-4 wheelers in 4-10 years.

I - Tell me about your life what would you like?

R - A four wheeler car and will be all. Getting more rich is also a danger four wheeler car to come & go anywhere.

I - Another thing? Home?

R - For home I said na that housing loan, home is not that tension.

I - And Salary?

R - Salary say…… or not I am not be able to work for salary true its like the disease I have got, if you ask for laborious work to go here & there Motorcycle is at my house but my wife will now allow me to go , she will say take a man with you so if I does this work who else will I pay.

I - Is health necessary for good life?

R - Good who does not want, Health is wealth, but when…..

I - When you came here for treatment, what you think could be improved in your treatment?

R - System, See I have got Kala-azar disease, Kala-azar is treated fever with grace of God got treated. Spleen also got right, Everyone made me anxious that (Platina) wanted to say platelets has gone down to 51000, a person saw my report and saw that its 51000, nothing to be anxious.

- On the name of Kala-azar, only 2 people know this, I have not told others sir, 2 people are my guardians I said that if something happens you have see the place. This is the sad truth of my life.

- Continued

- Now what would I do of earning?

I - What would you do of earning?

R - Whatever has to happen……. Normally I will earn because earning form the running work cannot be done by me now, At most 10 km or 20 km by motorcycle if I will energy then.

I - Ok….Ok…. what difference have you got in the health due to this disease?

R - Until now, now difference.

I - No difference?

R - I kg weight has decreased.

I - Any problem in sleep?

R - No problem.

I - Has there been any impact on your future plans or family plans owing to your disease?

R - No any difference

I - Mentally are you fit? Or any problem?

R - No any tension. I have two daughters… their marriage is yet to be taken care of.

- There is little tension, marriage of a girl whenever I want I can do she is not of age so how could I say…….. (Pause)

- They are doing research on me at the hospital. I cannot do any work while I am taking the medicines. They have made me jobless.

I - They? Who has done this?

R - The staff here. They told me to be part of a study that is happening. It okay it happens I have stayed for 40-45 days 17 to 17 and this 24-25 has gone; I was told that doctor called me. They keep taking blood tests. Even Paras (a private hospital) in Patna won’t treat patients like this.

I - How did the staff at [redacted] behave with you?

R - I don’t have any complains about them. They told me that the doctor was busy.

I - You came here in March 17?

R - Yes; they said this doctor is very capable. I would be treated well. But here they took 12-10 days in testing only I said that spleen is punctured ok its rejected I understand that there are tests of whole body which can be done in 2 days.

I - Hmm.

R - It seems like everyone here is an executioner (“Jallad”). I have seen patients in more serious condition on O_2_ or ventilator their test are processed in 24 hours. What do you think Sir? Is that so or not?

I - It all different, your disease is complicated like you have HIV and Kala-azar both in this there is problem.

R - It ok, then why should I wait?

I- We cannot be sure what medicine to give without doing a test, right? So we have to see that.

R- Okay. Now what has already happened…

I: Not like they’ve just kept you for no reason. But uh, tell me one thing- when you come to a hospital for treatment, what things do you find important? What do you think? What things should be there for treatment in a hospital?

R: Starting with stretchers. You need a bed for the patient, as a facility. Whether you get food or not, the patient eats food somehow in today’s date. But they are giving it here- that is very good. On one or two days the salt was more in the food and it was not edible. I complained about this to the doctor, and he said okay I’ll talk about it at the meeting. I didn’t even eat food for 20 days. I just drank Maaza and Sattu (gram flour)- I did not used to feel hungry.

I: When- during illness or during treatment?

R: Yes illness, this uh…during illness. I did not eat for 20 days.

I: You did not feel hungry at all? How are you now?

R: Now I am okay. I am 90% okay.

I: And what do your kids think? With what’s happening in your body and…

R: They don’t know anything.

I: They don’t know?

R: They are too young, they are studying right now.

I: Any other problem?

R: No problem.

I: Okay. What else would you like going forward? And has this changed because of the illness?

R: My haemoglobin is low. When I came here my weight was 60 kg, now it is 59 kg. Since coming here, her [wife’s] weight increased by four kg and mine decreased by 1 kg. I started my medicine. When I go back maybe I will be alright for 10 months or so.

I: Anything else? Anything else that could have been different in treatment?

R: No no whatever has happened has been fine- since I am OK still…

I: And why did you decide to go to private setup instead of government setup?

R: Not all treatment happens in government hospitals. The wards are not maintained well. The government hospitals are not maintained well. TB is more infectious – it is there in the air.

I: Okay, thank you very much.
