# Supplementary material for: Quality of life perceptions amongst patients co-infected with Visceral Leishmaniasis and HIV: A qualitative study from Bihar, India
Source: PLoS One. 2020 Feb 10;15(2):e0227911. doi: 10.1371/journal.pone.0227911 (PMC7010301; doi:10.1371/journal.pone.0227911)
Supplement: S3 File — (ZIP) [file pone.0227911.s003.zip › Transcripts/Patient 23 Female Age 45.docx]

**Patient – 23, Age – 45, Female**

I - Who all are there with you?

R - There were two daughters and one son

I - Why where? Where is he now?

R - He lives outside

I - Two daughters?

R - They reside at their in-laws place

I - If they both are married, who resides at your place.

R - No one….. I reside alone

I - Alone……. Your husband?

R - Husband is no more…….. he died long time back

I - 13 years back??.......... Ok…… so what do you do?

R - I feed school children

I - Where……. Aaganwadi?

R - No……… In middle school………

I - Ok…….. In middle school…. the mid-day meal……..

R - Yeah…… I work there……

I - Do you cook food? Or help in feeding children?

R - No……. I help in feeding cooked food to children.

I - How much money do you get?

R - 1200 rupees/month.

I - In one month?

R - Yeah….. In one mouth

I - So……… Is this money sufficient for you?

R - No…. but somewhat ok……

I - Any other source of income? And do you eat one meal while helping the children feed??

R - Yeah…… I do……..

I - So….. One time meal you get from your employment?

R - Yeah

I - Any other help from anyone?

R - No………no help…..

I - What about your son?

R - Son??.......... He sends some money….. my parents help me…. (pointing her fingers towards her mother who was accompanying her)

I - Is she your mother?

R - Yeah…… she is my mother……..

I - Does she reside with you?

R - ……No…….. when I get ill……… she comes to me.

I - ………So……. tell me in detail about your illness? When were you perfectly fine.

R - I had three episodes of Kala-azar…….. including this.

I - When it happened first?

R - First……….(enquiring from her mother)…… thinking…….. How many years back……….. (Then her mother replied – (R_2_)

R_2_ - I don’t remember correctly…….first episode of Kala-azar was treated……then after one year she had another episode………. then got some relief after treatment………Then after two years third episode……..so overall it was approximately 5 years.

I - So…….. you have been suffering for the illness for the past 5 years……….. So what used to happen before 5 years?? How the disease was started?? What symptom?

R - I was having altered bowel habit…….. Diarrhoea……

I - Diarrhoea………?? And fever?

R - Yeah fever………..and vomiting……

I - Diarrhoea means??

R - Having passage of many stools in a day…….nothing was being digested……..

I - ……..Ok……….food was not being digested and do you have any complaint of night sweats?

R - Yeah….

I - Any other complaint like……weight loss……..

- How was your appetite?

R - After treatment……. It is fine…..

I - Before treatment

R - No…… no there was a loss of appetite

I - How was your sleep?

R - Sleep is fine

I - Before your illness?

R - Earlier I would not get sleep.

I - For how many hours you use to sleep…..?

R - I used to sleep continuously only for 1-2 hour

R_2_ - She was not having food… how will she sleep?......... She was restless for the whole night. We were not aware that she is having Kala-azar…….we went to a local quack for the relief of symptoms where the quack enquired about Kala- azar episode……Then I said that she had two episodes of Kala-azar…..we want to get her liver cut?

I - What do you mean by that?

R - Liver

I - I mean what the quack will do with liver?

R - Quacks cuts liver

I - Any wound in that area?

- What do you mean by liver?

R - In Kala-azar………Liver gets enlarged

I - Ok

R_2_ - And this ….. a problem in digestion of food.

I - So you get is cut?

R - Yeah..

I - Who does that?

R_2_ - She is made to lie down and ask to ingest rice flakes……..

I - So this reduces liver??

R - Yeah…… It does so…

I - When you had first episode of Kala-azar, where did you go?

R - I went to Jaipur

I - Jaipur?

R - Yeah

I - Private or government?

R - It was private…but he couldn’t diagnose it.

I - How may days you spend in Jaipur?

R - In Jaipur, the doctor diagnosed it as Malaria…….. the treatment continued for a month.

I - How much money was spend on treatment?

R - Too much money

I - Still………. Approximately?

R - Approximately – Rs. 20 thousand……… I got this aid from government to construct house………all was spent on treatment.

I - From where you got this Rs. 20 thousand aid?

R - From government…….

I - Ok

R - Yeah……. All money spent on treatment (repeating he same)

I - So you got this money for?

R - For making house

I - Ok……..Did you get any other money from government?? For Kala-azar?

R - No……..

I - Then where did you go after Jaipur?

R - I had a fight in [redacted] with the doctor…….. Then I went to Dr. [redacted]………he got same tests done…… he took out some blood and performed the test and confirmed that I have been suffering from Kala azar…..

I - There??......

R - Yeah……..at [redacted]

I - Any other diagnosis was told to you apart from Kala-azar?

R_2_ - No……There he didn’t tell anything else…… Then we went to [redacted] hospital…….she was admitted there and stayed there for one month and got treatment.

I - [redacted]………Government or Private?

R - Government………and the treatment was completed there

I - Did you spend money in government hospital [redacted]?

R - No………….Some money was spent on food etc….

I - Tell me in detail?...........

R_2_ - She was recovered from Kala-azar from [redacted]hospital…….then again one episode of Kala-azar….second time it was again told that it is Kala-azar……. there is no need of injection…..tablets needs to be taken for this…….people suggested that we should go to [redacted]……then she got tested there……and this disease was diagnosed??

I - What was told to you in [redacted]?

R - He didn’t tell anything……only wrote the diagnosis on a piece of paper and asked to go to [redacted] hospital…….5-7 days were spent there………..and afterwards the medication was started………for one month two months…..

I - Which disease was told to you?

R - Thinking……….. pause……….he told……. about disease

I - Was it HIV?

R - Yeah…… He said HIV………..

I - Have you ever heard about this disease or do you know about HIV?

R - No……….

I - So for HIV…….. for how long you have been taking medicines.

R - For last 5 years.

I - 5 years??

R - Yeah…….. He gave me two bottles of medicines.

I - So………You have been told about HIV first or Kala-azar first.

R - I was told about Kala-azar first

I - Then HIV?

R - Yeah…………

I - When you came to know about HIV was your mother was with you?

R - Yeah….. She is the one who accompanies me everywhere.

I - When you were told about HIV in [redacted]……How did your feel?

R - After taking medicine…… Kala-azar was Ok……..

I - Have you disclosed your disease status to anyone………?

R - No…….No………..

I - Why?

R - Doctor asked me not to tell it to anyone…….medicine will be continued……

I - That means your mother knows about your disease……..your father??

R - No….. He doesn’t know

I - That you have HIV……… Is it known to your father?

R - No…..No…… No one knows……..

I - In neighbourhood?

R - Oho………No….

I - Your son or daughter?

R - Ohho….. all No………

I - No one knows?

R - Yeah…….. daughter and all doesn’t know

I - So……. You have not told them?

R - I tell them that I take one medicine from [redacted] and one from Patna for Kala azar.

I - Why do you think that you should not tell them?

R - All start getting nervous…………disease is Bad………assumes dirty……..

I - Dirty……..assumes dirty?

R - Yeah

I - Do you think in the similar fashion, if you think of other?

R - No………..I don’t think this way

I - But the way you are telling that assumes dirty…… why assumes dirty?.........

- In what context?

R - People say………

I - What people say……..please tell in detail……

R - Disease is bad…….. बल is bad

I - बल means?

R - Disease is bad

I - Other disease are also bad………..no disease is good

R - Yeah only

I - Why only HIV is such a bad disease about which we don’t want to tell to anyone……. What do you think about this…Why people will think like this?

R - Yeah………

I - Will you please tell about this?

R - I am poor Beta………..I have son…….Daughter-in-Law

I - Is there someone who has the same disease in your family or neighbourhood?

R - No………

I - Here at [redacted]….

R - No……….

I - You don’t know about others.. as no one knows about you?

R - No……….Not in my colony

I - No?

R - Only I have this

I - Only you?

R - Yeah

I - Anyone with Kala-azar?

R - Kala-azar too not there

I - Kala-azar too not there…….. You told that your husband died…..How many years back he died?

R - 12-13 years back

I - He was having what? Due to what reason he died?

R - Due to disease……..it was not known

I - Which disease?

R - Disease was not revealed………got treatment for Kala-azar himself treated for everything got himself treated for T.B……. treated for everything

I - From where you got the treatment?

R - From [redacted]. I was tired of [redacted], [redacted] [her husband was admitted in [redacted]]…he was admitted in hospital for 2 months. When he came back from there, everyone told us to go to [redacted]. We were planning to go to [redacted], but in the meantime, he passed away.

I - At that time…….when he was tested….?

- Did you got any tests done?

R - No……

I - No………at that time you were not tested?

R - No………… I was not aware regarding the medicines.

I - At that time of your husband?

R - Yes

I - Did he also have HIV?

R - Yes, I guess

I - Did he tell you?

R - Just like me he also suffered in the same way. So I guessed.

I - But he didn’t tell you?

R - No

I - But you are guessing on the fact that he suffered in the same way as you are suffering.

R - Yes

I - Where did he work?

R - Out of village/town

I - What did he do?

R - Phuldari (फूलदारी)

I - What is Phuldari?

R - Loading and unloading of sacks of grain.

I - Was he a truck driver?

R - No, No

I - Did he load bus?

R - Yes loading & unloading from bus

I - Ok, How was your last year?

R - Very unfavourable. It was doing today & tomorrow………I will die now…… I will die tomorrow……… I carried to Patna in my arms.

- I was brought to Patna. Investigations was done for 10 days after that treatment was started. Then I got some relief. After one month of treatment the disease was in control and I was discharged from hospital. Second I was kept in hospital for 2 months. Then the disease was controlled.

I - Tell me one thing, what all thing are needed by a person to lie a good life?

R - Pankiller medicines

I - Anything else?

R - Food

I - Can you tell me more about it?

R - No don’t talk about that. I have left all those things.

I - No, No, you told me to live a good life things like medicine, food are needed. Anything else about it?

R - What other things?

I - House?

R - I don’t own a house. I have one son, I am worried about the house.

I - Do you bother about the house? What is importance of money to live a good life?

R - Everyone need a house. I have a son, how the thing will work?

I - Do your son do a job?

R - No, he do not do any work. Just wanders here and there (He is unmarried).

I - Do you want him to get married?

R - I do not have a home. So I do not think about it.

I - You do not own a house that why you son is not getting married?

R - Yes, That is the reason

I - Why is it?

R - The girl’s family look for these things………….I have helped him in completion of his studies……….But seeing the broken house no one will come………That’s why I have kept him with myself.

I - Ok

R - Rs. 10,000/- will be needed to repair my hut. From where I will get that.

I - How the disease has affected your life? Tell me about it.

R - I got the disease after death of my husband.

I - Did it happen just after his death?

R - Yes, I got the disease only after his death. There was nothing before his death.

I - How is the social environment where you live?

R - It is good

I - How are the people?

R - They are good

I - Do they talk with you?

R - Yes, They all do

I - Do they have friends in neighbours?

R - Yes

I - After getting this disease you remain ill must most of time. Is there any change in their behaviour after knowing it?

R - No sir

I - Do they talk to you in the same manner as previously they do?

R - Yes

I - After the death of your husband and getting this disease. Did you thought that your life was waster? What the point in continuing this life?

R - After the death of my husband, I was tensed about my children. They were younger. I had no idea at that time how would I earn? How will I feed my children? I did not have my house. After that I got ill.

I - Had you ever thought of doing?

R - No

I - How did you marry your daughter? Did they get married before or after death of your husband?

R - All were married after he died.

I - Done by maternal grandparents?

R - Yes

I - Do your mother and father support you?

R - Yes

I - Do they support you till now?

R - Yes, After death what will happen only the god knows.

I - Why do you not live with them?

- (To mother) Do she live with you?

R - No, I live in a hut

I - (To mother) where she live?

R - She live in her house. She comes to use once in a month.

I - How far is your house form your parent’s house?

R - Very far

I - How much time it take to travel?

R - It takes 1.5 hours.

I - How often do you meet with your parents?

R - Whenever I want I go to their house. Since I had to come here today, I came to her [daughter’s] house yesterday only. I came today, and my mother will stay with me for 1-2 days, and then she will go back to her own house. I will call my daughter- she will stay with me for 1-2 months.

I - Your daughter?

R - Yes

I - Do she know about your disease?

R - No, she don’t know

I - When you got ill, did it affect your daily work like feeding your children?

R - No

I - Did you feed your children even during illness?

R - When I am very sick, my mother comes to my house or my daughter comes.

I - Ok, when you become ill any of your mother or daughter helps you. Do you get Rs. 1200/- per month?

R - Yes

I - For how much duration do you work in this field?

R - Approximately 1 year

I - 1 year. What did you do before it?

R - Before also I used to do the same work but I used to get 50 paisa for one child.

I - Did you work anywhere before it?

R - Yes. I do this work for last 5 years

I - After the death your husband?

R - Yes

- He did not allow me to go out of house.

I - Did he not allow to go out?

R - Yes

I - You did not work when he was alive?

R - He (husband) never even provided me wood [fuel] for cooking food.

I - Meaning?

R - Now I get wood or fuel for cooking food…and for the past one year, I have gas [LPG cylinder] for cooking food [provided by the school].

I - Do your illness has affected your work?

R - No

I - Do you work in the same manner?

R - Yes

I - Can you tell me about your treatment? How do you feel?

R - It feels good

I - Do everyone behave you in a good manner?

R - Everything is fine

I - Tell me about the treatment done here.

R - I feel good. They have treatment me very well

I - Do you want any change here? Anything you wished like if this was available then it would have been better.

R - She comes to me and wish me. Medicines are given. Everything is right.

I - You do not see any problem in it.

R - No

I - All things are good.

R - Yes

I - Tell me, what do you think of your life? IN the situation of having this disease what do you think?

R - I have a son so I want to buy some land for him. I do not have a house so wish to have one.

I - So you want house and land?

R - Yes

I - Why?

R - Source of money plus having a home is must. Can’t be bought from my income. I have a piece of land worth making 2 rooms.

I - Ok……….So…….land is sufficient for making 2 rooms?

R - Yes

I - So…………What are you thinking about it?

R - I tell him to earn and get married (to his son)

I - He doesn’t follow you?

R - He says I don’t have a house.

I - What is he doing?

R - He doesn’t do any work. He goes out and comes back………I got seriously ill and he came back.

I - He went to work outside

R - Yes

I - So you called him back because of your illness?

R - Yes, Even he left exam of 10^th^ standard, started working and used to send money.

I - So, he have not passed 10^th^ standard?

R - No

I - Why?

R - He said he has only one mother and wanted to keep finically. So he went for earning.

I - Where he went?

R - Bangalore.

I - What he did?

R - Fitting the tiles.

I - lifting (stone lifting)

R - Yes, these floor lifting item, tiles fitting

I - Yes means tiles

R - Yes

- Escaped once, had a good future, so I told him to go do her grandmother place. He is there past 5-6 months

I - Why denied?

R - Risk of getting out in legs. Once escaped on accident had minor cut.

I - Where is your son?

R - Grandmother house (Maternal)

I - What he does for living?

R - Nothing

I - What he does all day long?

R - Stay at home

I - Neither he studies or work?

R - No

I - His age

R - 18

I - Would you like to tell more about your disease?

R - No, not more

I - How do you feel about your disease?

R - I am okay

I - Who goes to take medicine?

R - Myself. Goes to [redacted] by own and if in Patna, my mother accompanying me sometime.

I - Any services from government regarding this disease?

R - Not for HIV, only getting medicine

I - So you get medicine right.

R - Received only medicine.

R - Used to get medicine for 2 months

I - You were given medicine for 2 months at one time.

R - Yes

I - So you used to go every 2 months

R - Yes

I - What were your expenses in up and down?

R - Approx. Rs.100 excluding food

I - Were you satisfied by medical service provided to your place

R - Did not consulted doctor

I - Didn’t went there

R - No

I - Why

R - None was there do accompany

I - Not consulted even in private

R - No

I - Okay

I - So you were told not to tell about this disease, why?

R - Even I don’t know

I - You were saying something, Don’t you?

R - Reputation in market regarding disease is not healthy

I - What will happen if you tell about it?

R - Nothing

I - No you were saying something that they won’t lot let you work?

R - Yes

I - If working anywhere also?

R - Every people will do the same

I - Example if you are feeding food to student in school will they let you work?

R - No

I - Okay thank you very much for your time.
