# Supplementary material for: Quality of life perceptions amongst patients co-infected with Visceral Leishmaniasis and HIV: A qualitative study from Bihar, India
Source: PLoS One. 2020 Feb 10;15(2):e0227911. doi: 10.1371/journal.pone.0227911 (PMC7010301; doi:10.1371/journal.pone.0227911)
Supplement: S3 File — (ZIP) [file pone.0227911.s003.zip › Transcripts/Patient 24 Male Age 35.docx]

**Patient – 24 Age – 35 Male**

I - What do you do?

R - Driver.

I - What do you drive?

R - Tractor.

I - Do you drive any other vehicle?

R - No.

I - Who all are in your Family?

R - I have 5 brothers.

I - You have 5 brothers, anyone else?

R - Wife.

I - Ok! Wife

Do all brother’s families live together?

R - No they all live separately.

I - And kitchen?

R - All have separate individual kitchens.

I - Who else lives in your house other than you and your wife?

R - My children

I - How many children do you have?

R - Four (4)

I - How many sons & daughters?

R - Three (3) sons and one (1) daughter

I - What is birth order of daughter?

R - 2^nd^ birth order

I - Have your children get married?

R - No, No

I - Has no one get married?

R - No

I - Okay, Tell me one thing, how much long back, you were completely fine? You had no problem how many years back?

R - Since many days I do not know, how did I get it?

I - From what time had you problem?

R - From 2 months

I - Only for 2 months? You had no problem before that?

R - For last 2-3 months.

I - Were you completely fine 3 months back?

R - Yes

I - Your work?

R - I used to do every work.

I - Driving Tractor?

R - Everything.

I - In which area did you drive tractor?

R - I used to plough fields.

I - Ok.. You plough in which district?

R - In [redacted] district.

I - [redacted]. What happened 3 months back, would you tell me in detail?

R - I first got cough, After that I got cold with fever. After cold & fever, I became dysphonic (breathless). Then medication started from [redacted].

I - For what medication started?

R - First blood was tested.

I - Okay

R - People said u got Kala Azar

I - Okay

R - They said it has increased. What is it called?

I - Is it the spleen?

R - Spleen. That has increased then we get it. They said go to [redacted]. So I went [redacted]. From [redacted] they said to go to Patna some investigation were done.

I - What investigations were done at [redacted]?

R - Blood investigations were done, sputum test was done. After that got report.

I - What was told in report to you? What did doctors tell?

R - Report said, it is T.B.

I - It is T.B. Any other disease?

R - No other disease was told.

I - Okay. Did not tell about any other thing?

R - No

I - Okay. After that what was done said to you?

R - To go to [redacted].

I - Who said this?

R - That [redacted] what is name?

I - [redacted]

R - [redacted] Jee

I - Yes

R - [redacted] ji said go to [redacted]. Will be cured at [redacted].

I - Okay

R - I came to [redacted] from there. Investigation started at [redacted].

I - So you were first told that it is Kala Azar. After coming to [redacted] you were told it is T.B. Apart from this you were not told about any other disease?

R - No

I - Then, When did you come to know about HIV?

R - That was also told.

I - Where, where was it told?

R - Told at [redacted].

I - Okay, Any test was done to your wife or children or anyone else’s?

R - It was not done there.

I - It was not done at [redacted]

R - It was done here, she had not get this.

I - Child??

R - Children were not.

I - Were children tested?

R - No, they were not.

I - They were not. So your wife done have this?

R - No

I - Okay. So when you were told in [redacted] that you have T.B., HIV, How did you feel at that time when you listened it for first time?

R - Felt very bad

I - Okay. What were you thinking at that time?

R - I was thinking how did I get it?

I - Okay.

R - It had never happened to me like this, suddenly how did I get it?

I - About this thing?

R - Started thinking, I lost my appetite.

I - What happened?

R - I lost appetite.

I - Okay, lost appetite.

R - It had never happened to me before.

I - Yes

R - So I became tense that how did it happen?

It happened for first time.

I - Okay, were you thinking something else at that time? Did you have feelings that your life is useless or what will happen now? You thought something like that?

R - No.

I - You did not think like that. So what were you thinking at that time?

R - I was thinking that I will be cured as I have come here.

I - So, you come to [redacted]?

R - Come to [redacted]. I was referred here from [redacted]. I thought I will get cured. Doctors will treat me properly.

I - So for how many days you were not healthy? From this disease?

R - 3 months passed.

I - 3 months passed. What were you told first of all?

R - About spring

I - Spleen?

R - Kala Azar

I - Kala Azar. What disease were you told after that?

R - I do not know that name.

I - HIV?

R - Yes

I - When were you told T.B.?

R - T.B. was told later on after coming here.

I - You were told here or In [redacted]?

R - Was told in [redacted]. I do not remember exactly.

I was not okay, I was completely like that.

I - Okay.

R - Condition was bad.

I - Condition was bad. But sputum was tested at [redacted]?

R - Yes.

I - When you heard this at that time, when you were telling about your weight that you had emaciated fully?

R - Yes.

I - What was your weight 2-3 months back?

R - In starting it was became 40 or 41 or 42 kgs.

I - Weight had decreased?

R - Yes.

I - Before that what was your weight, when you measured and you were healthy?

R - Earlier it was 55 kgs

I - It was 55 kgs. How much it decreased to from 55 kgs?

R - 42 kg.

I - 42 kg. And did it go below 42 kgs?

R - No.

I - Did not go. And now your weight??

R - It is increasing.

I - What it is now? Had you seen now?

R - It is 400 gram less than 50 kgs.

I - 49 kgs Okay. So when problem started, you got fever 3 months back then, First of all where did you go? Whom did you visit?

R - Visited [redacted].

I - Where in [redacted]? Visited private?

R - Private.

I - Or in government?

R - It is government (Asking someone).

I - Government. How much did you pay for appointment?

R - Hmm……………….. (Thinking).

I - Money for appointment was taken by doctor?

R - By doctor.

I - Is money paid for appointment in government?

R - He took. What can I do? He asked for, So I gave.

I - Was that [redacted] Hospital?

R - It was [redacted] Hospital.

I - Okay, What investigation was done your?

R - Blood, only Blood investigation was done.

I - How much money did you spend?

R - Rs. 1100/-

I - Rs. 1100/-. Where you went to in [redacted].

R - Yes.

I The [redacted] Hospital you are talking about, for how many days you continued going there for your treatment?

R - 2 days, 2-3 days like this.

I - Then after that, what did doctor says?

R - After that he said, Go to [redacted].

I - Why, Why did he ask to go to [redacted]?

R - Do not know.

I - Okay, Had you any improvement in your health?

R - Did not have improvement. Went to [redacted]. From [redacted], they referred here.

I - So, What did they (Doctor) tell that it is Kala Azar? Was it told?

R - Yes.

I - Did not tell about any other disease? What else was said to you?

R - Go to [redacted].

I - Go to [redacted]. In [redacted] to any Government or any Private??

R - In Government.

I - Go to Government. Okay. So after going for 3 days to [redacted] and spending Rs. 1100 as you are telling, any other amount of money was spent by you?

R - No.

I - No. What was done to you in [redacted]?

R - Only that treatment was done at [redacted].

I - Treatment of what?

R - Of sputum, Blood investigations.

I - Investigations were done?

R - Yes.

I - Investigations were done. Did you pay there also?

R - Paid. Little bit amount paid. I do not knew, about how much?

I - Okay, right. Were you given some medications there?

R - Did not get medications.

I - Did not get medications. What was said to you there?

R - Go to [redacted].

I - Go to [redacted], Okay. When you heard about your disease at that time, who was there with you?

When you went to [redacted]for your disease, at that time?

R - There was my wife.

I - Wife was there. So when you were told about your disease, who told you?

R - Doctor.

I - Okay. To whom did you tell about your disease?

R - No one.

I - To your wife?

R - Wife knows.

I - How does she knows?

R - I told, when doctor told she came to knew.

I - Okay.

R - She was with me at that time:

I - She was with you. What if she would not have been there with you? Then?

R - I would have kept it to me.

I - Then you would not have told?

R - No.

I - Okay. Why do you think so? Why do not you want to tell about this disease?

R - What is there to tell about to anyone.

I - Did you tell anyone about Kala Azar?

R - I told about Kala Azar.

I - You told. About T.B.?

R - I told.

I - Why do not you want to tell about HIV. Means what do you think about that? We just wanted to know this.

R - That little….

I - That What do people think about his matter?

R - Do not know.

I - If any one.

2^nd^ I - You were saying that little. What do you know, What you think about this?

R - I think right about it. I just get mistake a little (थोड़ा mistake कर जाता हूँ)

I - What you feel?

R - I do not get anything in mind.

I - No, If you listen about someone else who has HIV?

R - Yes, Then I also say it. What else.

I - What do you say?

R - I say that. While saying there is a little problem about it.

I - Means you say something wrong about it?

R - No. I am not able to say that talk (word).

I - Okay. So you do not say to anybody about it?

R - No.

I - If your would not have been there then?

R - I would have told at home.

I - Okay, Did you tell any of your child about HIV?

R - Sons know.

I - How did sons know?

R - I told them, by mobile.

I - By calling that you have HIV?

R - Yes, Do not tell anyone.

I - Okay. All your children, told to all three sons?

R - Yes.

I - How old is younger one?

R - 8-9 years old.

I - 8-9 years old?

R - Yes.

I - He would not have understand? He would have not been told?

R - They listen conversations.

I - To anyone in neighbourhood?

R - No.

I - Do you know anyone in neighbourhood who has such disease?

R - No.

I - In [redacted], wherever your [redacted] is, Do you know about anyone who is talking medications for such thing?

R - Yes, there is one person, He lives away from my home.

I - Okay, He is also talking medications. Did you know about him earlier?

R - Yes, From earlier.

I - Okay, But in your neighbourhood no one knows about you?

R - No, they do not know.

I - They do not know. The person who is taking medications, Does he know about you?

R - He is like my brother.

I - He is like your brother. Okay, Did you tell him?

R - Something like brothers in neighbourhood.

R - Yes.

I - So you told him who was already talking medications?

R - He knows.

I - Why did you tell him?

R - He came. He undergoes some investigations.

I - Yes.

R - In Patna

I - Yes, He also came?

R - Yes.

I - So you met here?

R - Yes.

I - Okay.

R - He accompanied me here.

I - He accompanied you. Then, as you are telling. After this disease did you notice any change in behaviour of your neighbours after you went home back?

R - No, Not till now.

I - No. Then talk to you as before?

R - I did not go home till now.

I - You did not go home, so what about when you went home from [redacted]?

R - After that when I went home, every family member started eating separately.

I - Okay.

R - After that I started eating food.

I - Everyone ate separately?

R - , ate separately in plates.

I - So, how did you eat earlier?

R - When I came from [redacted], then people told……..

I - Yes

R - You have a such disease.

I - Who told you?

R - Doctor, So I started getting separated food.

I - So, they said?

R - Yes.

I - who asked you to eat separate food?

R - Doctor Sahab

I - That your food?

R - Yes.

I - Okay, then

R - Everyone’s was cooked commonly. Only mine one was separate. Sons ate together.

I - Then?

R - After that it continued.

I - Okay, The medications, you are talking, you bring it every month?

R - Yes.

I - Or 2-3 months medications at once?

R - No, every month

I - Gets every month. How many tablets in one box?

R - 28 tablets.

I - Okay, When do you take?

R - At night.

I - Anything related to that?

R - Eats in morning.

I - From where do you get T.B. medications?

R - Like that where it gets.

I - Okay.

R - Now when I will be discharged then in [redacted]….

I - Okay, You have not been discharged till now?

R - It is first time today.

I - So you, when you came to know this, you told everyone to your family. But you tell me one thing that you get a good life, you had not disease or for that what things you think are needed??

R - …………………… Do not know (Cannot tell).

I - Like many people wants money,

R - Yes

I - Many wants Home, What do you think about it?

R - I have a house already.

I - You own your own house?

R - Made up of Karkat?

I - House is made up of Karkat. So do you think it is Okay??

R - No.

I - You want to make some house?

R - Want to make.

I - What type of house do you want to make?

R - Of Kotha (Pucca house).

I - Of what?

R - Pucca house.

I - Or Do you feel, money is needed for household (Living)?

R - Yes,

I - Do you have money?

R - Where is money madam? I am poor.

I - How much money was spent during your treatment?

R - I know only that at [redacted] I spent 1100-1200 Rs. How much was spent in [redacted] I do not know?

I - You do not know that. Who paid money?

R - No.

I - Who paid?

R - Wife gave most probably…………..

I - Are your Mother-Father alive?

R - Yes.

I - Do they know about it?

R - Yes.

I - You told?

R - No I did not know.

I - What?

R - I was not oriented. What did I know?

I - Why were not you oriented? What happened to you?

R - I mean, I could not even speak.

I - At that, you were not able to speak?

R - My whole health was bad.

I - Okay, And on your wife’s side (Wife’s Parents) or your Mother in Law or Father in Law.

R - So Mother in Law – Father in Law. Father in Law is not there.

I - Did you tell anything to your Mother in Law or your Wife’s family? At that time, when you were in a condition not to able to speak, your health was bad, who came to help you?

R - My wife came to help me.

I - Your wife?

R - There is one my brother’s wife, she helped a little.

I - Your brother’s wife?

R - Yes.

I - With money?

R - Yes.

I - Did anyone else helped you?

R - No one else.

I - No one else helped. Did you get any help from government?

R - From government?

I - Any amount of money? When you came for treatment?

R - No.

I - No, Anywhere?

R - Did not get till now.

I - Did not get anywhere?

- Okay, What do you think about your life?

R - In life, I think that if I get cured, will children, build house, do some take care of work.

I - So, Are you doing any work for last 3 months?

R - I am doing nothing for last 3 months.

I - You have not been able to do any work?

R - No.

R - Nothing.

I - How are you managing household expenses?

R - Brother is giving for expenses.

I - Do all brother give?

R - Yes, I have only one brother. He drives 10 wheeler. I get for expenses from him.

I - He gives?

R - I say Okay, I will work, will earn, then I will return.

I - Then you will return?

R - Yes.

I - Does his wife help you?

R - Yes.

I - You were telling, you have only one brother?

R - Yes.

I - Brother helps you?

R - Yes, he helps. He also does it with anger.

I - Why with anger?

R - I mean, He does not want to give (Laughing with hiding sadness). I have to make requests.

I - You have to ask repeatedly?

R - Yes.

I - Did you tell him about you?

R - Yes, He knows.

I - And, your brother’s wife?

R - Yes, She knows.

I - Okay, So you have told everyone in your family? I mean all people know it in your family?

R - They know, Everyone knows.

I - Okay. The treatment you are getting here, what about it?

R - I am getting good treatment.

I - Are you satisfied with treatment here?

R - Very Nice

I - Anything do you want to tell about this place? What happens here?

R - I get that T.B. medications on time. Get good injections, water, get food on time. What else should I get?

I - Anyone thing you want to be changed here? Or anything else you think it would have been good if you got. Do you think so?

R - What should I get?

I - What do you want to work? What did you think earlier? What works did you want to do when you had not get this disease 3 months back?

R - ……………………. Hmm, What should I say?

I - Like you have children?

R - Hmm.

I - About their studies? Their marriage? Did you think anything like this? About home?

R - I thought that. I was not able to do work for 2-3 months. I will work when I will get cured. They are studying. They do study.

I - In which school do they go?

R - They go to this government school.

I - They are studying?

R - Yes.

I - Has your illness affected their studies?

R - Yes, it is affected. They have left studies. They do some work.

I - Who has left studies?

R - My boys.

I - Since when did they leave studies?

R - Since 10-15 days.

I - Did they stopped going to school?

R - They go. Sometimes they go, sometimes they do not.

I - What works?

R - We say, you have to go daily.

I - Okay.

R - We say from here, then they go on time.

I - What works do boys go do? You are telling?

R - They forget to go on time while playing at home.

I - Okay. Children do not go because they keep playing and they forget.

R - They forget.

I - They are not going to school because of your illness, anything like that?

R - No. No. for studies.

I - That is okay. It has not affected that.

R - Hmm

I - Okay. Do you want to build house? You were telling something that you should build house?

R - I will have to build after earning money.

I - Okay. Did not you ever feel that your life is a mesh? What is meaning of being alive? Anything like that?

R - I had almost died, my wife saved me.

Long Pause……………………………….., I had almost died, my wife saved me otherwise I would have died.

I - So your wife saved your life?

For what did you get the first treatment?

For Kala Azar?

R - For Kala Azar.

I - After that?

R - For all of these.

I - After that you came here?

R - Yes.

I - So what are you feeling that do you have enough energy left for doing work, after you go from here?

R - Not now. Will rest for 1 month. Will rest for 1-2 months. Will get energy. If I will be able to do, then I will work.

I - How? If you will rest for one month, from where will you get money?

R - Will get from brother. What else?

I - Will get from brother?

R - Hmm

I - Okay, Did you think anything else for future? About work? How will you work in future?

R - I will have to work same as a driver otherwise I will have to do labour work.

I - Will you able to do?

R - Will not be able to do now.

I - As a driver?

R - Will think of as a driver after 2-3 months. If I will be able to do, then I will do otherwise I will leave it. What can I do? If I feel problem in abdomen I will leave.

I - What happens? Abdomen?

R - Yes, while driving in fields, it feels like discomfort in abdomen. (तबकता है पेट)

I - Yes, that moves you (वो जो हिलता है│)

R - Yes

I - Okay

R - Then I will leave driving and will do another work. Will go somewhere else (बाहर भीतर चले जाएँगे│)

I - What other work will you do? Like?

R - Other labour work. What else.

I - You will have to lift in labour also?

R - Just will have to lift only 2-4 bricks and give it to construction worker.

I - Okay. It is not much difficult work?

R - Hmm (Laughing) which one more difficult.

I - Did you think anything else for your work?

R - No, Not any more.

I - Or like starting a business or running a shop?

R - Not shop.

I - Did not think. Okay anything else do you want to say? Any problem arising from medications you are taking?

R - No

I - No problem. Hmm

R - Everything is okay

I - Everything is okay. There is no problem arising from medications?

R - No

I - you take medications on time?

R - Yes, on time.

I - Okay, Anything else do you want to say?

R - No

I - Okay, Thank You.
