# Supplementary material for: Quality of life perceptions amongst patients co-infected with Visceral Leishmaniasis and HIV: A qualitative study from Bihar, India
Source: PLoS One. 2020 Feb 10;15(2):e0227911. doi: 10.1371/journal.pone.0227911 (PMC7010301; doi:10.1371/journal.pone.0227911)
Supplement: S3 File — (ZIP) [file pone.0227911.s003.zip › Transcripts/Patient 25 Male Age 28.docx]

**Patient 25, Age 28 HIV VL TB**

I - You tell about-yourself, that this problem you have, this since how many days?

R - From one year

I - Before one year you do not have any problem?

R - No

I - Will you tell openly about this, Very first what problem started, then where did you go?

R - We ran a shop, then after that shop closed. After that was running a vehicle, driving/running a vehicle then after started feeling sick. Fever started coming everyday. Then I went to a doctor shown there. Fever was not going, then again we shown to Dr. Deepka kumar in Betiah doctor of fever shown to him, he checked after checking he told you have HIV. You go to Motihari then we didn’t go to Motihari, again we came to government hospital in Betiah, we came to Betiah then we got tested, after diagnosis/testing he referred to Motihari then there Jitendra Jee sir met us and he brought/takes us Motihari, came Motihari he told that from here medicine, it will be not cured, is Kala Azar, is HIV, it fever and all, T.B. was not there but not there, but here came to M.S then T.B. here T.B. was coming, then from there we got refer to Patna. Here we came then our testing/Diagnosis done, run medication and we admitted here two months in hospital, again 1^1^/_2_  months later then when it came time of relieving/discharge that we have to go home, then again we T.B. occurred, Treatment of T.B. again started/began. Then I again got healthy. So, from today again two months one day occurred again we from there got discharged.

I - So, you told that one year before you don’t have any problem?

R - No, there was no problem.

I - You told in your talk that you own a shop then it closed, then you started driving vehicle. So, the shop was your own?

R - Yes, it was mine shop.

I - Then how it get closed?

R - Was grocery shop, not ran, that’s why got closed.

I - So, this business not ran then, you started driving vehicle?

R - Yes, was brother’s vehicle?

I - Which vehicle was that?

R - Was Maximo Vehicle.

I - So, did you lived in Betiah? Or you come and go somewhere.

R - No, from Beitah come to Chhapra.

I - How many hours of journey?

R - Of half an hour.

I - In that how you make them sit everyone/all?

R - 15 men be sited, Rs.30 fare was taken from one.

I - Again whole day from morning to evening what you do?

R - Go/went – come

I - How many times most probably in one day you come and go?

R - Oftenly you can say one time/once, sometimes in only one side it get completed, we go and come only. Sometimes problem of vehicle happened then two side as supposed to be taken or either only one side.

I - That very first problem started to you, you told you were driving, then you felt fever?

R - Yes, during driving vehicle only I got fever.

I - After this what you did?

R - Gone to Betiah and shown there.

I - Gone Private or Government?

R - Gone to Private.

I - Was big hospital or small doctor?

R - No, was good doctor

I - Was his own clinic, or worked in hospital?

R - No, was clinic, of own, not worked at hospital.

I - Every one were aware/know him in colony?

R - Yes, everyone told show to K.K Sahay, So if there not cured then again to Rashid Ali, that/who worked in government hospital in Majhuwalia. Then from there medicines run 2 months, then also fever not cured.

I - So, Dr. K.K Sahay was in Private?

R - Yes, He was in Private

I - One month medicine took/ate?

R - Ate one month medicine.

I - Again what happened after that?

R - So after that not cured/got well.

I - They do not done any test?

R - No, not any test done.

I - Any blood test?

R - No, any test.

I - One month of which thing you ate medicine?

R - Of fever

I - After then what you did?

R - After then our weight become low.

I - How much you weight earlier?

R - Firstly 72 kg, Again got weighted there the it came 52 kg

I - Means you lost 20 kg weight?

R - At first was 52 kg, after 20 days weight done then 51 kg it become again 50 kg it became, so again we here to Dr. Rashid Ali Khan who medicates at government, shown to him. He gave medicine, till the effect of medicine fever was under controlled, Afterwards it happened/reoccurs.

I - He (Dr. Rashid) referred you any test?

R - Test was done but there was nothing.

I - So he told nothing was there?

R - No, he told Typhoid fever.

I - How many days you ate Typhoid medicines?

R - 2 months.

I - Total how many months you ate medicines, again how much money you spent?

R - Rs. 20000-22000 spent, in 3 months.

I - Dr. Rashid told Typhoid, then what happened there?

R - Again there not cured, then villagers told go to Dr. Deepak Kumar, then went there then he tested then again of 1000 test done of mine, then in that proved that I have HIV and he told you go Motihari.

I - Dr. Deepak was a private doctor?

R - Yes, was private doctor.

I_2_ - In this about HIV was known but about Kala-Azar nothing was known since then?

R - No, also was Kala-Azar

I_2_ - Now you say that he told you about HIV does Kala-Azar was also diagnosed in that?

R - Yes, the test which was done at Deepak kumar of Rs.1000 is diagnosed with both Kala-Azar and HIV.

I_2_ - But about T.B. don’t come to know?

R - No

I_2_ - Again after Deepak Kumar where did you visit?

R - After that we visited to government, and was very nervous. I felt that I won’t be alive, and thought life was got hell, and not satisfied then again came to government, there again test was done, same result came. One Jitendra sir is there he called me on phone and called me to Betiah. There from government hospital took address and came Motihari, again test was done there doctor saw everything then referred from there.

I - When Dr. Deepak told you about Kala-Azar and HIV. How did you felt after listening?

R - Everyone felt very nervous no food was cooked at home, my life was destroyed, there is no chance of being alive brothers were crying/weeping. How this disease happened.

I - So you told everyone about your disease?

R - Yes

I - Who people are there in your home?

R - Three brothers, Mother, Father Family is there.

I - Are you married? You told everybody

R - Yes

I - Wife and children?

R - All are there, two boys and one girl

I - Youngest child is how many year old?

R - Of five years

I - So, you told everybody at your home, to your wife also?

R - Yes

I - How they felt after hearing, what were their reaction?

R - After hearing everybody started crying, everybody were worried, how this disease happened.

I - Did your wife and children got tested?

R - Yes, they are not having

I_2_ - So, when you told that your life was over/Destroyed, of which disease you were thinking of that

I_1_ - Since you have been diagnosed with Kala-Azar and HIV both then by which disease you felt that your life was destroyed.

R - People used to say, illegal relation has been made that’s why he got this disease. I sued to read newspaper I knew I don’t got this as I was being told. Because 7 years ago I suffered from Kala-Azar, again it caught me once, again twice suffered from Kala-Azar. In that I got HIV.

I_2_ - So, you suffered from Kala-Azar, that time you were afraid, the same you feel for HIV too?

R - Yes

I_2_ - When you told in your family then what else people said after knowing?

R - Everybody started saying from where you bring this disease but they trusted me that I was not wrong father and everybody knew that from needle may be administered because my brother was with me all the time while driving and we always go together and come to together. So brother knew he was right. Therefore told, treatment will be done, and he will be taken care of .

I_1_ - So did you tell everybody at your home, the village you lived in Majhauli, There anybody having HIV you heard?

R - Yes, one man is having

I_1_ - What will be the age by the disease he is suffering?

R - 30 or 33 years, older than me.

I_1_ - Then how did you come to know about him? Did you tell about your disease in your neighbourhood?

R - Didn’t said, But people knew, they say he is having HIV. He will die, will die. But I was in hospital for 2 months, was miserable, was in very much fever.

I - For whom these people tell?

R - About me, that I will die, will die.

I - He is having HIV, How they came to know?

R - Oh one test was done there, he only made aware/spread to everyone about disease.

I - So, the village man made aware to everybody?

R - Yes

I - Once where you were tested, so you were being get tested to Deepak Kumar?

R - Beside him one Krishna test centre is there, two boys of our village work there, He only spread the news

I - We want to know any change in the behaviour of people before and after the disease?

R - Changes! The people who were literate, think they should not stand or sit, one or two boys are there, who are literate they think he is not having any disease.

I - Ok, they think this is not mere disease?

R - But those who tested knew he has HIV.

I - For this if there is any changes in their behaviour?

R - No, those who have diagnosed, come to our house, they sit, together also go for a walk too. I own my grocery shop in that I sit and run the shop.

I - Did you felt any changes in behaviour of your family members?

R - First when I was suffering from this disease, then one or two members think deficient/inferior. Brother don’t think like that but Sister-in-Law/brothers wife think like that, she used to tell he is having disease but family’s behaviour was god, Only brother’s wife’s behaviour somewhat changed. Her bathing, washing was separate, they are having one son, for whom they use o tell me don’t make him eat defiled food. Do not kiss him. My wife’s behaviour was also good.

I - Ok, she talked to you like that and behave also like that?

R - Yes, one or twice she spoke, in front of someone also told that she feels Yucky/hateful about him. So I told let it be. I will not touch his son, my son is there and sometimes they used to eat with us too.

I - So you have been explained that how this disease spread.

R - From eating, from shanking hand and from kissing it doesn’t spread.

I - This you can persuades them also, will see this later?

R - Gone to nearly for shaving, then they told he is having HIV. Then that Barber removed me, gone to another place then that barber also informed to him that he is having HIV, he also denied.

I - So, they also inform in group by phone?

R - The barbers were in contact with each other so he called him on phone, then I come back then I bought a razor, from that only I shaved.

I - So, According to you, the man who is 30 years old having HIV, How you come to know about him?

R - Have been diagnosed, so his wife was having HIV and she died, he survived, have been tested in village so he knew he is having HIV sometimes there were fights then people/they used to say that hit him with stick/cave. Otherwise if he will bite with teeth they you will have/caught HIV, if he will nail you/scratch you, you will have HIV. So if there were fight people used stick/bathi to hit, so we came to know.

I - That’s why people hit him with stick/cave as it may be spread by touching, so they don’t touch also?

R - Yes, Yes, don’t touch, Now everybody were like that then what we can do, don’t go there.

I - How many days back, story was this?

R - You think, It must be have been 2 years.

I - This not occurred very long ago?

R - No, No, his family also (wife too) died, he is having 2-3 children/kids, they are good, he is having, so he eat medicine or not eat, this don’t know. Newspaper comes in our house so our brother, father everybody eat together, brother’s wife felt something but now that also vanished, now she don’t keep that much feelings but then also keep alert, when other told don’t make eat to children, so don’t fed them, fed my son.

I - So, according to you for leading a good life. What things are required to man/human being? You are very sensible/understanding you talked enough about yourself. So we want to know your view that, for leading a good life what things are required in life.

R - For leading life first if a light spot/taint is applied then family think that…………This table is about, after disease.

I - If you remove disease then what you think what should happen?

R - Then, think that for children have a walk with them/drive them, for/to eat with them, sit, sleep with family, for kissing, they too sit but, live separate. When mother is at home, she tells them. But when come to shop then play together, come and sit.

I - How do you feel?

R - Feel very much, sometimes it happens that man should die that will be much better, But 1 second, like me so many people are there, many people if die, no space will remain.

I - You feel that you should die, sometimes you feel like that?

R - Yes, before it was

I - This type of imagination comes?

R - Yes very much felt like, that, that, should go somewhere, Should hang myself, again after that I started seeing newspaper, started taking medicine then told that so many peoples are living and/तो I am having three children’s, I will also live like that not meet with others, only will do own work.

I - Your’s thought is good, that you very bravely tackled your imagination, when you felt like that and anything you felt?

R - No, felt anything, only these friends who were together with me while eating make me eat, now don’t eat, don’t go. Now I don’t go to anybody’s door, go to my shop sit there.

I - Job of driving?

R - Now, left that

I - Again you opened shop?

R - Yes, again opened shop.

I - If everybody comes to your shop to borrow anything else?

R - Yes, everybody come. Nobody think that he is having any disease. Nobody knows that he is having any disease.

I - So, now when this type of feeling came inside you then did you told this to your family, wife or to anyone?

R - Yes, had told, my family too arrived in hospital have seen there. Again when went to Motihari then there also they saw, then she told, suppose if you give up your life then who will look after three kids…. If suppose, I too may half drowned, then will wherever go. People will complaint! She is having this and that. They will spit on our family too. You stay at home then where is problem of eating and drinking, from government side grains/Ration is supplied, Ration work will be fulfilled. Vegetables also will be managed. Have opened shop, from there buy and eat, managed from that only. Father is there Rs. 10-20000 he given, then opened shop and sit, Vegetables are managed from there only.

I - You are having vegetable shop?

R - Is grocery shop, Potato, Veggies all we kept.

I - So, to run that shop your father gave money?

R - Yes, father had given only, for starting.

I - So, your mother, father and wife supported you very much?

R - Yes, brother also supported, they knew everything then also they never denied in anything. He knew that this is spread through blood and Physical relationship and from nothing.

I - So, for leading good life in your opinion how money is needful? Is money important/needful/necessary?

R - Necessity/Importance of money is there, Now man, other work we can’t do go to other place then keep on commenting, keep on talking, If will do own business then nobody will say that he is having disease.

I - According to you being healthy is how much important/necessary, good health?

R - Health is very important, sir, told that take medicines on time then no problem will occur. So I don’t leave medicines, eat medicines on time, at I.

I - How is your surrounding?

R - Everything is alright environmental alright. In starting 5-10 boys were there of village, in Holi and every festivals they used to come now they don’t come, not eat together, now don’t eat.

I - The disease you have, did you felt any pain also?

R - No, not any pain, only fever.

I - Besides fever any other Complain/Problem?

R - No

I - Any weight loss?

R - Yes, weight was decreasing day by day.

I - Breathlessness?

R - Here you can say that, when was admitted in the hospital kidney problem was also there, stone was also there, then everything treatment was done. Everything got alright.

I - Have you ever been complained/in problem of breathlessness in one year?

R - No

I - Does blood came after coughing or vomiting?

R - When I had T.B. once I coughed, blood came then told to sir, that blood has come.

I - When did you experienced blood/when it come?

R - In this RMRI, once blood come. After that don’t/it not came.

I - Any other problem, the medicine you eat, have you any problem from medicine?

R - No, not any, only in night not feel sleepy. It is hot is night.

I - How was your sleep in beginning/before?

R - In beginning didn’t wake up in early morning.

I - Now?

R - Starting, wake up at 4’O clock, now can’t wake up.

I - Now you left driving job?

R - Yes, Now left that work.

I - How much you enjoy your life after having this disease?

R - Now you think, I eat my medicine, busy in myself and my family, living in my kids if somebody fights then I don’t go there.

I - You told earlier that your weight was decreasing it become 50 kg, now after taking medicine you feel any improvement?

R - Yes, now increased.

I - How much increased?

R - Today it is 71.1kg, As it was before one year. Now it become that much only

I - Now, while doing work do you feel energised/strong?

R - Yes, I feel energised/strong enough, no any problem in doing work.

I - Are you satisfied with your sleep?

R - Yes

I - And you, When you went to RMRI, are you satisfied by their treatment?

R - Yes, am satisfied, going to that hospital my life was saved. If I have been went to other places, have couldn’t saved my life. There medicines, food and, medicines served by nurse. Everything was very good.

I - If their behaviour for you was good?

R - Yes

I - Did they talked to you nicely?

R - Yes. In 15 days they used to call me twice that Rajan jee how are you? If there will be any problem then tell us. Oftenly used to call me on phone.

I - So, do you want any change or suggestion in treatment, or you are fully satisfied?

R - Am satisfied, the only change would be if any such medicine would come that cure if from root, everybody become happy but yes everybody knew that in year. 2 year this will be erupted from root.

I - This is good that you feel that energy is coming in your body and everything is running alright. You told about the medicines, beside that what you want to do in your life?

R - In home nobody wants that I should do some other work, look after home and family, see the shop cut fodder for cow, after then come to shop run it.

I - No, I was asking to you that what other things you want in life, that may, I should do/complete this work in life. Everybody is having some goal in their life.

R - Kids study and run the shop nicely, ear some money and proper educations of my children now they study in government, and everybody (other children) study in private, now having no money they study in government.

I - Did you think, earlier make them learn in private?

R - Yes, I thought.

I - So, when you were driving vehicle?

R - Yes, my children too would learn/study in private, would drive vehicle nicely, had earned money but that not happened.

I - Any effect on your children’s school due to your disease/illness, like drop out from school?

R - No

I - Yes, all three go to school, one in Anganwadi and other two in government.

I - So, before you got diagnosed with this disease, what other things you thought of doing? Like many people think of making home/house, did you thought like that?

R - Thought that

I - What did you think of, tell me?

R - Making house

I - Now what do you think?

R - Think that, if my life would saved then will earn money. House will be built then after.

I - Now, how is your house?

R - Now, it is of straw, Brother’s house is having roof.

I - Brother’s are separate?

R - Yes

I - Kitchen is separate?

R - Yes

I - And of mother father?

R - Father is in older brother, our separate and brother also live separate. Two people and my father are in one.

I - In your family, you, your’s wife and children beside them nobody is there.

R - No

I - Your’s house is of straw, So do you think to repair it?

R - Yes

I - When Kala-Azar was diagnosed, Did Dr. Deepka kumar told you about it?

R - Yes, he told me to go Motihari

I - From there your medicine got started?

R - No medicines got started from there, directly from here medicines got started. He told that you are diagnosed with two diseases HIV & Kala-Azar. So medicine will be given there, if you were having HIV only then it would be started from here.

I - So, what do you think of your future now?

R - I think that in future if medicine will come the men will lead their life nicely. There is hesitation, is a fear, don not attend any function, somebody laugh at us, comment on us, so we don’t go. People think that we are ill, so didn’t come. At home only, cook and eat food. Papa or brother if they bring then eat that otherwise eat at home only. Remaining beside/far away all eat together. Who knows who is having disease.

I_2_ - When you were diagnosed with T.B. then what else you were feeling?

R - Feeling that, when only 5 days were left for discharge and then I was diagnosed with T.B. and continuously 6 days I suffered from fever, it was 106, 107 and at that time I ate pill of 9’O clock Right after that or how it happened after that I diagnosed with T.B. Then I started thinking what disease has caught me. Then sir told don’t worry, everything is cured/treated here, sir told everything alright.

I_2_ - How long you took medicine of that?

R - Means, It was up to yesterday, now will got to Motihari, 6-months course has been completed. Today is closed, tomorrow also will be closed, day after tomorrow will go, then sir has told 6 months course has been done. When didi will tell you to stop then you stop it and if will say to continue for a month, the get it started.

I_1_ - T.B. medicines were run from Motihari?

R - Yes

I_1_ - And HIV medicines were also running from there, so, when you go to take medicines of HIV from Betiah then how much time and money you spent?

R - Money spent Rs.60 from one side, Rs.60 from other side, Rs.120 and again Rs.20 more fare it took, Rs.10-10 of both sides. Took Rs.140 spent only in fare.

I_1_ - So, T.B. and HIV medicines were provided were given together or you have to go separately for that?

R - In starting, have to go separately twice in a month, again didi stopped medicines for 17 days in Motihari, said Rajan your medicine got done. But when I added it was 4^1^/_2_ months only. Again I called to Rohit sir didi throw the file, told no medicine will run. I calculated there 4^1^/_2_ months, Sir told ok, you come here, very next day date was given here, came here and shown to sir then sir asked, If I was having Jitendra Jee’s number. I said yes, then called Jitendra Jee and asked why you stopped Rajan medicine then sir told no, I haven’t stopped. Didi might have stooped. Then she again calculated it was 4^1^/_2_ months. So it stopped on 17^th^ date and again started on 29^th^.

I - So in earlier there were gap of 15 days to get medicines. Now that both medicines were given.

R - Yes, given together

I - Earlier it was given separately?

R - Yes, earlier it was given separately.

I - So, In a month twice Rs.140-140 must be spent.

R - Yes

I - At one time for how many days you get medicines?

R - Of only one month it was given.

I - Are you alright, satisfied with this?

R - Yes, I am taking medicine, so am alright. If medicine would not run, then life would become hell/destroyed. After eating medicine am alright.

I - Anything more you want to speak about yourself for this?

R - I would like to say that for families happiness that, some kind of medicine should lie developed made that this disease would be vanished or erupted from the root by that medicine. If such medicine will come then more spirit of happiness will arrive in home.

I - So, you want such medicine that can erase this disease from root?

R - Yes that’s it, Now I don’t go to anybody’s door, be seated all the time.

I - You do not talk?

R - If somebody come on shop then talk to them, otherwise don’t go. If 10 boys keep standing somewhere then also I don’t go there. Men makes laugh. Say that he is having this disease.
