# Supplementary material for: Quality of life perceptions amongst patients co-infected with Visceral Leishmaniasis and HIV: A qualitative study from Bihar, India
Source: PLoS One. 2020 Feb 10;15(2):e0227911. doi: 10.1371/journal.pone.0227911 (PMC7010301; doi:10.1371/journal.pone.0227911)
Supplement: S3 File — (ZIP) [file pone.0227911.s003.zip › Transcripts/Patient 26 Male Age 37.docx]

**Patient - 26, Age - 37, HIV VL TB**

I - Will you tell me a little about yourself from where you come here, from starting.

R - I am from district Samastipur VS+PU Bariyanwa Jalapur. I under wnet investigation in Jazipur, they reported HIV, then I went to Jazipur government hospital, where I was investigated four time and said same.

I - What Said?

R - HIV and T.B. was not said they said your medications is not here, your medication will be started from district hospital. Then I went back to Samastipur, Sir. I underwent investigation is Samastipur, and then started medication from Samastipur. Then I started medication for Malaria, Kala-Azar from Samastipur. They refereed me to RMRI Patna for Kala-Azar for four-five day underwent sevral investigation, in initial report everything was ok, in second series of investigation I lost my consciousness, for 24 hours there were 2 doctors, both were on continuous duty; they said. “It’s our patient nothing should happen to him, if something happens then it will be a big loss” They treated me till my health started to improve.

I - Answer one thing you said earlier that you went to Hazipur? When you were first informed about the disease, when you first feel fever?

R - First time 6 month earlier sir

I - Started 6 you earlier, what-what started fever was on, hunger was lot, stone in stomach developed, half of the stomach was converted to rock type, cereal were not digested; everything I eat was not digested, then I saw myself to a doctor.

I_2_ - 6 months back, you were absolutely alright you had no complains?

R - Means fever occurred several time?

I_2_ - From when was the fever?

R - Fever was from 1 year, Madam.

I_2_ - How long earlier you were absolutely alright; free from any complains?

R - Before 2016-2015 I was completely all right.

I_2_ - So your difficulties started from 2015

R - Yes

I_2‑_ - What happen to you in 2015?

R - This fever, cereal not digested, slowly-slowly body started weaking.

I - How do you come know that your body is weakened?

R - As I use to any work, madam, the power is starting use to be ok then I use to feel weak. When weakness started I started feeling it.

I_2_ - Any change in weight?

R - Yes there was change in weight and body too. Weight kept on decreasing.

I_2_ - What change in body?

R - Madam, I became lean and thin stomach protruded outside and body progressive became lean.

I_2_ - Do you get yourself weighed somewhere, how much reduction in weight occurred?

R - No madam, I am driver by occupation, madam this thing, we don’t do.

I_2_ - When you felt the difficulties then where do you go?

R - When I grew completely weak and there was nothing I can do, then I stopped driving and show myself in Hazipur.

I_2_ - You were taking about thing happened in 2015?

R - No in 2016

I_2_ - In 2015 when you felt fever & chills, where you went for treatment? Do you drive truck?

R - Yes, in Andhra, there was a small doctor whom visited. I developed backache and progressively become weak then I said people form Bazar Samati that sir now I can’t tolerate the pain in back. Then they took me to a doctor where I was given 2-3 injection and medicine for 2-3 days. Then I returned from there and I developed cold in 2015. Yes I drive truck.

I_2_ - After visiting to doctor in Andhra, After now many day form then you were effected by cold?

R - Almost 20 days after

I_2_ - Where were you then?

R - I was going from Andhara to Tiliya which is in Jharkhand Jhumari talaiya

I_2_ - So when your health become bad, you were in truck and driving it.

R - Yes mam I was on duty

I_2_ - So what had you done?

R - So I arrived and said I man passing stool like blood.

I_2_ - You mean to say blood in stool

R - Yes, blood in stool was coming for 4-5 day, I use to pass this 3-4 times in night

I_2_ - When you went from Andhara to Tiliya, This thing happened?

R - Yes

I_2_ - So you informed this to anybody?

R - I informed this to my master, who is form my village.

I_2_ - So this is how you come to know that you have caught cold. Else you have problem of passing stool 4-5 time.

R - Means cereals were not digested?

I_2_ - Then what happened?

R - Then he got me medicine form medical shop.

I_2_ - Going back, when you were in Andhra. When you got cold & fever, your health deteriorated 50 after how many days you visited small doctor?

R - 5-7 days after

I_2_ ‑ For 5-7 days you didn’t take any medicine, you were in pain for these drug?

R - Yes, like this only in pain.

I_2_ - After how many days of blood stool you took medication, which you informed your master.

R - 20 days.

I_2_ - For 20 days you didn’t take any medication, you were left like this only.

R - Yes in the very same way.

I - Then after this where you come?

R - Went to Tailiya, Where I took medicine for cold, bloody stool also improved.

I - For where you took medicine?

R - In Tailiay there is Jhanda market from a shop there.

I_2_ - Do you show it anywhere?

R - No

I_1_ - Why you don’t show it to anyone?

R - Master said it is a normal medical shop, dispense medicine, his medicine suits.

I_2_ - What do you do after that?

R - After this I take medicine from 2-3 days. Stool became alright, but body progressively became weak.

I_2_ - What do you do after this?

R - After this I returned to Hazipur, here also I was unable to digest the cereals, status remain the same, but here, I was able to digest still have to pass stool 4-5 times.

I_1_ - In Hazipur you visited government or private?

R - Visited private first

I_1_ - What did they say to you?

R - There they wrote USG, Blood test and X-ray

I_1_ - What outcome their?

R - There I visited doctor with report, Dr. Amit Kumar checked me and said you have HIV, you visit big hospital.

I_1_ - At present you have HIV< Kala-Azar and T.B. at that time what were you informed only HIV, T.B. or Kala-Azar?

R - At that time I was informed nay about HIV only.

I_1_ - Private doctor said what?

R - Sir, then visited government hospital in Hazipur, there I asked blood test to be done, they asked for what I said for HIV they tested 3-4 times. They said they have doubt for two times and on third time they said confirmed HIV.

I_1_ - You knew you were suffering from HIV then you visited government hospital to confirm it.

R - They said they won’t believe on the reports of other. They will test by themselves.

I - They investigated and after three test they said you have HIV.

R - Yes

I_1_ - Ok, Is their anything Kala-Azar?

R - At that time their was nothing of Kala-Azar or T.B.

I_1_ - When they informed you about HIV, did they informed you how it happens?

R - Means it happens by two three way, wrong did’s from injection, injection used in infecatead patient and then used to you, third they said from blood from HIV+ , from HIV+ blade used blade used.

I_1_ - This information was shared to you in government or private hospital?

R - No sir, it was informed in government hospital.

I_1_ - What was informed in private hospital?

R - In private4 they said you are suffering from HIV, visit some big hospital.

I_1_ - Ok, they said that it will not be treated here?

R - Yes, it will not happen

I_1_ - After that what happen to you?

R - In Hazipur they asked where is my house, in which district. I give full address then they said, you will not get medicine from hear cared will be made from Samastipur district and treatment also.

I_1_ - So card was made in Hazipur and medicine was given in Samasitpur.

R - No sir, card and treatment both from Sasmasipur.

I_1_ - So after how many days you visited Samastipur.

R - After 8 days I visited Samastipur, there I took 8 days, there blood test was done again, T.B. test was done, they didn’t informed about T.B.

I_2_ - In Samastipur they didn’t informed about T.B.

R - No

I_2_ - Kala-Azar

R - No sir they didn’t said anything, I took medications for HIV for 1 month, their also fever didn’t came down, then I visited a quack he said to investigate for Kala-Azar.

I_1_ ‑ Quack said about it.

R - Yes, then I visited a special doctor who see’s patient in both government and in private, I asked him to investigate me for Kala-Azar, he asked to salt outside, and was diagnosed Kala-Azar there after 15 days of taking medication.

I_1_ - After taking HIV drug for 15 days

R - Yes

I_1_ - So you were not informed about Kala-Azar in private. Hazipur or Samastipur, then you visited special doctor were you were diagnosed.

R - Yes

I_2_ - So you said doctor to investigate for Kala-Azar, after you weak advised by quack, he himself didn’t investigate for himself.

R - Fever remained for 24 hours. Sometimes increased sometimes decreased, but never free from fever.

I_1_ - So it was like this from 2015?

R - No sir from 2016

I - What happen afterward.

R - When I was diagnosed with Kala-Azar, then I became very weak, and then I as referred here.

I_2_ - How much do you weight now?

R - 30 kg will be my weight now.

I_2_ - How much it was earlier?

R - Earlier it was around 55-60 kg, means I was almost reduced to half, when I see myself in mirror. I used to say that I have reduced to almost a skeleton, heard even looking to myself, how was my face and how has it become now?

I_2_ - Do you accept yourself like this?

R - No, for 2 months I haven’t seen myself in mirror. When I was admitted to RMRI, I was defected, visited boring road for x-ray. Lost consciousness for 24 hours.

I_2_ ‑ From where do they send for x-ray?

R - From there RMRI

I_2_ ‑ This all from starting?

R - Yes, at that time my medication for Kala-Azar was not started; I show my full reports, they said that this reports doesn’t have any value; will have to repeat all the investigation, then will start the drug.

I_2_ ‑ Than what happen?

R - After this I as admitted for 2 months and now I take medicine for HIV and T.B. from Samastipur.

I_2_ ‑ How many days of medicine do you get?

R - 1 month

I_2_ ‑ When do you come to know about T.B.?

R - T.B. was diagnosed here.

I_1_ - After how many day, medication was started?

R - After medication started here, I was informed of T.B.

I_1_ ‑ So they also investigated for T.B.?

R - Yes, they investigated sputum.

I_1_ - Do sputum was strained will blood or anything?

R - No, blood didn’t come with sputum, but I use to cough.

I_1_ - How long you were suffering from cough?

R - From many days.

I_2_ - Then also you were saying from 2015?

R - I have cough for 5 years

I_2_ ‑ What type of cough it is?

R - Dry cough it was.

I_2_ ‑ Do you take bidi, cigarette or do you drink anything?

R - Madam, I was in that line so use to smoke and drink a little bit

I_2_ - Does it happen like this or but increased on smoking?

R - Never experienced it.

I_2_ - Cough was there for 5 year, do you visited anybody for this?

R - No for cough I didn’t visited anybody, use to take cough syrup from medical store, and drink it, and was relieved form it a little bit.

I_2_ - How as the weight at that time?

R - Never measured it

1_2_ - Weakness you were taking about, do you feel like that?

R - No mam nothing like this.

I_2_ - Any other difficulties, chest pain?

R - Look, in between, I developed habit of chewing tobacco. So whenever I chew I developed pain in chest then I stopped chewing tobacco.

I_2_ - So the pain was very intense in nature?

R - No it was not like that

I_2_ - Do you feel shortness of breath?

R - In very late stage, I had shortness of breath I can’t breathe from nose.

I_2_ - Last means which year?

R - 2016 last, 12^th^ month of the year, when I use to sleep I was difficulty for me to breathe; so I have to breathe with mouth open. Throat use to dry up so I have to drink water 5-6 times.

I_1_ - Do you visit anybody for this problem?

R - No sir, not visited any doctor, directly I have consulted here in Patna.

I_1_ - Why don’t you investigate for cough when you say my throat used to dry up?

R - None of the government doctors could catch it, one doctor in Bidyopur I consulted but he said nothing.

I_1_ - You have cough for 5 years, why did it take so long to meet a doctor?

R - Means, long as I had power in my body, in ran, poor men sir, Grihast ashram men sir, not felt it’s needed to anybody. When started becoming weak and your body power is source of earning for use then doctor sir, I visited to doctor, else use to take medicine for the medical shop.

I_1_ - If I ask what do you need for a good life than what will you say?

R - For good life at present I need to cease smoking drinking alcohol and have to take medication timely?

I_1_ - And anything else?

R - Yes sir almost

I_1_ - What apart from health you think essential for your life.

R - Apart from health, family, wife and child.

I_1_ - Tell something more

R - We say good life to this only; living happily educating child properly and marrying them.

I_1_ - What about employment

R - We were unable to do work as we were illiterate I will educate my children and let-them do good work.

I_1_ - Yes all this are important

R - Yes

I_1_ - When you were first informed about the disease what do you thought about your life, was it possible or not?

R - No, I was nervous at that time, life had almost ended due the disease; was very much depressed, what would happen to my wife children; now I can’t do anything.

I_1_ - Were you worried about your future?

R - Yes

I_1_ - Worried for what?

R - Worried for children and nothing else we don’t have land, we have to earn every day to eat, earned a bit, educate the children and make some saving for future what else. What else to worry for.

I_1_ - How much does your illness cost to you.

R - I have spent almost 2 lac in private for investigation and medications.

I_1_ - From where did you get this 2 lac.

R - Sir, I was driver by occupation, had a small family and could ear maximum of Rs.10000/- per month. So I used to save money form this. This 2 lac was my saving; which now I have stopped in this disease.

I_2_ - Do you how to lend money from any were?

R - No, Mam

I_2_ - You said you have land in Samastipur did it needed to sell it out?

R - No mam

I_1_ - How is your house

R - Normal house sir, with four room

I_1_ - Kuccha or Pucca

R - Pucca

I_1_ - Roof is RCC

R - Yes sir, we four brother have made it together, father in high lifetime was unable to make. All four us are driver by occupation.

I_1_ - All of you live together?

R - Sir, now that we all are married, we live separately and cook food separately.

I_1_ - Who lives with you in your family?

R - Myself, my wife, children and my mother, father has passed away 10 days back.

I_1_ - So your father have passed just now.

R - Yes, from then only it has increase, we have ritual for 13 days after passage.

I_2_ - How many child do you have?

R - Mam 2, 1 girl, 1 boy

I_2_ - Age of boy?

R - 10 year of age, lives with my sister in Ranchi

I_2_ ‑ Do you get your wife tested.

R - Yes three months back

I_2_ - So she doesn’t have

R - Yes mam, she doesn’t have

I_2_ - How old is your child?

R ‑ She is 6 year of age

I_2_ - Why don’t you get her tested?

R - No sir, doctor sir said that It’s not needed but I am thinking to get it done once.

I_2_ - When you heard that you have HIV, how do you feel after hearing this news?

R - Felt very anxious

I_2_ ‑ Do you develop any negative thought about your …….as life is waste, what’s need to live?

R - Yes mam all this idea’s came due to this dangerous disease entered my body and might engulf my family.

I_2_ - Do you tried or thought to suicide?

R - No never, thought to leave home, but not to suicide, will earn money outside, educate the child, this is what I have thought off.

I_1_ - Wow did you inform this at our home?

R - At home my wife and my mother knows it.

I_1_ - How did you tell them about this disease do you tell it outside?

R - No sir, id didn’t tell them, here doctor sir inform. It to them and advised them to maintain distance.

I_1_ - Do you inform it to so anybody else?

R - No

I_2_ ‑ Brother, to sister in law, father.

R - No

I_2_ - Do they know it now?

R - Yes

I_2_ - how do they know it?

R - I, informed it to them.

I ‑ To all your brothers?

R - No, I informed to my younger brother, I didn’t talk to my elder brother.

I_2_ - What did you say them?

R - He asked me what is there in report, then I informed them about the disease which has engulfed my body.

I_2_ - Ok, so when you informed them about your disease was there any change in behaviour towards you.

R - No, there was no change

I_2_ - So they were exactly same as earlier they use to.

R - Yes, the very same

I_2_ - So you didn’t notice any change in their behaviour?

R - No sir not at all

I_2_ - Do they eat with you?

R - Yes sir, they eat with me. Me younger brother have 2 child, he never ask them to stay away from me.

I_2_ - What about your wife attitude?

R - She speaks out sometime, asks me from where I get this disease, then I say, I am only having this disease, there are many more living with this, visit hospital someday you will see hundreds in line, taking medication form 10 yers-15years, and I am taking it from 2016 only.

I_2_ ‑ Do you feel any change in your wife attitude anytime?

R - Yes mam some time she become angry.

I_2_ ‑ Do you know anybody in your neighbourhood who takes such type of medicine?

R - No, none, my elder both brother have same disease and he has passed away.

I_2_ - Was he elder than you?

R - Yes he was elder than me

I_2_ ‑ So he also died with same disease.

R - Yes with same disease

I_2_ ‑ How before he died of the disease?

R - Around a years back.

I_2_ - After this death, his family live together?

R - Yes, brother has also died and sister-in-law (Bhabi) has also died from this disease, one nephew is there whose marriage. I have managed to do; in Patna district, near Mokama side.

I_2_ - This is all about 3 brothers, where is one brother else?

R - He is at village, looks after farming and housing.

I_2_ - You did not tell him about this?

R - He has knowledge that he has this disease but I have not told this. That quack doctor has linked, he has told this.

I_2_ - Other people of village, with whom you sit and stand. Is there any change of behaviour?

R - No, not any change

I_2_ - Do they know, you have this disease?

R - Yes, they know, then also there is no change in their behaviour.

I_2_ - Like for making beards (having)……share at home or you go outside?

R - Make outside?

I_2_ - Do they make it outside?

R - Yes-Yes

I_2_ - The barber who are there?

R - Yes, they make

I_2_ - Like earlier, you used to feel that what will happen to family; after treat your feel same?

R - Like doctor sahib told, there is no permanent treat of this disease, if you take 24 hours medicine, this germ c virus/will be dormant.

I - What do you think about this?

R - This that, I will have to eat this medicine for life throughout, but doctor Shahab at RMRI told that it is possible that some new drug will cure, which may uproot it.

I_1_ - What you told was about HIV, what’s your opinion about Kala-Azar?

R - I had suffered from Kala-Azar 12 years ago.

I_1_ - What had happened at that time?

R - There was no any test done at that time, one vial was given which contained 12 (injectable) needles, it was very hard, I used to take that injectable, but Kala-Azar was not cured at that time.

I_1_ - When it happened to you?

R - I was (2 years old at that time)

I_1_ - Where had you shown yourself, at government or private clinic?

R - Had taken medicine in village, doctor sahab used to come and give injection daily.

I_1_ - Disease had ended (cured) at that time?

R - Disease had not ended that time, but doctor Sahab told that disease not ended by root (cured). Something was left.

I_1_ - What, What happened to your body?

R_1_ - In Kala-Azar, my half abdomen had become stone, abdomen had distended, had a lot of weakness, and fever was not leaving me.

I_1_ - What problem it had on work?

R - Work, mean, sir fever was there, food didn’t seem well, water also didn’t seem well, so it affected work sir? More sleep, had weakness, it was drivery line, more of night duty, less work was in day.

I_1_ ‑ So what had to be left?

R - Sir from 1^1^/_2_ years, I had left house

I_1_ - During this time, you not used to earn?

R - Nothing now, managing taking debt from either and neither.

I_2_ - Who is supporting you right now? Help from brothers?

R - No, I have a friend, he is support me, I take loans from him only

I_2_ - Then how do you pay for your daily requirements like groceries?

R - I have nothing, whatever is……..from my body.

I_2_ - Any farmland?

R - I have a little of farmland. My dad has given it to my elder brother.

I_2_ - You don’t have a share in that?

R - I do have a share, but at the moment I haven’t been given it.

I_2_ ‑ You haven’t been given it?

R - No

I_2_ ‑ Any inheritance?

R - No, nothing

I_2_ - So far the last 1^1^_/2_ you are making ends meet with the help of your friends?

R - Yes he only is supporting me. He has 2 sons, born in the military, earlier he was also, driver, both the children studied & joined the military, he too has 5 kinds of disease, he has kidney stones, blood, sugar problem, the salt disease is no less visible, he says I shouldn’t worry, that I had taken care of him earlier so he will do the same for me now.

I_2_ - So how had you helped him?

R - I mean, he had asked for money for a job for the employment of his 2 sons and both of them got employed at the same time, I didn’t have money, but my wife took a loan of 50,000 and gave him.

I_2_ - So tell me, what are the requirement to lead a good life?

R - Madam, for a good life, the most important thing is this world is money, if you have money, you are not face any difficulties. After that one can get their children educated and get those jobs, or one can start some business, half of my life is already one, now I love more about my children, that they should earn, eat and lie well. I have a daughter, whom I want to marry off in a grand mama.

I_2_ ‑ Other than these, how important is health?

R - Health is very important madam, without health how can one live a good life, the biggest thing is to have a healthy body. To maintain my health I take medicines from time to time, I need to eat, take a walk, then only can my health remain fine.

I - For how long have you been taking medication for T.B. (which you have mentioned about)

R - Sir I have been taking it for 5 months

I - Is it complete?

R - No, I month course is still left. I have to go on 5^th^ of next month to take medicines.

I - Are medicines for HIV & T.B. available together at one place?

R - Ok yes, they are available together at Samastipur.

I - Is have any difficulty in going there and receiving medicines?

R - No sir, there is not much difficulty, government auto rickshaws are usually available.

I - Can you give an idea of total expenditure/cost of going and coming?

R - About Rs.200/- is spent in total

I - Do you go alone or your wife accompanies you?

R - I go alone

I - Do you go only once a month to take medicine?

R - Yes

I - You are saying that after one month, course for T.B. will get completed, Right?

R - I swear Sir, Doctor has told me to take medicine for one month and then my CD4 count will be tested.

I - But CD4 is done for HIV. What has he said about T.B.?

R - For T.B. he has told that a bit more is left.

I - Do you take your medicine daily? Do you have any problem?

R - I don’t have any problem

I - What about the problems you earlier had?

R - Soon after taking medicines, I used to vomit.

I - And now?

R - Now, I don’t have any problem. I take 3 tablets of T.B. empty stomach, one tablet at 9:00 P.M.

I - Is your digestion normal?

R - Yes, My digestion is first class.

I - How much do you eat?

R - My health is alright. My weight has become 50 kg

I - Tell me one thing, how much you of used to eat during your illness and how much are you eating now?

R - During illness I used to eat sometimes one chapatti and sometimes two, and little bit of rice. Now I am able to eat 5-6 chapaties.

I - At your present condition, what do you think you should do?

R - Presently I am keeping faith and courage to do that much only.

I - What do you think, which kind of work you can do?

R - Presently, leaving that work, I won’t be able to do any other work madam.

I - Can you drive? Do you feel yourself capable?

R - Yes, I can drive

I - Before your illness, whatever hopes you had form life, do you still have?

R - Yes, Now also I have hopes.

R - Yes, you can get free medicine there.

I_2_ - Anything except medicine?

R - You all get fruits except medicine.

I_2_ ‑ Where?

R - In RMRI

I_2_ ‑ Anywhere except RMRI?

R - Only medicines are available, in Samastipur

I_2_ ‑ What is the expenditure in travelling to Samastipur?

R - Rs. 200, including snacks.

I_2_ ‑ Anything else you want to tell?

R - What else

I_2_ ‑ So, when are you going to start?

R - I have weakness right now. I will complete the 1 month course, which is left. They give 15-15 days of timing during a month of course at RMRI. SO, if once started, I would not be able to go for my duty anywhere at two weeks interval. Whenever they start long course, like of 2-3 months of medication, then I would start going for duty.

I_2_ ‑ You would like to take medication for 2 months continuously, why so?

R - The business of drivers works this way only, ma’am. Like if we go to Nepal, then 5-6 days are spent in traffic. I would mises The dose then if there is medication for 2 months, then I could take the medicine form Samastipur as we can avail it 5 days before and Samastipur falls in the way of Tilaiya to Nepal.

I_2_ ‑ So, what would you do now?

R - Now, as we brothers have separated, so we bought land in Ranchi. I was planning build a home for my children, as the villagers don’t have a good behaviour.

I_2_ ‑ You want to take them away from village?

R - Yes

I_2_ ‑ That’s why land in Ranchi! What’s the plan next?

R - I have bought land now and I am already tried! Nothing has happened yet. My sister lives there, she suggests to build home by getting loan on the same land.

I_2_ - You eat medicines, are there any side effects?

R - No sir, everything’s fine.

I_2_ ‑ What do you have to say about RMRI, where you got treatment.

R - Mam, they have treated well there. I know few sir there Ranjeet sir, Deepak sir. They are like God to me.

I_2_ ‑ Do you want any improvement there?

R - I just want a medicine to be developed which treats the disease by its root. I just pray to God for the same.

I_1_ - And when you visit any government hospital, what more facilities do you want?

R - I want life, nothing more than that.

I_2_ - This benefit you did this in any way?

I_2_ ‑ Any other change you want?

R - What new change could be done, doctors feel that this disease don’t heave a permanent treatment so after hearing this I become scared.

I_2_ - Like this there are many disease such as diabetes, hypertension, which do not have a permanent treatment, patient have to take lifelong medication?

I_1_ - What difference does it make, fi you suffer from diabetes, hypertension you have to take lifelong medication?

R - Mam, this disease is very common disease but people are HIV disease as an evil eye. Those how are little learned only they don’t see it as an evil eye.

I_2_ - But from conversation with you I didn’t feel that your family, village, family members, relative have misbehaved with you or you had to face any kind of discrimination.

R - I don’t have felt any discrimination. Initially for one and half month I was at my in laws place. They used to eat with me, then was no difference in the talking even. But sometimes after seeing other people, people feel bad about this.

I_2_ ‑ Do you feel like that?

R - Yes maximum when my brother was diagnosed with same disease then I felt that.

I_2_ - Do you felt that everything was not right?

R - Yes mam

I_2_ - And when you yourself suffered from the disease.

R - Then felt about this

I_1_ - Anything else would you like to say

R - No sir
