# Supplementary material for: Quality of life perceptions amongst patients co-infected with Visceral Leishmaniasis and HIV: A qualitative study from Bihar, India
Source: PLoS One. 2020 Feb 10;15(2):e0227911. doi: 10.1371/journal.pone.0227911 (PMC7010301; doi:10.1371/journal.pone.0227911)
Supplement: S3 File — (ZIP) [file pone.0227911.s003.zip › Transcripts/Patient 27 Male Age 26.docx]

**Patient 27: Age 26, HIV VL TB**

I: So you were telling us about your family members…

R: There’s my parents, my sister, my wife, my daughter and son at home. I have 3 brothers but they live outside. We are over here.

I: How about their families?

R: They aren’t married yet.

I: How old is your sister?

R: She is around 17 years old.

I: So what do you do for a living?

R: I drive heavy vehicles (trucks).

I: In which area do you work?

R: I drive trucks between Kolkata to Tata (Jamshedpur) and Ranchi

I: For how long have you been driving trucks?

R: For approximately 7 – 8 years now.

I: Do you do anything else apart from driving trucks?

R: I used to have a job as a welder in Delhi for my uncle. The work didn’t suit me, I preferred driving. I learnt to drive from my neighbour in Kolkata.

I: Your neighbour from Chhapra?

R: Yes. He’s <patidar>

I: You learnt how to drive in Kolkata?

R: Yes.

I: Did you directly learn how to drive heavy vehicles or did you start out with smaller vehicles?

R: No, no, no, it was directly trucks. The ones with 10 – 12 wheels.

I: Wow, that is nice. Since when have you been sick? When was the last time you were completely alright? Tell us in detail.

R: I was in Jamshedpur during *Sankaranti* (the harvest festival in early January). I took bath in a water body there. There were insects in the water but I didn’t pay too much attention at that time. The other drivers were taking bath there, so I took a bath and cleaned up my truck in the same water. That night, around 10:00-11:00 pm I started feeling cold. I bought some medicines but it didn’t help. I was sick for 2 – 3 days. I took medicines again and felt fine for a few days. I thought it was generalized weakness, so I decided to get an IV infusion done. When I came back to Tata (Jamshedpur) from Kolkata I had 3 bottles of IV infused. I then made another trip between Tata (Jamshedpur) and Kolkata. When I came back to Tata (Jamshedpur) my condition had worsened. One doctor told me that I had malaria, the other told me that it was not malaria. I went back home to Chhapra, where my condition become even more serious. I was not able to move around by myself. I was having fever alternating with chills. My body was covered in sweat as if I had taken a dip in water. When I got myself tested, I was told that I have Kala Azar.

I: What other disease did they tell you about? Apart from Kala Azar?

R: HIV.

I: I see. When did this happen? Which year?

R: This January. This year.

I: How many months has it been since then?

R: *(pauses to count)* It has been 3 months, ma’am.

I: You had no complains before these three months?

R: No ma’am, there were no complains before that.

I: Never at all?

R: No, not all. After a road traffic accident, I had tests done in Tata (Jamshedpur). The reports were all normal. I have been sick only in the past three months.

I: So your sickness has been very recent?

R: Yes, yes.

I: When you had the IV infusion and tests done in Jamshedpur… (cut off)

R: I have been anxious and sick since then. My body has not been keeping well.

I: Where did you first seek treatment?

R: I went to a private doctor in Chhapra, Dr. Shambhunath Singh. Then to Sahu *ji.* Then Tipu Jhadav called up the *Sadar* (District) Hospital and referred me to Mano *ji* there. He further referred me to RMRI, Patna.

I: So the tests – the blood tests, where did those happen?

R: At the District Hospital, Chhapra

I: And the other doctors you saw – Shambhunath Singh, Sahu *ji* and Tipu Jhadav – didn’t they conduct any blood tests?

R: They did conduct tests but then directed me to the District hospital without telling me anything.

I: They didn’t tell you anything at all?

R: No ma’am, they didn’t tell me anything.

I: About the disease?

R: They told me about Kala Azar but not about HIV.

I: When you had the IV infusion done (in Jamshedpur), were blood tests done?

R: Yes, but there they told me that it was malaria.

I: So when you were tested in Tata (Jamshedpur), they told you that you had malaria?

R: Yes ma’am.

I: And then they (local doctors in Chhapra) told you that you had Kala Azar?

R: Yes ma’am. They didn’t say anything about HIV. He asked my father to take me to the District Hospital.

I: Did they tell you or your father?

R: They told my father.

I: Then what did you do?

R: My father took me to the District Hospital. (05:50 – 05:52) He took me to Tipu Jhadhav first.

I: “Baat nahi patiyay” – what does that mean?

R: He (my father) thought they (the doctors) were lying.

I: He did not believe what was said?

R: No, he did not believe them. They told him that I had contracted HIV – AIDS and that it was because I had an extramarital affair. They told him that directly.

I: To whom?

R: To my father.

I: Who said this?

R: *(doesn’t understand)*

I: Who told your father that you had contracted HIV AIDS meaning that you must have had an extramarital affair?

R: The doctor did.

I: I see.

R: I said there was nothing of the sort. I have been married since 2012. I drive trucks for 3 – 4 months and then come back home for 2 – 3 months. I don’t know, maybe he was threatening me in front of my father.

I: Why did you feel that he was threatening you?

R: When he said these things in front of my father, I felt really guilty. I felt that I had done something wrong. He admonished me in front of my father.

I: What did you feel at that time?

R: I felt really bad at that time. He could have told me this when I was alone in the room, not when my father was around. I did not feel very nice.

I: Did you already know about this disease (HIV) when they told you that you had it?

R: I did know about HIV – that it happens because of *(hesitates)* extramarital affairs umm… that it spreads by touching / contact. In Tata (Jamshedpur), there is a company called TISCO – you must have heard of it. I would deliver goods there. So people from the government departments would come and conduct blood tests (for HIV)

I: And?

R: I did not test positive at that time. All this (the disease) has happened only over the last three months. When I would take goods for delivery to TISCO, a lot of government officers would come to check (for HIV). The doctors would come in Sumos and Scorpios. Posters would be put up (about : HIV). They would conduct blood tests. At that time nothing was found.

I: You got yourself checked then?

R: Yes. I got myself tested several times. I have been having fever since the day I took bath on *Sankaranti*. I was not sick before that. Now it has become such a big thing.

I: How much money have you spent seeing the private doctors?

R: Approximately ₹15000.

I: I see. So what is it that you are doing now?

R: I stay at home right now. One of my brothers is studying in Delhi. One brother is getting trained before starting work. Everybody depends on me. I am thinking of getting back to work as soon as possible. I will come here as when the doctors ask me to be here. I think I have become alright. I am taking the medicines regularly, ma’am.

I: Your father knows already because they told you in front of him. Have you told anyone else about your disease (HIV)?

R: I have told my brother. I told him that the doctors said that the disease spreads by contact/ touch. He said we would overcome this together.

I: How about your wife? Did she say anything? Has her behaviour towards you changed?

R: Her behaviour has not changed at all. It is just like it was earlier.

I: I see. Have you told anyone else in your family?

R: My mother, my father, my wife and my brothers are the only ones who know (about my HIV + status). No one else knows.

I: Did any of them ask you how it happened?

R: No, they didn’t.

I: Have you told anyone in the neighbourhood?

R: *(nervous laughter)* People look down upon those who have the disease. That is why I have told only my family about it. I haven’t told anyone else about it.

I: But you have told your family about it?

R: Yes. My mother knows, my father knows, my wife knows. Even my sister knows. But I haven’t told my neighbours or anyone else. That I have this disease.

I: Is there anyone in the neighbourhood who has a disease like this?

R: I don’t think so. Even if someone has it, they won’t tell me, will they, ma’am?

I: So even you don’t know about it?

R: No, I don’t know.

I: Why do you think that they won’t tell you?

R: This disease is such that um… people will not even um… come close to you if you tell them that you have it. Even if someone in my neighbourhood has it, they won’t tell me.

I: Do you think this happens with Kala Azar too? Or is it just with HIV? That people won’t sit with you? Or that you will be outcast?

R: Just HIV

I: How about Kala Azar?

R: Kala Azar is just a type of fever. If someone has it then we can tell Mano *ji* at the District Hospital. He will bring us here for treatment.

I: So there is no stigma associated with Kala Azar? It’s only with HIV?

R: Yes, the stigma is associated only with HIV.

I: And the third disease that you have (TB)?

R: There is no stigma associated with it. It is air borne. I take medications for it. The doctors talk to me too. They told me that the medication has a six month course.

I: When did you get to know about the third disease (TB)?

R: I got to know that I had Kala Azar, HIV and the third disease (TB) all at the same time.

I: Where did you get to know that?

R: Here only.

I: At RMRI?

R: Yes.

I: But you told us that they told you about HIV and Kala Azar at the District Hospital itself?

R: But they didn’t tell me about this disease.

I: Did they test you for TB in the District Hospital? In Chhapra?

R: *(long pause)* They conducted all tests – ultrasound, X-ray and everything.

I: But they didn’t tell you about TB there?

R: No, they didn’t tell me about TB. They did tell me directly about the HIV though.

I: Yes, you told us, they said it in front of your father.

R: The doctor told me I have TB.

I: But you’re saying that they didn’t tell you about the TB diagnosis at the District Hospital?

R: No, they didn’t tell me about TB. They did tell me directly about the HIV though.

I: And Kala Azar?

R: Yes, they told me that. I remember clearly that they told me about the TB diagnosis only here (RMRI). Maybe they told my father, I do not know. But they didn’t tell me.

I: Do you information about TB? The causes, what it is?

R: The doctors told me. They told me to cover my mouth while breathing around other people. There is a possibility of spreading the disease to the other person if my breath enters their body. I don’t have any other information ma’am.

I: I see. Did you have any other complains? Decrease in weight perhaps?

R: My weight had reduced to 50 kg by the time I was at the District Hospital. Now that I am taking medications, my weight has increased by 7 – 8 kg.

I: What was your weight earlier? Before the disease?

R: My usual weight is around 70 kg. It reduced to 50 kg.

I: In how many days did it reduce to 50 kg?

R: Approximately 2 months. I would feel extremely cold. My mother, my wife used to hold me tightly, but even then it did not work. When I used to get chills, I had very high grade fever.

I: How about the sweating?

R: It would feel like I was drenched in sweat.

I: Would it happen in the morning or at night?

R: Both times. There was no guarantee of this. I would eat only two *roti*-s. I had lost my appetite. I would not be able to walk to the bathroom by myself.

I: So your family had to help you with that?

R: Yes, yes. I was in a bad condition. Thankfully I am better now, with God’s grace.

I: Did you have any complains of coughing?

R: Yes, I did. But it subsided when I took the medicines.

I: Did you ever cough out blood?

R: No, that has not happened yet.

I: After having lost so much weight, did you ever look at yourself in the mirror?

R: My face had blackened.

I: How would you feel upon seeing your reflection?

R: My eyes had sunken. My hands become very thin and wiry. I did not like looking at myself.

I: I see. Did you feel bad?

R: Yes. I felt bad.

I: According to you, what are the things required to lead a good life?

R: *(long pause)* If you have to lead a good life, with family, then one must use a condom. If you want to ruin your life, then don’t use it.

I: Anything else?

R: I wish that I earn well and my family can be happy and prosperous.

I: Is money important to lead a good life? How important is it?

R: Yes, it is important. Money, ma’am *(laughs nervously)*, how can I tell you?

I: Why not? You must be having something in your mind?

R: I have one son, one daughter and one sister, ma’am. If I were earning right now, I could save up for their weddings.

I: And your children? What do you wish for them?

R: If I were earning right now then I could have thought of getting a life insurance for them.

I: Why do you think this way?

R: No one knows what the future holds. That is why it would have been helpful.

I: So you want get a life insurance on their name? How will that help?

R: It will help by the time they are of marriageable age.

I: How so?

R: I can save up money for their wedding.

I: Anything else that you deem necessary to lead a good life?

R: What else would be necessary ma’am?

I: Like treatment?

R: Yes ma’am, good treatment is necessary.

I: Why do you think so?

R: The doctors told me to take my medication regularly – should not be missing even one dose, otherwise I would regret it.

I: How satisfied are you with the healthcare that you are receiving?

R: I am satisfied with it.

I: When you got to know that you had Kala Azar and HIV, did you feel inferior somehow? Or did you have any feelings of hopelessness?

R: Yes, I did feel that there is no hope left now that I have contracted HIV. As a child, I used to hear of people dying of AIDS because medicines for its treatment were not available at that time. I did not have information that there are medications available for it. I thought I would not survive. My father told me that medications are now available for it. The doctors told me not to worry – that I would not die but I would have to take medicines lifelong. I found some consolation in the fact that I would not die if I took the medicines regularly for the rest of my life. Otherwise I would die within three – four months.

I had no disease or sickness before this. It all happened in these three months only.

I: Have you ever contemplated committing suicide?

R: No, no, no.

I: Have you ever had kind of suicidal thoughts?

R: No, I don’t have such wrong thoughts.

I: Did they first tell you about Kala Azar or HIV?

R: They first told me about Kala Azar, then HIV. My liver and spleen were enlarged.

I: Anything else that you think is important for leading a good life? A house? Or anything else?

R: The doctor told me to be careful if I wanted to see my wife and kids.

I: How is the house that you are living in right now? Do you have your own house built?

R: Yes, I have my own house. It was built around 2000.

I: Is it a *pakka* house?

R: No, the plaster work is incomplete. But it is made if bricks. My father built it. My father has five brothers, but now everyone lives separately.

I: How has the disease affected your work?

R: There is nothing that I think I cannot do. I think I will be able to do all the work properly. As long as I take my medicines regularly, I will be alright. The day I stop taking medicines, I will fall sick. So I will not stop my medication. I will take them regularly, without fail.

I: So you think it will not have an impact your work?

R: No, it won’t.

I: What are doing currently?

R: I am not working right now.

I: How are you meeting your expenses then?

R: Um… that… *(laughs)* I have taken some money from my (paternal) uncles. My father also works, back in the village. He earns around ₹800 – ₹900 monthly.

I: How do you manage in ₹800 – ₹900 monthly?

R: There is the ₹800 – ₹900 that comes monthly. We own some cows. The vegetables and spinach grows on land. I had brought some money with me when I came back home three months ago. That is why I am thinking of going back to work. I will ask the doctors soon. They have called me for a review on the 18^th^. I will confirm with them and then go back to work.

I: Do you think there is anything that you earlier thought of doing but won’t be able to now?

R: I used to think that I wouldn’t be able to do a lot of things earlier. But now that I have become much better, I think I will be able to do everything that I need to.

I: How has your sleep pattern been?

R: I would feel sleepy all day long. For the past three months. It was not always like this. Earlier, however late or early I slept, I would wake up at 4 am in the morning.

I: And during the disease?

R: I would feel very sleepy throughout the day. It was difficult to stay awake.

I: Were you able to visit your friends when you were sick?

R: No, I was not able to. After I came back from Kolkata, the treatment and check-ups in Chhapra took me 17 – 18 days. Then I went to the District Hospital. My parents had to carry me on their laps on the passenger trains when we had to meet the doctors. My condition was bad.

I: What do you think of the treatment that you are getting here?

R: I am satisfied with it.

I: Tell us in more detail?

R: I have been treated well here. I can walk on my own now. The things that I was incapable of doing because of my disease, I can now do.

I: Incapable of?

R: I was fed up and distressed because of my diseases earlier. Now I face no problems. Earlier I was not able to even walk by myself. Now I can. I was not able to eat two *roti*s earlier but now my appetite is back to normal. I am eating 4 – 5 *roti*s. I have been feeling better since *Holi* (a festival in March). I have been feeling much better since the day that I have stepped into this place.

I: How has the behaviour of the staff here been with you?

R: They have been very nice to me. The doctors, nurses, everyone.

I: Is there anything you want to be done different?

R: No, everything is great. The doctors are good. They look after everyone equally.

I: Have your brothers treated you differently because of the disease?

R: No, there has been no difference in their behaviour to me. It is all good between us.

I: Nobody in the family has behaved differently with you?

R: Yes, everyone is good with me. They believe that what has happened has happened.

I: What do you expect from your life now? What kind of a job do you want to have?

R: I want to continue driving trucks ma’am. I don’t like any other job.

I: What else do you think you will do if your health supports it? Why do you want to drive trucks?

R: I want to drive trucks to support my family – my parents, my wife, my sister, my son, my daughter. I will save some money for their wedding. My brothers might move out when they get married. That’s why I want to save up money on my own.

I: In these days of hardship, who were the people who supported you?

R: My entire family was supportive. My father, my mother, my wife.

I: And the uncle who helped you out financially?

R: Yes, him too. He told me that he would help us with all the money that was required. But I told him that I had some money right now, I would not need too much.

I: What does your father do?

R: My father worked in Nepal earlier. When the earthquake happened? In Nepal? Till then he knitted clothes there.

I: In Nepal?

R: Yes.

I: Till 2015:

R: Yes.

I: And after that?

R: He was sent back home after that. He now takes care of the fields.

I: Do you have your own land?

R: Yes. We have one *bigha* (about 5/8^th^ of an acre)

I: What do you cultivate there?

R: Wheat and rice. We have let it out to others.

I: So you get enough to rice and wheat from the land? And vegetables too?

R: Yes. We have to buy the vegetables and spinach sometimes though. We don’t have to buy wheat and rice. We have to buy oil too.

I: Anything else that you would like to tell us about yourself?

R: I don’t know what else to say, ma’am. You tell me *(laughs)*

I: Anything else that you would like to tell us about yourself? Or about your experience? The impact that the disease has had on your life?

R: I don’t understand how I am supposed to behave with my wife ma’am. If you could explain that to me? They told me when I came here for treatment, but if you could explain too.

I: So when you leave here after the treatment, you will go back to driving trucks?

R: Yes, ma’am. Now I think I can go back to doing everything that I want to do. My strength is back.

I: Thank very much. You have given us enough information today. Thank you.
