# Supplementary material for: Quality of life perceptions amongst patients co-infected with Visceral Leishmaniasis and HIV: A qualitative study from Bihar, India
Source: PLoS One. 2020 Feb 10;15(2):e0227911. doi: 10.1371/journal.pone.0227911 (PMC7010301; doi:10.1371/journal.pone.0227911)
Supplement: S3 File — (ZIP) [file pone.0227911.s003.zip › Transcripts/Patient 28 Female Age 45.docx]

**Patient 28 Age 45 VL HIV TB**

R: 2 kids. The girl is the elder, so the people from the community are teaching her.

I: Meaning, (what do you mean by) people from the community?

R: Yes like now that she is going for coaching, learning computers, she is getting *help* from the community.

I: That’s what I am trying to ask. Who is helping? Is it one particular person?

R: Like the teacher agrees that our (financial) condition is not good.

I: Okay so the teacher is not charging any fee?

R: No.

I: Anyone else who is helping in her education?

R: My parents give us Rs. 500-1000 to meet our basic needs.

I: You mean your father-in-law and your father?

R: My father. My father-in-law is old and sick he doesn’t keep well.

I: So he doesn’t help you out?

R: No. We are 4 sisters. I am the smaller one. So I am the smallest right?

I: Meaning you are the only sister?

R: No there are 4 brothers and I am the wife of the smaller one. None of the other 3 take care of our father-in-law, we have to do it.

I: You mean that the other 3 sons stay elsewhere, your father-in-law stays with you?

R: The in-laws stay here but not with us (in the same house).

I: What help do you get from their side?

R: No thing. Nothing at all. (in a low tone). The situation is tricky. Tricky.

I: So how do you make your ends meet?

R: I can’t do any work. Since the illness my body has become weak and my body can’t work anymore.

I: Okay. Were you doing something earlier?

R: No.

I: Okay then how did you make your ends meet that time (earlier)?

R: Dad and Mom send us 1000-500 every month. We get rice, daal, wheat etc. from the government.

I: So the government gives you?

R: With the aadhar card... We have our name in BPL and we get it with the ration card.

I: In BPL? So when your husband was alive, how did you... how long ago did he die? How many days ago?

R: 2012.

I: Can you tell u something about it?

R: 7 years have passed.

I: What happened to him, how did it happen, can you tell anything?

R: First he was in Delhi in...when he was unmarried... sorry, in Mumbai. After marriage we started living in Delhi. First he got TB, a long time ago. After TB he got kala-azar. We went to a private clinic but nobody could detect anything. So we brought him to Darbhanga from Delhi and a private doctor did a full check-up and HIV was detected. He sent us to DMC. When he got checked in DMC in the govt., there was confirmation of AIDS. So, his condition was really bad. People from the village started saying that he won’t be able to survive. When the kala-azar medication was started after the earlier detection. It didn’t have any effect on him. Somehow he stayed bedridden for 6 months. He couldn’t do any work. His hands and feet became completely loose.

I: Loose? In the sense that they became thin?

R: I mean, he couldn’t manage to stand anymore. If you took him to the bathroom, he wouldn’t be able to get his feet up. Here nobody could see him so we took him to Patna and he got admitted in DMC. They said they’ll send him to Delhi. We found out the details, but he wasn’t in any condition to travel. Sir told us we could take him, but he wouldn’t survive, we should rather take him home. He died at home. We took him home, he stayed bedridden for 10 days, then died. After 19 days he broke down and his condition worsened.

I: This incident is from which year? Like 2012 was the year of his death. When did all his diseases and the subsequent chain of events start?

R: It started in April and after 6 months, by June-July he was dead.

I: The whole thin lasted for how long?

R: He lasted around 6 months. Like April is going on, say on the 8^th^ he came home, (after a pause), by August, he was dead.

I: First you said he was diagnosed with HIV in Darbhanga. Later on you talked about AIDS. Do you know what AIDS is, how is it different from HIV?

R: No... After they told us about HIV in the private clinic...

I: Where?

R: In Memorial hospital.

I: In which city?

R: In Darbhanga.

I: RB Memorial Hospital. Later on, we were sent to a govt. setup where we were told the disease was *heavy* (severe), it had taken the form of AIDS from HIV.

I: You said everyone started saying, who were these people? When you said everyone started saying (he won’t survive)?

R: The people from the society, those from the olden times, they said that people don’t survive this disease.

I: People around you?

R: Yes. I being a new person didn’t understand what they were talking about.

I: Was a similar thing happening to other people in the village too?

R: No, I hadn’t seen anything earlier, but after seeing git for the for time even I was astonished. That what kinds of disease is this which even medicines can’t cure.

I: Did you have any check-up at that time?

R: Yes, I was told by the doctor at that time to get a check-up done.

I: So can you tell us about your problems from the start?

R: When we were in Delhi, I used to feel cold, so my husband came home to give me medicines, but no medicines suited me, from that time, his body became very weak, couldn’t get up, as if all his strength had finished.

I: We want to know about your illness.

R: I also started getting a fever around 2012, sometime after my husband. Fever, cough, everything started happening.

I: So you were all right before this time?

R: Yes.

I: Tell us more about your illness.

R: When my husband was admitted in Darbhanga, all the doctors kept telling me to get myself checked. Even though I was having to already take care of a sick person, I still got myself checked and tested positive for HIV. I was told to go Dhobi Ghat, here there etc.

I: Why? What’s there in Dhobi Ghat?

R: Check-ups. I had to undergo 10 types of check-ups. I didn’t go but they still said I needed more check-ups, like sputum check-up and then TB was also detected.

I: In 2012 itself?

R: Yes. I started the medications.

I: Which disease did you get to know about first?

R: Kala-azar.

I: After that?

R: Then I took the medications, but during the time my husband’s condition worsened, I left taking the medicines.

I: So you left the medications. Since when are you taking the medicines for Kala-Azar?

R: From that time only, when I visited multiple hospitals.

I: Where? In Darbhanga?

R: In the govt. setup, there is one in manigaichi..

I: Ohh, your village manigaichi?

R: Yes, there after a hospital they said its kala-azar and gave me medicines.

I: After kala-azar what happened?

R: Here in DMC in Darbhanga I got tested for TB and it was then detected. HIV was also detected.

I: Out of HIV and TB which was detected first?

R: First HIV, then TB. My body became weak and I started the medications.

I: What problems were you facing after HIV?

R: Fever.

I: What kind of fever?

R: Like the normal fever one gets.

I: I mean, did the fever stay all the time? Or it was on-off?

R: On-off. It used to subside on taking medication.

I: Did you feel anything else too along with the fever?

R: No. A normal fever only.

I: Any other difficulties? Weight, appetite?

R: Appetite was normal. I wasn’t weak at that time (answering about weight loss)

I: Night sweats, out blood in sputum?

R: No. Nothing.

I: How was your mental status? Were you sad?

R: I had tension at that time since my husband passed away during that time. My heart rate had increased (palpitations?), I had stopped taking my medicines.

I: Why did you stop taking medicines?

R: Many things…I thought what happened? There was no one else to take care of the household. The children were still very young and naïve. Then slowly I started taking the medications and my health improved. For 2-3 years… The TB medication I took for around 6 months and then stopped... then I had to take it once again.

I: So when the TB medications stopped, when did you start getting your current problem? What happened right now (current symptoms).

R: Right now there is fever, weakness, chills.

I: Since when is this happening?

R: I’ve been admitted here for the last 1 month.

I: Since what time (have you had these problems) before coming here?

R: Around 10 days before coming here. I started having a swelling and feeling tightness.

I: What did *you* feel? Someone else would have told you about the swelling.

R: Like I would feel cold and then get a fever. I didn’t feel like eating anything. My breathing started becoming harder as if I was reverse breathing. I also had problem speaking. My daughters said since I already had had Kala-Azar once, I should go to Patna once. I became alright after coming here.

I: You had already come to RMRI once?

R: Yes.

I: When?

R: This is my 3^rd^ visit. Once in December, then in August…

I: Since how many years are you coming here?

R: 3 times in 1 year. Since 1-1.5 years.

I: The breathlessness you are talking about, did you have any other difficulties too, fluid in the chest? Were you told anything here? Did they do an X-Ray or anything?

R: They didn’t do an X-Ray. A blood test was done and a sputum test was done. TB was detected in that.

I: When you were first told in Darbhanga that you had HIV, what did you feel at that time? What did you think?

R: That time I didn’t feel anything. My health was also good. Everyone told me I’m well and will live for another 10 years. TB was detected but its medication had been started. This time by I started feeling breathless.

I: Did you feel anything that time after hearing about the name *HIV*?

R: I thought it was nothing because I wasn’t feeling any problems. Now I am feeling a little bit.

I: Since your health was alright at that time you didn’t worry much?

R: Yes. If there is strength in the body, one feels alright... but then if one gets fever now and then... like this is my 3^rd^ time...

I: How many people have you told that you have HIV? How many people know about your condition?

R: Slowly everyone gets to know about things like this. Like… after seeing my husband’s condition everyone in the neighbourhood came to visit him, after the diagnosis at DMC Darbhanga and his subsequent condition, everyone got to know that he had this disease (HIV).

I: What did the people say during that time?

R: They all just said that he had this disease (HIV).

I: Did their behaviour towards you change in any manner?

R: Yes, there were some foolish people who said things like they’ll not inject any needles, or stay around us, they’ll not eat from the same plate, not share the same bed. I felt a little sad hearing all this.

I: What about the other 2 diseases? What did the people say about them?

R: They said that these are *heavy* diseases and that people don’t survive for long. The blood had become very bad.

I: Any change in behaviour regarding these 2 diseases? Was there any difference in behaviour as in the behaviour of the people if one has HIV compared to their behaviour if one has TB/Kala-Azar?

R: Who?

I: The behaviour of the people regarding the disease.

R: No there was no difference.

I: Do your children know about HIV?

R: Yes, they understand.

I: The daughter and the 2 young sons too?

R: Yes.

I: Any change in their behaviour towards you that you have noticed?

R: They are scared of blood-borne contraction of the disease. That blood shouldn’t be contaminated with other blood.

I: Your daughter’s behaviour towards you is all right?

R: Yes it is all right. They are children so it is all right (she means they don’t understand much?)

I: And you’re other relatives (in-laws)?

R: They say things like not to eat from the same plate as mine, not to sit with me, that I have a disease.

I: Do they visit you?

R: Yes, they visit me. Even I visit them.

I: Where, you said that they lived somewhere else. Where do they live? In the same village or somewhere else?

R: No in the same village. Everyone has got their share and lives in separate houses with their families.

I: Do you guys meet?

R: Yes, during functions like marriages and parties, we meet. They visit my house, I visit theirs too. There is no block (disharmony?). Only thing is that when taking medications if there is some need le to be injected, then they say they won’t do it.

I: Who says so?

R: The compounder in the village.

I: Even they know (about your HIV)?

R: Yes, everyone knows.

I: To live a good life, what all do you think is required?

R: You can say better what can I say. I think that my suffering should go away and that I can live with my children happily.

I: What would you like to do for your children?

R: Any rift should go away, we should eat well, they should get help in their education, we should live properly, and I would want all this only... what else would I want?

I: What kind of house do you have?

R: She has a thatch house

I: What kind of support do your parents provide you?

R: What can one possibly do to someone who is so sick?

I: Still, like you said earlier they give money...

R: Yes, like that only sometimes 500 sometimes 1000.

I: Do they visit you? Where do they live?

R: They live in the village.

I: Do they visit you or you visit them?

R: Yes, I visit them but they don’t visit me. I go to them and give-take things (money?)

I: Their behaviour towards you?

R: They make me eat, sleep separately.

I: There also?

R: Yes.

I: How do you feel about this?

R: I “*feel*” this.

I: you feel this?

R: Yes. That what is the disease that everyone seems scared.

I: Even though you know that the disease doesn’t spread like that. I mean, you must have been told at RMRI about how the disease spreads?

R: Yes. But still people are ignorant, they remain scared, even though we are well informed that it spreads in a blood-borne manner, not from eating/sleeping together. Still, people stick to whatever beliefs they have in their minds and say that this is a bad disease.

I: When your husband was admitted in the hospital and you were having to incur treatment expenses, who helped you that time financially, or in some other way?

R: It was totally financed by my father-in-law how sold some land to arrange for the money. For both the private and govt. treatments. But he too couldn’t handle it completely (regarding the disease being cured)

I: So he helped?

R: Yes, he spent 5000-10000 but the condition (of her husband) kept on deteriorating. He became completely *loose.*

I: What about your medical expenses?

R: No. After my diagnosis of HIV, I’ve received medicines from DMC>

I: Before that, when you were diagnosed with Kala-Azar?

R: After the diagnosis at a private clinic, I took some medicines here and there, then it was confirmed at a govt. hospitals and from then I received medicines form there.

I: How much did you have to spend for the check-up of Kala-Azar?

R: No, I bought some medicines and ate. No, after the HIV diagnosis, the TB check-up was done at the Govt. hospital and after that I have been taking medicines from there only (DMC?).

I: Do you think your life has been affected due to HIV? Or all the 3 diseases?

R: I become very week from time to time, then with time, around 6 months later I again feel okay.

I: Regarding TB? Has TB made you feel any different?

R: The same problem (weakness?) was there but it isn’t there presently.

I: Like you talked about the behaviour of the people regarding HIC, do the people think similarly regarding TB?

R: People know that I am a TB and HIV patient so they talk things, but can we really stop them?

I: What would you want them to think?

R: I would want them to think about how I should live safely. What else can I say?

I: Tell us about your treatment here. How did it go, what all was done.

R: It was good. As soon as the diagnosis was made, the treatment was started.

I: How long did they keep you here?

R: 1 month.

I: Can you tell us what all went well, what else could be improved?

R: I feel weak from the inside. I have 3 diseases. But compared to the state in which I came here, I am fine now.

I: How was the behaviour of the staff towards you?

R: Okay. After the tests all the subsequent measures, diet, I got from here. Whatever was to be monitored, the sirs here monitored.

I: Who accompanied you from your house?

R: My son.

I: How old is he?

R: 18y old.

I: What does he do?

R: He is doing his intermediate.

I: In your village?

R: Yes, but he came to the hospital today.

I: What about the educational expenses of your sons? Like you said your daughter is receiving help from her teachers.

R: My younger son is in 7^th^ grade. The elder one passed 10^th^ from a govt. school and has now enrolled in intermediate.

I: Their educational expenses?

R: He goes to Delhi, study and do some jobs and earn 500-1000.

I: What jobs does he do?

R: He works as salesman. Running around here and there.

I: The younger son?

R: He is studying.

I: Has the illness affected your day to day activities?

R: I can do all my work, but after walking for some time I become breathless and get palpitations.

I: What do you do then?

R: I work at home. Cooking food, cleaning, washing dishes, washing clothes.

I: You do all this even in this state?

R: Yes, all the daily chores like cooking, cleaning, getting the kids ready for school etc.

I: So you are doing everything?

R: Yes, I don’t realised I am ill till the weakness hits me from the inside. Like, I take care of my home just like other healthy people.

I: So, right now due to the disease, what difficulties are you facing?

R: Breathlessness, weakness, fever, the kala-azar fever gave me chills and rigor, after which I came here and took treatment and got better. This thing right, this spleen? This always gets bigger. If it doesn’t get big, then I’m good- I’m strong from within- it always increases and that’s why it is a problem.

I: Where do you get your medicines from?

R: From DMC Darbhanga.

I: What is the travel expense from your village to there?

R: Around Rs. 100.

I: To and fro combined?

R: 150-300.

I: 300? Every time?

R: Yes, every month.

I: How many days’ medicines do you get?

R: 1 month.

I: Would you like some change in the treatment that you are receiving for HIV or TB or Kala-Azar? (Explains what *change* is being referred to)

R: Even if I want to, the disease (HIV) won’t go away. I’ll have to keep going and taking medicines. I would like to be free of the Kala-Azar medicines. It should not recur. I am staying away from my home, my family for 1.5-2 months for the treatment. I would want that I don’t get TB again.

I: Anything else you would like to say about the treatment you are receiving at RMRI or DMC?

R: No, what can I say. One has to do what has to be done.

I: As per you are all the facilities you are receiving all right, or do you think you should be receiving something more?

R: From where will I get madam? We have a lot of deficiencies (in facilities) but from where will we get them?

I: (long pause) during the disease what did you think about the future?

R: What will happen how will I know? Whether my condition will deteriorate or stay the same. I obviously fear that when time comes if I will completely fall (bedridden?). But these things only God knows, it is not in our hands. I only hope that my condition should not deteriorate from the current situation. It is a lot already.

I: Moving forward, what do you expect from life?

R: I want happiness. What else?

I: What is this happiness according to you?

R: Happiness regarding everything. I don’t have a home, I have to get my daughter married, how to stay healthy, the future of my 2 sons, happiness of a lot of different things is there, but from how will I achieve them.

I: When you got to know about your disease, did you ever contemplate suicide, or question your will to continue living?

R: Yes, these things keep coming to my mind. If my daughter had gotten married and I would’ve died, it would have been nice. What will I do by living and bearing so much pain?

I: You get such thoughts?

R: Yes, why wouldn’t they come?

I: What all do you think regarding this?

R: Till I am alive, I have to keep running around. Till what time can I sustain this? Till old age? If I were healthy and if it were good times, it would be good for everyone…there should be treatment. Everyone thinks this. If it happens, then great. The disease should end from its roots, then people’s time will pass nicely. That’s what I think. If it doesn’t then anyways I have to suffer my current life.

I: Anything else you would like to say?

R: All I would like to say is that treatment should happen on time and (I?) should be HIV-free.

I: TB and Kala-Azar? You don’t pray for a similar things for these diseases?

R: Yes, I would like to be disease-free.

I: (Laughter) you said so much about HIV but TB has given you more problems. You said you had it 3 times.

R: I had Kala-Azar 3 times too.

I: So what about these?

R: It all comes from there (HIV) only. People say HIV is the main cause. So I want to be HIV-free first.

I: Have you completed the TB and Kala-Azar medication?

R: 1 month treatment of Kala-Azar is complete. For TB they have given med medicines from DMC and they will test for 2-3 months.

I: Did you get all the medicines together from DMC or you will have to go there every month?

R: I get from DMC only.

I: No, I mean how do you take the medicines? Do you go to DMC and take them or they have given it someone in your village?

R: From ART.

I: So you have to go one time only? For both HIV and TB?

R: Yes. Like after the completion of a course of 1 month I get the next month’s medicines from the hospital.

I: You get both the medicines together?

R: Yes. Now that the TB has recurred, I have to take an injection and pills. Both. I will have to go home. I will be discharged today. So, there (in my village) nobody wants to apply the injection so I ask the doctor to not prescribe injections but they are adamant and say it is necessary.

I: So how do you manage this situation?

R: I will go to Darbhanga and tell them that I am a housewife, to change my treatment or do something. If they don’t there will be gaps. I’ll receive the injections sometimes and sometimes I won’t.

I: How frequently do you have to take injections?

R: First time I had to take pills for 6 months. Then after it came back I did a 9 month course. 1 strip, and when you finish that, then one injection.

I: That injection had to be taken from Darbhanga or you could take it from anywhere?

R: The needles and pills were provided by Darbhanga DMC. At home I had to continue taking the pills and getting the injections on my own.

I: Okay, so the injections which you are to take at home, nobody is willing to inject them?

R: No. They treat everyone at the hospital, but at the village it is different.

I: So you *are* wanting this to change right? If getting injections are so hard at your village, then what will you do?

R: I will go to Darbhanga and talk to the doctor.

I: What will you tell him?

R: I will tell him about the situation. If he provides a solution, then it will be good.

I: Anything else you would like to say?

R: No.

I: Okay. Thank you.
