# Supplementary material for: Quality of life perceptions amongst patients co-infected with Visceral Leishmaniasis and HIV: A qualitative study from Bihar, India
Source: PLoS One. 2020 Feb 10;15(2):e0227911. doi: 10.1371/journal.pone.0227911 (PMC7010301; doi:10.1371/journal.pone.0227911)
Supplement: S3 File — (ZIP) [file pone.0227911.s003.zip › Transcripts/Patient 29 Male Age 37.docx]

**Patient 29: Age 37, HIV VL TB**

I: What do you do for a living?

R: I am a driver.

I: What vehicle do you drive?

R: Bus.

I: In between cities? Or in the same city?

R: In the same city. From my home to Motihari.

I: From your village?

R: Yes

I: Is it your own bus?

R: No, it is someone else’s bus.

I: For how long have you been experiencing these problems (of ill health)? Tell us everything in detail, from the beginning.

R: I was perfectly fine two years ago. I would drive all kinds of vehicles. After my wife died, I fell down once. I was treated by a private doctor. Nobody was telling me what was wrong so from Sheohar I came to Patna.

I: What were your complaints at that time?

R: I used to have fever, fatigue and weakness. I had lost my appetite. I no longer have these complaints (after coming here for treatment).

I: Were you always running a fever? Or at particular times?

R: All the time.

I: Did you ever suffer from excessive coughing?

R: Since childhood.

I: Do you smoke?

R: No, I have no addictions. I used to drink but stopped four years ago. Otherwise I have had no other addictions.

I: So you’ve told us that you had fever, fatigue, weakness and loss of appetite. Any other complains?

R: No, no other complaints.

I: So what did you do next?

R: I spent a lot of money on private treatment.

I: About how much money did you spend?

R: About Rs. 60,000 – Rs. 70,000. When I went to Sheohar, they referred my wife and me to Muzaffarpur.

I: What disease did your wife have?

R: The same one.

I: What was the name of the disease?

R: (*Hesitates)* The one that is spread by sex, you know.

I: HIV?

R: Yes. That one. She was very sick. I got her treated for a long time. They referred her to Muzaffarpur PHCH *(sic)*.

I: Where in Muzaffarour?

R: PHCH.

I: Where did they refer her to? Was it a government hospital?

R: Yes, yes.

By the time I got her there, my wife passed away. Then I was left alone with the children – they are very young. My health was failing.

I: Did you both fall sick at the same time?

R: Yes, we fell sick at the same time

I: Was she completely fine before that?

R: Yes, she was fine.

I: Your wife, was she completely healthy before that?

R: Yes, yes. When she started having fever, I took her to a private doctor. I spent a lot of money on her treatment but she did not get any better. They referred her to Sheohar where they referred her to Muzaffarpur because she was not getting any better.

I: Why did you first go to a private doctor first? Why not a government hospital first?

R: Doctors don’t see us quickly at government hospitals there. Like here, I got saved because I got treatment on time. But there, I don’t think so.

I: Oh, so that’s why you went to a private doctor first?

R: Yes.

I: So when you reached the government hospital at Muzaffarpur…

R: I couldn’t reach there on time, my wife passed away before that.

I: I see. So what did you do after that?

R: I had to take care of my young children by myself. Then I had a high grade fever – my whole body was trembling.

I: How long after your wife’s death was that?

R: Six months later. Or you can take it as 2 – 3 months later. I would be coughing even in the night. I had severe fever. Then I went to private doctors in Sheohar. They conducted tests and told me that I had Kala Azar. They referred me to Motihari. Jitender *ji* from Motihari referred me here (RMRI, Patna). By the time I reached here, I was bedridden. No one thought I would survive. I have been on treatment and medication right from the day I reached here. I also got food on time. My troubles have been wiped off.

I: So where did you get to know that your wife has HIV?

R: A private doctor told us.

I: Were you tested too?

R: Yes.

I: What did they tell you?

R: At that time I was told that I did not have it (HIV)

I: I see. So you did not have HIV?

R: No. One child, my daughter, she didn’t have it that time. But now she has it too.

I: Your daughter has HIV too?

R: Yes. The doctor at Sheohar asked me to get tested. And my children too. Otherwise my other three children are fine.

I: She is your eldest daughter?

R: Yes

I: How old is she?

R: She is 16 years old.

I: When you heard all of this, how did you feel?

R: I felt weak. I told others that I did not have the disease.

I: Why did you think that you did not have the disease?

R: I did not have trouble with my daily activities, so I didn’t understand. Only when the doctors in Motihari and Muzaffarpur told me I decided to go to Patna at once. I was also told that I had TB.

I: Where did you learn about the TB diagnosis?

R: The doctors got an X-ray done.

I: There were no tests done before that?

R: No, no where. TB didn’t appear on any report before that.

I: So about which disease were you told about first?

R: Kala azar.

I: Then?

R: Then I was told of this one…

I: HIV?

R: Yes.

I got to know in Sheohar. In the government hospital. Then in Motihari they gave me medicines. I brought those along here.

I: How has all this affected your work?

R: I was able to do my work but I would feel very tired and by the time I got home I would develop a fever.

I: Are you able to work right now?

R: I am still admitted in the hospital. But with your blessings, this place is not a hospital, it is a temple for me. Everyone here is very nice.

I: In the two years that you have been sick, what has the status of your work been?

R: I had to take care of my family, what other option does a poor man like me have? I did not have enough information about hospitals and medical treatment, that is why I did whatever other people told me was best.

I: Anyone who supported you through all this?

R: No, no one. There is no one in my wife’s family to take care either. They’re all dead. My father has died. Only my mother is alive. There is nobody else in my family.

I: Any siblings?

R: One brother. But he lives elsewhere. I only have my three daughters with me.

I: Did you tell anyone about having the disease when you got to know? Your neighbours or friends?

R: Yes, I did tell them.

I: You told them by yourself?

R: Yes, I told them myself.

I: What did you tell them?

R: That I had contracted this disease and that I was going for treatment to Patna. I asked them to take care of my house and other things till I came back.

I: About which disease did you tell them?

R: Kala Azar.

I: HIV?

R: No.

I: You didn’t tell them about HIV?

R: No.

I: Does anybody in your neighbourhood have HIV?

R: 2 – 4 people have Kala Azar. My father died of Kala Azar. My *bhau* ( ) died of it too. I thought everyone is dying of the same disease. That us why I came here for treatment quickly. You will find a lot of patients of Kala Azar there.

I: A lot of patients?

R: Yes. Everyone has high grade fever, everyone is dying of it. The hospital there is such that you have to wait in line for a long time to get registered. They don’t give proper medicines either. No one looks after the patients properly.

I: Where you able to do your work properly, with all the weakness and fever?

R: I had to somehow manage to earn my daily wages. I was driving slowly. It was a school bus.

I: Were you able to do these during the course of the disease?

R: Yes, I was able to. But for around 3 months when I was severely sick, I was not able to work. Then I had to start taking treatment in Sheohar.

I: Before that where did you seek treatment?

R: Local doctors. That did not help me at all though. Multiple blood tests were done. I spent Rs. 12,000 – Rs. 13, 000 on tests, but in vain. They said it’s just fever, it will resolve on its own. It would reduce briefly when I was given medicines and injections. But I would always be tired.

I: Were you able to sleep well?

R: Yes, I had no problems related to sleep.

I: Did you notice any change in your sleep pattern? Was it increased or decreased? Any changes at all?

R: No, no. There was no change at all.

I: Did you have pain anywhere in your body?

R: No.

I: Did you know about this disease earlier?

R: No.

I: About HIV?

R: No. I knew about Kala Azar. No one told me about it earlier. When I went to Sheohar for treatment, that is when I learnt about this disease from the doctor.

I: Are you satisfied with the treatment here?

R: Yes, yes, I am very satisfied with the treatment here. I feel much better now. I feel my strength has returned.

I: Did you notice any change in the behaviour of your neighbours or friends towards you because of the disease?

R: No.

I: They behave in the same manner as they did earlier?

R: Yes, everyone is the same. Even at home.

I: Why did you not tell your neighbours about the HIV diagnosis? Even when your wife was diagnosed with it?

R: I told them when my wife was diagnosed with the disease.

I: What did you tell them?

R: That she had the disease. I told my aunt who runs a school too. My sisters in the village know too.

I tried to arrange for money for her treatment, it was a Sunday. By the time I arranged for the money, it was Monday. She died that night. She was anaemic too. She need blood transfusion too. I thought I would get her all the treatment that she needed but she died that night at 4:30 am.

The doctors told me very late to get some tests done. I had a fight with them and asked them to refer her to a better hospital if they could not make her better. That is when we got to know that she had this disease. From the blood tests.

I: Before that you were not told?

R: No one told me anything before that. I spent Rs. 14, 000 getting treatment from private doctors. I was too tired by the end. If only someone had told me at the very beginning… She would not have been so sick. She died because I did not have enough money at that time to rush her to the hospital that very day.

*(long pause)*

I: When you told your aunt and other people in the village about the disease, did they behave differently with you? Any sort of discrimination?

R: No, not at all.

I: That’s a nice thing.

R: Yes, yes. We all eat and live together. There is no tension. The way you are sitting down with me, they also sit with me in the same manner. There is no separation or discrimination.

I: You said you were not able to work for three months in the middle. How did you manage your expenses then?

R: I told my aunt that I would come and work for the school later, once I was better.

I: So your aunt helped you financially?

R: Yes, she did. A friend of mine has taken my son outside. He’s 10 years old. These two help me with my expenses, even today.

I: Why did you let your son go? Isn’t he too young?

R: He wasn’t studying here.

I: So what is he doing outside?

R: My friend has a clothes shop in Kolkata. My son helps him in the shop. I am a poor man, I have no means of support, what else can I do?

I: So he sends you money?

R: Yes, he sends Rs. 2000 – 3000. I have no interest in that money though. It’s my son’s money. He should eat well and live well. He is only a child. But then this is enough to take care of the needs of my daughters. I don’t have any land for farming.

I: How about your daughters? What have you thought of for your eldest daughter?

R: Once I go back after I get better, I want to bring her here to Patna for treatment.

I: Is she married?

R: No. She is very young, only 16 years old. I want to get her checked here. I want her to be healthy. I will do my best to send people who have this disease in my village for treatment here. No one in my area has seen or heard of this place before. They only know about the local doctors. Many people have died of this disease in the village. Of Kala Azar. And no one in the local hospitals tells us about Kala Azar. Not even in Patahi.

I: Why do you think so?

R: It is a small hospital. Doctors are not present there. If they are there in the morning, they won’t come in the evening. (Long pause) When I got checked in Patahi they said I had nothing, then I went to Sheohar where I was diagnosed with Kala Azar.

I: How many people have died of Kala Azar in your village?

R: A lot of people have died of Kala Azar. My dad, my grandmother, my *bhau*. I performed her last rites 10 days before coming here. She too had fever which was not going away. There is nobody at home. Only my 16 year old daughter. The 2 daughters, they cook food (themselves) and eat. The younger one didn’t agree to stay back home and came here with me since she doesn’t have her mother anymore. I keep her outside.

I: Where do you keep her?

R: [19:40-19:42] There only.

I: Why do you keep her outside?

R: No no. I keep her inside only. I have kept my daughter (with me). There (they) hit her.

I: Where was she before? In the village?

R: Yes.

I: Now where have you brought her?

R: One man was coming here so she came along with him.

I: To the hospital?

R: Yes.

I: There she was beaten? I didn’t understand this part.

R: The men there, after her mother died and I wasn’t there, used to beat her up due to which she wanted to come to me. [20:08-20:15]. so a man brought her here.

I: So now in the hospital your daughter, your mom and you… 3 people are living?

R: Yes. My mother is here since the beginning. When she came here to look at how I was doing, my wife died.

I: Have you ever felt that there is no point in living anymore, have you ever had any suicidal thoughts or tried to commit suicide?

R: No. There is nothing like this.

I: Was there any loss of weight?

R: No. My weight has remained 45kg throughout.

I: Right now?

R: They do not let me look at the scale of the weighing machine. I have gained some weight.

I: When you look at yourself in the mirror, do you feel that you have changed in any way?

R: Earlier I wasn’t like this, I was darker.

I: So you are feeling better now?

R: I am feeling much better. I am feeling strong too.

I: How were you before the illness?

R: Before the illness I was darker. I used to get fever any time. I used to pop a pill the fever would subside and I would sweat and then I would restart driving.

I: For how many days did you do this?

R: Around a year.

I: So for one year you battled fever while driving.

R: Yes. That time even food was a problem. I had to ask others.

I: But you must be earning some money?

R: No, I wasn’t driving that time.

I: But you said just now that you had been driving.

R: No, I wasn’t driving during the time I had fever. When my wife died, and the tension of taking care of my kids, [22:41]

I: What about your surrounding environment?

R: It’s alright.

I: In Patahi?

R: There is no problem.

I: Your relatives, neighbours, how are they (treating you)?

R: They are fine. We all stay together, sit together, there is no problem.

I: (pause) anything you would like to say about the treatment here?

R: This is not a hospital, this is a temple and the doctors here are Gods. They save people like us. What else can I say? First time in Patna…. I have come here 3-4 time with family... But I haven’t found a hospital like this anywhere else.

I: What would you like to do about your family and yourself (asking about future plans)

R: About my family, I would like to get all my family members tested here once.

I: Anything else you would like to do?

R: Any person with illness, I would recommend him/her to come here. If nobody helps, then I will myself bring the person here and save his/her life.

I: What all do you think is necessary to live a good life?

R: I don’t understand.

I: Like every person thinks about how their life is and what all is required (explains question)

R: In life. I want that my kids stay together, they live decently and die when the time is right (old age), live well, speak and conduct themselves well in society, have no tension, don’t worry about others and be focussed on their own lives. I don’t go to other people unnecessarily. Only if they invite me, like for any function, I go. Otherwise I stay to myself. I do my job as a driver. Regarding the hospital, I am very happy. This not a hospital, it is a temple.

I: This is a great thing and a reward for the people working here. Now that you will go back to yur village, what have you planned? Will you be able to restart your job?

R: Not straight away. I will get myself checked 1-2 times again and when I receive confirmation from you (doctors) that I can work ….

I: How does your body feel?

R: It feels very good. Much better compared to the past (before treatment). Only when I eat and drink something will I become healthy. I am feeling better than what I felt 4 years ago. As if I have gotten a new life.

I: So your illness has been there for the last 4 years?

R: Yes. 4 years ago, I had weakness, which has gone now.

I: So for 4 years you suffered from this illness?

R: Yes. In 4 years what other doctors couldn’t do, the people here in this temple (RMRI) did in 1 month 15 days.

I: How many years ago did your wife die?

R: Not even a year. Around 8 months ago.

I: For how long had she been suffering?

R: She got a wound in here (shows site) which didn’t heal. So we went to Arun Tiwari, after which the hospitals in Patna were closed. We again took a bus and came. They told us she is eating soil [27:36-27:39] and that she needed a blood transfusion. The problem was that after we got the transfusion done and we were going to bring her back, she died.

I: Any kind of worry regarding the work you were planning to do?

R: No no. There were worries earlier, but after my wife died I forgot all my worries and concentrated on taking care of my children. I have since focussed on my own health and taking care of my children. I am trying somehow to earn enough to take care of my children till they are old enough and then get them married after which they will go to their homes ( refers to daughters). I have one son, if he wants to he can take care of me otherwise I will just live alone.

I: Any financial assistance you have received from the government in all the 4 years of your illness?

R: No. Nothing.

I: Have you heard anything regarding this?

R: No.

I: Any changes you would like in the government hospitals you visited? Like you said in the beginning you had visited private hospitals. Why didn’t you visit govt. hospitals straightaway?

R: I *had* gone to one govt, hospital in the beginning, after getting a Rs. 2/- ticket. That time I was coughing a lot. So they gave me 2 tablets.

I: How many years ago was this?

R: This is 4 years ago.

I: 4 years ago you had cough (as the first symptom)?

R: Yes. So they gave me tablets. It (cough) didn’t subside. It was winter. I then went to a pvt. Doctor. He gave me medicines and injections and I got relief. My cough used to worsen in the winters and get better in the summer. So I thought it wasn’t anything much. I got to know about the disease from here. In Sheohar.

I: When you went to the govt. hospital, how was your experience?

R: There they told me I had HIV.

I: Where? In Sheohar?

R: Yes. After I was told this, I ran there daily asking them about treatment and what was to be done and they referred me to Motihari where I got blood tests done and I was diagnosed with Kala Azar. They asked me if I could go to Patna and I said that I would go there immediately as I wanted to get better, so they told me to reach Patna by the next day and I said that I would come to Patna and knock on the doors at 3AM. I came to Patna on time and waited. I had decided that I won’t leave the hospital till I am cured. Only when I am cured and the doctors tell me that I can leave, will I leave the hospital. I would stay here (till I was cured) even if that meant dying here. Amit Sir sent me. Medicines and injections were started soon. After 10 days my appetite started increasing, other symptoms like the fever also started subsiding. Earlier I would get fever and chills a lot but the doctors gave me medication and treated me very well.

I: So total in 4 years including the charges of the pvt. Hospitals, what was your total medical expenditure?

R: Around Rs. 60,000-70,000/- . There (at the pvt. Setup) the doctor’s fees itself was Rs. 300-400.

I: From where did you arrange for these funds?

R: I took loans.

I: From whom?

R: From relatives and friends [32:06-32:10]

I: How much time will you take to pay these loans off?

R: I won’t be able to pay them back. There is 5% interest too.

I: (explains the concept of interest) so you have taken a loan *with interest*?

R: Yes, I have paid some of it back but some is still left.

I: How much time do you think it will take to pay it back?

R: Let’s see. My son sends around Rs. 2000-3000. I earn around Rs. 3000-4000. I have to pay around 5000 (monthly?). If I am unable to pay, then they will start taking my possessions. I will apologize saying that I cannot pay all of the loans back.

(Interviewers discus about total loan amount)

I: All the 70,000 was taken on loan?

R: Yes. I don’t have any land.

I: But you said your aunt helps you out?

R: Yes. She gave me Rs. 2000 to travel for treatment when I was sick.

I: The money you took on loan, it was only for medical expenses?

R: Yes.

I: What about household expenses?

R: That too I managed by asking around people for money. (pause) I had to sell off land which was in my father’s name to perform the last rites of my wife.

I: So you had to sell off land.

R: Yes. I have a loans of Rs. 150,000 to pay right now.

I: But you said 70,000.

R: That is only the medical expenditures.

I: How do you plan to pay it back?

R: I will do whatever I can from my earnings. If I cannot, then my son is there, he will send 2000-3000, slowly he will pay it back.

I: Any worries you have regarding all this?

R: No.

I: Any other worries you have regarding the future?

R: No, nothing if that sort. Regarding the loans, I have taking money from people who are willing to take back the money without interest too. They understand that I am a diseased old person, with 3 daughters.

I: Any worries you have regarding getting them married?

R: The society, friends are there to help me. What do I worry about my son. He can go and fend for himself even without me. It’s just the 3 daughters.

I: Any plans to build a house or anything? How is your house?

R: No I have a mud and hay (kuchha house) house. How can I build a house without money? I will feed myself first or build the house? I live in 1 room and my brother in the other. They say it is better if the family lives together.

I: Anything else you want to say?

R: No. You (doctors) have saved my life which is more than enough.

I: Thank you very much.
