# Supplementary material for: Quality of life perceptions amongst patients co-infected with Visceral Leishmaniasis and HIV: A qualitative study from Bihar, India
Source: PLoS One. 2020 Feb 10;15(2):e0227911. doi: 10.1371/journal.pone.0227911 (PMC7010301; doi:10.1371/journal.pone.0227911)
Supplement: S3 File — (ZIP) [file pone.0227911.s003.zip › Transcripts/Patient 3 Male Age 39.docx]

**Patient 3, Male, Age 39**

R- I boarded the vehicle at 6:00 am to come here.

I- You took the bus? You did not take the train?

R-No…bus is more comfortable. My house is there..buses keep crossing across it..

I-Your house is on the main road?

R-Its on the main road…[redacted]

I-That’s good.

R-That’s why I board from there at 6:00 am I-By the way, what kind of work do you do?

R-I did the work of loading as well as drove vehicles.

I-Loading of what?

R-Loading of powder.

1. You mean you lifted it up and kept?

R-Yes ma’am..it came from foreign to Chennai.

The plastic (inaudible) which is made..water tanks..their powder ..chemical…I loaded all that…

I-Ok..so this work you did in [redacted] or somewhere else?

R- No ..in [redacted]

I-Ok..you worked in [redacted] So , how long have you worked in [redacted]?

R-In [redacted]I worked….(thinks) I worked for three years..after that

went to [redacted]

I-Ok..you lived at [redacted]after [redacted].

R-I lived in Ludhiana…mom and dad reside there. When Dad married again…he did not look after us..we were very young that time. When we started working..we worked at some place or the other and fed ourselves…2 ..brother and sister

I-(trying to clarify) you have two brothers and sisters?

R- Only 1 brother and sister

I-Ok..so two siblings

R-yes..mummy..mummy was no more..

I-Your father remarried?

R-yes..he remarried…

I-And they lived in [redacted]?

R-Yes..they lived in [redacted].

I-How young were you when he married?

R-I was around (pauses) 13-14 years…sister was 10..8(thinks and tries to confirm)…8 years …and paternal grandmother was there..

I-so you all lived at [redacted]and father at [redacted]?

R-No ..we lived there only…

I-At [redacted]itself?

R-Yes ..at [redacted]..in separate house..

I-Ok…at one place..your father and new mom lived..and at other..your grandmother with you and your sister.

R-Nods..(in yes)

I-so…your expenses…of food and others

R-grandmother was there ..she worked at “kothi”…200..1000..500..as it came..we ate

I-Father..did he help?

R-No..nothing…in the sister’s marriage also..he didn’t help

I-Oh…then all your works..who did? Sister’s marriage or..(interrupted by respondent)

R-I did them myself.

I-You yourself did all that?

R-I have done myself..some money was saved from grandmother’s earning…I worked when I was young…all that money

I-From when are you working?

R-Work…I am doing from 14 years of age..

I-Since then..you have been doing work of loading..unloading?

R-No..lived in [redacted]

I-Tell me from beginning…from 14 years till now..what and how you have done?

R-Initially..lived in kothi..there I lived 4-5 years..started working…they gave 1000-500 per month..there I worked..

I-Where your grandmother was working?

R-No..

Yes…the place where my grandmother worked..at a little distance from it ..i worked in [redacted]..brought vegetables..thats it..and then when I started doing work of loading I went to Chennai..with person from village..

I-Someone took you there?

R-Yes…villager took me..when I came to village..he asked how much do you earn monthly? I said 1000-2000 per month..then he said..come..you have to do work of loading there..earning is good

..so I went there…lived for 3 years..

I-then…how many years?…started working from 14 years of age in [redacted]..after how many years you went to [redacted]?

R- after 12-13 years I went

I-Then..you..(interrupted)

R-It must have been 10 years..

I-Went after 10 years? You must have been 24 years of age by then?

R-Yes..only then I went..I lived for 2-3 years there..

I-At [redacted]?

R-At [redacted] (agrees)

I-Did you drive truck there?

R-No..drove but not here..

I-Then where?

R-At [redacted]..

I-Ok..you drove truck in [redacted]..

R-Yes..

I-So you both worked together? [redacted]and truck?

R-I left [redacted]after working for 5 years..then I went on to vehicles

I-Ok..

R-Then..vehicles(fades)

I-Till where did you use to drive the truck ?

R-It went to [redacted].. [redacted]

I-Ok..so 5 years you worked in [redacted]..then

R-5 years with truck

I-5 years you worked with truck

R-Then I came back to village. Came to village..then a villager took me ..

I-Ok..so u came to [redacted]?

R-Yes

1. From [redacted] a villager took you to [redacted]

R-Yes..took me to [redacted] where I lived for 3 years..

I-Who all are there in your house?

R-In my house..my grandmother is there..children..my wife

R-One is of 2 years

1. 2 children are there?

R- 2 sons..and 1 girl..girl is the eldest

I-Your grandmother returned with you to village from [redacted]? R-yes..came to village

I-The same place where u cited the house at main road

R-Yes..came to village

I-Ok..now..now what work are you doing?

R-initially did the work of loading

R- I came from there only after doing work of loading

I-How much time has elapsed of your staying at [redacted]?

R-7…7 months have passed

I-Oh..only 7 months have passed?

R- yes..from [redacted]..from the time I have become sick..i returned

I-How was ur last year?

R-Took medicines..used to get relieved..medical drugs I used to take…I used to be back to work when the fever settled down..In [redacted]

I-What do u mean by medical’s drugs?

R-I took drugs for fever…and used to recover

1. You mean from the shop?

R-Yes..from the shop

I-Did you take any doctor’s consultation?

R-I did..but they told me other things..that you have less haemoglobin and (mumbles)

I-Was it government or private setup?

R-I consulted in private set up

I-How many months in [redacted]..(pauses)7 months..when did your problem start?

R-It started in 1 month

I-Did it start immediately after you reached?

R-No..after working 2-3 months

1. You worked for 3 months

R-Yes…worked for 3 years

I-Then it started?

R-Yes…

1. You lived continuously for 3 years in [redacted] (interrupted)
2. R-Not continuously..used to work for 1 year and then returned I-U returned here? And then went back? R-yes

I-Well..the problem that u told…haemoglobin deficiency..after how many days of working in [redacted]did it start?

R-2 years

I-Were u completely well for 2 years?

R-Yes..i was alright. After 2 years..i started feeling a bit..problematic

I-Started feeling problematic

R- yes..felt feverish..took the drugs and got relieved for 1-2 months..again had fever

I-So how did you know about your disease?

R-from there I went to [redacted]..govt. set up..from where they referred and told me that there is no treatment here..u can go to [redacted]

I-was any kind of test done there?

R-yes..

I-which test?

R-blood test was done

i-what else?

r-nothing else..did a blood test and referred me

i-apart from blood..like urine test..sputum test

r-that was done in private set up

i-where in pvt.? [redacted]..or(interrupted)

r-no..at [redacted]

i-it was done at [redacted]..so along with ur blood test..urine test was also done

r-urine test and blood from bone was also tested

i-ok..from bone was also tested..

r-yes..in pvt set up

i-all in pvt..what was done in govt set up?

r-only blood test

i-which blood test?

r-kala-azar

i-was it blood test or took from bone?

r-no..from bone they took in pvt..in govt , blood test for kala-azar

i-ok..after that where and who told u about this disease?

r-nobody told me

i-nobody told u?

r-nobody

1. what were u told?

r-yes..only that there is no treatment here..u will have to go to [redacted] govt hospital- people of [redacted] sent me

i-so..at govt when they did test for kala-azar(fades)..from there only r-yes..from there I came..the same day i-ok..the same day

r-by ambulance of 2000 rupee fair

i-oh..by ambulance u came..

r-yes

i-after reaching here..who told you and when?

Did you know about your disease before coming to [redacted]?

r-no..before coming to [redacted] I only knew about Kala Azar..

i-only Kala Azar I see

r- I did not know about this other disease

i-when you came here… what happened then?

r-here they took sample from bone..and blood test ..x-ray, ultrasound was done. I was admitted..2 and half months and then from there I came to know about this disease [HIV].

i-who told u?

r-[redacted] sir told me

i-oh..how did u feel?

r-ji..drugs(interrupted by interviewer)

i-having listened that, how did you feel? When [redacted] sir told..

r-when he told..i started thinking what disease have I got in life..then I started thinking how will I earn and feed my 3 children

i-did anything else come up in your mind?did you feel anything else?

r-after that nothing pleased me…I was afraid..i felt that I should eat something and die

i-but why did you feel so?

r-because..this disease they told will require treatment the whole life

r-as long as you take medicine you will be fine..when you don’t take medicine…you will not be fine

1. how did your thoughts change over time, if at all?

r-i got relieved here then..i got over it. Doctor helped me..and madam ji helped..they told don’t think otherwise..u will be fine..slowly

i-so you shared your feelings with them?

r-yes..they said..keep taking the drugs..nothing will happen..slowly I consoled myself that they are right

i-did you tell your wife about these diseases?

r-i got my wife tested too..she also has it

i-oh..she also has it?

r-yes…but my child doesn’t have. child was also tested…child doesn’t have but wife has

i-when you came to know that you have the disease, did you tell your wife?

r-yes,there itself I got her tested

i-ok..she was with you that time?

r-yes. She was with me.here the younger child’s test was also done.he was of 2 years. His test was done,it was negative. The older one was tested there.

i-oh..what about ur grandmother? r-grandmother didn’t live with us for this long i-but is she with u at present?

r-yes,at present she is. She goes after a month or two

i-where?

r-at her son’s place , she goes

i-but since childhood, ur grandmother has kept u with herself more often, na?

r-yeah…she did since childhood but now she goes home(interrupted) i-means, u have not told her about this? r-no, we have not told her.

i-she doesn’t know that u are ill or something like this?

r-(promptly) no..told about kala-azar.

i-about kala-azar,not about this?

r-no..not about this..

i-children….obviously not..what about children? What have you told

your children?

r-with children, we don’t eat..we don’t kiss them on hands and face(mumbles,inaudible)

i-ok..from the time you and your wife have been told about this..how has your life changed? Did something happen that made you think that now(pauses)

r-life..to me not like this..i still think that how my 2-3 children will be brought up…even now I am thinking..that both of us are ill..with this disease how will our children be brought up?

i-your wife is also being treated?

r-yes, her treatment is also going on

i-so,what do you think about a good life..or happy life?

r-(promptly) for a good life, I am thinking that children(interrupted)

i-no..according to you ,what is a good life?

r-when my children grow up and start earning, then my tension will come to an end..when they start taking care of their own houses…I am thinking only this much..till the time they are young, there is tension.

i-do you think about some other thingwhich you feel is necessary about a good life? Like environment or some other luxuries?

r-luxury..look..now I am not able to understand anything.what will happen?after getting the disease, I am not thinking this in my mind

i-oh..but for being happy, are other things required in life?

r-mumbles

i-one…as you told..is important for you is that when your children are able to earn, your responsibility will be fulfilled..apart from it, if

you sit and think about a good life then what do you think? Few people think about house(interrupted)

r-I think that if I were fine and didn’t have disease..then I would have bought land and constructed house and earn and save money..this is what I think..if I were fine(upset and thinks)

i-you think that if you had money..your life would be better..

r-would be better..my children would be brought up(mumbles)

i-apart from it,anything else? Like good family life..clean environment at your residing place..do you think something like this?

r-inaudible

i-or.. people residing in your neighbourhood..your relationship with them should stay good?(pauses) do you think about this?

r-yes..i think that if relations are good..only then people will ask about well being

i-amomg relatives, you have your sister,u talk with her?

r-yes

i-do you visit each other’s house?

r-yes,my sister comes..grandma’s sister..she also comes.

i-ok..something about environment? Cleanliness? Do you have your own house?

r-yes.own house is there..my father..that mummy has four sons..they have built their house.

i-where?

r-here itself..and my house is a hut there..there we live

i-ok..do you feel that if the environment was better, then(pauses)

r-yes..i feel that..if I were better then I too could have built

i-oh..so you feel that if you were fine..you could have done all those things?

r-i could have done

i-so, what do you think about your life at present?

r-about life..i think that I am taking medicines..till the time I am alive..i will take medicines..thats what I think.. about work I think..that if I work I will earn money(interrupted)

i-well..life and death are the ultimate things..but apart from it..if you look at your life, how it changed after the disease, what do you have to say?

r-(mumbles)yes..my life changed..i am fine

i-so..at present do you feel fine?

r-yes..at present I feel fine

i-no, I mean, from the time you came to know about this disease(interrupted)

r-from that time..i think that how did I get this disease?

i-oh..you think how you got this disease..

r-yes..i didn’t know about this

i-you didn’t know

r-i didn’t know…when the doctor told then I knew

i-(pauses)ok..so now..the place you live..you told your house is kuchha or a hut..what do you think about this?

r-about this I think that I will earn and make 2 rooms..when I earn(thinks..) this is what I think..when I go to earn..both of us will go out..will work(interrupted)

i-how has this disease affected your job?

r-job has been affected much. there is no money to feed the children

i-are you working at present?

r-no..just sitting at home

i-only because of this disease?

r-only because of this disease..i am sitting(upset)

i-You will get to work there as well.. r-no..i am not getting any work..i get agricultural works i-apart from agriculture works? You can do agriculture works?

r-yes..i can do agriculture works..but they have asked me not to work for 6-7 months..doctor has said..so I am unable to do

i-how do you feel yourself?

r-i..(interrupted)

i-Will you be able to do if you are given a work?

r-yes, I will be able to do the work.

i-so you feel..you can do..

r-yes

i-but..but you are saying that you have been taking medicinesfor 7 months..

r-no

i-so for how many days are you taking medicines?

r-3 months

i-the treatment that you are receiving here(pause)..as you have been coming for 3 months for the treatment..

r-yes

i-what do you think about the treatment?uh..the staffs who are giving you medicines..the doctors who are meeting you..writing about you..how do you feel about their behaviour?

r-its ok..good..there is no problem

i-is the behaviour good?

r-good behaviour..they talk nicely

i-do you have any complaint against them?

r-no complaint

i-ok..if you are told a thing..that the way they behave with you..give you medicines..one thing that you wish to change..or you want that if something was this way…it would be better..do you think something like this?

r-i think

i-what..what do you want? What could have been better?

r-i think that I get no work in village..i am thinking about job

..both of us will go from here

i-good..very good

r-(inaudible..)I can do some easy works..this is what I am thinking

i-the medicines that you get ..or when you come to [redacted].. from the hospital….from where you take medicines..there what do you think

could be better?

r-i wish my treatment goes well..this is what I m thinking..that I become fine here..

i-you think that u become well…but this is about medicines..the way you get medicines…how many times you..i mean..as many times you come(pause)..uh..once you were discharged after 2 and a half months,na?

r-yes

i-after that how do they call you?

r-uh..they call after a week

i-so..are you satisfied with it?

r-yes..whenever they call..on whichever date they call..i come on that date..they called on 16^th^..i came on 16^th^

i-do you want any improvement in it?the way they are giving there

r-yes

i-do you want there should be any change in it?

r-no

i-are you satisfied?

r-i am satisfied

i-what do you think further in your life?what you plan to do in life? Like your disease..your wife..your 3 children..so now what do you

think? How you imagine your life in future?..like I have to do this thing

r-ji

i-so what is that thing you want to do?

r-uh..anything like this..about work ..i will work and make something in future..so(inaudible) I am thinking

i-ok

r-both of us want that we stay fine..do a nice job..make both (corrects)all three children independent..even if some money is there in their account..they will marry

i-oh..so you want to earn and deposit money in their account..(interrupted)

r-yes..in their account..i want to deposit

i-and if(pause)..due to this disease..do you think you will be able to do?how?

r-now..i will be able to do(mumbles)..i want to..i want to work from the core of my heart(interrupted)

i-before disease..what did you think?

r-before the disease also I worked….did not want to work on the

days when I had fever

i-so before that..when you thought what to do in life..before the disease what you thought?

r-initially in life I thought I will buy land

i-ok

r-will do farming..work for 2-4 years..and then work at house

i-means..after working outside..you wanted to come back home..after buying some land

r-after buying land..i thought I will build a house..will live in the house..do any work

i-means..you wanted to start your own work?

r-yes

i-did you change your thought due to the disease now?

r-it has changed..now I wonder.. how will I earn?(upset)

i-do you feel that you will not be able to do whatever you thought?

r-i will not be able to do

what I initially thought will not be possible. Shall I feed them or save money..clothes(interrupted)

i-but you were saying that you can work..

r-i can work(emphasizing)but that enthusiasm…won’t be able to do that much as I did earlier..like I did loading..earned 10000-20000

a month..15000 .how can that happen now? Now it can’t happen..i can do a work of 4000-5000 monthly

i-ok

r-(mumbles)

i-so…are you feeling this?

r-yes..the heavy work earlier for which I earned 12000-13000 monthly..weekly..how can I earn that way?not possible

i-what are you thinking now?

r-both of us are thinking to go out ..take a room somewhere and do an easier work..this is what I am thinking..when some money is earned..i will open their accounts and deposit

i-will the children reside with you?

r-children will reside with us

r-you will take them too?

r-yes

i-do you want to say something else?

r-no..thats it..

i-ok..thank you for taking out time to talk to us.
