# Supplementary material for: Quality of life perceptions amongst patients co-infected with Visceral Leishmaniasis and HIV: A qualitative study from Bihar, India
Source: PLoS One. 2020 Feb 10;15(2):e0227911. doi: 10.1371/journal.pone.0227911 (PMC7010301; doi:10.1371/journal.pone.0227911)
Supplement: S3 File — (ZIP) [file pone.0227911.s003.zip › Transcripts/Patient 5 Male Age 30.docx]

**Patient 5 Age 30 Male**

*Note: patient had an intellectual disability which made it very difficult to comprehend questions and communicate. Patient’s attendant assisted in translation and communicated on his behalf.*

I1: Can you tell me about yourself ?

R: Yes

I2: tell something …. Where do you come from ?

R: I came from [redacted]

I: when did you come first to [redacted] where your treatment is undergoing?

R: I was having Kala-azar and HIV

I : How did you hear about this (repeat)

I2: How do you know about this?

R: aree I know about this by someone who told me and given some medication when I went to [redacted]. In [redacted] I was told that you will be treated in Patna

R2: some investigations were done in [redacted]

I2: who told you about this in [redacted]…. Doctor or someone else

R2: yes doctor told about this , some investigation were done in [redacted] and after getting report he was told that he is having Kala-azar and HIV . From there he was referred to [redacted]. there was one person there itself as they were unaware about that so he admitted him

R: he belongs to [redacted]

I2: Ok.. what happened to you that make you to went [redacted]

R: there was a lump in abdomen on one side after eating food

R2: when he was eating food he feel lump in abdomen.

R: when I ate food I was having breathlessness and pain

I2: any other problem or you was having only this problem

R: No

I2: how many days you was having such symptoms

R: About 1 month

(I clarify)

I: ok after having such symptoms for 1 month then you came. Where did you went first?

R2: at first here and there, at first he went to [redacted] and one or two time came to [redacted] but investigations were not done , only medication were given. When the symptoms become severe like he was very breathless and lump was there then we came to [redacted]

I2: was he completely alright before one month ?

R2: yes

I2: what happened after that

R2: after that some investigations were done ,HIV and Kala-azar was diagnosed in [redacted] and from there he was referred here

I2: In [redacted] there was govt. hospital

R2: ye, govt. hospital , it’s RC….. means Sadar hospital

I2: what happened in [redacted]

R2: treatment was started and then he get well . he was also treated for kala-azar here

I: How many days he was treated in Patna

R2: 1 month

I: before going to [redacted] did you went to pvt. Hospital ?

R2: if he was having pain sometimes then he went to pvt hospital and take medication

I: For this why did you went to pvt first ?

R2: he was having mild pain so we were thinking that he may having some gas problem , we take medicine from pvt. and get relieve

I2: pvt means some doctors or medical store

R2: yaa, from medical store at marketplace. After seeking doctors he take medication, he was having lump so people think that it is due to gas and get relieved on medication but it became severe so he was having difficulty to breath then we went to [redacted]. here test was done and HIV Kala-azar was diagnosed.

I: you know about both HIV Kala-azar simultaneously

R2: Yes

I2: You went to [redacted] by your own wish or someone told you

R2: own wish , [redacted] is also local but [redacted] is near, for me [redacted] is near but for them [redacted] is near that’s why he went to [redacted] where he was diagnosed after report

I2: how many days before he was completely well from now?

R2: one and half month from now . in [redacted] we spent 5-7 days as report was delayed. And after getting report we came here.

I2: how many members is in your family ?

R2: in family he is having mother, father and sisters. All sisters are married but one of them is staying there as his mother and father is old so she prepare food for them. He was married but his wife left him because he is having tremor. So younger sister is there who prepare food for them

I2: do his sister’s husband also resides with them

R2: No, he lives outside but when he comes then he also resides with them.

I2: any child

R2: No

I2: what did he work ?

R2: he didn’t work . His hand and feet are having tremor , u can see when he walks

I2: it happen in last 2-3 months or before ?

R2: It was present since childhood but this disease occurred in last 2-3 months. Not 2-3 months but may be 5-6 months . he was not having problem but when lump was developed then he seek for treatment. After that when he didn’t get well we went to [redacted] hospital where we know about this disease.

I2: what do his parent do for their living ?

R2: farmer

I2: do they manage their house with that

R2: yes ,yes

I2: Do they have their own house?

R2: they don’t have their own house but it is on the government plot

I2:how has this past month been for you? How do you feel after you was having this disease?

R: it feels like…whenever I eat food..food..i developed some lump so I came [redacted]. I told my father that I am having lump in abdomen

R2: he was also having weakness like symptoms, at that time he told that when I stand I feel like my eyes are getting closed means he was having weakness and having lump so we seek for treatment. This weakness was present for around last 1 month

I2: Fever?

R2:yes, when he was having fever he didn’t tell anyone. He roams here and there in days but in night he was having fever . when it became severe then he told this to his guardian

I2:why didn’t you told this to your parent?

R: I told them

I2: where do his wife lives?

R2: she left her and went to her parent’s house.

I2: any child?

R2: No….the neighbor told her that when he don’t earn then how do he maintain family . he don’t earn so upto how many days they remain dependent on his parent? Other people told this so she went to her parent’s house and she married to someone else.

I: it happened before the occurrence of this diseae?

R2: yes, it happened before that…Perhaps, I can’t say that this disease happens before she came or after she left him

I: ok, you can’t say

R2:yes

I:when you found out that you was having HIV kala-azar , how was you inform about that? What was told you about this disease?

R2: I wasn’t available at that time but his father was available

I2: what do the doctor told you about this disease ?

R: doctor told me that I am having Kala-azar and HIV

I2: what do you understand about this disease? What happened in this disease, was this told about you?

R: No

I2: ok, when you came to Patna for treatment then is there something was told what happened in this disease , why it occurs?

R:yes, but I don’t remember

I2: what do you understand about this disease?

R: I don’t remember

I2: what do you feel psychologically about this disease?

R: Psychologically, I feel how this happened to me, feeling like insane.

I2: before this disease you don’t feel like that?

R: No

I2: what do you know about this disease?

R: I don’t know

R2: he is illiterate so he have to explain in Bhojpuri

I2: Do everyone in your family knows that you are having disease?

R: yes

I2: who inform them?

R: Doctor told this to my father

I2: and mother?

R: my father told her

I2: and sister knows about this?

R: yes

I2: do you noticed any change in their behavior towards you after this disease? (R2 explains)

R:No

I: and friends or neighbors, tell something about them (R2 explains)

R: No

I: Do anyone tells you anything in your village? Do people in your village knows about this?

R: No….In village only one person knows about this

I2: Who

R2: one who sprinkle insecticide for Kala-azar from Chakiya block, he also came with him and knows about this

I2: how are you feeling now?

R2: now he is fine

I2: what do you feel that has become better than before?

R: I feel better than before

I: what do you need for good quality of life? (R2 explains)

R: I don’t understand anything about this (R2 explains)

R2: Normal, what is present from before is what he need. He don’t need anything special.

I: what is needed for this normal? How is he spending his day?

R2: days…just he needs food and rest in house

I: what do he do after that?

R2: nothing else, just roaming around

I: roaming around…outside?

R2: no, only in village

I : with whom? Alone or with someone?

R2: sometimes alone or with cousins

R: I was having weakness so I don’t roam with anyone. I cannot walk.

R2: when he went from [redacted], he feels like weakness so he left to walk around.

I: how do your neighbor or friends behave with you so that you feel good?

I2: Do you like any work? (R2 explains) what do you like? Food or something…?

R: No I don’t like roaming around . I like food and earning money…

I2: which kind of work do you like?

R: I am not able to do work.

I2: which work do you like to do?

R: I like to do any work

I2: how do your neighbor or friends behave with you so that you feel good?

R: I don’t understand (I2 explains) …..I need food only

R2: actually the matter is… he is not able to do any work even a cow is there in house (interruptions)

I2: are you enjoying now?

R: yes

R2: what I was telling that there is a cow and even he can’t tie her, whenever he try to do so his hand start trembling. He don’t do any work.

I2: you don’t work because of this tremor? Do you feel bad about this?

R: I was having weakness because blood was drawn out from me.

I2: Do you feel if this doesn’t be there then you can do something better? (R2 explain)

R: yes

I2: which work

R: Any type of work

I2: what do you do whole day?

R: I give food to cow.

I2: Then?

R: Nothing (I2 explains)….after waking up I goes to toilet then eat and then give food to cow …that’s it

I2: and rest of the day?

R: nothing….earlier I can walk but from now it become difficult for me to walk some distance.

R2: if anyone having something to his hand and feet then he feels if that if it doesn’t occur then I can do something and do something further. The same thing apply to me also, I am a polio patient

I2:How do feel about your care and treatment in [redacted]?

R: I feel good.

I2: are you happy with it?

R: Yes

(I2 and R2 explains)

R: everything was good

I2: How are the staff?

R2: they all are good

I2: behavior? (R2 explains)

R: they speak rudely

I2: what , if anything , would you change anything about your care and treatment …(repeat) doctors or staff (R2 explain)

R: the care and treatment is good

I2: (from R2) can you say something?

R2: the treatment is all good

I2: what do you feel good about there?

R2: everything, compounder and nurses come from time to time and give medications. They always ask that is everything fine or not?

I2: and doctors come to see you? their behavior?

R: yes

I2: any changes in all that?(R2 explains)

R: no

R2: everything was good there

I2: what do you want to do in future? How will you spend your life?

R: life…I am having many things in my mind but I as I said I can’ do any work

I2: your parents are caring for you now but how will you live after the death of your parent?

R: That is the problem

I2: have you think anything about this? how will you eat?

R: No

R2: for him, he have sisters, brother in law and no one else

R: no one else other than them

I2: if all goes well and you don’t have any disease then what do you want to do in future?

R: In future many a thing….

I2: like what

R: Driving and working many a thing…

I2: marriage or not?

R: I want to marry but I can’t talk to parents.

I2: do you think this disease may affect your marriage in future?

R: no, no problem at all

I2: how do you think this disease affected your life?

R: I feel like my life become bad

I2: any other disease occurred to you in past?

R2: no, only minor cold and cough

I2: do you know anyone having same disease? In whole village ?

R: no

I2: what is present in his house?

R2: they are farmers , 2 rooms are pucca and rest are kuccha

I2: is the farming sufficient for their living?

R2: they gets grain from field and also earn by making kuccha house so may get Rs200-250 for that where he works and due to bad situations, his mother sweeps in school .he don’t have other sources only mother and father earnings. He is not able to work so can’t earn. Until when they are alive they earn and sustain. After they will die god knows what will happen in future.

I2: Do you think anything about your future?

R: I have think many a thing about future but I am not able to do any work.

I: you said you think many a thing about future? What are they?

R: I am thinking about driving a car…

I: any other thing

R: earning

I: from which thing would you like to earn?

R: by driving

I: You like driving a lot?

R: Yes.

I: Feels good?

R: Yes.

I2: do anyone in family is having such disease?

R: one of the sister is having Kala-azar

I2: what about your wife?

R: she was having TB and went for one month to take treatment for that. She died after that.

R2: perhaps, she died but I don’t know exactly but I think she was also having such disease and from her it occurred to him.

I2: do you also having TB?

R2: no, it is not proved yet

I2: Cough for more than 2 weeks?

R2: No

Thank you
