# Supplementary material for: Quality of life perceptions amongst patients co-infected with Visceral Leishmaniasis and HIV: A qualitative study from Bihar, India
Source: PLoS One. 2020 Feb 10;15(2):e0227911. doi: 10.1371/journal.pone.0227911 (PMC7010301; doi:10.1371/journal.pone.0227911)
Supplement: S3 File — (ZIP) [file pone.0227911.s003.zip › Transcripts/Patient 6 Male Age 59.docx]

**Transcript Patient 6 Age 59 Diagnosis HIV-VL**

Interviewer - Tell me about yourself ………Where do you live? About your family?

Responder - I am from [redacted] block, [redacted] and born and brought up there. My wife dead 6 months back.

Interviewer - What was the cause of death?

Responder - Doctor said, she had infection in throat, she use to have fever, initially then there was a difficulty in eating.

Interviewer - Any other symptom?

Responder - Yeah, during the last month, just before her death, she was having continuous loose stools, which was of very foul smell, her skin turned black and the doctors in the [redacted ] said that she won’t survive hence we brought he back home and she died within 3 days at home.

Interviewer - Did doctor said about her diagnosis? I mean what was the disease she was suffering from?

Responder - No…….. We only knew that there was some infection.

Interviewer - Who else are there in the family?

Responder - I have three sons but they work outside Bihar, I live with my one Daughter in Law my mother and my three grandchildren.

Interviewer - What do you do?

Responder - I am a farmer, I own some land also and I work on other’s farm also.

Interviewer - How was this past year been for you?

Responder - 6 months back everything was fine but suddenly while working in the farm, I developed high grade fever. After two days of the fever, I consulted a local RMP (Quack), he gave me few injections and tablets.

Interviewer - Did he prescribed any tests? (Pause)

Responder - No……………. he didn’t [*Smiling*]. You know these Jhola Chhap doctors. They don’t prescribe any tests.

Interviewer - Ohh……. Then for how long you were under him?

Responder - Thinking…………. I was there for 10 days.

Interviewer - Ohh……….. and how much money did you spend?

Responder - Hmm………. Approximately Rs.10000/- (Ten Thousand).

Interviewer - Then what happened?

Responder - As there was no relief, someone in my neighborhood told me to got to [redacted] to a private practitioner. I went to [redacted]. The doctor got X-ray and blood test done and conveyed to me that I have Kalazar and HIV both and referred me to [redacted].

Interviewer - Did the private doctor referred you to the Patna?

Responder - Yeah…………. He said that you will go broke seeing a private doctor…. Even I can’t correct your illness.

Interviewer - So……….. The doctor was very nice?

Responder - Yeah……… He didn’t waste my time……..

Interviewer - That’s really nice of him………….So how did you feel when you heard about your HIV-VL status?

Responder - I got very nervous listening this Kalazar.

Interviewer - And HIV?

Responder - I didn’t hear HIV……………. Knew nothing about it but heard that kalazar is a dreaded disease. The patient can die also due to Kalazar. I had one episode of Kalazar long time back.

Interviewer - When was it?

Responder - Doesn’t remember correctly but I had ………….. got cured.

Interviewer - Ohh……… So what happened next………. How did you told this to your family.

Responder - My daughter was with me. She knew……. And when reached home……… she told this to my sons………. They said……….They will provide money for treatment.

Interviewer - But the treatment is free of cost in [redacted]?

Responder - Yeah…….. But I was not knowing this at that point of time.

Interviewer - So how did you adjust to this news?

Responder - …..Thinking……. what to do ………… I thought everything will happen according to God’s will

Interviewer - Then what happened? Did you tell your disease status to Community members or neighbors also? Or Do you know any person suffering from the same situation in your neighborhood?

Responder - No……. I didn’t tell……

There is one person in my neighborhood who is also suffering from the same disease and taking treatment from Patna (RMRI).

Interviewer - Hmm……… Then………

Responder - But he told this to me when I was back to my village after spending 30 days in [redacted].

Interviewer - So you also told your status?

Responder - Yeah…….. he was sharing his experience of treatment at Patna.

Interviewer - Why didn’t you tell this to Community members?

Responder - Because I have heard that HIV is “छुआछूत की बीमारी है” (spread by touching & talking to person suffering from HIV). Hence having known the disease status of mine, everyone would have stopped talking to me.

Interviewer - So………… How the disease affected you?

Responder - I couldn’t work. Initially I used to carry 50 kg of raw rice on my head and used to walk for 1-2 km. Now I can’t even walk 200 meters with 10 kg on my head. My earning have stopped.

Interviewer - What do you about the factors of Good quality of life?

Responder - According to me it is अच्छा खाना पीना (Eating good food)

स्वस्थ रहना (Being healthy)

खुश रहना (Being happy)

Interviewer - Why are these factors important?

Responder - Thinking……..because if you eat good food, then your health will be good and you will be happy.

Interviewer - Ok….. So how do you feel about your life?

Responder - Now it is good……….. I am eating. I am able to eat now…….. and I am feeling well.

Interviewer - What about your work………… now?

Are you able to do your work which you used to do?

Responder - Not really……. Now I am taking rest at home…….. my sons will support me.

Interviewer - How do you feel about your care and treatment?

Responder - The treatment is very good. I am feeling good can walk without support.

Interviewer - What do you want to do further?

Responder - I want to become alright, like I was before……….

Interviewer - So before starting illness ……….. what were you planning to achieve?

Responder - I was planning to buy more lands……… to save more money for my kids………….. I have to get my children married………….

Interviewer - Do you think…… you will be able to do so now………..?

Responder - No……… I can’t do that now…….. not much energy is left in my body. Now I am dependent more on my sons for the income……..They have assumed me the economic support……
