# Supplementary material for: Quality of life perceptions amongst patients co-infected with Visceral Leishmaniasis and HIV: A qualitative study from Bihar, India
Source: PLoS One. 2020 Feb 10;15(2):e0227911. doi: 10.1371/journal.pone.0227911 (PMC7010301; doi:10.1371/journal.pone.0227911)
Supplement: S3 File — (ZIP) [file pone.0227911.s003.zip › Transcripts/Patient 7 Male Age 42.docx]

**Patient 7, Age 42, Male**

I:For how many years have you been living at [redacted]?

R: [redacted] is our district

I:(Interrupting) Were you born there ?

R:Yes,my house is in the village called [redacted].There I was born and brought up.

I:Have you been living in the same house from the beginning?

R:Yes

I:Didn’t you go anywhere else in between?

R:I did go outside. I:Where?

R: To [redacted]..had to do labour.

I:Who all are there in your house?

R:My father is there,mother and wife is there. I:Oh..

R:Umm..one son

I:One son..

When did you return from [redacted]? Did you go anywhere else other than [redacted]?

R:No..didn’t go to other place. I:Where in [redacted]? Which district? R:The district was [redacted].

I:Did you work in farms there also?

R:Yes

I:How much did you earn there? R:300

I:For one day? R:Yes..for one day.

I:But here,you must have been getting more than that.How much do you get

here?

R:There, many people are from my village. I:Oh..people go together.

R:Yes..we harvest wheat..in the sun(laughs)..together we felt good..we stay for

two months and return back to home. I:How much wages do they give you here? R:Where?

I:Here..in [redacted]?

R: [redacted]..uh..no..here rupees 200..280..we get 280.

I:But if you got 300 there..you must have been spending for staying there? In

Punjab?

R:No.

I:Is that free of cost?

R:Yes.They give you work..you stay at their house..flour..rice..you make and eat, then you work..ummm

I:(Interrupting)Oh..they give you ration?

R:No..we buy our own.

I:Then..what benefit did you have of going to [redacted]? R:Money is saved there.Even if we earn 200, it is saved.

I:Oh..so whatever small amount..rupees 100..you earn, that is saved. R:Its saved.

I:And here?

R:Here..they say..there is no vegetable..child is sick..someone has come..

I:Oh..so if you stay at home..your money is spent on illness..other people coming..on all this?

R:Yes.

I:But you must have been sending money to home anyways? R:I send..I send once..5000..2000

I:Oh..at once

R:At once..umm..whatever work they have, they can do..buy flour, rice and keep..

I:For how many years did you stay there?

R:We go in this season only..live for 5-6 months..then return back. I:For how many years,have you been doing this?

R:Not many days..umm..earlier used to do washing and all..

I:Washing of what? R: Of clothes.

I: Oh..of clothes. R:Yes..

I: Where did you do that then?

R: In village...people stopped calling me.

I:Yes..now-a-days..machine and all.

R:Yes.there is washing machine..they wash themselves..so we left the work..we returned and whatever we like plough the soil or do farming..that gives us livelihood.

I:What is your son doing at present? R:He too is working as a labourer..

I:Did you also take him with you to [redacted]? R:No..he is not old enough to earn.

I:No..Like it happens many a times that people take their children

also(interrupted) R:No..no..my son doesn’t go..

I:Oh..he doesn’t go..how many years old is he?

R:He is (thinks)..16 years now.

I:Tell me about this then..for how many days have you left working?

R:Work...used to do work at home and feed domestic animals there I fell ill..then work(trailing)

I:(Interrupting)Did you go to [redacted] last year..i mean 1 year back?

R:I came back in Dussehra..I do Navratri pooja from my

childhood..Goddess..During that I fell sick..got fever…when I got fever..in village I consulted doctor..injections were given..fever subsided and recurred..there is a small town..Bengali doctor is there..I went to him..he also give medicines..my fever subsided but I had swelling in this..i had no blood..i became pale..family members were worried..then there is a village called [redacted]..

I:Oh..Near [redacted]?

R:Yes..near my village. His name is Dr. [redacted]..I went to him..in night I vomited..I had gastric discomfort..so I vomited..I said that I will not be alive anymore..get me treated somewhere else..they took me..there blood was drawn and sent for test..and intravenous fluid was administered..they told 2 unit blood needs to be transfused..then I thought I will make blood by eating and not transfusion. The way it was before, I will make the blood by eating [well]. Blood made by eating and that by transfusion will be entirely different. Then they saw me there, drew the blood and sent for the test. Then they called me again..both of us went, then he told that I had Kala-azar and said –go to government hospital, gave me a written prescription and we went to government hospital. And also said that- if you take treatment here and there then whatever 1 or 2 katha land you have will be sold out and

still you will not be cured. I went to government hospital. I stayed for 4 days there. Medicines were not given.

I: Government…In [redacted]?

R:In [redacted]. Hmm..A person named [redacted]..whether a compounder or something else..he told that after doing this, I will take you to [redacted]. I told I had never been to [redacted]..and I am not able to go.He told come along with me and my sister’s husband was there.He came from [redacted] and I went by vehicle from [redacted]. When we came we stayed here in the temple..she rang me and got me admitted here.

I:When you fell ill, what happened first? Fever? R:I had fever.

I:After fever, any other problem?

R:When I had fever, blood was not forming inside my body.

I:How did you know this? Like you said you became pale, did somebody tell you that or you felt so?

R:No..I myself knew. Fever subsided on medicines and I had no balance to stand or walk.

I:You mean you had no strength..

R:Yes

I:To stand?

R:Yes

I:Ok..when you worked earlier,you needed somebody’s support to stand? I

mean, after the disease?

R:Yes..I holded onto something to stand. I:How long did it last?

R:Just few days. I mean, 15-20 days.

I:When you went to Dr. [redacted], was blood transfused? R:No..not blood..saline was infused.

I:Saline was infused.

R:Yes..1 bottle.

I: When you came to know, did they tell you anything else other than Kala- azar? Or only Kala-azar?

R:Only Kala-azar

I:Only Kala-azar..did not tell anything else? R:No

I:And only blood test was done?

R:Yes.

I:How did you feel on hearing that you have Kala-azar?

R:On hearing this I felt that I had Kala-azar. I went to government hospital. I:How did you feel when you heard this? Were you alone that time or was

somebody with you? R:No..my son was with me.

I:Did you feel something when you heard ?

R:I felt that I have no money..I will not be ble to survive.

I:Why? Did you hear this somewhere that in Kala-azar, you don’t survive?

R:My(trailing)

I:Oh, I see..you thought this.

R:Yes..then I called my sister’s husband and went to the hospital.

I:That you told in government.

R:Yes. When we went , we took the reports..tests were done there also..then my son was tested..my wife was also tested. What is that disease called(unable to recall)..all that was done.

I:Ok. Disease name..

R:I am not able to recall..(pauses and thinks)..the disease is not there

I:Your wife doesn’t have?

R:Yes

I:She too was tested?

R:Yes. Uhh(trailing and interrupted) I:Did you know about the disease? R:No.

I:What is the disease..how it happens..did you know anything? Did you hear about Kala-azar earlier? That what is Kala-azar?

R:When we were very young..many days ago..I heard about kala-azar. I:What did you hear about that?

R:That kala-azar has occurred.

I:No..what did you hear about kala-azar? That how is this disease? What is it?

R:It’s a bad disease..earlier injections were given..big injection.

I:And the other disease which you are unable to name , did you hear anything about it?

R:About it that people do not survive. I:And what else have you heard? R:thinks

I:Anything else that you heard..from friends, relatives when you sat together..if there was any discussion about that disease? Did you hear the name of the disease earlier?

R:Which disease(thinks) I:Something with H R:Yes..AIDS ..AIDS I:AIDS…One is HIV R:Yes..HIV..HIV

I:Did you hear about this disease earlier? Did you ever hear the name of HIV?

R:Not earlier..but heard now.

I:Now..you have been told. But when you didn’t know.then were you aware

about any such disease?

R:No..there was no such disease in my village.

I2:Did people living around you know about this? About this disease? R:About other people..

I:No..how would he know..

R:Do you know about other people around you having this disease?

I:In my village, a person younger than me lived in Pune. He died of the same disease in Pune.

R:Of kala-azar or HIV? I:No..of HIV.

R:Our cattle folds and houses are close to each other.

I:What is the cattle fold? R:Cattles are fed there.

I:You knew one person who had the disease..anyone else?

R:No..I don’t know about anybody else.

I:When you heard that you had HIV, was your son with you? R:Yes.

I:How did you feel that time?

R:I told that I will not survive anymore. You will have to take care of youselves on your own.

I:You thought you will not survive.Why did you think so? R:Thought so because there is no medicine.

I:Are you talking about HIV or Kala-azar? R:HIV..Got them tested.

I:Your wife and child? R:Yes..then I had more doubts. I:Oh..about what the disease is.. R:Yes.

I:When you brought your wife for test, what did you tell her?

R:The doctor told she doesn’t have.

I:Didn’t she ask that why am I being tested?

R:No..I told that get yourselves tested. The doctor has said so.

I:She got tested easily? Didn’t say anything to you?

R:No.

I:Before the disease what did you think you will do in life? Everyone thinks I

will do this(interrupted)

R:If I earn 10 rupees, a house can be built. There will be food for mother and father who have given birth. Otherwise they will abuse us after their death(laughs)

I:Before the disease did you think to do something else for your children, for your house?

R:To earn and feed.

I:Before the disease what other expectations you had from life?

R:I worked. Thought about food. To serve my old parents and I have a son

I:You thought something about your son?

R:About son I thought that I will earn and save money, he will then do as he likes.

I:After the disease do you think you will be able to do what you thought to? R:I will not be able to do as much as I thought.

I:Why do you think so?

R:I will do a bit of work..not go out in sun.

I:What do you plan to do now? Do you plan to go to Punjab?

R:No..I am not thinking of going to Punjab. Will arrange money and sit on a shop.

I:You mean some work that can be done while sitting..(interrupted) R:Yes..If I stay here farming can also be done..will keep cattle..sell milk. I:That means you are planning to change your work?

R:Yes.

I:What are the things you think are required for a good life?

(pause) like family life..surrounding environment..good health(interrupted) R:If health is good, only then everything is good.

I:Anything apart from this? R:What more

I:Why..apart from health ,are other things not required for a good life?

R:I pray for wealth to God. But I will only get what is written in my destiny. I:How will the God give wealth?

R:No..if I work hard only then can I have it.

I:Yes. What other things you pray for? Like every person thinks that if these things were there in my life, my life would have been be very good.

R:What shall I pray for..I pray that my child lives well, earns , feeds well. I:How is the environment around you?

R:Its good.

I:Are the people good? R:Hmm

I:Do you have any problem with them?

R:When you live together, problems definitely arise. I:What kind of problem?

R:This didn’t happen, that didn’t happen(laughs)

I:During the disease, was there any problem? R:No.

I:You told they didn’t know about the disease..there was no discussion ? R:No.You eat and stay in your house. If you don’t go there is no problem.

I:You told about someone who died of HIV..did he take treatment in your

knowledge?

R:He consulted in Muzaffarpur. I:Government?

R:Who knows where he consulted..but told there is no medicine. Many days ago it happened..his wife also died..his child also died.

I:Did they die infront of you or where he took them? R:No..at house.

I:What do you think about your life? How is your life at present? R:I took medicines and I am fine now.

I:How is your health? R:Fine.

I:What did you feel in your body during the disease?

R:I felt that I had less blood..in liver..I had gastric discomfort..vomiting

I:You had vomiting or felt like vomiting? R:No..I had vomiting.

I:For how many days did it last before you came to hospital?

R:One month later..I mean when I had vomiting..i started taking medicines..in dussehra..then I started medicines.

I:1 month had passed? R:Yes..1 month had passed.. I:Then you took the medicines?

R:No..after that during Chhath I was in government hospital.. [redacted]. From

here they gave me reports..then I went to government hospital. I:How many days you stayed there? 4 days you told?

R:Yes..4 days. From there they referred me to [redacted].

I:Where were you told you had kala-azar and HIV? In private or government? R:No.I was told in [redacted]

I:In [redacted]?

R:In [redacted]..there they wrote on the paper and gave me. I:That you have both these diseases..

R:Yes.

I:Who told you this? Government hospital? R:The doctor in government hospital. I:What about Dr. Pramod in private?

R:He told me about kala-azar.Here..for 8 days tests were done..medicines were

given.

I:Tell me something more about the treatment. R:Treatment is very good.

I:What do you mean by good?

R:I mean..treatment is good..medicines are good..good food was given. I

became fine..I am infront of my children. I:Behaviour of people there?

R:Fine.

I:People who give you medicines..who come and talk to you..all staffs?

R:All are good. Whoever comes..he asks- how are you? Fine? Tell me if you have any problem. They ask all these there.

I:Have you been discharged from there? R:Yes.

I:After the discharge , have you visited your house?

R:Yes..I come after 15 days for check up.

I:When you go to your house, what work you do there? Has it affected your work?

R:Out of fear, I don’t work now. I:You do nothing? Sit at home? R:A bit of work I do.

I:How are the expenses managed then?

R: Expenses..God..I do one work and manage. I:What? Do you drive vehicle?

R:No..just work.

I:Which work? R:As labourer.

I:I thought few people drive auto. R:No..where are the autos in rural area.. I:No..now a days

R:Work here and there.

I:When they told you to go to [redacted]..how was work going on during that period of 1 month?

R:It was very bad. It was very difficult to even eat. I:What was the difficulty in eating?

R:I didn’t feel like to eat. My family members were worried.

I:You vomited after the food? R:Yes.

I:Would you like to have any improvement in [redacted]? Had this thing been the

other way around, it would have been better..do you think so?

R:No..I mean..whatever the doctor says I will take that medicine..as he says..

I:You don’t think anything more needs to be done?

R:Yes.

I:Whatever is being done is good? R:Yes.

I:Would you like to say something else about the treatment there? R:Where?

I:At [redacted]?

R:My treatment was good. I come after 15 days, my treatment is done, I then go to my house.

I:If you have any questions about the disease, are they answered there?

R: Whatever happened right…I had papers made [prescriptions] just like this.

I:Where?

R:Here.

I:What differences you appreciate in treatment of private and government?

R:In private, money is spent. Right treatment is also not given.

I:Why do you think so?

R:The medicines of government are good. In private duplicate medicines are given.

I:Anything else?

R:A thing of rupees 10 will be charged 20 there.

I:And in government?

R:In government good medicines are given.

I:If it is so, why do people first go to private? R:If they have money, they will definitely go. I:But you too first went in private? In [redacted]?

R:I went to a small set up..quack. I:What do you think about quacks?

R:He sent to government hospital. He said everything will be sold out but if you

don’t get good medicines, your disease will not be fine.

I:Then wasn’t he a good doctor? Sent you?

R:Yes

I:But you are saying that private people are not good. R:No..everybody is not the same.

I:Did he waste your time? For how many days he kept you with himself? R:He didn’t keep me.

I:Tell me something more. First you fell ill and met a doctor..for how many

days did you go there?

R:Approximately ..I took his medicines for 10 days. I:Which medicine did he give?

R:Allopathic medicines. My fever subsided…but recurred. Once I ate it and

slept..I had gastric discomfort and vomiting. I told I will not survive anymore…get me treated somewhere else.Then thry went to the doctor. He wrote that you have kala-azar..consult in government hospital.

I:Did the Bengali doctor tell you this? R:No..Pramod.

I:How was he?

R:We regard him as a very good doctor. I:Is he a quack or other doctor?

R:No..he is not a quack.

I:Ok..private practitioner. He tested and told you have kala-azar..then you went to government..

R:Government.

I:There you came to know about HIV.. R:Yes..there they did tests.. I came to know here. I:Where?

R:In [redacted].

I:That means you didn’t come to know in [redacted]?

R:No.

I:What were the tests done there? R:My blood and all were tested.

I:Where were your wife and child tested? R:In [redacted].

I:They must have told you in [redacted]then.. R:In [redacted], doctor didn’t tell anything. I:They told nothing..only ran the tests? R:Yes.

I:What did they tell about why were they testing your wife? R:They told me that bring your wife and child for the tests. I:Didn’t you ask then?

R:I asked here.

I:Ok..and there you got the tests done without asking?

R:Yes.Then I asked here and they told that they didn’t have anything.

I:Were the tests done here or in [redacted]?

R: In [redacted]. I: [redacted]hospital? R:Yes.

I:There they gave you reports and asked you to go to [redacted].

R:Yes [redacted].

I:But they didn’t tell you what you had? Not even the name of the disease? What did they tell you about [redacted] that why are you required to go to [redacted]? (pause) They sent you to [redacted], right? What did they tell?

R:They asked to go to [redacted].

I:Did they give you any reason about why to go there? R:They told – don’t worry. Go there, you will be fine. I:Before coming to [redacted], you only knew about Kala-azar? R:Only kala-azar.

I:And after coming here, they told you about HIV..in [redacted]?

What did the tell about what is the disease..what causes it? Did they make you understand?

R:They told wrong things should not be done.

I:Whatever you planned earlier,what do you think about it now?

R:What should I think. Whatever is destined, that will happen. Let me go wherever God takes me. Don’t do wrong works.

I:God takes you where? What do you mean? R:As I earn and feed..may that goes on.. I:You want the same ?

R:What else shall a poor person think? Nothing will happen on thinking.

I:Like you told you want to work here now? R:Yes, I want to work at home.

I:You told you want to open a shop, buy cattle and sell milk.

R:Yes.

I:Do you want this or want to go outside and earn?

R:No, I won’t be able to go outside, work under sun. My body is that of a sick. If I stay here, I will work in cool environment, keep sitting on a shop, farming can also be done.

I:When you return back after getting well, what do you plan to do next? R:Work(interrupted)

I:Work always doesn’t mean to earn..like a work of family, you have your

parents, your son..his marriage,treatment of parents..do you think so?

R:I think how they will be taken care of as I don’t do a job.(pause) My parents are there..i have to feed them. I have to think all that.

I:Anything else you would like to say about yourself? What you think about your future?

R:What shall I think about future..

I:What do you want? What do you pray to God? At present, what do you pray to God?

R:Nobody prays for less to God..whatever work I do may God give me success there.(pause) I f I snatch 10 rupees from someone, that will not give me success, I will remain poor. So never harm anyone.

I:Ok..thank you very much.
