# Supplementary material for: Quality of life perceptions amongst patients co-infected with Visceral Leishmaniasis and HIV: A qualitative study from Bihar, India
Source: PLoS One. 2020 Feb 10;15(2):e0227911. doi: 10.1371/journal.pone.0227911 (PMC7010301; doi:10.1371/journal.pone.0227911)
Supplement: S3 File — (ZIP) [file pone.0227911.s003.zip › Transcripts/Patient 8 Female Age 53.docx]

**Patient 8, Age 53, Female**

*I: Interviewer*

*RF: Respondent Female*

*RM: Respondent Male (Husband)*

I: How have you travelled here today? By a vehicle?

RF: Yes, a vehicle

I: What time did you leave from home?

RF: I left home around 9 o’clock

I: You left home at 9 o’clock in the morning?

RF: Yes

I: I see, I see.

I: Did you leave home by a bus?

RF: Yes, by a bus.

I: I see. Was there traffic on the way here?

RM: Yes…

RF: Yes, there was a quite a lot of traffic.

I: Who all are there at your house?

RF: The entire household includes my mother-in-law; my father-in-law has passed away; my husband, two daughters, one son.

I: All your three children live with you?

RF: Yes. One daughter has been married off. One son and one daughter live with me.

I: One son and one daughter live with you?

RF: Yes.

I: How many children do you have?

RF: I had one child. I’ve been married twice.

I: I see.

RF: I had one son from my first marriage. My husband has one son and two daughters with his first wife.

I: You have married twice?

RF: Yes, I have been married twice, and my husband has been married twice.

I: So his first wife…

RF: His first wife died so he married me.

I: And your first husband?

RF: My first husband died too.

I: How?

RF: He died of the same disease.

I: Which disease did he have?

RF: He hung himself after fights with his family

I: So do you have children from your first husband?

RF: Yes, one child

I: One son?

RF: Yes, one son.

I: And how many children from the current one?

RF: None.

I: So you have only one child in all?

RF: Yes.

I (to RM): How many children do you have from your first wife?

RM: Three.

I: Okay, three children then. Do all the four children live together with you?

RF: My first child isn’t there anymore. He died.

I: How did he die?

RF: It happened… He was 9 years old.

I: I see. Was he suffering from any disease?

RF: Yes

I: What had happened to him?

RF: He had rashes over his body. What we call *mata* (local term for Chicken Pox)

I: I see

RF: So I have no children of my own. Only my second husband has children. From his first wife. Two daughters, one son.

I: So the first wife has three children, right? They all live with you?

RF: Yes, they live with us.

I: Will have some tea?

RF: Um… no *(hesitates)*

I: No, please have some

RM: Have some, it’s okay.

I: So from which district are you?

RF: [redacted] district.

I: And which block?

RF: [redacted].

I: And what is the name of the village?

RF: [redacted].

RM: [redacted].

I: So where is your maternal home (*to RF)*?

RF: It’s very far away, a place called [redacted]. It is close to [redacted].

I: Is it in Bihar?

RF: Yes

RM: Yes

I: Which district does it fall in?

RM: *(tries to recollect)*

RF: In [redacted]

I: Oh, it is in [redacted]? Which district in [redacted]?

RF: They call it [redacted].

I: So how did you two meet?

RF: My aunt (father’s sister) lives here

I: Where his (RM) house is?

RM: Yes

RF: Yes, in [redacted]. She is the one who arranged the wedding.

I: So for how many years have you been living in [redacted]?

RF: I was visiting my aunt at [redacted].

I: What is your age currently?

RF: Um… I have my Aadhar Card *(looks for it)*

I: Your name is (redacted)? It says here that you are 53 years old.

RF: Yes, yes.

I: So for how long have you been living in [redacted]?

RF: Around six years now.

I: So you must be 45 – 46 years old when you came here ([redacted])?

RF: Hmm

I: I see. So how did your aunt know you (RM)?

RF: He would visit her house often.

I: I see. Do you work currently? Or did you work earlier?

RF: No, I didn’t work outside anywhere. I stay at home.

I: I see. Can you describe the events over the past one year? How did you fall sick? How did you get to know about your disease? Tell us everything in detail.

RF: I was not sick earlier. Do you know September-October? I’ve been having fever since then.

I: So you have been having fever since one year now?

RF: Yes, it’s been one year.

I: I see. What happened after the fever?

RF: After the fever, I go this disease, what is this called? *(tries to recollect)*

I: For how many days did you have the fever?

RF: Approximately four months

I: I see. Did you not get yourself checked in those four months?

RF: No

I: You didn’t approach anyone at all?

RF: No, no one at all

I: No doctor at all?

RF: I just had a few medicines that a private doctor prescribed.

I: So you did seek treatment from a (private) doctor?

RF: I took the medicines for symptomatic relief.

I: Whom did you go to see for (private) treatment?

RF: In the village itself

I: Were they quacks (“*jhola chap”*) or did you go to a hospital?

RF: No, no. I didn’t go to a hospital.

I: So the fever persisted for four months but it would subside on taking medicines?

RF: Yes, I would become alright after taking the medicines.

I: Okay, so what happened after four months?

RF: When I got myself tested, it turned out to be Kala Azar.

I: When did you get the tests done? Four months later?

RF: Yes

I: Were did you get the tests done?

RF: In [redacted]

I: At a government hospital or a private one?

RF: In a government hospital.

I: Who referred you to the government hospital? How did you know that you should go there (to get tested)? Why did you first go there?

RF: [redacted] *didi* asked me to go to Patna to get treated for Kala Azar.

I: You told us that you were initially seeking treatment in a private hospital in [redacted]. Who referred you to the government hospital there - where your tests were done?

RF: The people at the block sent me there. The people who came for fogging to eliminate Kala Azar, they told me to go to the government hospital.

I: Approximately how many days after the four-month long fever did you go to the government hospital?

RF: Immediately. I spent 20 days there.

I: What happened in those 20 days?

RF: They gave me medicines.

I: Were they only pills or injections too?

RF: Both, pills and injections.

I: You were discharged after 20 days?

RF: Yes

I: Were did you go next?

RF: Then I came here, to [redacted].

I: They referred you to [redacted] then?

RF: Yes. I was admitted for 1 month 26 days here.

I: You did not go back to your village in the middle?

RF: I went back for 2 – 3 days and then left.

I: Did you directly come to [redacted] after the 20 days that you spent at the hospital in [redacted] or did you go back to your village in the middle?

RF: I did go back to the village. Only for 2 – 3 days. I called the hospital in [redacted] from the [redacted] Block.

I: What did they tell you in [redacted] about what disease you had?

RF: They told me that I had Kala Azar.

I: Any other disease that they told you about?

RF: Yes, what is that other disease called? (*pauses to remember)*

RM: She doesn’t understand anything. I’ll tell you. It has been five years since our wedding. She was alright till two months after the wedding. Some days she would complain of body ache. Some days of fever. So I took her to get treatment. Her fever was intermittent. No one told us that she had Kala Azar. Around the time of *Durga Puja* (October) last year, she went to the fairs that come to town. I asked her not to eat out, but she didn’t listen. Didn’t rest at all. She stayed up all night. She contracted an upset stomach. She got diarrhea. I got her medicines, so she got better. But the fever started again. After 2 – 3 days, she became unconscious. She got medicine…and then she had a wound here. You must not know…she got a big wound.

We took her to a doctor. I decided that her condition was not stabilizing. I thought on my own that this wound will not be cured. One person said- do one thing, take her to medical. I got on vehicle immediately and got her to the medical (hospital). The wound ruptured on its own. When it ruptured, the doctor saw it. The doctors gave her injections, IV infusions were also done.

I: By medical you mean the hospital in [redacted]?

RM: Yes, [redacted]

I: [redacted] that is the one?

RM: Yes. After the IV infusions, they admitted her that night. We stayed there for at least 10 – 15 days. The doctors were treating her. The wounds healed. The fever did not subside. We then took her to the college for tests. First the doctors told us that she had H… (*mumbles)* she has that disease. We got anxious.

I: Which disease?

RM: H, H. HPI (sic)

I: HIV?

RM: Yes, HIV.

I: Hmmm…

RM: The wounds were not completely healed by then. She was discharged. They told us that she would have to get blood tests done. We got blood tests done at 3 – 4 places. They checked in the college and told us that she has Kala Azar. We got tests done at the (government) center – where medicines are available – they also told us that she has HPI (sic), we will have to send her to Patna for treatment. So we had to come to Patna.

I: You must have come to Patna earlier too?

RM: Yes, yes. When I retired, the fare to Darbangha from Patna was merely ₹60. Now it ₹150. So we came to Patna and admitted her. In the one month that she was admitted her, eight bottles were IV infused. They called us for a review after a month. They gave us medicines for the next three months. And told us to come back on the 18^th^ after three months.

I: So when you were told that she has HIV at [redacted], what did you two feel?

RM: We got very scared. We have gotten so old and till now I have never heard of such a disease. I have spent my entire life, but I have never heard of such a disease till now. I have never seen anyone else having this disease. Her fever would come and go. Yes, I had heard of Kala Azar. But I don’t know anything about this (other) disease.

I: You were not that scared when you heard about Kala Azar (in comparison to HIV)?

RM: No. I don’t know about her, but I was wondering about how she has become sick with all these diseases.

I: When you (RF) that you had HIV, how did you feel?

RF: I didn’t know what to do.

I: Did you tell anyone else about it? You (RM) must be with her at that time?

RM: Yes, I was with her.

RF: No, we didn’t tell anyone about it.

I: Did you tell your children or anyone else?

RF: No, no, no. We didn’t tell anyone.

RM: No, no one. When they told us, I starting having doubts that I could have it too. So I got myself tested too – in [redacted].

I: So you are safe? You don’t have the disease?

RM: No, I don’t have it.

I: So in the neighbourhood where you live does anyone else have this disease too?

RF: No

RM: No

I: If I were to ask you the question that what are the things required to lead a good life, what would you answer?

RF: Umm…

I: You know, before being diseased the way someone prays to god – some people pray for good health, some for money, some for family and some for a good house. So what would you think? What were the things for a good life according to?

RF: I thought of leading a good life, that I should live well.

I: What do you mean by “live well”?

RF: Meaning that I don’t have any disease. If I do, then that I be treated well for it.

I: I see. And on the home front? How it should be? The environment at home? Or anything else in particular that you would think of?

RF: The house environment…like there are four kids…the way we run it will be the way it will be. Kids are like that. There are four kids, like the kids will behave the way we control them.

I: Okay. How about health? Do you think good health is necessary to lead a good life?

RF: (16:46 – 16:54)

I: Well, what you are saying is true. But good health and all the things that you said you thought make for a good life – why do you think they improve the quality of your life?

RF: If I am able to get treated with good medicines and have good health, only then will life be good, isn’t it?

I: So you’re saying that if you have access to the proper medicines then you will be able to lead a good life?

RF: Yes, yes.

I: I see. So, how do you feel now?

RF: Right now, ma’am, I feel tired all the time. I don’t feel very well. My body aches all over. When I walk, it hurts more. I feel dizzy too. I’m not able to identify people from a distance. It’s all hazy.

I: How was it before your disease?

RF: It was all good.

I: Before you had the disease, how did you like your life?

RF: It was great. I was able to walk well.

I: Would you feel like indulging in your daily activities?

RF: Yes, I would. In all kinds of work.

I: What kind of work would you do?

RF: All the household chores, the work on the farm…

I: Cooking?

RF: Yes, cooking, working on the field – I would do it all.

I: So you would work on the field as well?

RF: Oh yes! I’m from a farmer’s household, will have to do it, won’t I?

I: So you would be doing work like harvesting and such?

RF: Yes, harvesting. And everything else. There’s wheat, rice, green peas, chickpea that we grow. If I don’t do all the work, then who else will? And then there are four children I have to care for, how will I feed them otherwise?

RM: We have to harvest the wheat crop now – it’s beginning to rain now. That’s our food for the whole year. But it’s important to get her treated right now rather than tend to the crops.

RF: If we don’t tend to our farm, what will we eat, ma’am?

RM: One of our daughters is about to give birth to a child soon. We’ve left her behind. What would have been her condition, only god knows – there is no one at home, only my old mother and one unmarried daughter.

I: She (your mother) wouldn’t be able to help out with anything, right?

RM: Yes, she is very old. Each extra day that she lives is a blessing from god

I: All the children live outside, is it?

RM: One son is in Hyderabad.

RF: He lives outside.

RM: It’s been three-four months…three months… since he left. There’s a daughter. She doesn’t work. Just a little bit of help here and there.

I: How old is she?

RM: 12 – 14 years.

RM: I had to get her checked and treated, that’s why I am here.

I: So the disease has had a lot of effect on your work, isn’t it?

RM: Yes, yes. A lot. In the past one month we have lost all the best crop of the year.

RF: The rice crop has failed.

RM: The crops are wasting away. The field is lying as it is, untended. We came to Patna for her treatment.

RF: The crops have gotten wasted. We could just manage to harvest a little bit of the wheat.

I: The fields are your own?

RM: Yes.

RF: Yes, we own them.

I: Wow

RM: We own three *bigha-*s (around two acres) of land

I: That’s a lot of land to own!

RM: We are four brothers [*inaudible*] I have about 9.5 *kattas* of land in Ranchi [*inaudible*]

I: So you can’t tell someone to care of the crops? You have to be there?

RM: No. It doesn’t happen like that these days. Earlier you could find trustworthy people to help out if one was facing some sort of hardship. These days they’re just waiting for us to die so they can usurp our lands and property.

RF: Nobody from the family has supported us. Not one person.

RM: No one came to visit us in the one month that we had stay outside for treatment. Not one person.

RF: Only my mother-in-law and him (husband)

I: I see. So because of the disease you have faced a lot of loss?

RM: Yes, yes, yes. A lot of loss.

I: And mental trauma? You talked of financial loss. How about…

RM: All the food that we grow has gotten wasted. I don’t have any job that pays me so we can buy grains and eat. Whatever crops that grow with the grace of god is the food that five people depend on for the whole year. But that didn’t happen. I was busy with her treatment. A lot of money has been spent too. There is no aid from anywhere.

I: Hmm, I see.

RM: Here and in the block, they told me that something like ₹7100 or ₹7200 is available. SPI ([redacted]) said ₹1500 would be available monthly. But we got nothing.

I: You haven’t got the monetary aid for treatment of Kala Azar till now?

RM: No, absolutely nothing from anywhere (*exasperated*)

I: Neither for Kala Azar nor for HIV?

RM: No, nothing. I told them that I’m a man of few resources, it’s the harvest season; if we don’t get aid, my children will starve to death. I don’t have a regular job; I am a farmer; whatever I grow is what I eat. They said I would get the money. I got tired of running around to get the monetary aid but in vain.

No one is giving us the right reports (updates). No one is giving us the right information. And I know what’s happening where. The government is sending enough money to the sick people. We are informed about that. But then the people working here (*clicks his fingers*). We aren’t educated or connected enough.

I: There must be ASHA workers in your village?

R: These ASHAs are of no use. If you give them 2000 rupees…or keep the same in toilet, they will take it from there as well. ASHA’s kids will survive on that. The ASHAs will not give it to a poor person so that the person will eat. That was 20 years ago…

RF: The floods that happened this year? The water entered inside everyone’s house. I could not light the hearth for 8 – 9 days in our house. Everyone got ₹6000, but we didn’t. We gave account number to the government, but 4-5 brokers tampered with the account number and ruined everything. Half got it, half did not. For 3-4 days, food was not cooked. Our kids used to eat the rice flakes- we stacked 3 beds one on top of each other, and then when the water increased, we stacked two more beds. This was the case, Sir. They were supposed to provide 6000 rupees, but we did not get.

I: What do you think of the treatment that you are getting here? How is the behavior of the staff? Do they behave well with you?

RM: Yes, here they have.

RF: Yes.

I: They have been talking nicely to you?

RM: Yes, yes.

RF: Yes.

RM: We had no problems here.

RF: No problems at all.

RM: The only thing is that all the facilities the government has allotted for the sick hasn’t been made available to us.

I: So what you are saying is that except for the financial aid that you should be getting from the government everything else was available? The drugs and treatment?

RM: Yes, we got the drugs and treatment properly. We have been getting food everyday for two people on all the days that we’ve been here. Reimbursements for medicines that we had to go buy from outside because they weren’t available have also been made.

I: Any suggestions that you would like to make about what we (the government healthcare system) should be doing for this treatment? That is, the facilities that you are getting, the medicines? Anything that you think could be done differently?

RM: It’s by your grace that us downtrodden people are being taken care of. It gives me hope that God has taken care of my troubles.

I: So you there is no need of improvement?

RM: No, no. It’s all fine.

I: Okay. So now that you know that you have Kala Azar and HIV, what is your outlook to life in comparison to earlier? How did you imagine you would spend your life before? The things you would do? What did you want to do before you turned old?

RF: Before turning old I wanted to earn a little money, save up some money, feed my children well, have some money saved up for a rainy day. Save up some money for the children’s weddings.

I: So how about now? All that work is still pending, isn’t it?

RM: Yes, yes.

I: So how do you now look at life? About what you can do and cannot do?

RF: That only you can tell us, ma’am.

RM: We are relying on you.

I: We will tell you all that (related to the treatment) for sure. But what do *you* think?

RF: I want to take the proper medicines, get well soon and that I eat and live well.

I: Meaning that you get completely cured?

RF: Yes, that I get well completely.

I: And that you get back to doing all the work the way you did earlier?

RF: Yes.

I: Working on the farm and all that?

RF: Yes. Working on the farm, looking after the buffalo that we have and all that.

I: You did go home for a couple of days now, right? You are coming from home now, isn’t it?

RF: Yes.

I: Are you able to do any work at all?

RF: Yes, I am to do work. But like before. I tried to mould the dung cakes by hand but started coughing up and my body was aching all over.

I: So are you able to do any work on the farm at all?

RF: Not that much, but yes, I am able to do work on the fields.

I: Good enough, you are able to work on the fields albeit a little less than before.

RF: (*agrees*)

RF: This harvest season I wasn’t able to do anything at all though. I was sick.

I: After how many days are you coming to Patna now?

RM: (*pauses to think)* 3 months

I: So in these three months what did you do at home? What did your average day look like?

RF: At home… *(cut off)*

RM: (She) Takes care of some chores at home, remove the weed on the farm, feeds the buffalo, that’s about it.

I: So you remove the weeds, feed the buffalo…

RF: There was some black mustard and mustard crop too. I cut them and separated the seeds out too.

I: How about cooking?

RF: My younger daughter is at home, she takes care of it. She is a child, so on the few days that she doesn’t do it *(cooking)* I do it.

I: So because of the disease now you have become lethargic while working?

RF: Yes, indeed. My hands and legs pain a lot. I have lost my appetite too.

I: Would you like to tell us anything else about yourself to us?

RF: About the disease? What else do I tell you ma’am? This is all that I have to say. There is lethargy and body ache. My body doesn’t work well now.

I: So you have been taking medications for a while now? You stayed at the center for 1 month 20 days? Are they calling you every 3 months?

RM: No, ma’am. *(pauses to recollect)* They called us after a month. We went back after a month.

RF: Yes, after a month.

RM: Yes, after a month we went back for tests. They gave us medicines for three months and asked us to come back after three months. On the 18^th^. The medicines that she (RF) is supposed to take at night, for a month – we would get those from [redacted]. If she (RF) wasn’t in a condition to get it herself, I would take the card and they would give me the medicines.

I: About how much time elapsed between the first bout of fever and taking her to [redacted]? Just an estimate?

RM: Um… approximately, two years.

I: Two years?

RM: Yes. It has been five years since our wedding. She was okay for about two months after the wedding.

I: So she has been having complaints (of ill health) since five years?

RM: Hear me out…

RF: It wasn’t very obvious at that time

I: He (RM) is telling us that you have been having complaints (of ill health) from two months after your wedding…

RM: Hear me out, I am telling you… She got married to me, then she came to my house, she was okay for two months. But then we are poor people, what will we eat if we don’t work. So we continued to work. I was in Jharkhand at that time.

I: You did not bring her to [redacted] after the wedding?

RM: Yes, she was at [redacted]. I was working in [redacted].

I: Were you doing farm work in [redacted]?

RM: No, no. I was working with vehicles.

I: What kind of vehicles? Trucks?

RM: No, buses.

I: So you were a bus driver?

RM: Yes. I was in [redacted]. I got a telephone call from home then about her not keeping well.

I: So she was in [redacted] and you were in [redacted] at that time?

RM: Yes, yes.

I: How many months after the wedding did you go to [redacted]?

RM: Um… *(pauses to count the months)* Take it to be around 3 – 4 months after the wedding

I: So she was in Balwa and you were in [redacted] where you got the call?

RM: Yes, yes. They said she isn’t well and very disturbed. So I said I would be back in 10 days. I made reservations. I asked them to start getting medicines from the local doctors and shop for her in the meanwhile. She got well for a while but then fell sick again. I did not know that it was Kala Azar at that time, otherwise I would have gotten her admitted here.

I: So it took you four years to learn that she has Kala Azar?

RM: No. We got to know about that only during this time.

I: This time as in at [redacted]?

RM: Yes, yes, yes

I: So that’s four years, isn’t it?

RM: No, no, no.

I: You’re saying that it’s been five years since your wedding. And that she has been sick since three months after the wedding?

RM: She fell sick two months after the wedding. Then she under medication. The fever went away. Then she fell sick again and was under medication again till the fever reduced.

I: So for how long did this happen?

RM: Um… about a year and half.

I: So this pattern continued for 1.5 years?

RM: Yes. When she fell extremely sick, then I brought her to [redacted]. That’s when we got to know. Her blood tests happened there.

I: Before going to [redacted], how many doctors did you consult?

RM: One or two local doctors. You know what happens with these local doctors – they give you can injection or two and you get well for a short time. Then when you fall sick again after a month or two, you go back to them.

I: So you were seeking treatment locally for 1.5 years?

RM: Yes.

I: And even after 1.5 years when she was still sick, then you brought her to [redacted]?

RM: Yes, yes, yes, yes.

I: So how long it has been since the onset of her disease, in your knowledge?

RM: I can’t tell you that.

I: Since when have the bouts of fever started?

RM: That I’ve already told you

I: Yes, two years you said.

RM: Before that, I wouldn’t know. You will have to ask her.

I: I meant since when have the symptoms begun?

RM: Yes, yes, I’ve told you everything

I: Yes, yes.

RM: I don’t know anything about her disease before I got in contact with her. You will have to ask her that.

I: Would you the bouts of fever before (you got married to) him too?

RF: No, no. I would scoff at others who had fever or headache.

I: I see *(laughs)*

RF: I would ask my mother, sister and sister-in-law how they would have a fever or headache so frequently. My sisters-in-law would tell me that I wouldn’t understand till I would be suffering from it myself.

RM: She has never even had a fever before. Only once actually – she was bedridden for 8 – 9 days. Nothing ever since that episode till this disease happened.

I: So the treatment for Kala Azar at [redacted]- it continued for 20 days?

RM: Yes.

I: And then when it (HIV) was found then they sent you here?

RM: Hmm…

RF: No, when I went to the center then I learnt of it

RM: *(Scolds her)* They did blood tests at the center

RF: Yes, that’s how they did it (confirm diagnosis).

I: Which center?

RF: The same place.

RM: The place where the medicines for HPI (sic) are given.

I: Which disease were you told of first – HPI (HIV) or Kala Azar?

RM: We were told of Kala Azar first.

I: I see.

RM: The doctor there sent us to the center for tests and treatment. After they did tests there (center), they told us that it was HPI (sic). At the college they told us that it was Kala Azar.

I: So they first told you about the Kala Azar and later the HPI (HIV) diagnosis?

RM: Yes.

I: Okay, I see. So first you were treated for Kala azar, then they sent you to the center for tests and when she tested positive for HPI (HIV) there the treatment for HPI (HIV) began?

RM: Yes, yes.

I: Then they sent you to Patna?

RM: Yes.

I: Okay. Okay. Anything else that you would like to share?

RF: No, nothing.

I: Thank you very much.
