# Supplementary material for: Quality of life perceptions amongst patients co-infected with Visceral Leishmaniasis and HIV: A qualitative study from Bihar, India
Source: PLoS One. 2020 Feb 10;15(2):e0227911. doi: 10.1371/journal.pone.0227911 (PMC7010301; doi:10.1371/journal.pone.0227911)
Supplement: S3 File — (ZIP) [file pone.0227911.s003.zip › Transcripts/Patient 9 Male Age 36.docx]

**Patient 9, Age 36, Male**

I: Yes, so who all are there at your place, [redacted]?

R: There are my kids living with me..

I: Your wife ? your parents ?

R: Yes, my wife; My parents passed away.

I: So how many children do you have ?

R: Four, two boys and two girls.

I: So how old is your eldest son, eldest child ?

R: The eldest one...He might be around 10-11 years old.

I: I see, 10-11 years...So do you have your own house ?

R: Yes

I: So what work do you do ?

R: Motor vehicle.

I: Oh, so you drive? What vehicle do you drive? A truck ?

R: I drive a truck.

I: So where all do you drive your truck ?

R: I used to drive the Kolkata-Pune route and now [redacted].

I: So for how many days now have you not been driving ?

R: you could say that past 1-2 years have been in a slump...been quite a problem.

I : Oh, I see. So how would you describe how was this past year for you ?

R: this past year…

I: yes, please tell me about it.

R: This wasn’t a good time for me..not good

I: Why wasn’t it good for you ?

R:My life got spoiled, you see, my body like… one day I went home after driving and after that, I came home, and then suddenly, a fever occurred.

I: Tell me all the details about your illness, when were you last alright? When did you start experiencing any trouble?

R: See, I kind of actually knew about HIV 2-3 years beforehand

I: So you knew about HIV 3 years ago? How did you come to know about it?

R: I had some issues with my nose and some issues with my stomach..

I: what kind of issues?

R: I was experiencing pain in my stomach. I got some treatment in [redacted].

I: Were you also experiencing any upset bowel movements or only pain ?

R: Mostly pain, and was having an upset stomach frequently…

I: What were your issues with your nose ?

R: I still have problems with my nose. I have even requested sir to kindly get me relieved of these problems of mine.

I: So do like have trouble in breathing ?

R: Yes, there is a difficulty in breathing, there is frequent ulceration in the nose. I have a problem of sinus. One day I even got a CTS done in the ENT department, even spent 20-25 thousand rupees in [redacted]. There he told me something about my nose..and even there I was screened for HIV.

I: How old is this incident ?

R: about 3-4 years old.

I: So 4 years back in [redacted] when you went to get your nose check-up, they got you HIV tested ?

R: Yes, I was driving in [redacted] at that time when my nose started bleeding. And then I called up my boss who resides in [redacted] and told him about my worries. He told my manager to get my check up done in a hospital there. Since, I’m a driver, I was screened for HIV. There, they initially kept the information of the report a secret.

I: They did tell you about the results of the test, right ?

R: Yes, they did tell me about it,but kept it a secret from others because he told me that being an HIV positive, I would be taken away to the HIV hospital here and since I’m not a resident of around here, it would be troublesome for me. He told me that I should go home and get myself treatment from there. They told me this would be a better option. And about the nose they told me that there a 2-2.5 cm mass present upwards and this infection is dangerous and this requires treatment as well. But he told me that since getting treatment at home is a better option, he did not mention this on the file but did tell me about me being HIV positive

I2: Did you know at that time what are the means of spread of HIV ?

R: Yes, he did tell at that time...and even I did something wrong...sometimes humans do commit mistakes...in my line of work, what should I say, even if a person does not want to, he might just commit some mistake. Not only this, but once in [redacted], for licensing purpose I had to get my blood grouping done, and at that time they even used common needles and how did it actually spread is still unsure to me.

I: How did you feel about it when you were first told that you were HIV positive?

R: I felt bad at point but I was quite alright at that time though but then later problems started to develop.

I: Did you feel out of the ordinary as of what had happened to you ?

R: I would say I didn’t quite feel much about it at that time. I was more worried about my nose at that time.

I: So you weren’t bothered much by the news of being HIV positive at that time?

R: not exactly...I knew there weren’t many facilities available at that time, I even heard that a patient of such sort in [redacted] was killed by injecting a poison. But now there are so many such patients and even the government is providing good facilities in every state now.

I: so you knew about this? You weren’t worried much ?

R: not much. In fact, I rather gave up on my body and thinking about my kids , I didn’t pursue the treatment.

I: But even after you knew about this , why didn’t you go for treatment ?

R: I didn’t try because I knew this body is of no use now, I am to die anyway and till I die I just wanted to drive and work for my children. My house wasn’t complete at that time, I struggled and worked hard and completed my house.

I: so for three and half years after knowing these facts, you didn’t do anything ?

R: No, I didn’t do anything.

I: so when did you start having problems again ?

R: it was again during my duty hours in [redacted] after 2-3 years.

I: 3 years after that ? So how many months ago from now would you say that would be ?

R: About 6 months ago ?

I : So till about 6 months ago , you felt you were completely fine? You had no issues while driving your truck too ?

R: Yes, it was all fine.

I: So like you went ahead with your daily work and other things for 3 years without any medication or treatment ?

R: I used to take medication privately for my nose problems…

I: But you didn’t take any medication for HIV ?

R: No, Nothing of that sort, I just used medication for my nose problems. Then there was body weakness and was I was applied drip but as soon as I felt fine, I reported back to my duty.

I: so you got all this done in a private setup ? how much did you spend for it ?

R: Quite a lot actually…

I: Still...what would you estimate, how much amount would that be ?

R: Prolly in lakhs...somewhat over 2 lakhs, I guess...I still struggled hard for my children..not caring much about my own body...I knew I was going to die and I wanted to do something for my kids before I died

I3 : didn’t you think that if you got yourself treated, you could have prolly done more for your children ?

R: I wasn’t aware of many things at that time.

I: He claims that even cows are killed by injecting some needles!

R: Yes! In Andhra patients diagnosed with HIV are killed off by giving them injections!

I: He Claims he’s heard such things happening

R: Under that impression, I thought I should keep this a secret and didn’t even tell this to anyone at home also

I: Not even your wife ?

R: No, not even my wife, I mended a couple of my habits but didn’t tell anyone about it.

I: So what exactly happened 7 months ago at [redacted] when you were driving out there?

R: So when I came back from [redacted], I felt quite irritated, I felt that weakness. The weaker a man gets, the more irritated he feels. I even had a little tension growing between me and my boss. I told him I will not drive your vehicle- you take your vehicle back. Then, I went to Baleshwar- my boss was younger than me- and he begged me to drive. Even if you abuse me over phone because you are a nice person, I tolerate that. This is humanity. You are not a liar, so he said this and said drive the truck. Come to [redacted]. I told him no, it won’t work now. If I’ll go home, I’ll go home. I felt okay for the 15 days that I spent at my home. Had some good meals at home. But then papules started appearing on my wound. Hm? And then it was spreading. So during this period, the treatment started in the hospital. So they gave me ointments and medicines, so the papules got better. It had barely gotten before, and my fever had not gotten better. Day by day, day by day, my stomach started swelling and the rest of my body started getting wasted. In the end-

I: For how many days did this happen ?

R: For about two months, anyone who told me to get treatment, I just used to scold them off

I: What did you think ? What were reasons for not getting any treatment ?

R: I had decided quite firmly that I didn’t want to live anymore since I’ve already constructed a house for my children

I: so you were quite single-minded about dying ?

R: Yes, pretty much. I used to even scold off my friend, my wife and even my younger sister. I told her not to cry and that she can cry as much as she wants when I die. So one day the contractor who built my house said he is going to take responsibility for me. By that time my urine reports came in and they found some infection and then I started feeling a lot of trouble while urinating.

I: Did you feel pain during urination ?

R: Yes I felt a lot of pain while urinating, excruciating pain actually.

I: what was the status of your hunger?

R: I did not use to feel hungry at all, I almost ate nothing at all. Then that friend of mine, the contractor told me that he is going to take care of me, he will look after all the finances. He said that if I die, he won’t ask for a single rupee from anyone and if I lived he was assured that I would eventually pay him off later. He trusted me so much. I tried to rebuke him as well but he was quite persistent. We then hired a car and went to [redacted]. [14:32-14:38]

I: So you did tell your sister about HIV ?

R: She eventually did get to know..

I: So you only told her about this when you were staying at your home for 2 months ?

R: Yes, eventually I only told everything about it to all the family members.

I: Even the kids ?

R: yes, they also know about it now.

I: So you felt like if you’re about die , you must at least explain the reason ?

R: I did tell them that I had a fatal disease and they should not get worried

I: oh, so that’s how your sister knew about someone getting treatment in [redacted]..

R: Yes someone from my sister’s village was getting treatment in [redacted]-

I: What village does your sister belong to ?

R: [redacted] village.

She started crying in front of the neighbours about me and started complaining about how

adamant I was for not getting treatment. Then that lady advised her to get me sent to

[redacted]. Hmmm! About six months back I once again got HIV testing done at [redacted]

hospital, [redacted]. So the guy said that the whole family should undergo screening and

that I should even bring in my wife and my youngest child. These two got their results as

negative while mine was positive. He told me that there wasn’t much medicine availability

before. He told me that people with CD4 levels above 500 will not get treated, and my levels

were 544. So he didn’t start with the treatment. He told me to eat well and try to stay healthy

and that I didn’t need medicine then. But when I came back from [redacted], my body kind of

fell…I had high fever and was tested for malaria and then local doctor said it was malaria and

gave me medication for malaria but it did not get well. Day by day, my blood got burnt away

and my body completely got wasted. In the end there was urine infection, that is where I felt I

would die in another 2-4 days. Then my sister came in from her in-laws and also got me the

address in [redacted] and convinced to go to so and so place in [redacted] even if I was

not advised for treatment at [redacted] hospital, [redacted]. I’m forgetting the name of the doctor

right now. She advocated for him saying, he can even make dead into living again. So my

contractor friend arranged for funds and we went there then.

I: So was that a government setup ?

R: no it was a private clinic and attended to HIV patients. And so I was admitted there for about 7-8 days. There also we spent about 80-90 thousand rupees. I was administered fluids and 3 units of blood. After that my weakness and to quite an extent my problem of urine was relieved, but then again I told him that I still feel discomfort in my stomach, my stomach still aches. He said that day he’ll investigate the swelling for kala azar and told me to say assured that he’ll cure me completely. The investigation revealed it was indeed kala azar. He said to me, [redacted], I’m sending you to a place where you won’t have to spend a single rupee and there would be food and accomodation for two and you must go along with someone only. Not only that, I’ll send some attendant from here who will get you admitted and then only will she return. I was afraid and asked him if he could arrange for the same facilities as the place he was referring to.

I: So you wanted to stay back ?

R: I told him why would you send me to [redacted], sir ? I’ve already spent 7-8 days here and now again do you want me to have the same suffering in [redacted]? He said I’m sending you to a place where you’ll be treated completely free of cost and that too with proper care, don’t you worry at all. I told him I was unsure about this, he said I’ll send someone along who’ll get all the tests done for you, will take care of the reports and will get you admitted to the hospital and will then only return here. He said If you just feel like paying the bus fare, then only you pay for it, otherwise you don’t even have to pay for that. He said that I’ve already spent around 80 thousand and if I wanted to stay here, it would cost me about 3-4 lakh rupees more. He said I want to save you and also take care of your pocket since you already have kids to look after. He said you just go there and do not worry at all. After that we came here and I let go of that lady...her name was [redacted] or something. She told me that since I was literate and could read as well as so could the person accompanying me, her services not actually required and I should not trouble her for menial work. She handed me the papers and told me to get the investigations done and go to [redacted]. She told me once I reach there, I should give her a call so that she could call some [redacted] who’d come and receive me and get me admitted. I did as I was told, I came here, I called her up, [redacted] came something after lunch and got me admitted and we began with my treatment. So basically, my swelling was quite large. So when they extracted blood from here, I felt like breaking glass windows and jump off the third floor and die, it was paining like anything. I even lost 2-3 kgs of weight in the past 3-4 days. At the end, the staff nurses and other health staff too were quite worried, and that this is the first patient who has fainted. And I fainted and even the doctor who was doing it became nervous. Even the doctors were worried, that while extracting blood if they have made any mistake. They even called their senior, and a team of doctors was sent. They wrote me double ultrasound but that day the guy there told me, it won’t be possible today and that I could come tomorrow. Then I got my ultrasound done and was told that the swelling has increased much and that when I get 2-3 bottles of liquids infused , it’ll get better. The same actually happened, after the i.v. infusion, I gradually started feeling better.

I: Do you know any more people from your workplace or where your village is at [redacted] who have this same problem?

R: No

I: Anyone with HIV or Kala azar in your village ?

R:not exactly, even kala azar is not there in the neighborhood. [redacted] told me that I should explain this to people and give details about it. If there is someone with HIV, they are getting their treatment done.

I: So is there someone with HIV ? Do you know about them ?

R: Yes I do know about them..

I: About how many people would be there if we talk about [redacted]?

R: A lot of people actually have HIV…

I: 10 people ? maybe 15 ?

R: Not 10-15, probably 2-4

I: What would be their approximate age though? Everyone less than 40?

R: There is one who is older than me, even he got worried when he saw that it looked like I was about to die. He said to me that I wasn’t getting any treatment and if I continue to not get any, even he’ll die. I told him I wanted to get out of the webs of the world, and I didn’t want to do anything. He started crying and told me that if I die, he’ll get a heart attack.

I: From where is he getting his treatment from ?

R: He’s getting it from [redacted].

I: From the [redacted]?

R: Yes, from the [redacted] hospital, even I get my medication from there only.

I: So you are delivered medicines every month ?

R: Sometimes I take a vehicle there, sometimes my madam gets it for me. She goes and brings it. Sometimes, they say that the patient should always be there, so then I stop the vehicle somewhere and- if I went to [redacted], I stop it at [redacted], then I come to [redacted], take medicine, wait 1-2 days at home, and then leave.

I: So we’ll ask you a different question now. You’ve explained us quite well about your illness, let’s talk about a good life now. What do you think according to you are the things that are required to live a good life ?

R: Now what can I say...my life will be incomplete now. I would just like to request you all to talk to the institution to make facilities-

I: We will talk about the institution later, right now let us say that you do not have any disease or when you did not have any disease, what did you think about all things you wanted to do for a good life ?

R: Nothing in particular as such, just wanted to get my children educated and help them succeed in life..

I: Just wanted to help your children by making them educated ? Anything else ?

R: construct a own house and such things…

I: RIght, a good house then ?

R: Yes, a good house, good education for children, these are important things to me.

I: Did you ever think of health ?

R: Once my health started deteriorating, I myself started further deteriorating it.

I: So you did not give any value to your health ?

R: I did not pay any attention to health. Once when I had made up my mind 4-5 years back that i’ll soon one day die due to my disease, there was no looking back.

I: you must have thought of your family life ?

R: everyone thinks about it that it should be good, no one would want it to be bad.

I: So whom did you think good of ?

R: I used to think good of everyone, my wife, my children too but I got such a disease that I am facing such problem now

I3: Suppose, you were not ill for these past 4 years, and are alright now, what all would you have done except construction of your house?

R: Getting education for children has been my priority, education these days is so expensive, I wouldn’t have been able to do anything beside this anyway…

I3: So you have no other desires ?

R: Desires...would have just expanded my business along with my children.

I: What do you think of your own life ? How do you find your life ?

R: I just think it is going fine. Since I have taken up this treatment, I just make a call whenever something happens and get treatment free of cost.

I: Have you been able to work ?

R: I worked for this one month but then again started getting these pimples which got me worried that if I fall somewhere it’ll even cause trouble for my children. All four of my children are currently studying and so the expenses are quite high, so I called up [redacted] and told him about the redness in my eye and other problem that I was experiencing in my eye and asked him for his advice. He asked me when did I have my next appointment, I told him it was 27/05. He said he’ll get back to me after talking to the doctor. He then told me to come right away and they’ll attend to me.

I: So you think the progress is not up to your expectations ?

R: No, not exactly…

I: you had all these eruptions and still went to work...I have to commend you on your courage!

R: I was driving for one whole month, drove from [redacted], that’s where the problem began. Even got a new vehicle. Thought if I would drive for 4-6 months, I would be able to take care of all my loans. There was an expenditure of 1-2 lakh rupees on my health care as well…

I had to do something to fill up this hole now and now till the time my eyesight was fine, i obviously could not be resting…

I: So you were trying to tell me about this treatment plan of yours...How is the environment there ? What is the behaviour of the staff, the doctors ?

R: Right now, the treatment is going fine…

I: How is the behaviour of the staff ?

R: The staff is also good. Now why would I lie? Why should I disgrace anyone ? I just feel I have been given a new life. If I hadn’t gone to [redacted] and [redacted], my life would already have been over by now. Everyone who would come to visit me would pity at my condition and think that I was about to die, even at one point I was quite certain that I am destined to die soon. I didn’t want to engage myself in any worldly things. My mother died, my father died, my children will go on after me, this all is the chain of life…

I: So you don’t have to say anything about your treatment part ? You think it is all fine ? Anything you would like to change about the hospital where you are getting your treatment ? Any scope of any kind of improvement ?

R: I have requested the institution, I have requested them today as well, made my requests to the staff, to the doctor that if something could be done regarding my nose and my pimples. That they should get any required investigations done like the CT scan, and take me to a throat specialist. Now that I would be living more, why not improve this quality of life for me ? They told me to wait for the reports and if it is required, they’ll take me to a specialist for a check-up. I even get reimbursed for the medicines worth 5-10 rupees on presentation of original bills.

I: I see, they have taken samples from your nose, I hope they come a conclusion quickly...So what more do you want to do in your life right now ? Like for example you said you wanted to repay your loans…

R: Yes, I wanna repay my loans, get education for my children-

I: You’ve already built a house for your family, what else do you think is left to do ?

R: There are a lot of things left to do actually...now why should I lie, I do have my own house but

I still have to install a gate in my house, I want to complete that unfinished house, get it plastered, get windows made out et cetera, get my children happy and well educated…

I: How old is your eldest child ? 12 years ?

R: Yes, 12-13 years.

I: There is still time for their marriage then… The eldest one is a son ?

R: Yes, the eldest one is a son, then there are two daughters in line and the youngest one is again a son. Each and everyone of them is studying in private school.

I: They are studying in [redacted] only ?

R: No no, it’s a private school nearby my place.

I: So any other purpose beside giving them a good education ? Anything that you seek from god ?

R: Definitely I pray to god to grant me good health so that I see my children succeed and that they turn out well educated. I feel this is my duty to get them married as well but if something happens before that, no one can stop that.

I: You used to think about all this before also and even now you think about the same things, do you think there has been any change in your thoughts due to the disease ?

R: Yes, some change indeed, like at this point I’m doing rather okay, at one time I had just thought of dying. I was tired of life but now I feel fine. Even my body does not feel weak anymore, although it does happen sometimes but the major problem that I have is that of my nose and these pimples that keep me worried.

I: What would you say, do you think you’ll be able to do all those things that you thought were feasible for you 4 years ago ? Do you think it would be possible for you to do it now ?

R: See, this is all in god’s hand, if I get good treatment, I will continue to put in my efforts…

I: So do you feel you’ll be able to do it even now ?

R: Yes! Definitely! Only if I’m in good health...If I’m not healthy, then it’s not possible.

I3: You did think that you were going to die due to this disease, what were your thoughts about what would happen to your family members after you were gone ?

R: Yes, that was the problem, I didn’t think of anything. All I was certain of was death-

I3: What did you think would have happened to your children?

R: Nobody would have looked after them, it would have been all over once I would have closed my eye-

I: So you would not have been here to look after them-

R: Once I would have died, I could obviously not look after them. How would I know if someone had started begging or if someone was using airplanes as means of their transport? There was just one thought in my mind that living this kind of painful and sad life is a waste.

I3: Do you ever feel any remorse that you have this disease and should not have gotten it ?

R: Yes, I definitely feel sad about it-

I3: that you wanted to do more for your family ?

R: Definitely I felt sad on how I got this disease. How did this happen to me ? There is this good friend of mine as well, he’s a [redacted]. Once he got to know of this, he got me admitted at [redacted] then. Even then, I didn’t have my treatment done because secretively, I wanted to build a house first. Whenever he used to meet me, he’d always ask if I was taking my medicine regularly? I used to lie that yes I was taking my medication. But when my condition got worsened , he asked me if I had stopped taking my medicine ? Then someone told him that I didn’t take any medicine at all since [redacted] people didn’t admit him and that they were referring him to [redacted]. He got this written from the [redacted] that I was his so and so staff...all this because he is a childhood friend of mine. I wasn’t from a family which was quite literate themselves, even my parents didn’t make me study much. He was from a rich family, he studied and became a daroga. His brother is also in the army. He still loves me due to his compassionate nature.

I: Would you like to say anything else ?

R: What special would I say now? I would just request the department to relieve me of my bodily pain because I want to work for my living and not be in debt of anyone. If my body gets alright then I’ll be able to even get education for my children. I was told at the department that there is so and so grant for the HIV patients and I even did fill the form but no one listens

I: So you didn’t get any money ? Not even from [redacted] district ?

R: No money at all, even when I did fill up my form.

I: And for Kala Azar ?

R:Even I heard that there is some housing support for Kala Azar patients but if they have HIV and Kala Azar, they won’t get it. So I said okay if I don’t get it…

I: That means you’ve not yet received any money or any facility of this sort ?

R: I haven’t received any such aid from the government but whenever I visit them, I am given 600 rupees for my to and fro fare for 2 persons. I was told while coming here that I would be compensated for this as well but now if I will actually be compensated is up to you people…

I: Would you like to say anything else ?

R: I just wanted better treatment for these pimples-

I: That treatment is going on ,right ?

R: Well, yes…

I: Thank you so much.
