# Supplementary material for: Quality of life perceptions amongst patients co-infected with Visceral Leishmaniasis and HIV: A qualitative study from Bihar, India
Source: PLoS One. 2020 Feb 10;15(2):e0227911. doi: 10.1371/journal.pone.0227911 (PMC7010301; doi:10.1371/journal.pone.0227911)
Supplement: S3 File — (ZIP) [file pone.0227911.s003.zip › Transcripts/Patient 1 Male Age 60.doc]

**Patient 1**

Age: 60, Male

*Notes: Patient was very hesitant to talk and didn’t seem to understand questions too well, despite asking in different ways. He was also hard of hearing, which made the interview process a bit more difficult. He chose to have his wife accompany him in the room for the interview.*

*R: Respondent*

*R2: Respondent´s wife*

I: Okay, so can you tell me a little bit about yourself? [*pause*] Where are you from?

R: My house is [redacted information].

I : I see and who all are in your house?

R: House is chazupatti.

I: I see. Who all is in your house?

R : There is one girl in the house, she will be getting married right away. There is no time.

I : No time. When is the wedding?

R : On the 30th approximately.

I : And uh, how long have you been staying there?

R : At home?

I : Yes.

R : It’s been more than one year at the house.

I : More than one year. And where were you before then?

R : I was in [redacted]. I used to work here and there, brick laying, and all. Now, I cannot do such work. My health is bad- on top of that, I sat on my house. I got it checked at [redacted] hospital, so they said that you should go to Patna. Then they sent me here from there.

I: When did this happen? When you first came to Patna? For treatment- when was this?

R: It has been two months.

I: It’s been two months. So two months, and then you got treated in Patna? How was the treatment there?

R: Here?

I: Yes yes.

R: The treatment here is good.

I: What did they do? What did they do?

R: They administered IV, needles, this tablet that you have- I got it in a timely manner. I got the food also in a timely manner. No kind of difficulty.

I: I see, I see. And was it explained to you what kind of thing you are getting treated for? Why you are here. Was all this explained to you?

R: [*pauses*] No not all this.

R2: For Kala Azar.

I: For Kala Azar, I see. So were you told?

R: [Redacted], the government hospital that is there- if you get treated for Kala Azar there, then you get money. Hm? I still have not gotten it [*chuckling*].

I: You haven’t gotten it? Why?

R: Where have I gotten it? I only got travel fare.

I: I see. You haven’t gotten the treatment amount yet?

R: Whatever I got, I am telling you.

I: Okay tell me a bit about your previous year. How did the previous year go for you? What all happened in the past year?

R: Last year, first I had a fever in the beginning. The fever happened, then after working every day – in the night, I sometimes ate food, sometimes did not eat food, sometimes I would not feel hungry…mhm? I used to fall asleep. This is it- what else can I say?

I: I see, I see. And the effect on your work?

R: Huh?

I: How did this affect your work?

R: Work…I do and don’t do. if someone tells then I work in field and earn Rs. 50 for that so what will happen with that.

I: Hmm hmm, so now what do you do?

R: Now, I do not do anything.

I: Now, you do not do anything, I see, I see. This, HIV-Kala Azar thing you had- how did you first find out about this?

R2: Here only.

I: Hmm. What was told to you about it ? What do you understand about this illness of yours? Can you tell me a bit?

R: First, I was going to [redacted] to get it checked out. So there’s a sahiya there- know the doctor a bit there- so I showed it to him. After showing him, he wrote a report. After he wrote the report, he had done the investigation for one hour. I got it inside. So I got it and he said don’t go to [redacted], go to [redacted] hospital. Ok? There this doctor saw me. He also did a blood test and everything. He told me you will get treatment for Kala Azar from [redacted] hospital. I had two illnesses- one was lymhadenopathy so I got treatment here. I got treatment for lymphadenopathy too. But, the treatment for that got transferred to [redacted]. So I had gotten papers from here, I submitted them in [redacted], so I got a book there. The book has my photo in it [*inaudible mumbling*] he said it’s medicine for one month. In one month, the medicine will be over, then come and get medicine. Then what Sir asked me was- when you were in [redacted], and you got treatment for Kala Azar, how much money did you get? I said Sir I got 100 rupees- nothing more. He said why? I said how would I know? We are new people.

I: So you were telling me about when you first found out about the illness, okay? So have you told anyone in the family about this? About the illness?

R: No…they just found out now when the investigation was done.

I: Who knows? Who all knows? Who knows in the family about the illness?

R: The investigation that was done, right? So she was with me [referring to his wife] and as we were sitting, and she heard. Then we shifted from there to here.

I: Ok but the other people that are in your family- like you must have children, or your mother and father, or brother and sister-

R2: No, no I don’t have mother and father. [*Recording was paused for a bit*]

I: Sorry sorry, so you were telling me about your mother and father, or your brother and sister- do you have a brother or sister?

R: No I don’t have a brother.

I: Sister?

R: I don’t have a sister either.

I: And mother and father?

R: Not there.

I: Ok so do your children know? That there is an illness and there is some problem?

R2: What do I say? They know it’s kala azar nothing more.

I: And the people who stay in your neighborhood? Your friends? Do they know?

[*shaking head*]

I: They don’t know? Why did- have you ever tried to tell them ?

R2: Kala Az- …no not to them.

I: So you have Kala Azar also, and HIV also. Both, right? If the people in your neighborhood found out about this, what would they think about this?

R2: Now what would they think- that they will go to the doctor and show. [*inaudible*]

I: You tell me a bit. What would they think?

R2:what would they make me understand – the doctor told that if you go from here and find someone having fever then send him to nearest hospital and if they don’t get treated there then send them here. So, two people are getting treatment by that way.

I: Hmm hmm. But uh, like you have your friends. What do they think about this illness? What do the others say?

R2: Nothing. What would they say?

I2: Like they tell you behind your back too, no? No one says anything to anyone’s face. Behind your back?

I: Nothing?

R2: Who’s going to tell me anything to my face? No one does that.

I2: Yeah no one says anything to anyone’s face. They will say behind someone’s back only.

R2: Yes.

I2: No one has the courage to say anything.

I: But it’s not like- they all interact with you? No problem with that?

I2: they talk to you? Come to your house? You go to their house, they come to yours?

[*silence*]

I2: Like it happens in society, going here and there to each other’s houses.

R2:yes, why don’t people go in that way .Anyone is going to anyone’s house (inaudible)

I: OK let’s talk about something else. Tell me how this illness has affected your life? Meaning, what else has it happened like- ?

I2: Are you feeling anything? Something different? Are you feeling?

R: First nothing had happened to me. That time, nothing had happened. This has happened suddenly. Since one year, I was having fever from inside, but I could not tell I had a fever. Like, little little- I did not feel like working. I used to fall asleep. Hm? when I got relaxed I worked a bit, sat quietly in the house, just sitting on the porch, not feeling like talking to anyone. Had come in the evening-

I2: Did not feel like talking to anyone?

I: Did you talk to other people?

R: Yes.

I: You did? Also during the illness?

R: [*shaking head*]

I: No? Didn’t happen. Now how is it? Have you felt differently at all?

R: After getting it looked at, I found out everything.

I: Yes, but after that, after you got the treatment done, have you noticed any difference in your life?

R: No, no difference.

I: No difference? You still feel the same tiredness? And no difference in talking to other people?

R: No, no difference. No difference in that, but I hear a bit less from my ear.

I2: Like the treatment is happening, so meaning, something must have improved, right?

R: No, no clue [*did not understand the question*]

R2: Improvement, improvement! Yes, there is.

I: Tell me, tell me a bit about that.

R: What can I say?

I2: No like earlier you said, you used to feel tired. You felt a fever from inside.

R: No it feels better than before.

I: Okay if I ask you, what things do you need to lead a good quality life? What would you like to say about this?

I2: What other good things do you want? What good things do you want? Like if I had this or that, I would feel better. Tell me about that. We all have things in our heart right.

R: We, folks, in our entire life earn money and eat even from childhood, my parents don’t educate me . earn and eat , life is going in that way and now I can’t do anything.

I2: You think in your mind no, that like if you had this, you’d feel better, hm?

I: So what else, what other things- like one thing you said was education- what other things hold importance to you in life?

R2: There are a lot of things in my heart just by thinking otherwise if you don’t think then there is nothing.

R: there are a lot of things in my heart just by thinking.

I: Yes tell me that! That’s what I am asking- what you think about.

R: No, there is nothing just by thinking about it. Now the time is like- I will work for someone, get money, go to bazaar and eat. If I don’t work, I will stay quietly hungry.

I: Hmm hmm hmm- so what other things are there that you need?

R: We do not have that much land. We have a house…[*trailing*]

I: Yes, and?

I2: Do you have a son?

R2: Yes, we have two sons.

I2: what they are doing ?

R2: they are…ummm…. doing some truth-lie work of house.

I2: of house ?

R2: yes

I2: do they help ? do they look after ?

R2: in the society sons are looking after and if they are in society they may look after or not

I2: like they know that he is having disease then

R2: yes , disease is there and even other is looking after so he will not do?

I2: like how are you feeling . do they help with money ?

R2: there is a small girl (inaudible) so for us how do they do ?

I2: where does he work ? in [redacted]

R2: he works in [redacted]..as a labourer

I2 : so, he couldn’t come ? may come after 6 months or 1 yrs

R2: no, he came today at our house

I2: so he ask about you or feel anxious about you

R2: yes

I: So right now who’s in the house? Just you two?

R2:one of the son is outside , daughter is getting married in few months and other son and three grandson

R: They uh- the elder boy, he stays separately.

I: So does his family stay here too?

R2: Yes, stay at home only.

I2: Grandchildren and all?

R2: Grandchildren are small.

I2: Do daughter-in- law look after you?

R2: (noise) overall she look after us. If the son is not good then no one’s daughter-in- law is good. if the son is good then she will be good also.

I: The treatment that is happening- what more do you want from it? What do you want which is not happening?

R2: What can we say?

I: You tell me.

I2: You think that there is no shortcoming in the treatment? It is happening the way it should? Do you think that way?

R: There is no shortcoming in the treatment. And…the way my health was bad before, I walk more now.

I: Now you’re able to walk more?

R: Yes! Earlier, I did not used to walk as much. It´s been one year since I rode a bicycle, but I have ridden it for two days or so and have not had any trouble. Rode only a little, did not go too far. I did not have any trouble.

I: You didn’t have any trouble? And other things? What other things have you noticed a difference in?

R: And if someone puts items or things on my head, my neck starts feeling unstable.

I: And how do you feel about that now? Is it still the same?

R: Yes-

I: Tell me what things you have noticed a difference in after treatment.

R: Now I am not feeling anything like that.

I: Not feeling anything? Like you just said, that you uh, meaning you are able to ride a bicycle now. And have you noticed any difference in other things?

[*shaking his head*, *looking unsure*] No? What else would you like? In what things would you like to see a difference?

R: Now I am not understanding what you are saying. Whatever I understood, I told you.

I2: Do you feel any weakness- do you not feel any weakness?

R2: He does feel weakness. one month he is under treatment , if people eat then they will have some strength. There is some problem related to money as sons are outside but now all are here and their sister is also getting married.People don’t get much strength just by eating rice and chapati

I2: So is the son helping out for the wedding?

R: A poor man always has some trouble with food and all.

I2: Yes, there is always some pressure. [*long pause*]

I: How were the people who were doing the treatment over there?

R: They were good.

I: How? Can you tell me a bit more about them?

R: In this hospital?

I: In [redacted] hospital. When you had come there for the treatment, how did the treatment go?

R: Very good.

I: What all did they used to tell you?

R: They told me about all these illnesses. When it came in the report, then they told us okay? They told us, and when I left there, the paper that I had gotten, the doctor said there is no paper in this. If there was a paper, you would have gotten money for Kala Azar treatment [from the Government]. I have not gotten anything yet, okay? So you have a date for the 15^th^ now. See if you get it by going there on the 15^th^ or not. This is what we talked about. And the paper was from Muzaffarpur. I went there. So they saw the paper, and started the medicine.

R2:they have given me a box having some medicine and again on 11^th^ I got another one

I: What other things do you want from life? [*pausing*] Meaning what do you not have? What do you have? What would you want going forward? [*pause*] What are your wishes?

R: Now it’s okay.

I: No, you can just tell me normally. Not about the illness necessarily. Generally. Meaning what do you want going forward? And what do you think- what should happen going forward?

R: A person won’t get anything just by thinking about it.

I: That I understand, but you can still tell me. What things…what things would you like? This is what we are trying to understand.

R: Until now, someone had gone to another place who got Kala Azar treatment. Hm? So he- how he got the treatment from the hospital in what way...I am not educated. So he- he has made a house. Got some money- made a house. He had a check in once. When he got the money, he went and made a house. So he had made a house- took a picture and went.

I: Meaning?

R: He took a photo of his house! The money that the government gives, hm? Not for eating and drinking. He had gotten a fund to make a house. the money that was given to him did he utilised to make house or to eat that’s why officer came for checking. But, he made a house.

I: And yours? Any change in your life?

R: What else can I say, Sir?

I: Related to the house? What else would you like going forward? Or regarding the family? Or the house?

R: Now, if I could get some money or something then…I don’t eat or drink that much. Nothing. Just that I will drink chai morning and evening, and eat food. Alcohol and all the other things- nothing.

I: Hmm hmm hmm. And what would you like for your children going forward?

R: For kids, what can I say… (pause) I made a small house by earning but my elder son have some problem and younger one left. Now younger one is living in hut and we are living in hut.

I: If you could change the house somehow, would you like to change it? What would you like to do with the house?

R: I will not be able to say anything. You have maybe been able to understand something [*chuckling*].

I2: Have you made the house?

I: When did you make your house?

R:The house- it’s been two years.

I: It’s been two years. 2 years…I see. You have a lot of neighbors nearby?

R: Yes. I have neighbors.

I: I see. And they talk to you?

R: Everyone talks to me.

I: Have you noticed any difference before or after illness about this?

R: No.

I: You haven’t noticed any difference?

R: No.

I: I see, I see. So you see them every day?

R: When I was well, everyone used to come, sit by the door, and talk and laugh with me. Now also, people come and sit down. They don’t have hatred for us Okay? One man said, so what if you have an illness, you’re a person. Everyone can get an illness. if I hatred someone after knowing him having disease then it may happen to me also

I: They said this?

R: Yes.

I: I see, they told you?

R: they don’t hate. Like before , if the four are sitting together then they will talk to each other.

I: So everyone talks with you together?

R: Yes!

I: And you haven’t noticed any change either? Anything?

R: In my area, the thing is- all people are poor, just working to eat.

I: [*pausing*] Hmm hmm hmm. Any other things that hold importance? Which you might want but don’t have right now?

R: [*coughing, long pause*]

[*silence*]

I: Would you like to say anything else about the treatment?

R: No whatever treatment happened- food and all was all provided well. [they talked in very nice way . However many staff there are- no one said anything rudely. Compared to [redacted] hospital, this place has better behaviour. You know [redacted] hospital right? Some doctor will leave at 2 pm, some at 3 pm, some at 4 pm. If a patient comes at 5 pm, the place will be empty. Here, you have someone 24 hours. I stayed here 1.5 months, right? One thing is- the Sir is going- but in his place, someone else is coming to do their duty. At one time, everyone is doing it. What is happening to someone or not happening to someone- there is no trouble.

I: Is this important to you? When seeking treatment? Do you see whether this is happening or not happening?

R: Yes.

I: I see. Are other places not like this?

R: No it does not happen like this.

I: How does it happen?

R: I told you right? There are 4 Sirs to look after me. If a patient comes at 2 pm there, some doctor will leave at 2 pm. Someone will leave at 3 pm. Someone will leave at 2:30 pm or 4 pm. If a patient comes at 5 pm, then the hospital is empty. “No, no, he will come tomorrow- Sir has left already.” Hm? Staff are there, right. They tell us like that: come tomorrow!

I: So you wish that when you come to seek treatment, someone should be there and see you on time?

R: Yes.

I: And this, I mean, what has happened now has happened just like that?

R: Yes the treatment was done properly. Food was also taken care of. I have no complaints.

I: I see. And when you were just called back today for follow up, did you face any trouble?

R: No no.

I: And you’ll have to come back again right, going forward?

R: Our date was 15^th^ April. But Sir did a big favour to us by calling us to say don’t come on 15^th^, it’s closed, come on the 16^th^ of April. Alright, okay. So I didn’t come yesterday, and came today instead.

I: Alright, then okay. Would you like to say anything else? About your living situation? Your life? Anything?

R: No.

R2: No Sir, now we have talked a lot.

I: Okay. Alright, thank you. Thank you very much.
