# Supplementary material for: Quality of life perceptions amongst patients co-infected with Visceral Leishmaniasis and HIV: A qualitative study from Bihar, India
Source: PLoS One. 2020 Feb 10;15(2):e0227911. doi: 10.1371/journal.pone.0227911 (PMC7010301; doi:10.1371/journal.pone.0227911)
Supplement: S3 File — (ZIP) [file pone.0227911.s003.zip › Transcripts/Patient 10 Male No Age.doc]

**Patient 10, VL-HIV, no age reported**

I2: Can you tell me a little about when you first came to get treatment here?

R: Here in [redacted]? [*pauses*] I think it’s been 6 months.

I2: It’s been 6 months.

I: So 6 months ago you came for treatment to [redacted]?

R: Yes I had come to [redacted].

I: I see. And is this your wife?

R: Mother.

I: Mother, I see. And where do you all stay?

R: Our house is in [redacted] district.

[*Redacted information on exact location*]

I: So what work do you do?

R: What work, madam? I am a poor man. I used to stay in [redacted]. I pull cart which carry goods

I: If you could just speak a little louder, since your voice is being recorded…

R: I used to pull a rickshaw which carry goods By doing this work, this poor man used to just…

I: So was there no work in [redacted]?

R: No, Sir, everyone was going- everyone used to stay from the village, so then I also went there.

I: Oh so your friends and all used to stay?

R: Yes, first in the village…[*mumbling*]

I: So you used to pull a cart?

R: Yes, Sir.

I: What used to be on the cart?

R:anything under transport like iron material, medicines. All kinds of goods come in transport. So that-

I: So you used to pull it or ride it?

R: Yes I used to pull it.

I: Not a truck?

R: No no no no. I used to pull it.

I: Meaning it required effort?

R: Yes, yes.

I: Ah, okay. So first of all, tell me- when did your problem- when were you completely fine first? Tell me everything properly.

R: I was completely fine 6 months ago.

I: No problem at all?

R: None- I had no problems on my end.

I: I see and 6 months ago you were there only, in [redacted]?

R: Yes. I got fever with chills there. So when I got the fever with chills, I went to the doctor and ate local medicine. And I didn’t get better. Didn’t get better. Then I came back.

I: This local place you went to- was it a doctor or a hospital?

R: Yes it was some doctor- who sits in villages. He was quack. So I got medicine from him. There some relief. I came back from there. I came back from there, then again I got fever with chills one day…and my body, means, became completely weak. Got a high fever, so I went to my brother and said, brother take me to some good doctor, so he took me to [redacted]

I: So when did you come back here from [redacted]?

R: 6 months ago.

I: So how many days did you stay there before coming back here?

R: I stayed for…a year over there.

I: For one year, you did medicines here and there?

R: No no. I did this one year.

I: When your health worsened, when you got the fever with chills

R: Yes meaning one month ago-

I: Then you stayed one month more there, and then came back.

R: Then I came back here. Yes. I had gone for a month over there in bad health, so I came here and I went with my brother to [redacted]. So in this big hospital, there is some [redacted].

I: Big hospital meaning a private hospital?

R: Yes. So there he did the investigation for Kala Azar.

I: Okay so why did you not go to the government setup anywhere?

R: I had gone. So we went to private right? Then he had written…Kala Azar. When he did the investigation, he said I have Kala Azar. You should go to [redacted] hospital. The government hospital. So I went there Madam ji, and they found Kala Azar, and they named one more illness- that I also have HIV. So you should go to [redacted].

I: So when they told you that you have both these illnesses, how did you feel? How did you feel? Had you ever heard about HIV?

R: No I had not heard about it.

I: You hadn’t even heard the name?

R: No, I did not know anything. So…they said go to [redacted]- the treatment for this is in [redacted]. So there is an [redacted] hospital. So I…came there, so for 8 weeks days, there were just investigations- took blood, took this, all this- so 1 month 10 days, I stayed there for treatment.

I: So when you found out about Kala Azar, what do you think about Kala Azar? What kind of disease is it? How did you feel finding out that you had Kala Azar?

R: Kala Azar was found after the investigation, right. Doctor said.

I: Were you not feeling worried after you heard this, that you have Kala Azar?

R: What worry, what will I feel- nothing for me.

I: Nothing- nothing hearing about Kala Azar or TB- uh, HIV?

R: I had no worry inside me. Did the treatment without any concern, and went. I don’t have a thing called worry.

I: So what do you think, meaning, when they told you that you had HIV and Kala Azar, did you tell your family members about this?

R: Yes I told them, Sir.

I: Right then?

R: I didn’t tell them right then. When I came to [redacted], I had to tell them.

I: Oh you told them when you came to [redacted]?

R: I had to tell them.

I: So who was with you then in [redacted]?

R: Mummy was with me.

I: So you told Mummy? And your wife?

R: Yes I told my wife at home.

I: And your kids?

R: I did not tell my kids.

I: You did not tell your kids. And anyone in the neighbourhood?

R: No.

I: Do you know of anyone else in the neighbourhood who may have a similar illness or who may be taking medicines?

R: [*pauses*] I don’t know, Ma’am.

I: Yes so you were saying- was there anyone in the neighbourhood?

R: No one in the neighbourhood.

I: No one? Who might be eating medicine for this illness?

R: Oh the medicine for this. Yes there is one…he…one died. He was eating medicine for this and died.

I: Anyone else?

R: If his children and all had it, they may be eating it.

I: I see, you don’t know for sure though.

R: Don’t know for sure.

I: So you must know- has your wife also been tested?

R: She has not been tested yet. I will have to do it now.

I: So when you went here in [redacted]? did they not call you and your wife to get a test?

R: They called. We didn’t go- didn’t get time. Now I shall go and get a test. I was involved in some farm work and cutting crops. So now I also have to get her tested.

I: I see. So what do you think- in order to live a good quality life, what all does a person need?

R: A good quality life…means, what do I think now…if the body becomes healthy, that is only a good life.

I: No what did you think before the illness- what all things did you want to get done?

R: Work, Madam ji, what needs to be done? I used to earn. I used to eat. I had kids, a girl. Have to get her married. I have to feed the kids. This is what I used to think- what else can I do?

I: Anything else? Building a house or buying any land?

R: no how do I buy house? Nothing…what land…[*mumbling*]

I: So means getting married, and kids education?

R: Yes education, that my kids should study, but the kids also did not study, Madam ji.

I: I see. So then…why…I mean…then tell me one thing. What do you think about your family? Or about your living environment…where you stay, is it a good atmosphere? In the village?

R: The atmosphere…is basically okay. Nothing bad, really.

I: Okay. And the house situation/outlook?

R: The house situation…okay, I mean, have to eat and earn…but I get this from the illness- feel broken.

I: Are you able to work after the illness?

R: I am not able to work.

I: What do you do now, then?

R: Now, I don’t do anything.

I: From morning to evening?

R: From morning to evening, there is one cow. Occasionally, I feed it grass, give it one bucket of water. I don’t do anything else. I would be able to do something, but I have one problem.

I: What?

R: Now I have started believing that I will not be able to do any kind of work. Earlier, there used to be a hope that I will do some work. Now, in this, when I walk…[*pointing to his legs*]…it catches. First it happens in this leg, now it feels like it’s happening to this one as well.

I: So because of this problem in your legs now, you are feeling like you won’t be able to work.

R: I am thinking I will not be able to work. If I get better, then I can do it.

I: I see. So how are you managing the household expenditures now?

R: House expenses…I have a son…he gives something.

I: Does he work?

R: Yes he work.

I: Where outside?

R: Yes, he gives something, so I can eat.

I: Who else stays at your home besides your mom?

R: We are 5 brothers.

I: So is everyone together?

R: No no. Everyone is separated.

I: So who is in *your* house?

R: In my house, I have my wife.

I: Whose eat all the food together [*clarifying*]

R. I have a wife, I have a kid or so.

I: How big?

R: He’s there…quite big. Something like 25 years old.

I: So he stays at home only?

R: Someone’s in the middle, someone is 16-18 years old.

I: How many sons do you have?

R: 3 sons.

I: All 3 sons work?

R: Yes all three are married.

I: So their family is not living separately?

R: Yes they are, but assume he gives a little from that.

I: Because after marriage it happens, no, that…

R: Yes it is separate. Suppose I have fallen, meaning become ill, so little bit…

I: [*interrupting*] How many people’s food is cooked at your stove? You, your mother, and?

R: And uh…this…

I: Your wife?

R: My wife.

I: And daughters?

R: Daughters have gone to their respective homes. When they come, they eat with us.

I: But you’ve married them off?

R: There is rule in village that when daughter came she stays fore 1-2 month at parent’s home. In towns otherwise, you see she comes today, is gone by the evening. Comes in the morning and goes in the evening.

I: So now do you think you will still be able to do the work you thought you would be able to do?

R: I will not be able to do it because of this illness- my leg. Otherwise, I can do the rest. I said I would do it. But from one month, right, because of this illness [*trailing off*]

I: So what do you think- what other things do you have to do?

R: If tomorrow I want to do, so what would I do- nothing happens by thinking about it. That I’ll do this, I’ll do that, but by doing it…it is God’s will, whatever will happen will happen. Nothing will happen just by thinking about it.

I: So will you be able to go back and pull a cart?

R: I will be able to, but I told you what I cannot do.

I: Because of the leg?

R: Yes. Weight now I can’t…when I walk I latches I feel like I fall down …I bend . So from this, it looks like I won’t be able to do it. If this improves, then I can do a little bit.

I: I see. And the treatment you are getting at RMRI- would you like to say something about that? How do the staff talk to you? How is their behaviour? Do they talk nicely to you?

R: Yes. They talk nicely, in a very good way.

I: The medicines you are getting, the treatment you are getting- are you happy with this?

R: Happy, well, I am, see there is one thing I didn’t tell you…this nail of it had gone dead. Not one nail was proper on this hand. Now, after eating this medicine, my nails have all come back. The other hands and legs were okay, but the nails on this hand were gone. This has become better.

I: So means you…

R: I feel good with the medicine.

I: It is working?

R: Yes. The medicine is giving me a benefit.

I: Any one thing you would like to improve over there? If this thing was like this, it would be even better? In [redacted] hospital where your treatment is happening or wherever you are getting your medicine from.

Now where are you getting your HIV medicine from?

R: [redacted].

I: So would you like to improve anything there?

R: Meaning…

I: Meaning anything which you may not like- that if something was like this, it would be better-

R: So if I say anything, it wouldn’t happen, no Madamji.

I: It will happen with what you say.

R: I want it to available in [redacted]

I: We need yours [opinion] only. Because when we write it and move it forward, it is possible there will be change- that’s why.

R: I want it to available in [redacted]

R: It takes money to go [to [redacted]].

I: It takes money?

R: Yes, if I go, I won’t go with a closed mouth [without eating anything]. Some chai, water, I will drink and go. So it takes money- 200 rupees to go and come. so I want it to available in [redacted].

I: You don’t get any kind of money?

R: Where do I get any money?

I: Conveyance fee for HIV?

R: I get conveyance here for coming and going.

I: You don’t get it there?

R: I don’t get it there- not one paisa.

I: Any form or anything they made you fill? For money?

R: There? I have not filled any form for money.

I: So you have not gotten any money from the government?

R: I have not gotten a single rupee. When I come here to [redacted], I come with another person, and I get reimbursed for the travel expenses that’s it. No other money. I have not gotten a single other rupee.

I: So, meaning, you are hoping that your medicines would be available in [redacted]-

R: [*interrupting*] So it is closer.

I: To save travel costs from coming and going or that you get it from here.

R: Yes, I would have gone by bicycle and brought it [medicines] from [redacted]. It would be close from there. It’s 6-7 km from there.

I: And now what all medicines- meaning, after illness, what kind of change do you notice in you life? [*pauses*] What you were 6 months prior, and your current situation.

R: I was very good [healthy] 6 months prior. When I fell sick, I felt down- the body remains weak. I can’t move my cart with my feet. Sometimes I get pain here, sometimes over there. Meaning, I stay sick- not any use for work. What should I do?

I: So means you feel less strength?

R: Feels less and I feel now that I will no longer be able to work. How will we survive? This I am definitely feeling inside my body- how will my wife eat? How will I eat? This I definitely feel.

I: Is there no solution in your village that you can get small jobs?

R: Small jobs well- I can’t push any carts- I can’t lift any weight on my head. In order to do something, sit and do something, I am uneducated. But I can do something. But there should be some money- a little bit. So I can do something and sit down.

I: Have you thought about anything like this?

R: I think about it, but nothing happens.

I: What do you think?

R: I think that I can set up a small shop. Set up a quarter and sit somewhere, then I will survive.

I: Meaning it shouldn’t be a job involving and a lot of strength.

R: Yes! Sitting-

I: [*interrupting*] Which you can sit and do.

R: I used to think this, but it doesn’t happen.

I: You think about this?

R: I think about it, I definitely think about it. One thing always stays in my mind- that I would have a small shop set up somewhere. can’t do some document work but when customer comes I would sell and get money , I can do this work

I: You can sit and work?

R: I can sit and work, but now I cannot do heavy work. I told you I used to pull the rickshaw. I could pull a load of 6 quintals or 7 quintals, tell me, but now my body…

I: You could pull 7 quintals?

R: Yes! I used to bring 6 or 7 quintals on the cart and pull it. The road there is good. It’s plain.

I: Meaning it’s not broken/potholed?

R: It’s not broken/potholed, and it’s not up and down.

I: It’s plain, so you don’t have to lift it up.

R: Yes, so I used to do it. That was my source. My wife and kids will eat if I do set up some shop or do some work. But now I can’t do it madam, how should I do it?

I: So what responsibilities do you have now? You have married your sons and daughters.

R: Yes.

I: At present, you don’t have such big responsibilities? Some small kid or anything like that.

R: One boy has yet to be married- there is still one responsibility. The small one is still a kid.

I: How old is he?

R: He is big…he is 16 years old now.

I: I see. So you think anything like this for him?

R: He works outside in some company or something. Earns a little bit.

I: He doesn’t stay with you?

R: No he’s outside too.

I: Where?

R: He stays in [redacted], in [redacted]. I used to also stay there in [redacted].

I: He had gone along with you?

R: He had gone along with us, but then he…

I: Got separated.

R: Got separated.

I: Does he come home?

R: He comes home. Comes home for one month in a year, in 10 months. He comes. He gives us a little of his [income]. All three of them give us some little amounts. It’s up to their own will. If they feel like it, they give, if they don’t, they don’t give. What can I say to anyone?

I: Would you like to say anything else here in this situation? [*pause*] Would you like to say anything else?

R: What else should I say, madam ji? I told you everything- if there was some help, then I could get some roti somehow [food]. What else can I say that is bigger than that? The biggest thing is getting roti [food]. Fixing the illness. Need food for the wife. If all this can happen, what else?

I: I see. This, means all three things you wish…

R: Yes. I think…but thoughts don’t come true…when I think, I think it would be great to have a proper house. It would be good, but now how will it happen? When I can’t earn myself, how will it happen? Who will give it to me? Will not give. Everyone has an interest in building a big house. Put in a nice table and chair, live in a good way, put a nice bed but now how will this happen? How does this happen for a daily labourer? And who gives it?

I: But even then, you had that thought, right?

R: I had that thought, but it wasn’t possible, it doesn’t happen, I got sick, so…what? Everyone thinks that I buy land for my son so that it would help him in future. This is what people think, that they can make a big flat or house. But it doesn’t happen- now what can I do? [*Taking long deep breaths*].

I: Would you like to say something, Mother?

R: What will mother say, she is uneducated. I am also uneducated.
