# Supplementary material for: Quality of life perceptions amongst patients co-infected with Visceral Leishmaniasis and HIV: A qualitative study from Bihar, India
Source: PLoS One. 2020 Feb 10;15(2):e0227911. doi: 10.1371/journal.pone.0227911 (PMC7010301; doi:10.1371/journal.pone.0227911)
Supplement: S3 File — (ZIP) [file pone.0227911.s003.zip › Transcripts/Patient 14 Male Age 43.doc]

**Patient 14, Age 43, Male**

I: Can you tell me a bit about yourself? Where are you from? How long, meaning how many days have you been here?

R: I am from [redacted] village in [redacted] district. I was admitted here on the 16^th^ …last month [March]. So here…first, I went to the private setup. In the private setup, they told me it was Kala Azar. When Kala Azar became positive, they told me you should go to your block. So I went to block, and in the block, the Kala Azar came back positive. And then after checking, HIV came out as well. Then, they told me to go to the district, so I went to [redacted] district. Then, there was a full investigation in [redacted]. After the investigation, they gave me the address for [redacted]…said go there, and made me talk to [redacted] ji. Through [redacted] ji, I got admitted here, Sir.

I: I see, I see. So when you first went to the private setup, was it a big hospital or a small corner shop type of place?

R: No, it wasn’t a big hospital, but he was a fine doctor.

I: The doctor was okay? What all did he tell you?

R: When I first went, then…first, I used to have gas in my stomach. After gas, I used to get pain on the left side here. So I suffered with this for approximately one month, kept eating medicine for gas. I used to get fever also as it came and went, and I used to feel chills also. At that time, I was in [redacted], Sir. So from there then, many days nothing would happen- I was not able to eat any food at all, and I used to get fever also and get angry also. So I left for home on the day of Holi. Then I went home, and as soon as I came to him, he checked my stomach. I told him my problem that I have gas in my stomach…meaning, I get fever also, I do not get hungry even a little, and I get a lot of anger. So he told me to first get an investigation. I got a blood test, an X-ray, an ultrasound, so then he said you have Kala Azar.

I: At that time itself, he told you that you had Kala Azar?

R: Yes Sir.

I: I see. This doctor told you. And then what did the doctor say?

R: He told me you get your workup done from the block. Go to the block. There is a big Kala Azar hospital there- I can’t do anything for you, no? Go to your block, there is a level there. When I went to my block, I got it checked and after checking there, I tested positive for Kala Azar. After that, when they checked, HIV also came out positive.

I: So when you say block, is this the district hospital or some block hospital? What is this?

R: Block’s, means, it is a hospital, it is a government hospital.

I: What is it called?

R: It is called [redacted].

I: [redacted]. Is this in [redacted]?

R: No this is in [redacted], and the district is [redacted]. From the block, from [redacted]block…it was a Saturday…so I did not go on Sunday. On Monday, I went, I went to [redacted].

I: Another hospital in [redacted]?

R: Yes, I went to [redacted] Hospital. After going to [redacted], for 6-7 days, all investigations were done.

I: So you were admitted there? Meaning you were staying there?

R: I was not admitted. Coming and going every day, coming and going every day.

I: Coming and going every day. What did you think about this? About coming and going every day?

R: I was thinking that a full investigation is being done about my illness in the process of coming and going every day. Where is Kala Azar, what is it, is it not there…if it happens to my satisfaction, then good. I don’t want to hurry it either. So they used to take blood, check- do this, do that, like this they used to tell me. I used to go every day, and come back in the evening. After that, they referred me here.

I: And at this point, they knew in Sadar Hospital that you had HIV also and Kala Azar also?

R: Yes, Sir.

I: They knew about both of them. You had found out about this in the block.

R: Yes, I had found out in the block.

I: They didn’t tell you in the private setup?

R: In the private setup, I had found out about Kala Azar.

I: I see, I see. Has anyone ever explained anything to you about this illness? In the block or the district?

R: No Sir.

I: What did they say? Did they say anything?

R: No Sir.

I: Meaning, what were you told? When you were told you had this, what did they say after that?

R: Who?

I: The people there, the staff at the hospital…who told you that you had Kala Azar and HIV?

R: The doctor who was there…the main doctor…he was the one who told me I had HIV also and Kala Azar also. Hm? Then, the report that they give, the report for HIV, I asked them how and where did this illness come from. Tell me something! So then, they told me- this is nothing- keep eating and drinking normally, stay happy like you were before, and keep eating your medicine.

I: I see, so did you get a proper answer to your question?

R: No, I did not get anything like that.

I: Okay. What do you think about this? About not getting an answer?

R: Regarding not getting an answer, Sir, I would like an answer- how I should go forward, how I should stay- you all will have to tell me.

I: Hmm hmm, but at that level, do you think that there should be a doctor who should tell you about this-

R: They give me proper advice- go like this, eat and drink like this, stay like this.

I: And do you think it is important to know the reason for this illness? That why this happened? What can be done about it? The doctor did not tell you about this?

R: No Sir.

I: What would you like to say about this?

R: Now when he didn’t say anything, he didn’t say anything at all…he just wrote down that I had HIV.

I: So do you wish that the doctor would tell you?

R: Yes, it is important to know, Sir.

I: I see. And then he told you that you should go to [redacted]?

R: Yes.

I: [redacted]. How did you come to [redacted]from there?

R: [redacted] from there….uh, he called with [redacted] ji over there. He also gave me [redacted] ji’s number and I talked to him. So he told me to come tomorrow. I said okay I will come tomorrow. He said you can come two days later too. I said I have an illness, and to stay with an illness like that is not good. I want to get rid of it quickly. So I will come tomorrow itself. So then there was some accident somewhere, 11 people had died in an accident, and there was a vehicle strike, which is why I wasn’t able to come. So I called him and said no I am coming tomorrow. So then on the 16^th^…I left…from there and I came here. Then there was a full investigation done here, whatever should be done. After it happened- it happened on the 16^th^- then I was admitted on the 16^th^, so my treatment started from the 25^th^.

I: Hmm hmm. And tell me about the time during treatment. How did the treatment go? What all did they do?

R: They took blood…took urine…took cough [sputum]…they took all this. Yes Sir.

I: And how was the behaviour of the staff towards you?

R: No Sir, the behaviour was good.

I: Hmm hmm hmm. Did you ever ask them any questions…about the illness?

R: I used to ask [redacted] Sir or some other Sir. They used to say don’t worry, it’s not something to get worried about. You will have to eat the medicine that is there. Until this illness is there- this illness will not get over- because of this, as long as you are alive, you will have to eat the medicine.

I: What do you think about this?

R: In order to stay alive…I have four kids, I am a poor person, I will have to eat it, Sir. Who will say anything against that?

I: Hmm, is there any effect of the medicine on your body? Good or bad- you can say both.

R: Until now, Sir, I have felt no effects…I haven’t been on them for that long yet.

I: Hmm hmm hmm, how many days has it been?

R: Approximately…5…and…it has been running since 8^th^ April Sir.

I: So are there any harmful effects of the medicine on your body?

R: No, Sir.

I: Stomach and all is okay?

R: Yes. Since I’ve been here, I have seen people…they get vomiting too, they get diarrhea too, they get fever also. Nothing like that has happened to me.

I: Nothing has happened?

R: Nothing has happened.

I: Okay, that’s very good. Um, when you first found about your illness, what was going on in your body, in your mind? Can you tell me a bit about this?

R: Yes Sir, in my head, I was thinking how did this illness- now that the illness has happened, what can I…do about it? It is not possible for us. It’s not even a small illness. It’s a big illness. And before that, it was that only- not feeling hungry, gas in the stomach, getting fever, vomiting sometimes also- it used to happen like that. If I was vomiting, it would happen in the morning, and…I was feeling nauseous that I will vomit anytime, now I will vomit. I used to get feelings like that, Sir.

I: And in your mind, meaning, what did you think about the life ahead of you?

R: About my life ahead, I was just thinking that now that I came here, I was thinking what will happen ahead? I have to eat medicine. For the body, it is such an illness that it will make me weaker day by day, so I need good food. Now how this will happen, what will happen, this is up to God. This is what I was thinking. Who should I rely on for support? Mummy is not there, Papa is not there- they have all expired. We are landless. So by thinking this, it is just God’s grace. Kids…

I: How many kids do you have?

R: I have 4 kids. 1 boy and 3 girls.

I: Are they all studying?

R: 1-2 of them left it. The big one came with me over here. He left his studies. The other three are girls, they are all studying.

I: So did he leave it because of your illness?

R: My son? No. Because of poverty- it becomes hard to run a family- I have four kids. Hm? This is the thing. Mummy and Papa have expired- till they were there, we used to manage. Now they aren’t there, farming is not there, I work outside- how much can you earn outside?

I: What work do you do?

R: 6 years ago, I used to do welding work in [redacted]. Welding- in fabrication. So when I came home from there, after coming home, Mummy and Papa expired. Within 1 month, within 1 month 23 days. Whenever we had money, I spent it on them. I had to sell 1760 square feet of land because of my father. After selling it, I brought them here to [redacted]. They expired here…in [redacted]. After them, I was left without any support. And who is related to whom- no one cares about anyone else. My younger brother left everything…right then. Then I didn’t think it was appropriate to leave for outside, because it’s my home. Where will I go after taking 4 kids with me? Even after going outside, there is more expenditure outside than here. I used to work as a daily labourer. For the past two years, I was in [redacted]- so in [redacted], there is this sweet sign- it’s sweets- they make sweets- it’s a company- so I am working there for 2 years.

I: And what effect did the illness have on your work…if any?

R: On work…the same…no desire to work, my body used to feel very lazy.

I: Anything else?

R: No nothing else like that. I was experiencing a lack of interest in work, hm? I wasn’t feeling any interest in work- if I worked a lot, then I used to get pain here on the left side of my chest. All this…

I: I see. And what was going on in your mind at the time? Any problems related to your family or- ?

R: The problem was that I am all alone. If something happens to me and I sit at home, then it affects our ability to earn every day, or eat every day. Now if something happens to me, what will happen to my kids and family? How will they eat and drink? How will they live? I don’t have anything…I don’t even have a home. It is a thatched roof hut. So the thing is…I used to get some government rations…they’ve come here now and they are saying construct a toilet, then okay, otherwise that will shut down too. Here I am suffering from an illness- who is going to build a toilet there? That is also shutting down, the ration is also shutting down for BPL.

I: The ration was stopped?

R: Yes, Sir.

I: And if I were to ask you what factors are required to lead a good quality life, then what would you say? What all comes into a good quality life?

R: In order to lead a good quality life, my job…there will be…nothing much happening. Meaning as I am ill, and I have to come here to get medicine every month, Sir. I cannot go out anywhere…to work.

I: Where did you used to go outside earlier?

R: Outside…I told you right, I was in [redacted]. And after that, I was in [redacted]. From [redacted], I came here to my house on the day of Holi. I was sick there for a whole month. I got a check up done, so they thought it was a fever, gas, etc. I threw 7000-8000 rupees at that. Then, I thought day by day, my body was getting worse. Then I left there to come home, so I came home during Holi. After coming home, I waited on the day of Holi, and after Holi, I went to a private hospital in [redacted]district two days later. So I came there and he gave me 5 days of medicine. He was saying get an investigation done. I got a blood test, and an X-ray, and I took an ultrasound. Did the ultrasound and x-ray and after doing that…it came out positive in the blood. That you have Kala Azar. Then they told me to go to [redacted]- it’ll all happen over there.

I: Hmm hmm hmm- so what effect do you think this illness will have on your work? One thing you mentioned was that you will not be able to go out. Any other things?

R: No, one thing is I won’t be able to go out. What can I do in the home? You also know what kind of daily labor is available in the village- there today, not there tomorrow, there for 2 days, not there for 4 days. I have to eat the medicine for my whole life.

I: So how will you run your family?

R: So I will not be able to stay outside and do it. Now here, if I start some business, it will require money which I don’t have [*long pause*]. Business, like if I do some business, so I know the work of fabrication, Sir.

I: How much money do you need for that- to start it?

R: To start it, I would need 1-1.25 lakhs to start it. I’ll have to buy a generator, a welding machine, hammer, drills, everything I mean…the items for it, I will have to purchase everything. So that is mine…so I know this [line of business]. I know everything. Whatever railings are put on, whether it was steel, etc, I can do all of it. There is a mall in [redacted], [redacted]- whatever railings are put up there are done by me. Like this, the panels that are made in Jackson company- so only the panel’s body is made.

I: I see. And during this time, how was your family life? Does anyone in your family know about this illness?

R: Regarding the family, only my family knows about it.

I: Meaning?

R: Like my wife. And one of my relatives, my brother-in-law, he knows. No one else knows…[*trailing off*]

I: Do they know what happens with this illness? What this illness for…

R: No, they do know about this.

I: I see. Was your wife also tested?

R: We had done the test in [redacted], but they did not give the report to us the day we went to collect our medicine. They didn’t give us our report for two days, saying go and come tomorrow. And uh she also had a little…due to having too much fluid in the body, she used to feel breathless from climbing too much or walking too much, so she…ran around here- with me. In [redacted]- no one else was with me. Both husband and wife used to go, come back, and in this running around, her health also got worse. She also had difficulty walking. So we stayed here for 5-6 days. So a person who normally stays outside then stayed here in the AC, drank that water, hm? Drank the water from the fridge. So then it had gotten more serious, so she was sent home.

I: But then, they never gave the result?

R: No, she neither went to get the result, nor did they give her the result.

I: And then?

R: And then she went to this place, so they said it’s been several days, we do not know.

I: And then when did you find out- so is your wife positive now or not?

R: No, now after checking- only after checking again will we find out.

I: So, when you came to [redacted], you didn’t get her checked there either?

R: I had told him- I had told [redacted] ji once. He said go for sure, we will get the treatment done.

I: I see, so he didn’t get it done during the 30 days which you spent there for treatment?

R: No, Sir.

I: I see, I see. Did they ever mention this to you? Whether you should do it or not do it?

R: Yes, I mentioned it also, he mentioned it also. So he said no it is required for her to do it- since the two of you stay together, it is required.

I: I see. Did they give you information about how or why this happens?

R: This uh…[redacted] ji had said something. This AIDS…this illness which is HIV…happens because of any extramarital affair, or it happens by blood…from someone’s blood. Then Doctor Sir, when it was my first day, he started asking me. When he saw all my papers and did the recording- when I was hurt, what happened, did not happen- whether any accidents happen- all this he asked.

I: After discovering this, the folks in your neighbourhood, has their behaviour towards you changed even a little?

R: No, Sir.

I: Everyone still meets together?

R: Sir, the thing is…there was no chance. Where did I get the chance to talk to them? When I found out there, I came and ran here [to [redacted]]. Nothing like that has happened now. Even in the village, with my uncle and all, nothing of this sort.

I: And with your wife- how is your wife’s behaviour towards you, after finding out?

R: Wife’s behaviour is also okay. She also just used to ask how did it happen, what happened- her behaviour is not wrong either, Sir, now what can I say?

I: So did you explain to her? Meaning did you talk about this?

R: Yes, I explained. Then she understood it’s not like that. Until you are on medicine, you will have to eat the medicine. And even I have been tested, and I will also eat medicine as long as it’s possible.

I: If you think, at this moment, do you have any trouble in going here to there?

R: No- walking, strolling…I have no problem.

I: Earlier you were speaking about pain- how about pain?

R: No, I don’t have any.

I: I see. And during illness, what all used to happen to you? Pain and- ? Tiredness?

R: During illness- when the illness first started, I used to feel bloated with gas, get fevers, and chest pain right here- all this used to happen. I used to also feel tired and sleepy. I did not have an appetite. I used to feel like vomiting. And if I ate something, then I used to vomit. Yes Sir, it used to be like that.

I: And now?

R: Now it’s nothing like that.

I: And if you need to go here and there? You’re able to?

R: Yes, it’s not happened till now, like I used to roam around two times a day, morning and evening, when I was in the hospital. I used to take a round over there. I used to roam for 30 minutes, so I didn’t feel anything like that, just that some weakness…

I: You’re still feeling it?

R: Yes. When I climb stairs, I feel weakness, I feel breathless.

I: I see. And do you feel like this will affect your work in any way? What you were saying earlier- on going out or on work?

R: On work…the effect…it’s not a particularly work-intensive job that it will affect it…but in the future…what can be said.

I: Okay, welding and all you were saying-

R: Yes, if it is welding, etc., then I’ll have to put in effort…what effect, I have to do welding, there will be no effect, if good food is there, then…everything will be okay.

I: When your illness first occurred, what had you thought about your life in the future? What would happen moving forward? What were you thinking about?

R: Inside, I was…what will happen to me? No one ahead, no one behind, I am landless- what can I do? I was just thinking that my kids will be orphaned. There’s not even a house to live on, there is nothing anywhere- if something happens to me, then what will happen to the kids? Like this…

I: How is the house situation?

R: It is pitiful. If I’ll earn, I will eat, otherwise nothing.

I: But how is your house built? I mean

R: The house, just like that- meaning it is built of hay.

I: It’s yours?

R: No it’s mine, but it’s made of straw, in a broken state. Not made of bricks.

I: So would you like to build a proper brick and cement house?

R: Yes, why not? Who doesn’t want that? That I can eat well, live well- my family sometimes eats salt and roti, sometimes I also get dal-roti, sometimes that is also not there, Sir.

I: So do you think it is possible for you to build a brick and cement house after the illness? Is it possible?

R: Effort …Sir, I will have to also put in effort into this, it won’t happen just like that. This is why I’m thinking that…welding work is done from my own hand…I can do it by sitting at home and eat my medicine as well.

I: I see, so it’s possible?

R: Yes, it’s possible. Like…my son is also ready, so he´ll lend a hand sometimes. Hm? So I can live from that.

I: That’s a good thing. And regarding the behaviour of others, are you happy or unhappy with it? To what extent? The way others talk to you, both family and those outside your family- during the illness- how satisfied are you with this?

R: I am satisfied. There’s nothing like that.

I: You’ve not noticed any change in it?

R: I have not noticed any change. Everything is going well.

I: Okay. How satisfied are you with your living environment? Your current situation.

R: [*pauses*] There…uh, outside? Or at the home?

I: At the home, at the home.

R: At home, what satisfaction? Unemployment is there. There’s no employment- even if it’s there, there is no capital money [to start a business]. My own- I need money for anything I do on my own. If I sit here for one day- I used to get 9000 rupees in [redacted]- in 9000, I’d have to pay rent, I have to send [money] home for kids. And the little I used to get from government is also going to be stopped. And when that also stops, there will be problem after problem, Sir. Now, if I don’t earn, then the only thing that will happen is I will have to die hungry. Even in this period, I’ll have to do something, otherwise my 4 children will wander around…or die hungry. My kids’ lives will be ruined, everyone’s lives will be ruined.

I: What does he do now?

R: No, now he doesn’t know anything- only after 18 will he find work in some company or something.

I: How old is he right now?

R: He is 15 years old right now, Sir.

I: 15 years old, I see. What are your thoughts related to health? About your own health?

R: Health Sir, this is in our hands. If we get good quality food, then health will also be good.

I: I see. What about during your illness?

R: During illness…I used to eat a little bit of food…eat little by little by little since I did not have much of an appetite. However I am, I’m okay sir. I’m eating food properly, etc.

I: When you found out about Kala Azar and HIV…first you found out about Kala Azar and then HIV, right? [*patient nods*]. Regarding this uh, which illness were you more afraid of?

R: I am scared of both things, Sir.

I: I see. Tell me a little bit about this- tell me about the fear.

R: About the fear, Sir…at the very beginning, 20 years ago, my brother had also gotten Kala Azar. My father also had it. So they had all stayed for 6 months or so in the hospital. The injection used to come from Nepal [?], right? So many days- too many days I have been through poverty and my family has passed through periods of illness. When I was 5 years old, I was afflicted with paralysis. But none of my organs were deformed, by God’s grace. So we have struggled through illness. It was a serious condition, my brother…my father also…swelling in the stomach, poor appetite, meaning eating simple food. So first Kala Azar happened…and we became troubled by Kala Azar. Then, later HIV happened. When HIV happened and we heard about HIV, then my willpower reduced even more- first Kala Azar, then HIV. After HIV, I had a little knowledge that this is a very dangerous illness.

I: What things? What had you heard about this?

R: There is no treatment for it, meaning it cannot be finished. I will have to keep eating medicine.

I: Now, Kala Azar is…finished?

R: Yes, Sir.

I: Would you like to say anything more?

R: What would I like to say Sir? [*laughing*]

I: Anything…about this, about the illness…about the treatment.

R: However the treatment happened with the doctor, it was good.

I: Anything that was lacking in the treatment? Or anything else that could have been done?

R: No Sir, nothing like that, I never felt anything like this.

I: Okay tell me one thing- when you first started having a problem, and when you went to get it checked, how much time elapsed between this?

R: When…a month had elapsed, Sir.

I: One month had elapsed?

R: Yes. When my illness started, I was in [redacted]. So…gas and bloating started. Gas in my stomach. So I used to go to the medical store just like that- so I took medicine from the medical store for 2 days…I took Eno, Digil, ate it…nothing happened. Then I thought yes it’s a hot place and I eat hot foods…it’s possible my stomach became too hot. So I would drink coconut water in the evening or drink anything…I would make the yogurt-based lassi with salt, thinking it will cool my stomach. This will make the gas go away. But nothing like that happened. I spent a month like this. I also showed a doctor there. Then also, the doctor there…fever used to come anyway.

I: All this is happening in [redacted]?

R: Yes this was in [redacted].

I: And this doctor is a private doctor?

R: Yes the private [doctor] used to give me an injection for fever…used to give me a needle. Until the amount of medicine was in my body, it was okay, otherwise the fever would come back. Hm? When fever used to come back again, then again…I used to not feel hungry. Not at all…I did not find food appetizing. If I ate, I eat one roti maybe, otherwise I wouldn’t eat. For 2-3 days straight, I would not eat. I would do anything and maybe make a lassi and drink it 2-3 times. So it happened like that, and even then, I would sometimes vomit. Then as things progressed, my body became weaker and I felt more tired/lazy. Everything happened. I used to feel dizzy also. Leaving my bed…I used to feel like if I could just stay put somewhere all alone then it would be better. Then, I thought it wouldn’t get better from here.

I: Stay put somewhere all alone meaning?

R: Alone! No one should talk to me, I used to not like talking to people either.

I: I see, you were feeling like this.

R: I was feeling like this.

I: During the illness? Now, how is it?

R: Now, it’s good Sir.

I: Now you think you can interact with other people?

R: Yes, I interact with everyone, talk to everyone.

I: Yes yes, and what else were you saying, sorry?

R: So…it happened like this. So it was like this- that I would go home, so after going home, my earning will stop. If my earning stops, then my house, my kids and earning, eating [*stuttering incoherently*].

I: Is this why you didn’t go for treatment? Meaning why it took so long?

R: I spent all this time over there. Because of house related problems.

I: Meaning income related?

R: Related to income…here, there is no balance. However much I earn, I spent more than that [for treatment]. I would go 1000-2000 rupees in debt every month, and then I have to sell something to repay that amount. Because of this, I was not coming back. I spent a whole month like this. When things were nearing the end, then I thought no I will die like this over here. Better than this, whatever happens can happen at home. I would stay with everyone at least. So then, on the 28^th^, I left from there.

I: I see. So when you first felt bad, meaning when you first felt pain, how many days elapsed before you went and saw a private doctor?

R: Sir, 1 month and 5 day had already elapsed.

I: Without showing a doctor?

R: The doctor, well, when it happened…after 3 days, I showed a doctor.

I: You went 3 days later?

R: 3 days later.

I: I see. But the treatment…had the doctor already said you need to go to the block? Or where did you need to go?

R: This is after I came home, Sir. In [redacted]…in [redacted], every doctor was saying it’s just gas. You are feeling feverish. You may get hungry…[*incoherent*]

I: So they had not done any tests?

R: They didn’t do anything.

I: Then? How did you know that you should come home and seek treatment itself?

R: As my body starting getting more ill, I started feeling loss of interest towards everything. I don’t eat food here at all. I vomit, I can’t digest anything, I feel lonely and feel like I should be alone. And I definitely have fever. I don’t feel hungry at all, and if anyone says anything to me, I felt like killing him or insulting him. I used to feel bad. I used to feel alone…each passing day, my body became thinner and thinner. Then I thought, no it’s best to go home, whatever happens here can happen at home. After coming home- I came on the 28^th^- after the 28^th^, I left there on the 28^th^ and reached home on the 2^nd^. I came home on the day of Holi at 8 from there. After come, I showered and everything. I did not feel like eating, but still I said to the kids- you celebrate happily- it’s Holi- why should I cause them any sadness? Told them we are fine. And then, after the 3^rd^, I went to the doctor on the 4^th^. To the private doctor.

I: And then from there, it all started.

R: Yes [*mumbling incoherently*]

I: So after the illness, have you felt anything else about life ahead? Have you noticed any changes about what you used to think about life ahead or any dreams you used to have? Either during the illness or after the illness? Whatever you thought about your dreams moving forward…getting fulfilled.

R: The thing is I am completely okay from the inside now. So I just wish that I should be able to work as well. Compared to before, I am okay now! I can work, I can talk also, I can walk also. I am definitely feeling this way.

I: Hmm okay. And what things are necessary during treatment, according to you? [*pause*] Meaning, during treatment, one thing is the medicine should work. You mentioned that.

R: Yes Sir.

I: Other than this, are there other things that you think are important…for good treatment?

R: For a good treatment, medicine is most important. After that, it’s good quality food.

I: Good quality food- meaning? Tell me a bit more.

R: Meaning if I earn well, I will eat well. If I eat well, whether it’s fruits or anything, if I eat well, then my body will feel good, whether it’s making my blood. By eating salt and roti or by remaining hungry, I’ll not get cured.

I: So you think that hospital should give food like this?

R: Yes, I definitely think that, Sir.

I: I see. Do you get this anywhere else?

R: [*laughing*] I have never had anything like this anywhere till now.

I: Okay, alright then, thank you very much!
