# Supplementary material for: Quality of life perceptions amongst patients co-infected with Visceral Leishmaniasis and HIV: A qualitative study from Bihar, India
Source: PLoS One. 2020 Feb 10;15(2):e0227911. doi: 10.1371/journal.pone.0227911 (PMC7010301; doi:10.1371/journal.pone.0227911)
Supplement: S3 File — (ZIP) [file pone.0227911.s003.zip › Transcripts/Patient 4 Female Age 48.doc]

**Patient 4**

Age 48, Female

I: So tell me about yourself. Where are you from, like [redacted], meaning where have you come from? And how long have you been there?

R: In [redacted]? It’s been 10 years.

I: 10 years. And before 10 years?

R: We used to stay in [redacted] district.

I: You used to stay in [redacted] district? So why did you go from [redacted] to [redacted]?

R: Mummy has a house there.

I: I see. Mummy’s house, meaning mummy’s mother’s house?

R: Yes. And father’s house is in [redacted]. In [redacted] district.

I: So, you- 10 years ago- what did you do? Went from [redacted] to [redacted]?

R: Yes.

I: The reason for going?

R: We could not stay altogether over there. That’s why we went.

I: That’s why you went, I see. So who else is in your house?

R: Papa, mummy, elder brother, younger sister, and I. So 3 siblings.

I: So when you went back to [redacted], who all stays in the house in [redacted] right now? One is mummy, one is you, and?

R: Papa, elder brother, and little sister.

I: Oh, so everyone is in [redacted] too? Er, papa is also in [redacted], and your brother and younger sister as well. Everyone went back to [redacted]?

R: Yes, yes.

I: And your grandparents?

R: She is in [redacted] district.

I: She’s in [redacted]. So now, in the one house you are staying, the place you just named, there is only- [*recording paused as phone rings*] Yes so you were saying-

R: In [redacted], we stay here only.

I: And what work does your mother do?

R: Mummy doesn’t do anything. She just stays at home.

I: And you?

R: I study.

I: In which year?

R: I just took my 10^th^ exam.

I: Okay. Okay tell me- for your mother- can you please ask her how this past year went for her? What all happened in the previous year?

R: [*clarifying the question* *and asks in local language*] Meaning in the previous year, she used to feel weak, “halka halka”- not that much.

I: Not that much? Can you tell me about the first time- you know about her two illnesses, yes or no? She has two illnesses- HIV and Kala Azar. Can you ask her when she first found out about her illness?

R: She doesn’t know. We only found out. She did not know. That’s why we had gone to show in the private setup, they were saying she has a seasonal fever. That’s it. After that, she needed blood. So, my blood is O positive and my brother’s is A and we didn’t found blood . the doctor said girls can’t give blood and she became very weak and she needed a lot of blood. That’s it. We bought blood and gave it to her.

I: You bought and gave it?

R: Yes.

I: When did this happen? When did this take place?

R: Two months ago.

I: Two months ago, I see. And after that, when did you find out that you have an illness called Kala Azar?

R: When I came to the government hospital in [redacted], we got a blood test done for her [patient], because we had also taken her to private, but they only said she had seasonal fever. When we went, her face had become a bit black. People in the village were saying she has Kala Azar probably…what you call “pilhi” right in our local village. And in the village, we give herbal medicine right? By giving her that over and over again, her body became weaker again. Then in the end, they said you take her to the government hospital. You get treatment for free over there. We took over there, so they diagnosed her with Kala Azar. She got admitted. When they said HIV, we don’t know what HIV means. They just told us that you have to go to [redacted]. So we said we will not go to [redacted], we went away from the hospital. We asked for the report, we didn’t get it, and we went away. Then, again we went to the private setup in [redacted]. They said we do not have a treatment for this- that’s how they responded to us. Then from [redacted] or from [redacted], a Sir called on my mobile and said they won’t take anything- you go. So how will we go? Where we haven’t seen, how will we go? So then they went to our home, talked to us in our home. Even then, we weren’t believing them. But you know the Mukhiya of the village? They talked to him and lifted all responsibility from him, then we came here.

I: Then you came, okay. Okay the first time your mom felt this weakness/tiredness- when did that happen? This tiredness related to illness, right? When did you first feel it? How many months ago?

R: When…uh, 6 months, 1- meaning 7 months ago.

I: 7 months ago. And how much time elapsed before you went to the private setup?

R: Within one month, we went to private and to the government setup. Within one month only.

I: Went to both within one month? [Yes]. What was the biggest reason or problem for which you went?

R: She used to get a fever, and she used to feel tiredness/weakness and was not eating.

I: I see, so she had a fever, and was feeling tiredness, and she also had a poor appetite?

R: Yes.

I: Did all 3 of these symptoms come together?

R: Yes, she used to feel hungry, but she could not consume any food when she went to eat.

I: So she was hungry but she was not able to eat?

R: Yes, yes.

I: What did she think when she ate?

R: Meaning she used to feel strange. She used to think that she would eat it, but it would not go inside. Was feeling like vomiting. And she would trouble us all at night, she used to get a high fever at night, a strange type of-

I: How much- did you ever check how high was the fever?

R: We did not get it tested.

I: With a thermometer ever or –

R: Did not do. Because there is no thermometer in the house, no, and the doctor does not come to the house in the night. In the daytime, her fever used to come down- used to come in the nighttime.

I: Fever used to only come in the night time?

R: Yes.

I: What was the biggest reason for your concern?

R: [*without hesitation*] Due to the fever.

I: From the fever. What about the other two things you mentioned?

R: No problem from that.

I: Not as much of a problem from that.

R: No.

I: And after that?

R: You know the period? Earlier, she used to get it twice in a month. Meaning, she used to get it twice in one month.

I: And this has happened 6 months ago?

R: Yes.

I: Did she also feel a lot of pain in her stomach once in a month?

R: [*checking with her mother*] There was not a lot of blood loss.

I: How many pads did she use?

R: One extra day.

I: Used to be one extra day. Which day?

R: The middle day.

I: How many days did it stay?

R: Three days.

I: Three days. So the middle day, meaning the second day. I see. And it used to stop after three days. And in one month, you said how many times?

R: Two times.

R2: Not two, one.

I: Oh only once in a month? Once a month is normal. Everyone gets it once per month.

R: Meaning she used to have more pain.

I: I see. She used to have stomach pain. You didn’t have this pain before?

R: No.

I: Used to happen afterward? [*nodding*] I see. Any other problem besides this?

R: Nothing else.

I: And then where did you go from there?

R: After that, we came to a private setup in [redacted]. We got a check-up and we threw all our money at it, but we could not find the cause of her illness. Just said seasonal illness…

I: How much money had you thrown at this?

R: Meaning in one day a lot- Rs. 50,000 was over. In the end, we went to the government setup.

I: Okay, so 50,000 was finished in how many days?

R: In one day, we spent 5000 rupees.

I: 5000 rupees in one day?

I2: Why did you- why did you go to private first? Why not go to the government setup first?

R: We didn’t know, Sir. Because we thought it would not happen as quickly in government setup. It would be faster in private, that’s why.

I2: But at this time, you did not know which illness you had?

R: Yes.

I2: Okay so you first you went to private, then government setup?

R: Someone told us that you get free treatment for Kala Azar in the government setup. That’s why we went there in the end. Someone told-

I: [*interrupting*] So you – oh sorry please finish.

R: Someone at our house- you know there’s Asha- she was from our [redacted] district- everyone told us to go, but no one came to front, but the ASHA took us in the end.

I: Okay she took you with her. What did they tell you in the private sector regarding what illness she has?

R: Just seasonal fever.

I: Seasonal fever? Nothing else?

R: No. Just that, tiredness, and shortage of blood.

I: Okay, so seasonal illness, tiredness, and shortage of blood. Nothing else?

R: No.

I2: Do you know what kind of investigation they did? What tests did they do in the private setup?

R: I don’t know.

I2: They didn’t tell you?

R: No.

I2: But had they asked you to get it from somewhere outside? Get this done, get that done? Were you told this?

R: Yes.

I2: I see. But they didn’t tell you what?

R: No.

I2: I see. Then what happened? Where did you go next?

R: Then to [redacted]. Then there was a complete investigation done there.

I: This is [redacted]? That is a very big hospital there.

R: Yes. We went there, and after a full investigation there- they did like that in private setup, and government setup too. They didn’t detect that I have Kala Azar in private setup. They detected it in government setup. Then, they told us that there is an illness known as HIV. So we didn’t know what HIV was! So I used to ask everyone what is HIV?

I: Everyone meaning who did you ask?

R: Meaning when I went to my village, so my brother- brother had not gone- I was the one who went to government hospital, so I told my brother it’s like this- there is an illness called HIV and we have to go to [redacted]. Brother was already tensed that we have to go here and there, private here, there- tired of throwing away money, no? So he didn’t tell us anything. Private doctor does not tell us the truth, because he used to hide the truth from us. Only I used to go, right? Papa used to be at home, didn’t take little sister outside anywhere. Just my brother and I used to go to the doctor…somewhere else. So afterward, brother has some friends- they only said to the private doctor that he has a friend who has an illness called HIV. If you don’t hurry, it will only be harder for you. When we found out later, we- [*trailing off*] The staff folks had gone to our house from [redacted]. There is a Sir from [redacted] who had called on my mobile and said that we have to go, but I did not believe anyone. So he told the Mukhiya of the village- he explained to us and then about that…

I2: Why did you not believe?

R: Because people say, that government is like this and that, they say a lot-

I2: You had heard like that?

R: Yes. That’s why I didn’t go.

I2 : I see. And from there how did you come to [redacted]? You know [redacted], where you just were- in [redacted] where you just came.

R: There is a Sir in [redacted]. He brought us.

I: The one who had called you?

R: Yes.

I: Where is this Sir?

R: [redacted], you know him right?

I2: Yes yes of course. [redacted]- so he had brought you?

R: Yes. That Sir from [redacted]called [redacted] Sir and he brought us here.

I2: And at this point, you knew you had both HIV and Kala Azar?

R: Yes.

I: So what had they told you first? Kala Azar?

R: Yes, only Kala Azar. I knew the name HIV, but I did not know what it was.

I: So when they told you what you had, did they tell you both illnesses together or…what did they say first and what did they say after?

R: They told me both together.

I: Both together. And after that? When you found out, what did your mother think about it, what was her reaction?

R: She used to say I don’t want to go anywhere. So the Mukhiya explained to her as well, that you have children, who will look after them, so she said okay I will go wherever you take me.

I2: So, everyone counselled her?

R: Yes.

I2: I see.

I: Why did you not want to go anywhere?

R: Because we went to [redacted] too, [redacted] too, and also went to [redacted] - had gone there as well. They didn’t give us treatment there either. That is why she got angry.

I: Oh, she got angry?

R: Yes. They said go to your own district. Yeah they said you’ll have this opportunity, that opportunity over there, but she needed the treatment not the other things.

I2: So they sent you away without looking at you?

R: Yes.

I2: Said to go to your own district?

R: Yes.

I: So she didn’t want to go because of her anger?

R: Yes.

I: I see. And did she know about her illness by the way?

R: She didn’t know that much. Only we knew about it.

I: You didn’t tell her?

R: we told her afterward, two days before coming here, we told her.

I: You told her two days before coming to [redacted]?

R: Yes.

I: You didn’t tell her what the illness was, what it does?

R: No.

I: And when you said everyone got together to explain to you, what did everyone explain to you? Like Mukhiya and the others you mentioned? You told us about your brother’s friends. Did they do something too?

R: They told her if you do not get treatment, it is the kind of illness that spreads, it will spread. If you don’t get treated, it will also be a problem for your family. So she also thought, if it’s like that, I will go.

I2: Did people say anything else?

R: No.

I2: Did they say anything about the illness? What happens in HIV or…

R: Where? Yes, here they told us, how HIV-

I2: [*interrupting*] But do the people around you in your neighbourhood know about HIV?

R: No they don’t know. No, they just know that she has something called HIV. But they don’t know how it comes, what illness it is, they don’t know.

I: They know the word, but what it is, how it happens, they don’t know?

R: No no.

I2: Do you have an idea whether anyone else has a similar illness in the village?

R: Illness like a lot of people- in government, even if it’s fever they take medicine and eat it, but they think- slowly, slowly- now they don’t know, but if a lot happens, with Kala Azar the treatment happened-

I: No Kala Azar is okay, but like the villagers got together to explain HIV to her, is there anyone else in the village whom people explained things to in a similar way? Friends, relatives, neighbors- have you heard of anyone who was convinced to go?

R: No.

I: No one like that?

R: No.

I: Do you know anyone else who faces anything similar?

R: No.

I: So then, how was the last year for you? You were saying you were tired, no…meaning, did she work before the illness too?

R: She used to do housework, and when it was time to go work in the field, meaning, she used to go in the sun.

I: So what did she do?

R: She didn’t go anywhere. She stayed in the home.

I: So what did she do in the home, like did she do anything with the fruits of your labor in the field?

R: Yes, yes.

I: What did she- rice or grain? What did you bring?

R:Paddy, maize …when it get ripe.

I: You mean the thrashing that people usually do, removing the outer coat? Did she do that at home?

R: Yes. We do not have our own field.

I: They do it in share-cropping right? Like someone has a field, and you are doing it. Was it like that?

R: Yes. It was in dehari

I: Yes, dehari. How much money do you get for one day’s of work over there?

R: [*holds up two fingers*]

I: 200?

R: Yes.

I: I see. And anything to eat along with that? Do they give anything else other than food? Paddy etc.?

R: No.

I: So where did you get this paddy from?

R: Like, if other people did it- we are farmers- we go to farmers, so like we work as dehari and get money. In that when we cut crops they give Dehari, paddy…or anything

I: Why not money though? I’m just wondering, you know, because people generally are preferring money nowadays.

R: we don’t have food.

I: So you can’t buy the food from the money?

R: Buying it…when we do it ourselves, the taste is different.

I: Okay [*chuckling*] so you think that the taste is better when you do it yourself compared to when you buy it? The bought one is from the market and you think that…

R: Yes they put chemicals inside that, that’s why.

I: I see. It has chemicals. And this you have sown yourselves, so meaning, you like it more.

R: Yes.

I: You like the taste more?

R: Yes.

I: Okay very good.

I2: If I were to ask you what is included in living a good life, what things are included in it?

I: Meaning what do you think? That what should I have or what things should I have, how should my environment be, how should my friends be, is there anything like that? Or how should my neighbors be? I said a lot at once, maybe that’s a bit much.

I2: Meaning what is the meaning of a good quality life for you?

R: Meaning we think that, by looking at others we think that if we could study and do a job, we would make such an environment, my friends would come like that, like them.

I2: I see and your mother? Can you say something? What is the meaning of a good quality of life for yourself?

R: She doesn’t want to say.

I: Okay. If you know a little by talking to her, what would you say?

R: She also thinks that her children should study and become like them. That’s a dream for her, but she is never able to fulfil her dream. In the middle, when she started to educate us, she fell sick. I have a small sister; they took her out of school too when my mother got sick.

I: Was she removed from school or did she remove herself?

R: No mummy…[*trailing off*]. She’s small- now, you have to give in the month right?

I: Money?

R: Yes.

I: But there’s also a government school in the village.

R: She studies in the government school, but there’s more of that in the private setup right, that’s why.

I: What do you mean there is more of that in private setup?

R: It is more tight in education [better education]. And in government school…[*trailing off*]

I: So in studying, but it’s better to study in a government school than not study at all?

R: Yes, she studies in the government school.

I: She does study right? So you removed her from private school?

R: Yes.

I: Because you weren’t able to give her fees.

R: Yes.

I: But she is going to the government school now, right?

R: Yes, she is going.

I2: And this happened after the illness, right?

R: Yes.

I2: Anything else? Anything else happen after the illness? What effects did it have on your life?

[*pause*]

I: What was there before and might have changed after the illness, like you mentioned schooling?

R: Because of that…

I: [*interrupting*] But she did not work before either, so what was the shortage in money that you had to bring her? She used to work in the home only, before right?

R: So brother used to also go out sometimes, right? He used to go out. We could maintain fees with that [income].

I: So with her going, how did it affect your brother?

R: He came home, right? Now my brother stays in the house. Who will look after at home? I came here. Then there’s just papa, and he doesn’t go anywhere either.

I: You must not come all the time to [redacted], how many days do you come for? Once per month? How do you come?

R: Had not come before. Since I’ve come, I’ve been here only.

I: Since when? From 30 days?

[*Respondent nodding*]

I: Have they just discharged you?

R: Yes.

I: But you have your father too.

R: Papa doesn’t do any work, because he was also ill before. He has bone TB.

I: Bone TB? So dad stays at home?

R: Yes. Now his doctor has said no to working.

I: Can he walk?

R: Yes.

I: So dad can also take care of the household work, why is the brother home?

R: Brother stays because he says he will not go anywhere until Mummy gets better. That’s why.

I: Meaning, not going means he won’t leave town and go, but within the town…meaning, if you’re here for 30 days, what is the problem with him working? There’s only a small girl at home.

R: Yes…meaning he stays at home. There’s cows and all…

I: So he works at home, no? Tending to the cows?

R: Yes he works at home.

I: So do you sell the milk of the cows?

R: No not now, meaning we don’t sell now. Earlier, we used to sell, now we don’t sell.

I2: Why? Any reason?

R: Meaning, if there’s a little milk, we drink it at home only.

I2: I see. And sorry if I didn’t catch this earlier, but what did your brother used to do? You were saying he went outside right?

R: Yes, brother used to study earlier. His father then fell ill, so he stopped going.

I2: He stopped studying?

I: Because he [the father] has bone TB? That’s why?

R: Yes.

I: Where did he study?

R: In the government setup.

I: In the government setup, meaning in [redacted] only?

R: Yes uh [*hesitating*] he used to study in [redacted] district. Because of that, he left his studies.

I: Because of your father?

R: Yes.

I: So he didn’t leave it on account of your mother, no?

R: No, first he left on account of my father, then…

I: Did he join somewhere after that?

R: Yes, this time too, when we wanted to build a house, we had laid the bricks down, but then mummy fell down with this [HIV-VL], so he left it.

I: Bricks are laid?

R: Yes.

I: So when he left the studies at one time because of father, he hasn’t resumed since then?

R: No, no.

I: So then he didn’t stop studying because of your mother’s illness, right, he stopped it because of your father’s illness.

R: Yes.

I: Right?

R: Yes.

I: Because of her illness, her- your sister went from private school to the government setup?

R: Yes.

I2: And you were telling us about laying bricks? That had stopped after her illness, as you were saying?

I: The house that you wanted to lay, you had said the bricks were laid?

R: Yes, yes. Happened after the illness.

I2: It stopped because of her illness?

R: Yes.

I2: Any other effects after the illness?

R: No no.

I2: Anything like neighbors talking to each other, like were people talking to you? Used to talk before, don’t talk anymore, anything like that happen?

R: No. They talk now too, they used to do it before too.

I2: They do it now too, and used to before as well. And have you felt anything different since the illness?

R: When she fell sick, she thinks more about how her grandmother does not come to look after her. They don’t talk to my father, they are angry with my father.

I2: Why?

R: This is from the past, when I was small…

I2: I see, I see. It doesn’t have to do with the illness?

R: No. That’s why they don’t come, that’s why thinking all this.

I2: And the neighbors know what illness she has?

R: The people in my village don’t know that much.

I2: They don’t know. Only that she’s ill?

R: They just know she has Kala Azar. That’s it. They do not know about her HIV.

I2: Do you know what the people from the village will think if they found out about HIV? Or you, if you ever find out that someone else in the village has HIV…

R: only one or two people knows that but they don’t understand

I2: They don’t understand it? So it doesn’t affect it?

R: No.

I2: And people…meaning, they don’t think anything about the Kala Azar either?

R: No.

I2: Okay. Okay, okay, okay.

[*pause*]

I: So when you found out she had both diseases, how did you helped her to cope with it? What- what was your first reaction?

R: [*long pause*]

I: Meaning, it happens no that you listen and you first try to find out more. So what did you think about this? Did you know what kind of illness it was?

R: [*pauses*] I did not know.

I: So how did you tell everyone in your house?

R: My brother has a friend. He’s the one who said. He told me too, and he told her too.

I: He told them both?

R: Yes, because the doctor was the one who told us, but he [the doctor] only told him that she has HIV.

I: I see, the doctor hadn’t told you two either?

R: No…because if we knew, we would get worried- that’s why they didn’t tell us. We found out later- doctor…I asked the new doctor why did you hide the illness from us? He said if I would have told you, then you would link it to…something else, that’s why I didn’t tell you all.

I: Did he tell anyone else? Mukhiya or anyone?

R: Yes, they…they also probably don’t know that much? He just knows there is Kala Azar. Mukhiya anyway knows there is Kala Azar- he does not know what HIV is.

I: Does the Mukhiya know she has HIV?

R: No…I don’t know, I don’t know whether he knows or doesn’t know.

I: So how- he must have explained to her, no?

R: She did not want to come, that’s all he explained. What did he say?

I: Only the Mukhiya? There must be other people besides the Mukhiya?

R: No. Some staff had gone from [redacted], so they went and did…

I: Meaning the Mukhiya knows she has Kala Azar?

R: Yes.

I: He doesn’t know she has HIV?

R: Yes.

I: I see. And who told the Mukhiya?

R: The staff went from here, so…well, they used to call us to tell us we have to go to [redacted], but we did not want to come. So the private doctors, I mean, the [redacted] staff called the [redacted] staff. So they combined and went to our house.

I: Okay, those staff went to your house?

R: Yes.

I: Was she ready to go then?

R: Meaning, after they explained, then…got ready to go.

I: So if she got ready to go, what was the need to tell the Mukhiya?

R: We did not believe it earlier. Mummy used to say that until she gets some evidence, we will not go to [redacted]. Suppose something happens, then? She used to think like that, that’s why.

I: Then when the Mukhiya explained-

R: Yes.

I: She thought it was okay to go. Okay. Okay, you…we were talking about a good quality life. So, what kinds of things- like you said education, kids study and get settled.

R: Yes.

I: Or like you said you wanted to make a good house.

R: Yes.

I: So these things, why are they important…for a good life?

R: [*long pause*] I cannot say that.

I: No think a bit. Why is it important to study and get a job? You said right, that…

R: Because we do not want to go out in the sun, like…

I: You don’t want to go out in the sun, I see.

R: Yes.

I: This is a very good thing right, like now you work in the field.

R: Yes.

I: Okay so that’s why you want to study, so you don’t have to go in the sun? What do you think, that people who study do not go…in the sun?

R: [*smiling*] It’s not like that. But like we go out in the sun, they don’t go out- do they?

I: No, absolutely, you’re totally right. Okay. Anything else? Anything else you feel?

R: If we study- if we study and become something, then our coming generations should also do the same thing.

I: I see. So the future generations should also be able to live that kind of life?

R: Yes.

I: I see. And regarding family life? Should have a good family? What do you think about this?

R: [*long pause*] That our family should live happily and properly, that’s it.

I: So is that important for a good life?

R: Yes.

I: Health? How important is health for a good quality life?

R: She thinks that…meaning…my kids and all, all of them, will see me and be happy that there is nothing bad happening in my body, meaning not happening from the illness. That whoever is in my family, sons and daughters, everyone gets the medicine, then…

I: Yes, I mean, is good health important for a good quality life?

R: For the body, you are asking? She wants good medicine, good food…

I: Great. Anything more?

R: [*long* *pause*] That’s it.

I2: You were mentioning family life earlier. Have you noticed any difference in this since the illness? During the illness or after the illness?

R: Meaning how we were before and how we are now?

I2: Yes, yes.

R: Meaning everyone in the house is less worried now than they were before. Now, everyone believes that nothing will happen.

I2: I see, I see. Can you tell me a bit about the treatment? How she felt during the illness? And what all happened? What all treatments were done?

R: After coming here?

I2: Hmm, hmm. Regarding the 30 days you spent here- a little bit about that. How was the treatment?

R: The treatment went very well. Treatment, I mean, before the treatment, she used to feel very weak, could not even walk, could not even eat. But after coming here, slowly slowly- she used to eat little food also- now, since medicine has started, she has started eating more and she has started walking…she didn’t talk to anyone either, now she has started talking to people- laughing.

I: I see, I see. This just happened?

R: Yes.

I: She was laughing and all?

R: Yes. She didn’t even talk to people before. Walk- while walking, she used to feel dizzy even if she walked.

I: Before illness, everything was okay with her?

R: Yes.

I: Did she used to talk then? Before she got a fever?

R: Yes.

I: So she used to laugh?

R: Yes.

I: After the illness, then? She had stopped laughing?

R: Yes, meaning she did not feel like talking.

I: Didn’t feel like it, I see. And the work she used to do at home?

R: After her illness, I was the one who did all the housework.

I: What did she do then?

R: Nothing.

I: Was not able to do anything.

I2: And the staff that were there, did they explain the illness to you? Like it happens like this, why this happened, did they ever explain like this to you?

I: In [redacted].

R: Meaning, about HIV?

I2: Yes. HIV and Kala Azar, about both.

R: Meaning, HIV, how it came to us…so they asked us does your father have it, so I said my father doesn’t have it. She got a blood test done, not my father. They asked had I gone to a private setup anywhere to get a blood transfusion…so we said yes. But we were told how Kala Azar is contracted. There are cows and all in the house, and the cow dung and we make fuel from that .You call it a sandfly, right?

I: Yes.

R: It is transmitted by its bite. When it bites? From 11 to 3. Bites in the night, you are not able to see by the morning, right? When…if it bites you once, you, I mean, you feel like you have a slight fever, you come and eat medicine, but after eating that, you don’t get a fever- the fever just remains inside. Slowly, we understand that weakness…we are not eating and feeling heaviness on one side. Our body is slowly becoming weaker, and then we understand that yes she is sick. Then also we don’t know what happened [36:48-36:50]. When you have a cow and it poops, it should not be kept at the side of the house. From that, the sandfly…in the cold, the sandfly is there.

I2: Who told you all this?

R: [redacted] sir.

I2: [redacted] sir. I see. And is it important to you that someone explains all this to you at the place where you come to seek treatment?

R: Yes.

I2: Has that ever happened before- that staff are telling you- this is how it happened?

R: No.

I2: Never?

R: No.

I2: I see.

R: We were told that when you go from here, we should tell the other villagers how they can avoid getting this illness. We said Sir we can clean the area around our house, but if they…if we throw something on the side, then what will we do? Do we tell them don’t throw here, so he said that you should do it yourself. If they don’t want to do it, tell them we can help them- clean it up together, since it’ll good for us also and for them also.

I: So whatever you have felt during the illness for the time you have been here, do you feel anything like- do you feel like if this thing was done differently it would be better or anything like that?

R: [*shaking her head*]

I2: You can speak openly haha.

I: Speak comfortably.

R: No no, I feel nothing like that. It’s good for me. And mummy says she has never seen a hospital like this. Nothing like this anywhere. Have never seen a government hospital like this with so many facilities, so clean and tidy, but after coming here, it doesn’t feel like I’m in a hospital.

I: How is the behavior of the staff who give you the medicine?

R: It is very good- very good.

I2: Any trouble at all?

R: No.

I2: Okay. Tell me this- what do you want from life moving forward? What are your dreams?

I: Meaning, what are you thinking now?

R: We think that after going away from here- go outside and finish education. Mummy also thinks that we will go from here- now I am alright- now even if I don’t work outside, I can do some housework. To go and do housework…so I can finish my education.

I: Whose education? Yours?

R: Yes.

I: And your brother?

R: He has left it now, he will not study anymore…and my younger sister.

I: So what else does she think about her future- your mother- ask her please? What things are left that she would like to do?

R: She thinks that…home, I mean work, just…just wants to do some housework. She does not wish to go back out in the sun [to work] now.

I: I see. Meaning, what used to think earlier about your future- has there been any chance in this since the illness and treatment? So have there been any changes in her thoughts since then?

R: [speaking for the Mother after translating] There is a little change from before, earlier I used to worry a lot- how will I survive with this illness, how will I stay in my family, but after coming here, I am not thinking like this. Whatever I have, I think I will complete my children’s education, they will grow up- this change is there.

I: So this is after treatment. Did she feel anything after learning about the illness? Like when she found out she had HIV and Kala Azar?

R: Earlier, she had gotten worried when she first heard about the illness.

I: She had gotten worried?

R: Yes.

I2: Tell us a bit more about this.

R: She found out that, when we went, we were told that we could not get treatment done in the private setup, because a lot of money is required for this treatment. Government- mummy used to think where will we get all this money from? She was very worried. How will my treatment be done? What should we do? That’s how she used to think, but after coming here, she got treatment and now she thinks- nothing like that anymore, now that I’m better, whatever I had thought about education for my kids will be completed. It doesn’t feel like this illness will now…[*trailing off*] because now, she has been told how the illness spreads to another person. If she does the blood herself, so it happens from the blood- HIV. If her blood, if she does everything by herself, then it won’t go to anyone else. She did not know all that before.

I: What else should she not do that it wouldn’t go to somebody else? Did they say anything else like that?

R: [*long pause, looking uncertain*] Nothing.

I2: Would you like to say anything else? About your life? Anything we haven’t talked about?

I: Just one thing I wanted- what you said about the illness…before starting the medicine and after the medicine started- whereas I wanted to know the difference in her life before finding out about the illness and after finding out about the illness. Because till now, you told me what she used to think before illness- meaning until medicine had not started- so if we don’t talk about medicine and talk about when she did not know about the illness, then what did she think? And when she found out about the illness, then? Was there any change in how her life or how she thought about her life?

R: [*long pause*] No before starting medicine I just used to think how will I get better, that’s it, however I get better, that’s it. Now that the medicine has started here, I think everything will get better, like before. Earlier, I used to not feel any hunger at all- now I get so hungry.

I2: How much did you eat before? How much do you eat now?

R: Earlier, I used to eat very little. She didn’t even eat roti. Now she eats properly. Now I require food 3 times a day.

I: Great 3 times a day. That’s very good.

I2: Yes it’s very good. One last question- earlier, you had mentioned the private setup right? Was this a big hospital or a little private corner shop?

I: Like there are doctors who have opened something small in a corner- was it like that or was it a hospital?

R: It was a big hospital. But in the government setup, it’s like we don’t know- if we catch someone- like in the village, the jhola jhaap as you say- we told him we don’t know which doctor and how they are, so you take us. So he is the one who took us.

I: So he is the one who took you to the hospital?

R: Yes, yes.

I: You did not go on your own?

R: No.

I2: Jhola jhaap doctor had taken you to the private hospital?

R: Yes.

I: And the private hospital told you the treatment won’t happen here- now you go to the government hospital? Who told you about the government setup?

R: No. There they only told us about seasonal illness, they did not tell us any other illness besides that.

I: Who told you about the government hospital? How did you get to [redacted]?

R: An ASHA told us. She only took us.

I: An ASHA, I see I see. You must have talked to the ASHA? How did the ASHA know?

R: Because she had already taken a lot of people to get treated for Kala Azar at the government hospital. They have gotten treated and come back, just for Kala Azar.

I: But she was told you have seasonal illness? That’s what she must have been told, right?

R: Yes, just that I get fever, seasonal fever, getting no relief from the medicine…

I: This is what was said, and then she took you to the government hospital?

R: Yes.

I2: And from there then?

R: Yes. And the face used to look a little black in appearance. I had gone all black.

I2: Alright then. Thank you very much.
